# Evaluation of fully-functionalized diazirine tags for chemical proteomic applications†

**DOI:** 10.1039/d1sc01360b

**Published:** 2021-05-07

**Authors:** Louis P. Conway, Appaso M. Jadhav, Rick A. Homan, Weichao Li, Juanita Sanchez Rubiano, Richard Hawkins, R. Michael Lawrence, Christopher G. Parker

**Affiliations:** Department of Chemistry, The Scripps Research Institute Jupiter FL USA cparker@scripps.edu; Research and Development, Bristol Myers Squibb Company Princeton NJ USA

## Abstract

The use of photo-affinity reagents for the mapping of noncovalent small molecule–protein interactions has become widespread. Recently, several ‘fully-functionalized’ (FF) chemical tags have been developed wherein a photoactivatable capture group, an enrichment handle, and a functional group for synthetic conjugation to a molecule of interest are integrated into a single modular tag. Diazirine-based FF tags in particular are increasingly employed in chemical proteomic investigations; however, despite routine usage, their relative utility has not been established. Here, we systematically evaluate several diazirine-containing FF tags, including a terminal diazirine analog developed herein, for chemical proteomic investigations. Specifically, we compared the general reactivity of five diazirine tags and assessed their impact on the profiles of various small molecules, including fragments and known inhibitors revealing that such tags can have profound effects on the proteomic profiles of chemical probes. Our findings should be informative for chemical probe design, photo-affinity reagent development, and chemical proteomic investigations.

## Abstract 2

The chemical proteomic properties of five diazirine-based, fully-functionalized photoaffinity tags, including a newly developed, minimal tag, were compared. This study provides guidance for the development of new photoaffinity probes.

## Graphical abstract

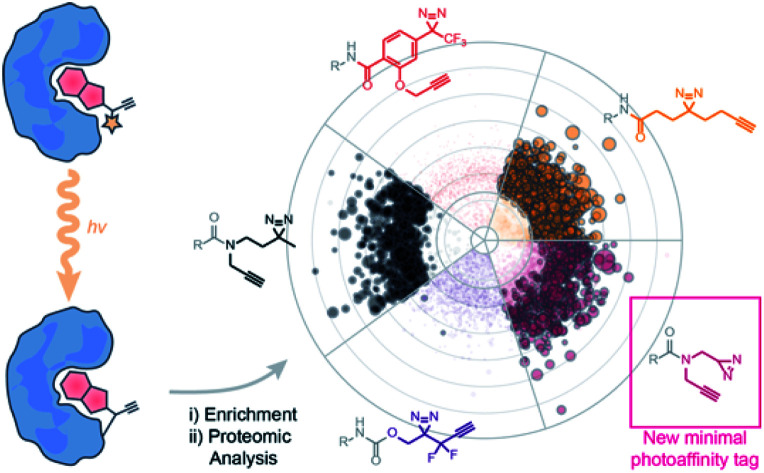

## Introduction

The mapping of non-covalent small molecule–protein interactions is a core objective of chemical proteomics. The ability to selectively tag proteins for downstream applications in native environments has dramatically enhanced our ability to investigate biological phenomena, design new chemical probes, and illuminate the mechanism of action (MOA) of bioactive small molecules. Chemical tools that enable such investigations are typically composed of a (1) molecular recognition group to engage a subset of the proteome; (2) a reactive group (*e.g.* photoreactive or electrophilic) to covalently capture engaged protein targets; and (3) a reporter group (fluorophores, affinity groups, or chemically reactive handles like alkynes or azides) for the detection, enrichment, and identification of captured proteins.^1^ Common photo-activatable groups include aryl azides, benzophenones, diazirines, and most recently, 2-aryl-5-carboxytetrazoles (ACTs),^2–4^ which upon exposure to a light source, form reactive intermediates that can covalently react with neighboring proteins or other molecules. Once covalently adducted, bound protein targets can be detected through a variety of methods, however, the most often utilized functionalization method is conjugation to reporters *via* copper-catalyzed azide-alkyne cycloaddition (CuAAC) reactions.^1,5^

Traditionally, reactive and reporter groups are embedded in the bioactive molecule (molecular recognition group) or appended separately, in such a way as to minimize perturbation to bioactivity. Such strategies can often require exploring various positions (structure activity relationship or SAR), which themselves can necessitate new synthetic routes, often requiring laborious efforts to achieve minimally invasive placement. Recently, the development of “fully-functionalized” photoaffinity tags has yielded a versatile alternative. Fully-functionalized (FF) tags contain a photoreactive group, reporter group, and a synthetically functionalizable handle that enables late-stage derivatization of small molecules often in one synthetic step. Diazirines are widely utilized photoreactive groups and are routinely employed in FF tags, likely due to their relatively small size, and therefore minimized proteomic interactions and physicochemical perturbations, as well as their irreversible and efficient photoactivation.^6,7^ Diazirine-based FF tags have been used to deconvolute the targets of drugs,^8–10^ phenotypic screening hits,^11–14^ as well as metabolites,^15,16^ and have also been employed for fragment-based ligand discovery in cells.^17,18^ Despite their utility, attachment of such tags can have consequential effects on the molecular interactions of the parent molecule, potentially convoluting proteomic analysis and even altering biological activity. Previous studies have compared the efficiencies of different classes of photo-affinity groups (*e.g.* benzophenone, aryl azides, and diazirines) against purified proteins with established ligands.^19,20^ Additional studies have inventoried relative background interactions of various photoreactive groups in cells^21,22^ and explored the utility of different bioorthogonal reactive groups.^23^ More recent investigations have led to a better understanding of amino acid reactivity preferences, as well as mechanistic insight into diazirine photo-induced reactivity, which includes the formation of a reactive carbene, ylides, and electrophilic diazo intermediates.^24–27^ However, the impact of FF tags on the proteome-wide profiles of molecular recognition groups has not been evaluated. Herein, we directly compare and characterize five diazirine-based FF tags containing dialkyl and aryl diazirines as well as a newly reported ‘terminal’ diazirine for their abilities to map small-molecule protein interactions in cells.

**Fig. 1:**
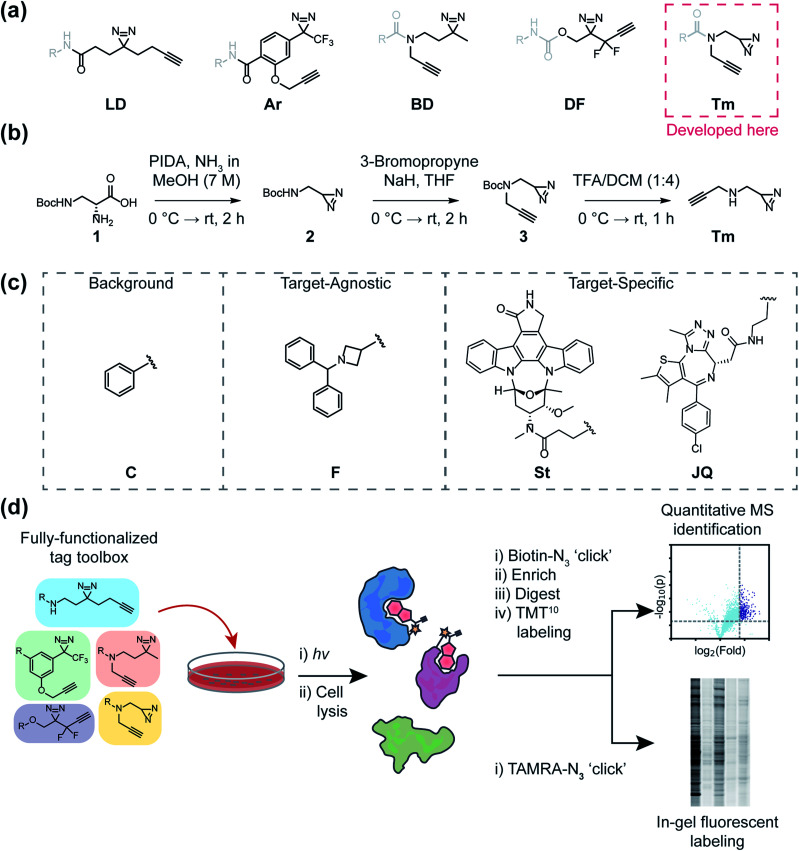
A general outline of this study. (a) Structures of fully-functionalized (FF) tags (black), which contain a clickable alkyne handle, diazirine and functional group for conjugation to molecular recognition group (gray). (b) Synthetic scheme of the newly developed terminal diazirine FF tag. PIDA: phenyliodonium acetate (c) Structures of probe libraries (i) designed to serve as control probes to assess background protein targets (‘C’ series) of each FF tag, (ii) fragment-based probes to compare tag effects on general proteomic interactions and site-mapping (F series), or (iii) target-specific probes (staurosporine-based ‘St’ and JQ1-based ‘JQ’ series) to evaluate each FF tag for target ID. (d) Schematic workflow for evaluating FF tags in human cells through both gel-based fluorescence and MS-based proteomics.

One of the most frequently used FF tags is a ‘minimalist’ dialkyl diazirine (Fig. 1a, ‘LD’) developed by Li *et al.* in effort to reduce the overall size of capture and enrichment group.^28^ A branched tag (Fig. 1a, ‘BD’),^29^ was chosen to be included in our study to assess whether a less embedded diazirine might improve its properties as a photoaffinity tag. As trifluoromethylaryldiazirines are proposed as efficient reagents for labeling proteins with minimized side products,^7^ we selected a fully functionalized derivative (Fig. 1a ‘Ar’).^30^ A compact difluoroalkyldiazirine tag (Fig. 1a ‘DF’) was recently developed and was chosen as it is proposed to have enhanced photo-reactivity driven by a proximal difluoro group.^31^ Finally, we report here the synthesis of what is to our knowledge the smallest diazirine-based FF tag (Fig. 1a ‘Tm’) and assess its suitability for chemical proteomics studies. For these investigations, we generated a library of 20 FF probes designed to assess overall reactivity, to compare the impact of each tag on the profiles of small molecule probes, and examine the relative utility of each tag to identify the targets of established inhibitors. In these studies, we demonstrate that FF tags have varying proteomic reactivities and can substantially influence the interaction profiles of small molecules. This work provides new insight and guiding principles for chemical proteomic investigations utilizing diazirine-based FF tags, and introduce a useful new minimalized tag to the photo-affinity toolbox.

## Results

### Development of new ‘minimalist’ fully-functionalized tag

Ideal features of FF tags are high capture efficiency, synthetic accessibility, as well as minimized physicochemical effects (*e.g.* solubility, cell membrane permeability) and background interactions. Although aryl diazirine photoreactive groups have historically been used across a variety of applications, most FF tags possess aliphatic dialkyl-diazirines, with some variations on tag length, branching, and neighboring substitutions. Considering these points, we designed a new ‘minimalist’ FF tag that incorporates a terminal diazirine (Fig. 1a),^32^ which we hypothesized would have fewer background interactions relative to larger dialkyl diazirine tags. This tag is synthesized in three steps starting from commercial *N*-β-Boc-d-2,3-diaminopropionic acid (Fig. 1b) in a combined yield of 35% and it possesses a relatively small molecular weight of 109 Da.

In order to assess the utility of diazirine-based FF tags, we designed a series of diazirine-based FF probes composed of three sub-libraries to explore (1) relative background proteomic interactions of each tag; (2) the impact of each tag on the target–agnostic profiles of small molecule fragments; (3) the relative utility of each tag to identify established targets of the broad kinase inhibitor staurosporine and the BRD4 inhibitor JQ1 (Fig. 1c).

### Photolytic decomposition kinetics of FF tags

**Fig. 2:**
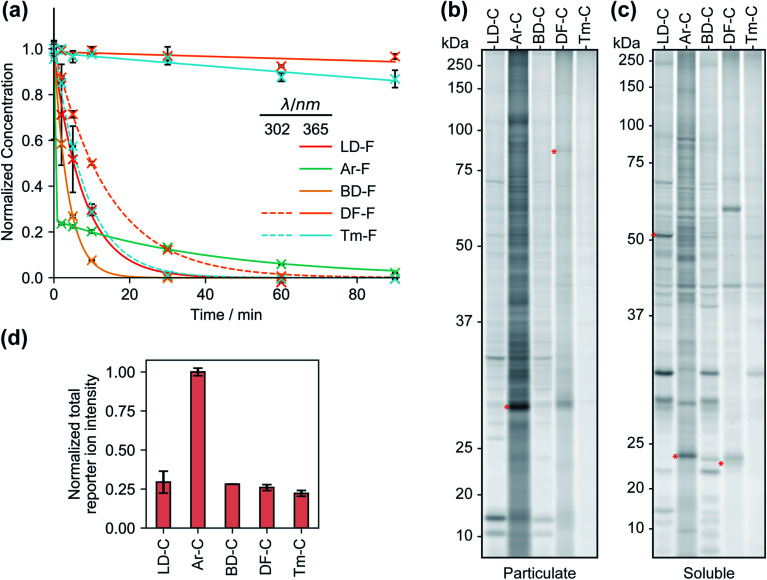
The kinetics of probe photoactivation and profiles of control probes C1–5. (a) The decomposition of the fragment-based (‘F’) probes upon irradiation with UV light was followed by LC-MS. Tags LD, Ar, and BD are photoactivated at 365 nm, while probes DF and Tm require irradiation at 302 nm. Data represents average values ± SD; *n* = 3. (b) Gel-based profiling of control probes (20 μM) in human cells. HEK293T cells were treated with control probes (20 μM) photocrosslinked and lysed, and proteomes were conjugated to an azide-rhodamine tag and analyzed by SDS-PAGE and in-gel fluorescent scanning. Shown are probe-labeled particulate (membrane) (b) and soluble (c) fractions. (d) Relative TMT reporter ion intensity for each control probe (20 μM) in HEK293T cells. Error bars represent standard deviations from two replicates.

We first compared the reaction rates of each FF tag upon irradiation at 365 nm in organic solvent *via* liquid chromatography-mass spectrometry (LC-MS). We observed first-order reaction kinetics for dialkyl diazirines LD–F and BD–F with half-lives of 5.4 and 2.6 min, respectively (Fig. 2a and S1†). Irradiation of aryl diazirine Ar–F appeared to result in the rapid consumption of ∼70% of the diazirine in less than 2 min, followed by a slow first-order decay (*t*_½_ of 29 min). Notably, the analogs based on the recently reported difluoromethylalkynyl (DF–F) and terminal alkyl diazirine (Tm–F) FF tags showed little photoactivation (<10%), even after 90 min of irradiation. Examination of the absorbance spectra of each FF tag revealed that diazirines LD, Ar, and BD have absorbance maxima at ∼360 nm, however, the DF and Tm tags possess shifted absorbance maxima of 300–310 nm (Fig. S2†). Indeed, irradiation at 302 nm resulted in the decay of probes DF–F and Tm–F with first order reaction kinetics (*t*_1/2_ of 10 min and 5.8 min, respectively). Reports suggest that aryl diazirines can form stable diazo intermediates, which can be subsequently converted to reactive carbenes upon irradiation at shorter wavelengths.^33^ Considering this, we hypothesized that the remaining fraction of unreacted Ar–F could actually be the isomeric diazo species, and indeed, after an initial exposure to 365 nm UV light, we observed rapid depletion upon irradiation at 302 nm (Fig. S3†). Together, these studies suggest that groups adjacent to the diazirine can affect optimal irradiation conditions for diazirine activation.

### Proteomic reactivity of FF tags

We next qualitatively assessed overall proteome interaction profiles for each FF-tag using established SDS-PAGE methods.^17,18^ For these studies, we utilized the ‘C’ series of control probes (LD–C, Ar–C, BD–C, DF–C, and Tm–C) wherein the molecular recognition group is substituted with a phenyl ring using similar amide linkage chemistry. We rationalize that the use of a shared, structurally minimized molecular recognition group enables relative assessment of the ‘background’ interactions for the tag itself.^17,18,21,28^ In brief, we treated HEK293T cells with each probe (2, 10, and 50 μM, 30 min) followed by exposure to UV light (365 nm for LD–C, Ar–C, and BD–C and 302 nm for DF–C and Tm–C, 15 min), harvesting, lysis, coupling of probe-modified proteins to an azide–rhodamine reporter tag using copper-catalyzed azide–alkyne cycloaddition chemistry (CuAAC),^5^ and visualization of these proteins by SDS-PAGE and in-gel fluorescence scanning (Fig. 2b, c and S4†). We observed marked concentration-dependent protein labeling by all FF tags, both in the cytosolic and membrane fractions, with substantially fewer labeling events by DF–C and Tm–C compared to LD–C, Ar–C, and BD–C. Notably, Ar–C, labeled membrane proteins to a greater extent compared to other probes. To determine whether the extensive membrane protein labeling by the Ar tagged-probe was a result of preferential labelling of membrane proteins or perhaps due to poor membrane penetration, the probes were tested in fractionated cell lysates and analyzed by fluorescence gel. This experiment resulted in a marked decrease in membrane protein and an increase in soluble protein labeling (Fig. S5†), suggestive of Ar–C having reduced permeability relative to the other tags. In agreement with our previous kinetic experiments, irradiation of DF–C and Tm–C at 365 nm resulted in little to no proteome labeling (Fig. S6†), even at extended UV exposure times (Fig. S7† and 2a). The lack of proteomic labeling by DF–C/Tm–C despite similar reaction kinetics as LD–C/BD–C might be suggestive of competing intramolecular reactions for these tags. Indeed, mass spectrometric analysis of solutions of the ‘F’ series of fragment-based probes after irradiation in isopropanol for 90 min indicated the formation of intramolecular reaction byproducts from all the tested diazirine tags. In the case of Ar–F the only intramolecular reaction observed is the presumed rearrangement to form the isobaric stabilized diazo intermediate, corresponding to a small change in LC-MS retention time. The remaining probes all showed evidence of the formation of olefin by-products, consistent with a recent study which demonstrated that dialkyldiazirines form significant quantities of undesirable side-products through rearrangements of intermediate carbenes and diazo compounds (Fig. S8–S12†).^24^ LC-MS peaks corresponding to intermolecular reaction (capture of solvent) were observed for all probes with the exception of DF–F.

We next evaluated each control FF probe in cells by quantitative MS-based proteomics to directly compare the relative amounts of proteomic interactions of each tag. HEK293T cells were treated with individual probes (20 μM, 30 min) and then exposed to UV light to induce photocrosslinking of tag-bound proteins. Cells were lysed, and the tag-labeled proteins were conjugated to an azide–biotin tag by CuAAC chemistry, enriched by streptavidin and trypsinized as previously described.^18^ Tryptic peptides were then treated with TMT reagents (10plex) enabling direct and quantitative comparisons to be performed in a single MS experiment.^34^ Comparisons of the total reporter ion intensity stemming from each probe revealed that Ar–C exhibited a much greater degree (∼4-fold) of background proteome interaction relative to the alkyl diazirine and difluoroalkyl tags, which had similar reporter ion intensities (Fig. 2d).

### Tag effects on proteome-wide small molecule profiles and probe mapping studies

**Fig. 3:**
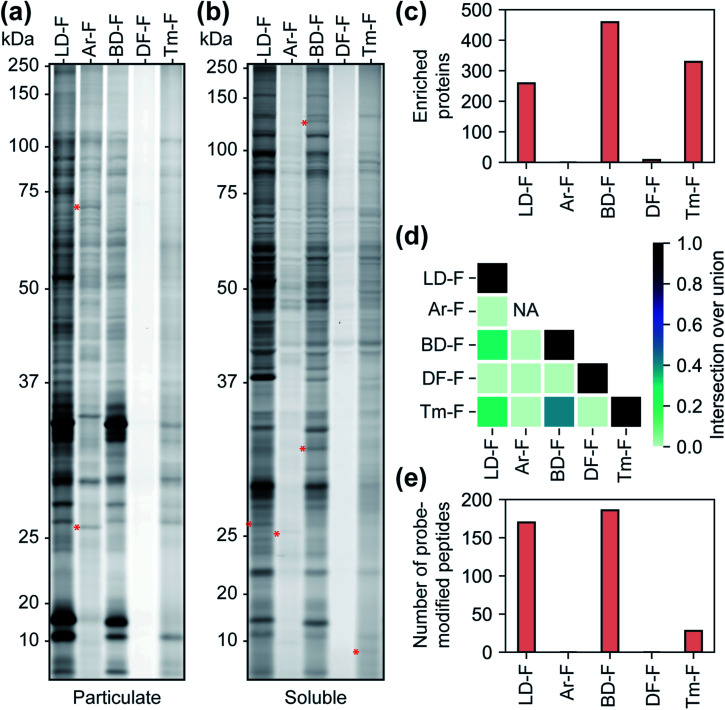
Characterization of fragment-based (‘F’) probes. (a) SDS-PAGE analysis of fragment probe binding to the particulate (membrane) proteome fraction and (b) the soluble proteome fraction. Red asterisks indicate examples of labeled proteins unique to each probe. (c) Number of proteins enriched by each fragment probe relative to corresponding control probe (>3-fold; *p* < 0.05 from two replicates). (d) A heatmap representing the intersection to union ratio of proteins enriched by each fragment probe. (e) The number of probe-modified peptides found to possess both isotopically ‘heavy’ and ‘light’ modifications.

We next sought to assess the influence of each tag on the proteomic profiles of small molecules. For these studies, we first compared the profiles of fully-functionalized fragments (FFF).^17,18^ We chose FFFs for this analysis as they generally have broad proteomic coverage, enabling comparisons across a large subset of proteins. Further, FFFs do not have well-defined or established functional targets, allowing us to bypass any complicating effects that the tags may have on bioactivity. Initially, we compared the interaction profiles of each of the five diazirine tags by fluorescence gel analysis. The fragment series (F) revealed a similar profile to the corresponding control probes in terms of the relative levels of proteome labelling (Fig. 3a, b and S13†), with the Ar-tagged fragment exhibiting more labeling of membrane proteins. The Df/Tm tags had substantially less overall labeling compared to LD/Ar/BD. We also noted many unique labelling events for each FFF, suggestive that tag structures have influence over the identity of proteins crosslinked (Fig. 3a, b and S13†). We next profiled the interactions of each FFF (200 μM, 20 min UV irradiation) alongside each corresponding control probe following the TMT workflow described above. For this analysis, a protein was designated as a FFF target if it was enriched >3-fold over its corresponding control across biological replicates. Very few targets were enriched by probes with the Ar or DF tag (Fig. 3c), while similar levels of targets were enriched by FFFs LD, BD, and Tm. Notably, we observed the greatest degree of target overlap between F probes with BD and Tm tags although the pairwise intersections between proteins enriched by LD–F, BD–F, and Tm–F were all substantial (Fig. 3d). Taken together, these data imply that Ar diazirine tags have substantial background interactions relative to their dialkyl diazirine counterparts while the DF tag might have limited protein capture efficiency compared to the LD, BD, and Tm tags, potentially due to competing intramolecular reactions (Fig. S10†). We also noted that while Tm appears to have relatively lower overall proteome labelling compared to LD–F and BD–F (Fig. 3a and b), we observe comparable numbers of enriched targets, likely due to fewer background interactions of the tag itself (Fig. 2b–d).

Photo-affinity tags can often be used to provide small molecule binding site information through the identification of probe-modified peptides. We next assessed the relative utility of each tag to map the site of photolabeling using our previously described protocols.^17,18^ In these experiments, following treatment with the F-based probes, irradiation and lysis of cells, captured proteins are conjugated to an isotopically labeled (‘heavy’ and ‘light’) dialkoxydiphenylsilane (DADPS) tag derivative (see ESI biological methods section m†),^35^ enriched, trypsinized and probe labeled peptides are subsequently eluted after acid-cleavage. Overall, we confidently identified (peptides carrying both heavy and light modifications) 139, 154, 28 labeled peptides for probes LD–F, BD–F, and Tm–F respectively (Fig. 3e). No peptides were found to be confidently labeled with tags Ar–F and DF–F. In the case of Ar, this could be potentially result of observed fragmentation of the parent tag (Fig. S14†), while overall poor capture efficiency could be responsible for the lack of labeling by the DF tag. Together, these data suggest LD and BD tags to be efficient capture tools to map probe-modified peptides.

### Tag effects on target-specific probes

Having assessed relative background profiles as well as effects of each tag on proteome-wide interactions of small molecule fragments, we next evaluated the relative ability of each tag to identify targets of established inhibitors in chemoproteomic experiments. For these studies, we prepared two series of FF probes: one series (St) is based on the broad-spectrum kinase inhibitor staurosporine and the other (JQ) on JQ1, a potent inhibitor of the BET family of bromodomain proteins. All five FF tags were attached to staurosporine and JQ1 at previously reported and well-established linkage sites (Fig. 1c).^2,28,36^ To determine how the attachment of each tag affects the activity of the parent compounds, we performed an *in vitro* kinase inhibition assay for staurosporine-derived probes (Fig. S15†) and cell proliferation assays for JQ1-derived probes (Fig. S16†). For the staurosporine series, nearly all FF-probes showed modest reduction in inhibitor activity, except for the Ar-based probe, which showed negligible inhibitory activity at the tested concentrations. All probes in the JQ series demonstrated antiproliferative activity with IC_50_ values within an order of magnitude of that of JQ1 itself. We next compared the ability of each probe series to label proteins upon photo-crosslinking. Both staurosporine and JQ1-derived probes showed concentration-dependent labeling of proteins (Fig. S17 and S18†), however, in line with previous reports on staurosporine-based probes, the St series were not membrane permeable,^37^ and so were tested in cell lysates, while JQ1 probes labeled both cytosolic and membrane proteins in live cells.

**Fig. 4:**
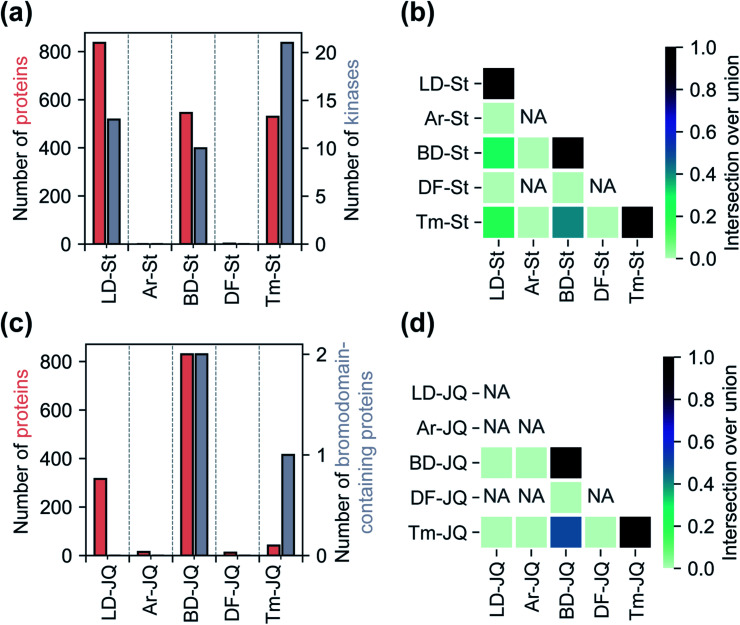
Analyses of the target-specific staurosporine-based ‘St’ and JQ1-based ‘JQ’ probe series. (a) A comparison of the total number of proteins and the number of kinases enriched by each staurosporine-based probe. (b) Heatmap illustrating the degree of mutual protein enrichment by different staurosporine-based probes. (c) Total number of proteins and bromodomain-containing proteins enriched by JQ1-based probes. (d) Heatmap illustration of the degree of overlap between the sets of bromodomain-containing proteins enriched by each JQ1-based probe. Enrichment defined as >3-fold enhancement of normalized abundance over control probe, *p* < 0.05 from two replicates.

We next sought to assess the impact of each tag on the overall proteomic interactions of staurosporine and JQ1 as well as their ability to identify established protein targets *via* quantitative MS-based proteomics. For the staurosporine series, K562 whole cell lysates were treated with 20 μM of each probe or corresponding control probes and processed as described above. Potential targets were considered to be those proteins enriched >3-fold above the corresponding control probe and following the same statistical criteria as described above. Using these criteria 836, 545, and 529 total proteins and 13, 10, and 21 kinases were identified for probes LD–St, BD–St, and Tm–St, respectively (Fig. 4a). Kinases enriched by all three of these probes included well-established staurosporine targets PRKACA, CDK2, and RPS6KA1, which have been previously been reported to bind staurosporine with *K*_d_ values of 19, 7, and 43 nM, respectively.^38^ Notably, only two protein targets (and no kinases) were substantially enriched over control probes by DF–St and no proteins were enriched by probe Ar–St. Interestingly, although probes LD–St, BD–St, and Tm–St enriched several of the same kinases, we observed at most 41% overlap of enriched kinases (between BD–St and Tm–St) (Fig. 4b). We performed similar profiling experiments with the JQ1 probe series, finding that BD-JQ enriched BRD3 and BRD4 while Tm-JQ, although enriching relatively few proteins in total, did also enrich BRD3. Once again, we observed the Ar- and DF-tagged probes to enrich very few protein targets, and no BRD-containing proteins over their corresponding controls. Taken together, these results suggest that tag choice can have profound impact on the overall proteomic profiles but also can influence the capture efficiency of targets even within the same protein class.

## Discussion

In summary, we systematically evaluated a panel of fully-functionalized, diazirine-based capture reagents for chemical proteomic applications. Included in this panel is a newly reported, ‘minimalist’ terminal diazirine FF tag. We found that diazirines within each tag, depending on their neighboring groups, possess varying activation wavelengths and photo-reactivity. Consistent with previous studies,^21,22^ we also found that tag structure has a substantial impact on overall background interactions, for example, we observed that Ar–C labels substantially more proteins compared to the alkyl diazirines. It has previously been reported that a drawback in the use of alkyl diazirines is rearrangement to form metastable diazo species that react with nucleophilic protein side chains rather than undergoing insertion reactions, increasing the radius of labeling.^24^ The diazo side product resulting from irradiation of the aromatic diazirines, in contrast, has been considered to be virtually unreactive and is thus expected to produce reduced background labeling.^39^ The high degree of background labeling that we have observed here, coupled with kinetics studies showing the clear albeit slow reaction of the diazo side-product resulting from irradiation of the Ar–F (Fig. 1a) suggests that the diazo product may drive increased non-specific capture events relative to other tags.

In contrast to the Ar tag and in agreement with previous reports, we have found that all the alkyl diazirine tags tested in this study are prone to intramolecular rearrangements to form unreactive side-products.^24^ In the case of the DF-photoaffinity probe the formation of undesirable side-products may occur to such a degree no products of intramolecular reactions are detected in our decomposition experiments (Fig. S11†). Further, MS- and gel-based proteomic analyses do show that the DF tag is capable of capturing proteins, but with substantially less efficiency than the other photoaffinity tags tested. We note that both DF and terminal diazirines were previously reported to be activated at ∼365 nm,^31,32^ however, we observed shifted absorbance maxima relative to the other diazirines. Likely, UV sources used in these previous studies possessed broad spectrum bulbs or filters. Our observations highlight the importance of characterizing new photo-affinity reagents in order to maximize crosslinking efficiencies and they also suggest that electronic substituent effects can significantly impact diazirine reactivity. Considering this, future studies investigating substituent effects on competing inter- *versus* intra-molecular reactivity rates are warranted.

Photoaffinity tags may also have preferences to certain cellular features. For example, we observed that the Ar–C probe labeled membrane proteins to a larger extent compared to the alkyl diazirine control (Fig. 2b and S19a†). However, when analyzed in fractionated lysates, we see a substantial reduction in labelled membrane proteins (Fig. S5†). Such preferences may be the result of physicochemical features of the tag itself (*e.g.* lipophilicity) or due to potential reactivity preferences of photo-induced intermediates (*e.g.* diazo).^24–26^ We note that, overall, all tags themselves predominantly label and enrich non-membrane proteins (75–85% of total proteins, Fig. S19a†), similar to the distribution of the non-membrane proteins proteome wide and to what we have previously observed.^17^ However, we did observe that the membrane *vs.* non-membrane distribution of tag-enriched proteins appear impacted by appended molecular recognition groups (Fig. S19b–d†). For example, we found that relative to the control probes, there is a substantial increase in the fraction of membrane proteins enriched by the fragment (‘F’) and JQ series. Such observations are in-line with a recent study investigating the impact of different molecular recognition groups on diazirine profiles.^25^ Together, this suggests that labelling profiles of FF tags may not be completely driven by inherent diazirine reactivity preferences but are also significantly influenced by attached molecular recognition groups, which may alter the cellular partitioning of the probes in concordance with their physicochemical properties.

The ability of photoaffinity probes to irreversibly modify the target proteins enables the possibility to identify the binding site of small molecules, a significant advantage over label-free target identification techniques.^11^ Depending on the goals of a chemical proteomics experiment, the suitability of the probe for identifying sites of labeling may be a major factor in the choice of photoaffinity tag. Using the benzhydrylazetidine-based probe series as a test, we found probes LD–F and BD–F to be the most effective, allowing us to identify modified peptides with a high degree of confidence for 11.6% and 11.3% of enriched proteins, respectively. Binding sites could be localized to peptides for only 3.7% of the proteins enriched by the Tm-tagged fragment, which in other respects we found to behave similarly to the LD and BD tags. Although no Ar tag labelled peptides were identified, a naïve search for probe adducts, characteristic fragments of the probe could be observed in MS^2^ spectra (Fig. S14†) indicating that the tag is unstable during peptide fragmentation. While probe fragmentation may complicate analysis, future strategies to identify high-confidence characteristic fragments may aid binding site mapping studies. No probe-modified peptides or characteristic fragmentation could be identified using the DF-F probe, potentially due to lower overall protein capture.

The overlap of enriched proteins is strongly affected by the choice of photoaffinity tag. For example, we found that proteins enriched by LD-, BD-, and Tm-containing photoaffinity probes typically are common to all three tags, however, a significant proportion of enriched proteins are also unique to each photoaffinity tag (Fig. S20†). On the other hand, proteins enriched by the Ar or DF-based probes are often more non-overlapping. Along these lines, we failed to enrich many well-established targets of staurosporine, for example PRKCH,^38^ with any of the tested probes, presumably due to unfavorable orientations of the diazirine which may occlude crosslinking or reduced affinities towards other kinase targets. In this regard, effects of photoaffinity tag conjugation can frequently perturb biological activities of bioactive compounds;^11^ while conjugation of staurosporine to photoaffinity tags generally increased IC_50_ values by two orders of magnitude, the Ar tag resulted in almost complete loss of kinase inhibition and so the biological relevance of any proteins enriched by this probe would be questionable. Together, these observations suggest that proteomic profiles of diazirine-containing probes are not only affected by differences in diazirine chemical reactivity but are also substantially influenced by tag structure.

In conclusion, we have developed a new, structurally minimized photoaffinity tag which is comparable in protein enrichment to the alkyl diazirine-based photoaffinity tags in common use with relatively fewer background interactions. In our study of staurosporine-based photoaffinity probes, the Tm probe proved most effective (greatest ratio of enriched true targets to total enriched proteins) and provided similar overall coverage to other dialkyl diazirine tags (Fig. 3c and 4a). We have, however, found that this tag may not be as effective for mapping photo-adducted peptides. The shorter wavelength required to activate the Tm and DF tags may be advantageous in certain circumstances; if the target recognition group is itself a chromophore, the use of an alternative wavelength could help minimize irradiation times. However, shorter wavelengths may also have undesirable effects, such as protein damage and cell death.^40,41^ Nonetheless, we envision that terminal diazirines can be incorporated in other photo-affinity reagents and their reactivity can likely be tuned with various electronic neighboring groups. In our comparison of diazirine-based probes, we also found that the high, non-specific background labeling of the Ar tag limits its overall utility.

More generally, given the observed influential roles that tag choice plays on proteomic profiles, we believe that the studies herein provide a useful template for the characterization of new photo-affinity tags and the evaluation of diazirine-based probes. Finally, we conclude that no single tag is likely ideal for chemical proteomic investigations, and that maximizing the probability of target protein detection may require the use of multiple photoaffinity tags.

## Author contributions

C. G. P conceived of the research. C. G. P. and L. P. C. wrote the manuscript. L. P. C., A. M. J., R. A. H., and R. M. L. designed and performed experiments, analyzed data, and contributed to editing. W. L. assisted chemical proteomic experiments. J. S. and R. H. assisted with chemical synthesis and biological assays, respectively.

## Conflicts of interest

C. G. P. is a co-founder and scientific advisor to Belharra Therapeutics.

## Supplementary Material

SC-012-D1SC01360B-s001

SC-012-D1SC01360B-s002

SC-012-D1SC01360B-s003

SC-012-D1SC01360B-s004

## References

[cit1] Parker C. G., Pratt M. R. (2020). Cell.

[cit2] Herner A., Marjanovic J., Lewandowski T. M., Marin V., Patterson M., Miesbauer L., Ready D., Williams J., Vasudevan A., Lin Q. (2016). J. Am. Chem. Soc..

[cit3] Zhao S., Dai J., Hu M., Liu C., Meng R., Liu X., Wang C., Luo T. (2016). Chem. Commun..

[cit4] Bach K., Beerkens B. L. H., Zanon P. R. A., Hacker S. M. (2020). ACS Cent. Sci..

[cit5] Rostovtsev V. V., Green L. G., Fokin V. V., Sharpless K. B. (2002). Angew. Chem., Int. Ed. Engl..

[cit6] Hill J. R., Robertson A. A. B. (2018). J. Med. Chem..

[cit7] Dubinsky L., Krom B. P., Meijler M. M. (2012). Bioorg. Med. Chem..

[cit8] Keohane C. E., Steele A. D., Fetzer C., Khowsathit J., Van Tyne D., Maynie L., Gilmore M. S., Karanicolas J., Sieber S. A., Wuest W. M. (2018). J. Am. Chem. Soc..

[cit9] Gao J. X., Mfuh A., Amako Y., Woo C. M. (2018). J. Am. Chem. Soc..

[cit10] Parker C. G., Kuttruff C. A., Galmozzi A., Jorgensen L., Yeh C. H., Hermanson D. J., Wang Y. J., Artola M., McKerrall S. J., Josyln C. M., Norremark B., Dunstl G., Felding J., Saez E., Baran P. S., Cravatt B. F. (2017). ACS Cent. Sci..

[cit11] Conway L. P., Li W., Parker C. G. (2021). Cell Chem. Biol..

[cit12] Hong W. D., Benayoud F., Nixon G. L., Ford L., Johnston K. L., Clare R. H., Cassidy A., Cook D. A. N., Siu A., Shiotani M., Webborn P. J. H., Kavanagh S., Aljayyoussi G., Murphy E., Steven A., Archer J., Struever D., Frohberger S. J., Ehrens A., Hubner M. P., Hoerauf A., Roberts A. P., Hubbard A. T. M., Tate E. W., Serwa R. A., Leung S. C., Qie L., Berry N. G., Gusovsky F., Hemingway J., Turner J. D., Taylor M. J., Ward S. A., O'Neill P. M. (2019). Proc. Natl. Acad. Sci. U. S. A..

[cit13] Ross N. T., Lohmann F., Carbonneau S., Fazal A., Weihofen W. A., Gleim S., Salcius M., Sigoillot F., Henault M., Carl S. H., Rodriguez-Molina J. B., Miller H. R., Brittain S. M., Murphy J., Zambrowski M., Boynton G., Wang Y., Chen A., Molind G. J., Wilbertz J. H., Artus-Revel C. G., Jia M., Akinjiyan F. A., Turner J., Knehr J., Carbone W., Schuierer S., Reece-Hoyes J. S., Xie K. V., Saran C., Williams E. T., Roma G., Spencer M., Jenkins J., George E. L., Thomas J. R., Michaud G., Schirle M., Tallarico J., Passmore L. A., Chao J. R. A., Beckwith R. E. J. (2020). Nat. Chem. Biol..

[cit14] Stepek I. A., Cao T., Koetemann A., Shimura S., Wollscheid B., Bode J. W. (2019). ACS Chem. Biol..

[cit15] Beiroth F., Koudelka T., Overath T., Knight S. D., Tholey A., Lindhorst T. K. (2018). Beilstein J. Org. Chem..

[cit16] Wang Y. C., Westcott N. P., Griffin M. E., Hang H. C. (2019). ACS Chem. Biol..

[cit17] Parker C. G., Galmozzi A., Wang Y., Correia B. E., Sasaki K., Joslyn C. M., Kim A. S., Cavallaro C. L., Lawrence R. M., Johnson S. R., Narvaiza I., Saez E., Cravatt B. F. (2017). Cell.

[cit18] Wang Y., Dix M. M., Bianco G., Remsberg J. R., Lee H. Y., Kalocsay M., Gygi S. P., Forli S., Vite G., Lawrence R. M., Parker C. G., Cravatt B. F. (2019). Nat. Chem..

[cit19] Brodie N. I., Makepeace K. A. T., Petrotchenko E. V., Borchers C. H. (2015). J. Proteomics.

[cit20] Yang T. P., Liu Z., Li X. D. (2015). Chem. Sci..

[cit21] Kleiner P., Heydenreuter W., Stahl M., Korotkov V. S., Sieber S. A. (2017). Angew. Chem., Int. Ed..

[cit22] Park J., Koh M., Koo J. Y., Lee S., Park S. B. (2016). ACS Chem. Biol..

[cit23] Pan S. J., Jang S. Y., Wang D. Y., Liew S. S., Li Z. Q., Lee J. S., Yao S. Q. (2017). Angew. Chem., Int. Ed..

[cit24] O'Brien J. G. K., Jemas A., Asare-Okai P. N., Am Ende C. W., Fox J. M. (2020). Org. Lett..

[cit25] West A. V., Muncipinto G., Wu H. Y., Huang A. C., Labenski M. T., Jones L. H., Woo C. M. (2021). J. Am. Chem. Soc..

[cit26] Iacobucci C., Gotze M., Piotrowski C., Arlt C., Rehkamp A., Ihling C., Hage C., Sinz A. (2018). Anal. Chem..

[cit27] Ziemianowicz D. S., Bomgarden R., Etienne C., Schriemer D. C. (2017). J. Am. Soc. Mass Spectrom..

[cit28] Li Z. Q., Hao P. L., Li L., Tan C. Y. J., Cheng X. M., Chen G. Y. J., Sze S. K., Shen H. M., Yao S. Q. (2013). Angew. Chem., Int. Ed..

[cit29] Kambe T., Correia B. E., Niphakis M. J., Cravatt B. F. (2014). J. Am. Chem. Soc..

[cit30] Chambers S. A., Townsend S. D. (2020). Carbohydr. Res..

[cit31] Chang C. F., Mfuh A., Gao J. X., Wu H. Y., Woo C. M. (2018). Tetrahedron.

[cit32] Glachet T., Marzag H., Saraiva Rosa N., Colell J. F. P., Zhang G., Warren W. S., Franck X., Theis T., Reboul V. (2019). J. Am. Chem. Soc..

[cit33] Brunner J., Senn H., Richards F. M. (1980). J. Biol. Chem..

[cit34] Thompson A., Schafer J., Kuhn K., Kienle S., Schwarz J., Schmidt G., Neumann T., Hamon C. (2003). Anal. Chem..

[cit35] Qin K., Zhu Y., Qin W., Gao J., Shao X., Wang Y. L., Zhou W., Wang C., Chen X. (2018). ACS Chem. Biol..

[cit36] Anders L., Guenther M. G., Qi J., Fan Z. P., Marineau J. J., Rahl P. B., Loven J., Sigova A. A., Smith W. B., Lee T. I., Bradner J. E., Young R. A. (2014). Nat. Biotechnol..

[cit37] Shi H. B., Cheng X. M., Sze S. K., Yao S. Q. (2011). Chem. Commun..

[cit38] Karaman M. W., Herrgard S., Treiber D. K., Gallant P., Atteridge C. E., Campbell B. T., Chan K. W., Ciceri P., Davis M. I., Edeen P. T., Faraoni R., Floyd M., Hunt J. P., Lockhart D. J., Milanov Z. V., Morrison M. J., Pallares G., Patel H. K., Pritchard S., Wodicka L. M., Zarrinkar P. P. (2008). Nat. Biotechnol..

[cit39] Das J. (2011). Chem. Rev..

[cit40] Kaya E., Vrabel M., Deiml C., Prill S., Fluxa V. S., Carell T. (2012). Angew. Chem., Int. Ed..

[cit41] Wells R. L., Han A. (1985). Int. J. Radiat. Biol..

