# Supplementary material for: Evaluation of fully-functionalized diazirine tags for chemical proteomic applications
Source: Chem Sci. 2021 May 7;12(22):7839–47. doi: 10.1039/d1sc01360b (PMC8188597; doi:10.1039/d1sc01360b)
Supplement: SC-012-D1SC01360B-s002 [file SC-012-D1SC01360B-s002.pdf]

Electronic Supplementary Material (ESI) for Chemical Science.
This journal is © The Royal Society of Chemistry 2021

Supplementary Materials

Evaluation of fully functionalized diazirine tags for chemical proteomic applications

Louis P. Conway,[a†] Appaso M. Jadhav,[a†] Rick A. Homan,[a†] Weichao Li,[a] Juanita Sanchez Rubiano,[a] Richard Hawkins,[a] R. Michael Lawrence,[b] and Christopher G. Parker*[a]

[a] Department of Chemistry
The Scripps Research Institute, Jupiter FL, USA
[b] Research and Development, Bristol Myers Squibb Company, Princeton NJ, USA
† These authors contributed equally

* Corresponding author: C.G.P. Email: cparker@scripps.edu

Contents

Supplementary Figures....6
Figure S1....6
Figure S2....7
Figure S3....8
Figure S4....9
Figure S5....10
Figure S6....11
Figure S7....12
Figure S8....13
Figure S9....14
Figure S10....15
Figure S11....16
Figure S12....17
Figure S13....18
Figure S14....19
Figure S15....20

Figure S16 21
Figure S17 22
Figure S18 23
Figure S19 24
Figure S20 25
Biological Methods 26
a) Decomposition Kinetics Studies 26
b) Labeling of Cell Lysates with Photoaffinity Probes 26
c) Labeling of Live Cells with Photoaffinity Probes 27
d) Gel-based Analysis of Crosslinked Proteins 27
e) Fragment Probe Cell Treatments 27
f) Protein Kinase A Staurosporine Probe Activity Assay 28
g) Antiproliferation Assay of JQ1 Probe Bromodomain Inhibitors 28
h) JQ-1 Probe Cell Treatments 29
i) Staurosporine Probe Lysate Treatments 29
j) Preparation of Samples for TMT Mass Spectrometric Proteomics 29
k) LC-MS Analysis of TMT samples 31
l) Preparation of Samples for Site of Labeling Experiment 32
m) LC-MS Analysis of Site of Labeling Samples 33
n) Site of Labeling Data Analysis 34
Synthetic Methods 36
a) Synthesis of terminal diazirine alkyne tag *N*-((3H-diazirin-3-yl)methyl)prop-2-yn-1-amine (Tm) 36
b) Synthesis of N-(2-(3-methyl-3H-diazirin-3-yl)ethyl)prop-2-yn-1-amine: 38
c) Synthesis of (3-(1,1-difluoro-3-(triisopropylsilyl)prop-2-yn-1-yl)-3H-diazirin-3-yl)methanol: Prepared according to literature procedures.[4] Analytical data are identical to previously reported data. 38
d) General procedures for the synthesis of diazirine containing tags: 38

e) Procedure for the synthesis of staurosporine amine linker: 39

f) Synthetic procedures of JQ1 amine linker: 41

g) Procedures for the synthesis of JQ1 acid linker: 42

h) Isotopically labeled dialkoxydiphenylsilane (DADPS) tag : 43

i) General procedure for synthesis of the cleavable biotin azide light valine and heavy valine tag 43

Characterization data for diazirine probes 48

N-(2-(3-(But-3-yn-1-yl)-3H-diazirin-3-yl)ethyl)benzamide (LD-C) 48

N-Phenyl-2-(prop-2-yn-1-yloxy)-4-(3-(trifluoromethyl)-3H-diazirin-3-yl)benzamide (Ar-C) 48

N-(2-(3-Methyl-3H-diazirin-3-yl)ethyl)-N-(prop-2-yn-1-yl)benzamide (BD-C) 49

(3-(1,1-Difluoroprop-2-yn-1-yl)-3H-diazirin-3-yl)methyl phenylcarbamate (DF-C).. 49

N-((3H-Diazirin-3-yl)methyl)-N-(prop-2-yn-1-yl)benzamide (Tm-C) 50

1-Benzhydryl-N-(2-(3-(but-3-yn-1-yl)-3H-diazirin-3-yl)ethyl)azetidine-3-carboxamide (LD-F) 50

N-(1-Benzhydrylazetidin-3-yl)-2-(prop-2-yn-1-yloxy)-4-(3-(trifluoromethyl)-3H-diazirin-3-yl)benzamide (Ar-F) 50

1-Benzhydryl-N-(2-(3-methyl-3H-diazirin-3-yl)ethyl)-N-(prop-2-yn-1-yl)azetidine-3-carboxamide (BD-F) 51

(3-(1,1-Difluoroprop-2-yn-1-yl)-3H-diazirin-3-yl)methyl(1-benzhydrylazetidin-3-yl)carbamate (DF-F) 52

N-((3H-Diazirin-3-yl)methyl)-1-benzhydryl-*N*-(prop-2-yn-1-yl)azetidine-3-carboxamide (Tm-F) 52

N^1-(2-(3-(But-3-yn-1-yl)-3H-diazirin-3-yl)ethyl)-N^4-((5R,7R,8R,9S)-8-methoxy-9-methyl-16-oxo-6,7,8,9,15,16-hexahydro-5H,14H-17-oxa-4b,9a,15-triaza-5,9 methanodibenzo[b,h]cyclonona[jkl]cyclopenta[e]-as-indacen-7-yl)-N^4-methylsuccinamide (LD-St) 53

N-(3-(((5R,7R,8R,9S)-8-Methoxy-9-methyl-16-oxo-6,7,8,9,15,16-hexahydro-5H,14H-17-oxa-4b,9a,15-triaza-5,9-

methanodibenzo[b,h]cyclonona[jkl]cyclopenta[e]-as-indacen-7-yl)(methyl)amino)-3-oxopropyl)-2-(prop-2-yn-1-yloxy)-4-(3-(trifluoromethyl)-3H-diazirin-3-yl)benzamide (Ar-St) 54

N^1-((5R,7R,8R,9S)-8-Methoxy-9-methyl-16-oxo-6,7,8,9,15,16-hexahydro-5H,14H-17-oxa-4b,9a,15-triaza-5,9-methanodibenzo[b,h]cyclonona[jkl]cyclopenta[e]-as-indacen-7-yl)-N^1-methyl-N^4-(2-(3-methyl-3H-diazirin-3-yl)ethyl)-N^4-(prop-2-yn-1-yl)succinimide (BD-St) 54

(3-(1,1-Difluoroprop-2-yn-1-yl)-3H-diazirin-3-yl)methyl (3-(((5R,7R,8R,9S)-8-methoxy-9-methyl-16-oxo-6,7,8,9,15,16-hexahydro-5H,14H-17-oxa-4b,9a,15-triaza-5,9-methanodibenzo[b,h]cyclonona[jkl]cyclopenta[e]-as-indacen-7-yl)(methyl)amino)-3-oxopropyl)carbamate (DF-St) 55

N^1-((3H-Diazirin-3-yl)methyl)-N^4-((5R,7R,8R,9S)-8-methoxy-9-methyl-16-oxo-6,7,8,9,15,16-hexahydro-5H,14H-17-oxa-4b,9a,15-triaza-5,9-methanodibenzo[b,h]cyclonona[jkl]cyclopenta[e]-as-indacen-7-yl)-N^4-methyl-N^1-(prop-2-yn-1-yl)succinimide (Tm-St) 56

(S)-N-(2-(3-(But-3-yn-1-yl)-3H-diazirin-3-yl)ethyl)-3-(2-(4-(4-chlorophenyl)-2,3,9-trimethyl-6H-thieno[3,2-f][1,2,4]triazolo[4,3-a][1,4]diazepin-6-yl)acetamido)propenamide (LD-JQ) 57

(S)-*N*-(2-(2-(4-(4-Chlorophenyl)-2,3,9-trimethyl-6H-thieno[3,2-f][1,2,4]triazolo[4,3-a][1,4]diazepin-6-yl)acetamido)ethyl)-2-(prop-2-yn-1-yloxy)-4-(3-(trifluoromethyl)-3H-diazirin-3-yl)benzamide 58

(S)-3-(2-(4-(4-Chlorophenyl)-2,3,9-trimethyl-6H-thieno[3,2-f][1,2,4]triazolo[4,3-a][1,4]diazepin-6-yl)acetamido)-N-(2-(3-methyl-3H-diazirin-3-yl)ethyl)-N-(prop-2-yn-1-yl)propenamide (BD-JQ) 59

(3-(1,1-Difluoroprop-2-yn-1-yl)-3H-diazirin-3-yl)methyl (S)-(2-(2-(4-(4-chlorophenyl)-2,3,9-trimethyl-6H-thieno[3,2-f][1,2,4]triazolo[4,3-a][1,4]diazepin-6-yl)acetamido)ethyl)carbamate (DF-JQ) 60

(S)-N-((3H-Diazirin-3-yl)methyl)-3-(2-(4-(4-chlorophenyl)-2,3,9-trimethyl-6H-thieno[3,2-f][1,2,4]triazolo[4,3-a][1,4]diazepin-6-yl)acetamido)-N-(prop-2-yn-1-yl)propenamide (Tm-JQ) 60

References 62

NMR Spectra 63

Supplementary Figures

Figure S1

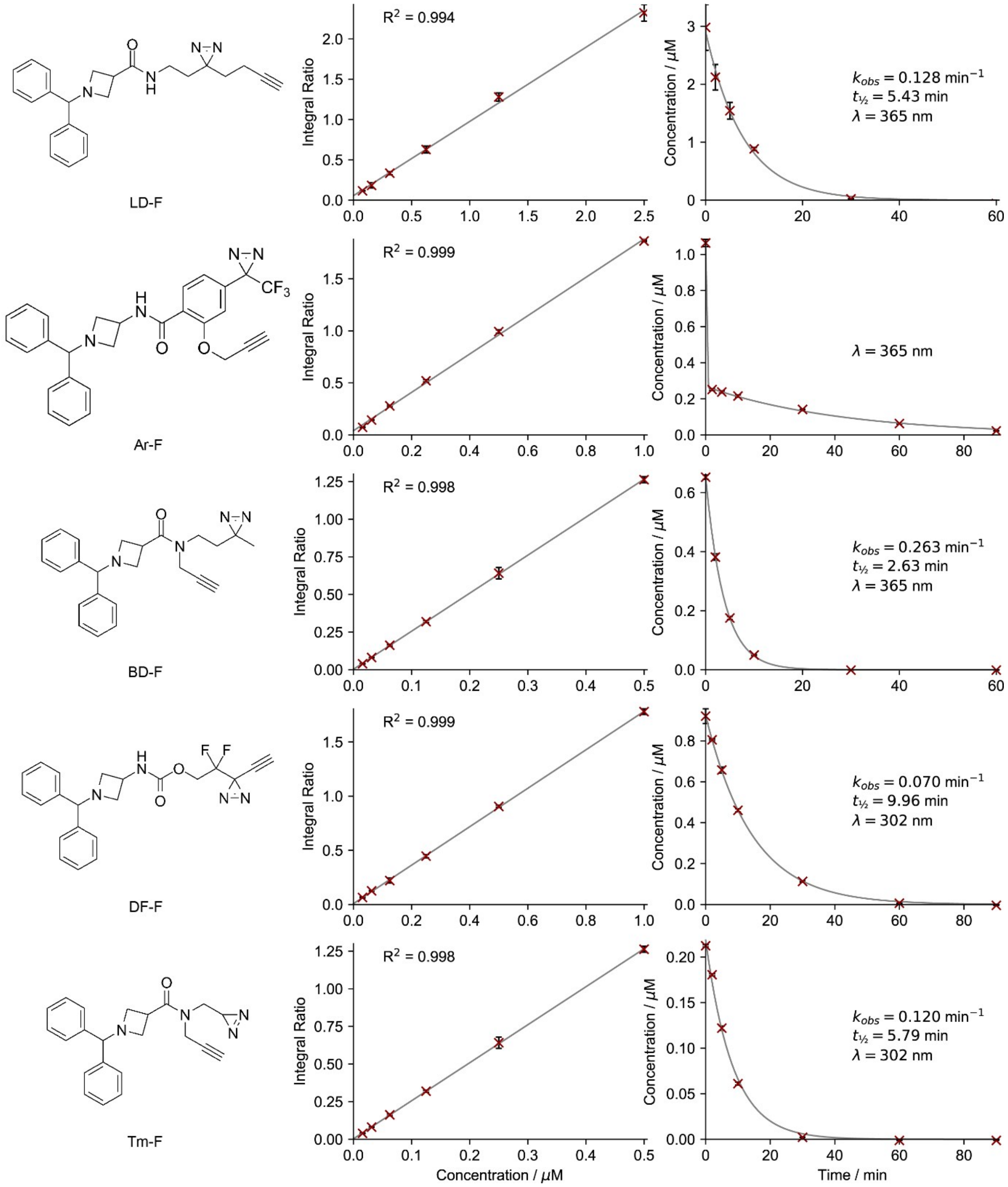

Figure S1. Calibration curves and plots of the time-dependent decomposition of solutions of ‘F’ fragment-based probes in isopropanol. Integral ratios are relative to a *p*-nitroaniline standard, error bars represent standard deviations from three replicates.

Figure S2

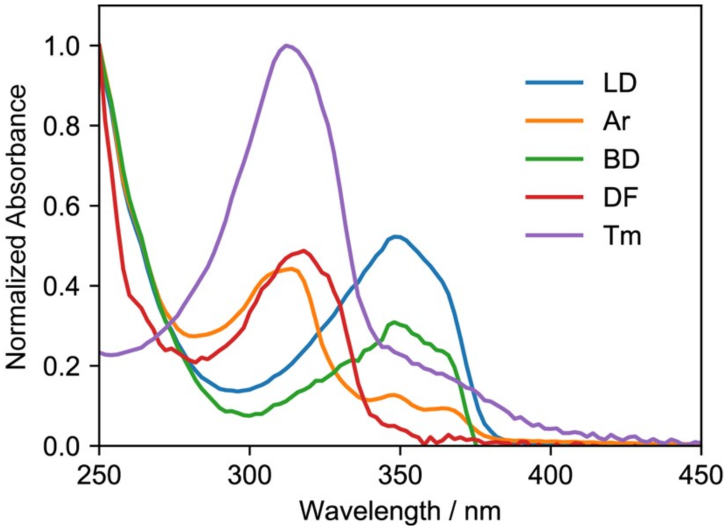

Figure S2. Normalized UV-Vis absorbance spectrum for the LD, Ar, BD, DF, and Tm tags. The DF and Tm maxima at *circa* 310 nm suggested that UV irradiation at a shorter wavelength might more effectively activate these tags.

Figure S3

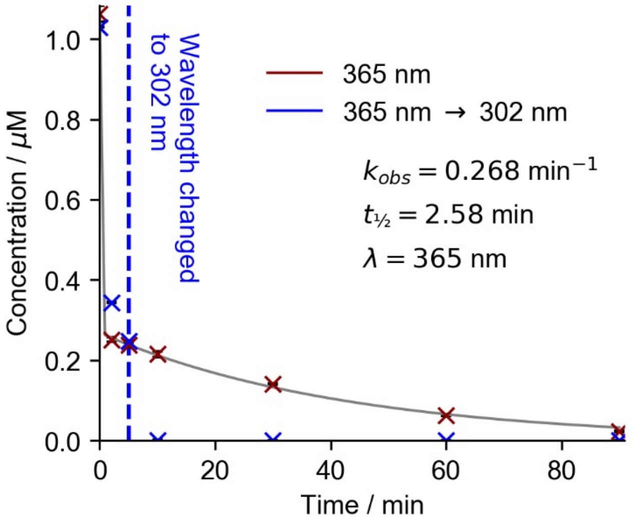

Figure S3. Probe Ar-F was irradiated at 365 nm for 5 min, followed by irradiation at 302 nm. Error bars represent standard deviations from three replicates.

Figure S4

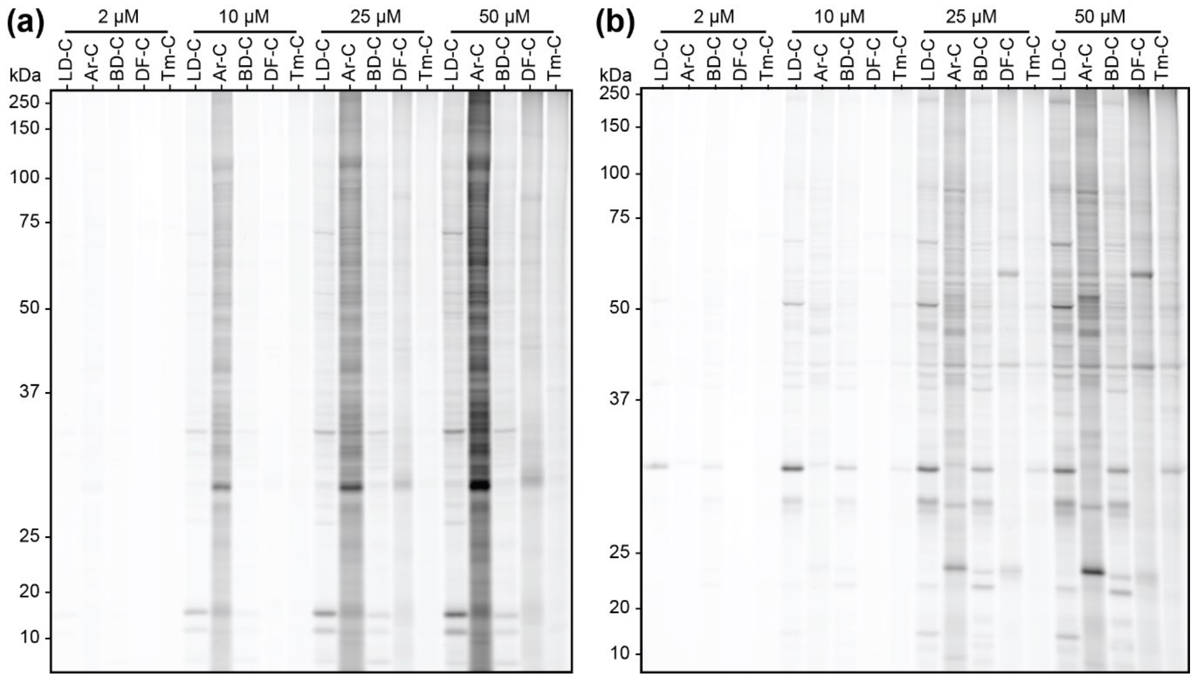

Figure S4. HEK293T cells were treated with 'C' control probes at a range of concentrations, irradiated (365 nm UV for probes LD-C, Ar-C, and BD-C, 302 nm UV for probes DF-C and Tm-C) for 15 min, and analyzed by in-gel fluorescence. a) Particulate fraction. b) Soluble fraction

Figure S5

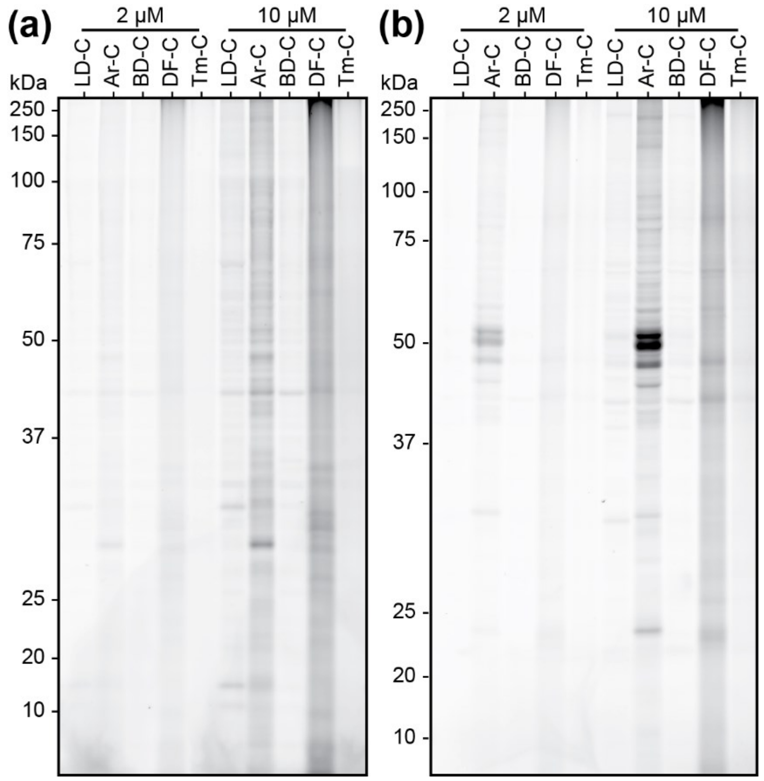

Figure S5. HEK293T cell lysates were treated with 'C' control probes at concentrations of 2 µM and 10 µM, irradiated (365 nm UV for probes LD-C, Ar-C, and BD-C, 302 nm UV for probes DF-C and Tm-C) for 15 min, and analyzed by in-gel fluorescence. a) Particulate fraction. b) Soluble fraction.

Figure S6

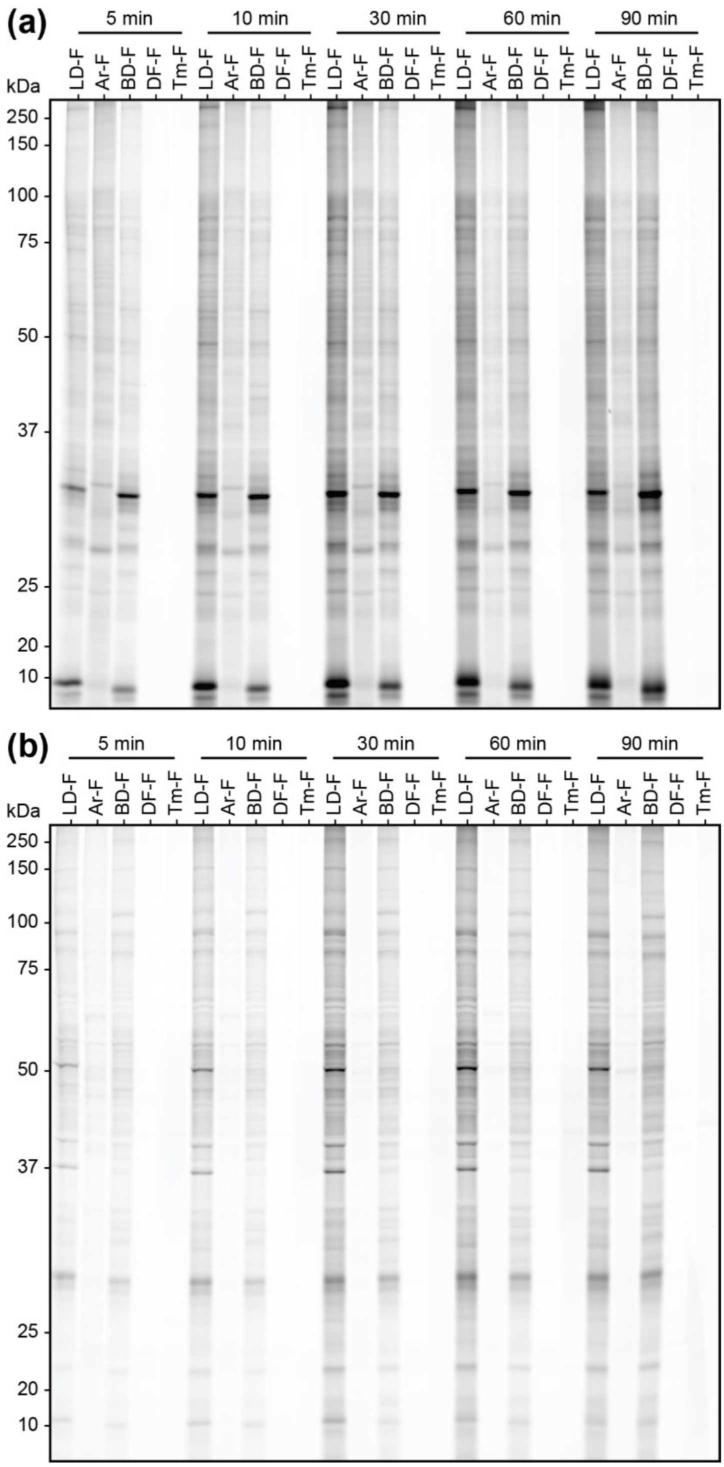

Figure S6. HEK293T cells were treated with 'F' fragment probes (20 μM), irradiated with 365 nm UV light for a range of time periods, and analyzed by in-gel fluorescence. a) Particulate fraction. b) Soluble fraction.

Figure S7

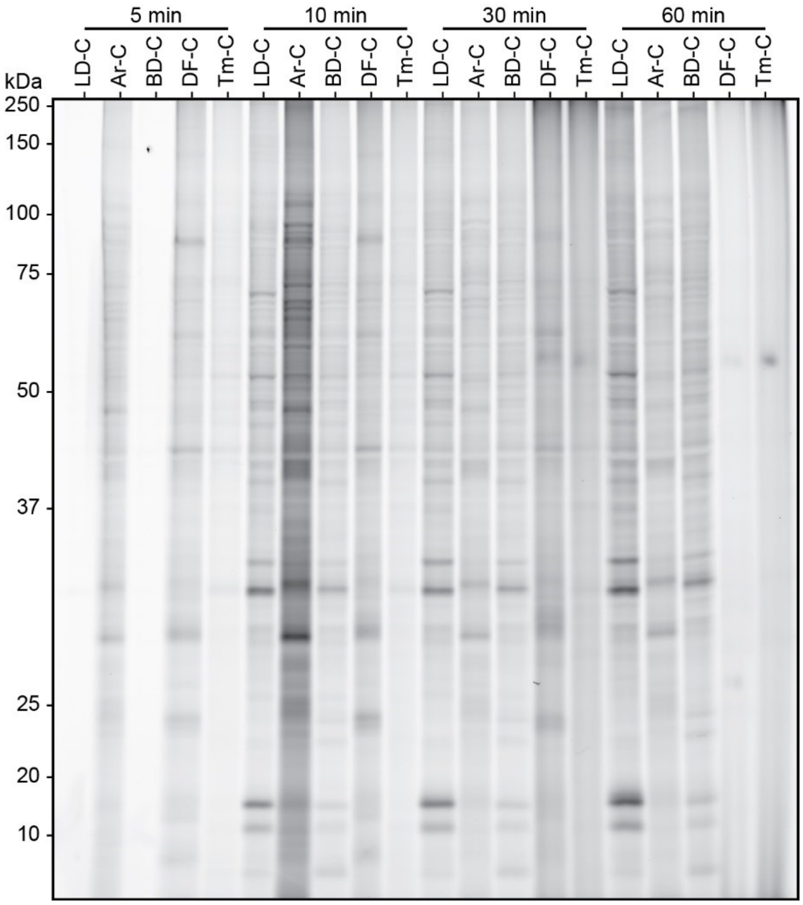

Figure S7. HEK293T cell lysates were treated with ‘C’ control probes (20 μM), irradiated with UV light (365 nm UV for probes LD-C, Ar-C, and BD-C, 302 nm UV for probes DF-C and Tm-C) for a range of time periods, and the unfractionated lysates were analyzed by in-gel fluorescence.

Figure S8

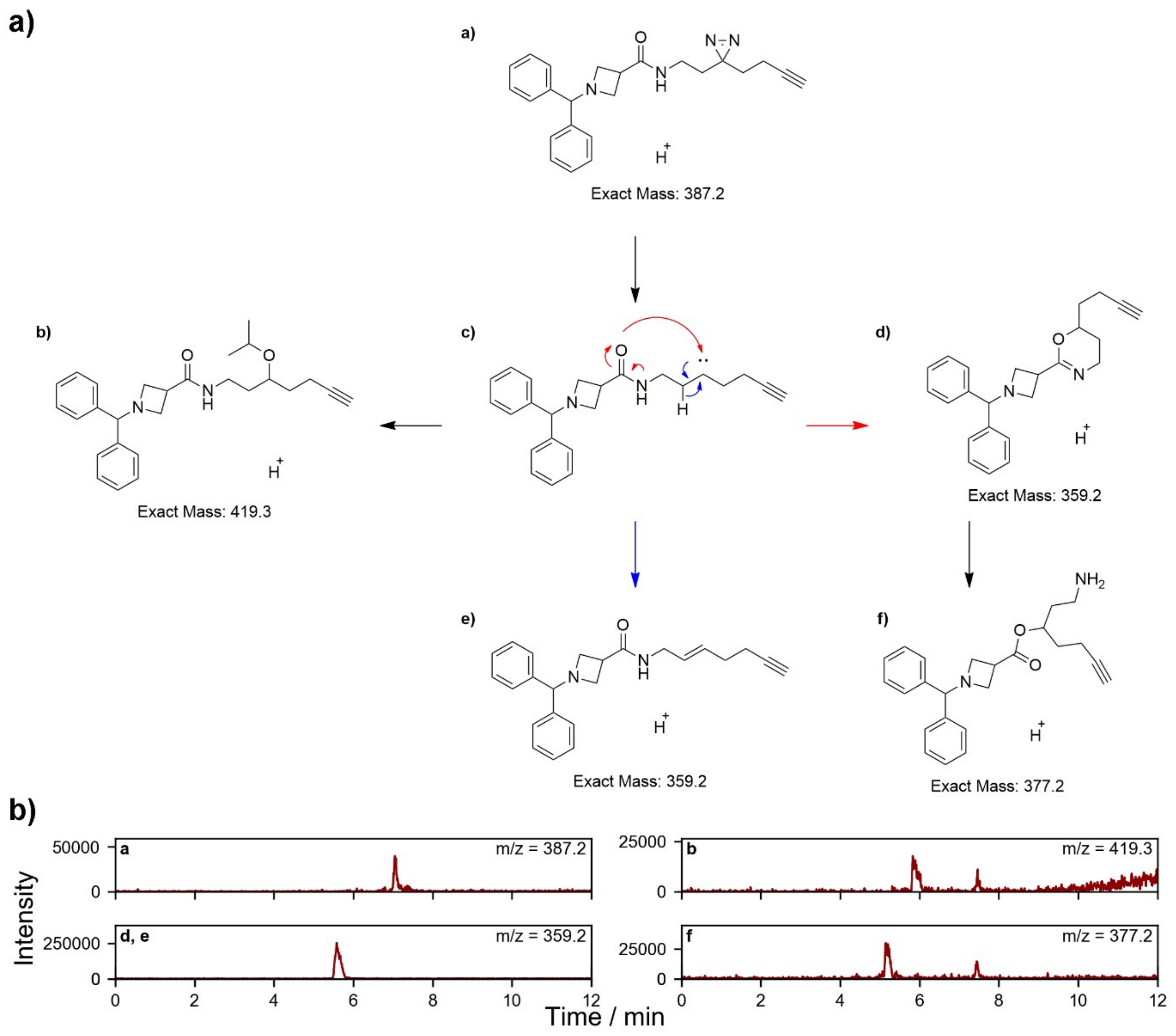

Figure S8. a) Postulated products of the LD-F probe upon UV irradiation. b) Extracted ion chromatograms for selected *m/z* values corresponding to potential side products, based on those postulated by O'Brien et al.[1]

Figure S9

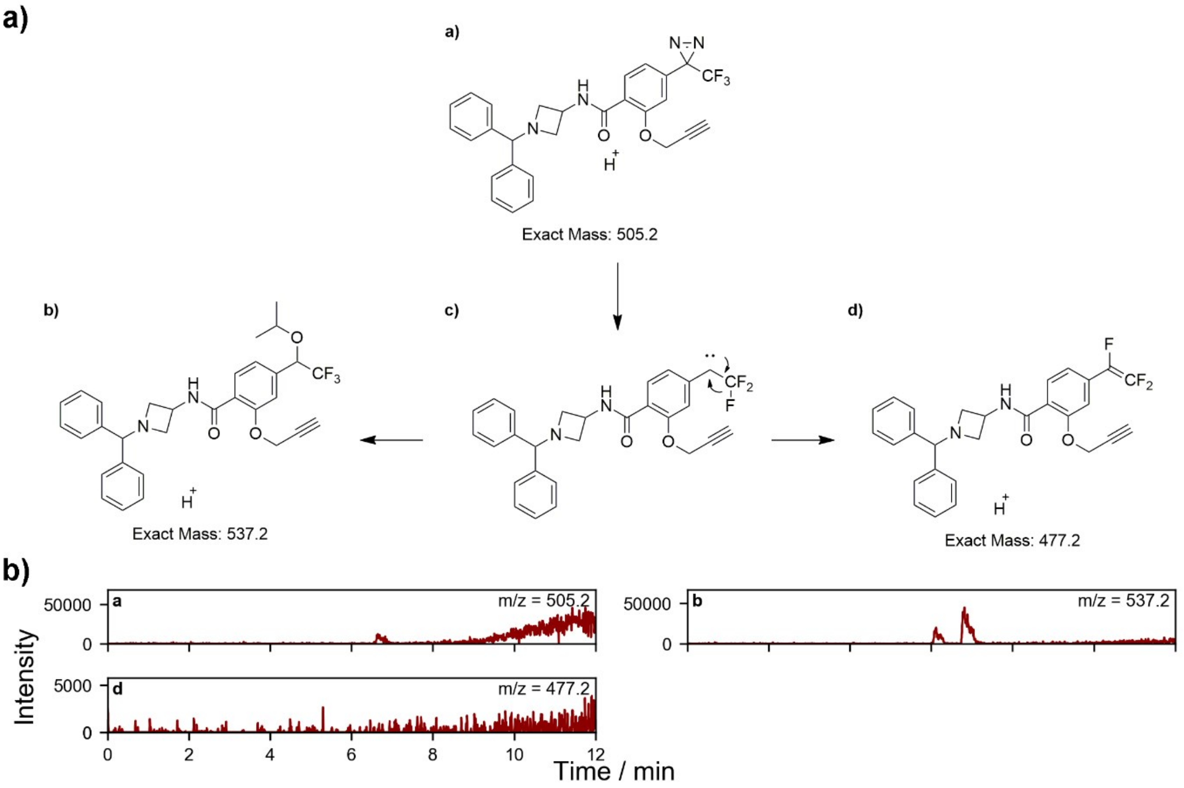

Figure S9. a) Postulated products of the Ar-F probe upon UV irradiation. b) Extracted ion chromatograms for selected *m/z* values corresponding to postulated side products.

Figure S10

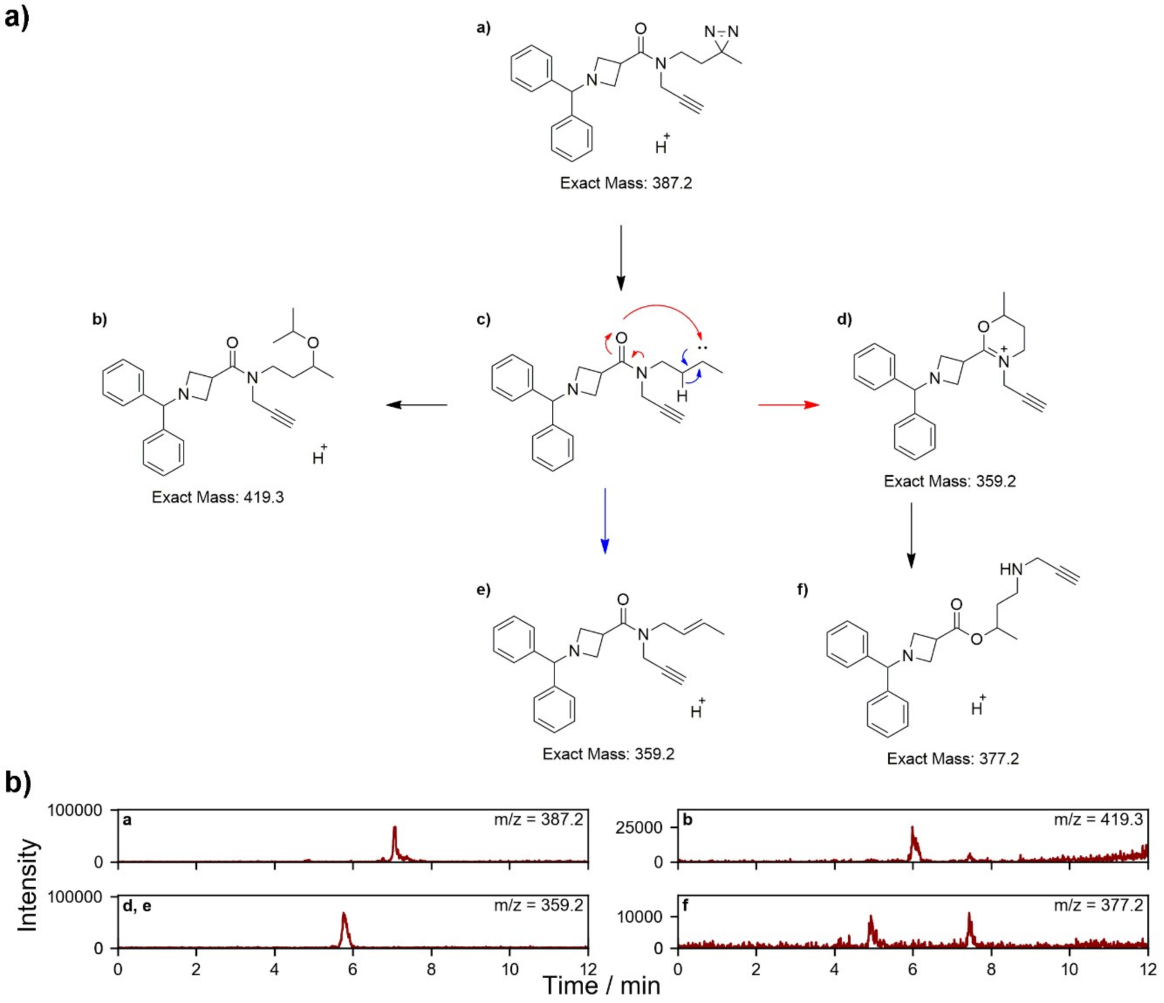

Figure S10.

a) Postulated products of the BD-F probe upon UV irradiation. b) Extracted ion chromatograms for selected *m/z* values corresponding to potential side products, based on those postulated by O'Brien et al.[1]

Figure S11

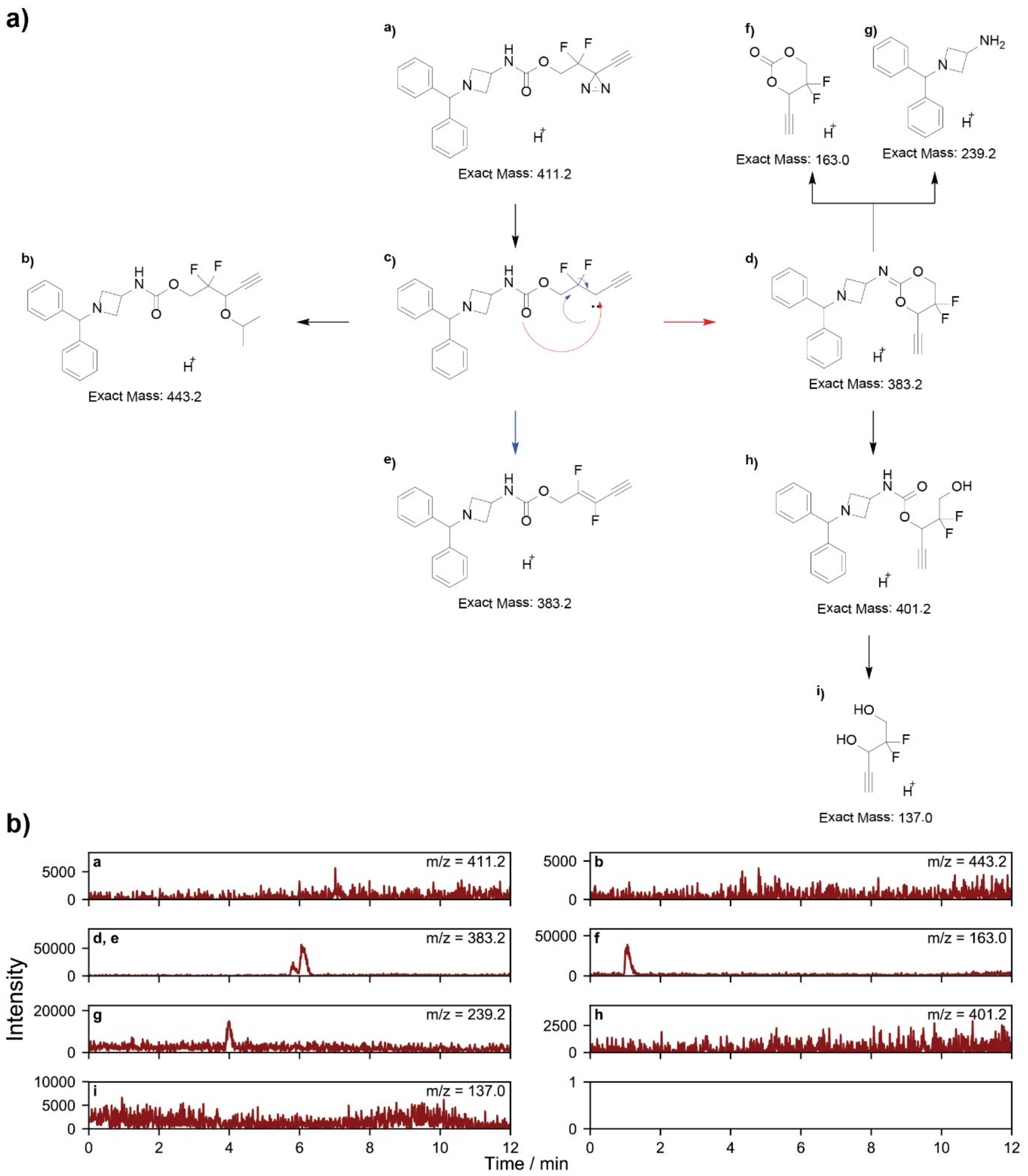

Figure S11. a) Postulated products of the DF-F probe upon UV irradiation. b) Extracted ion chromatograms for selected *m/z* values corresponding to postulated side products.

Figure S12

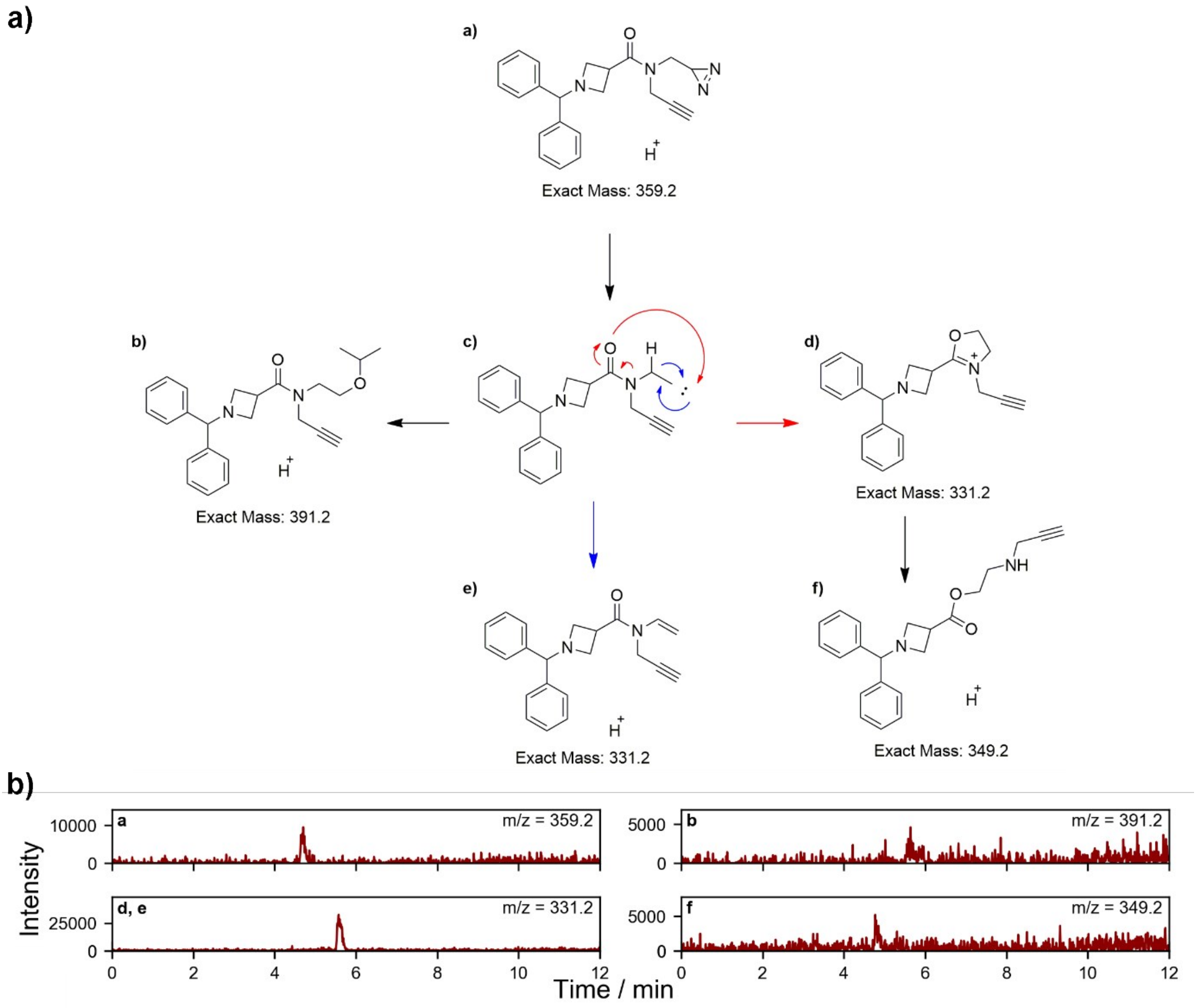

Figure S12. a) Postulated products of the Tm-F probe upon UV irradiation. b) Extracted ion chromatograms for selected *m/z* values corresponding to postulated side products.

Figure S13

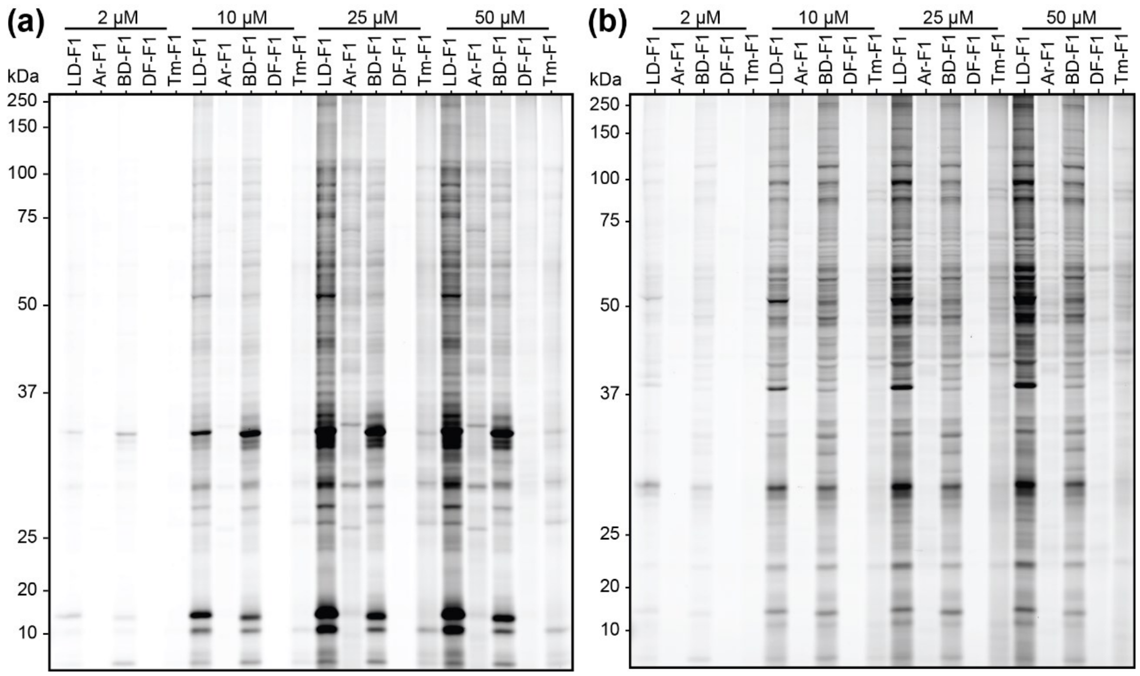

Figure S13. HEK293T cells were treated with ‘F’ fragment probes at a range of concentrations, irradiated with UV light (365 nm UV for probes LD-F, Ar-F, and BD-F, 302 nm UV for probes DF-F and Tm-F) for 15 min, and analyzed by in-gel fluorescence. a) Particulate fraction. b) Soluble fraction.

Figure S14

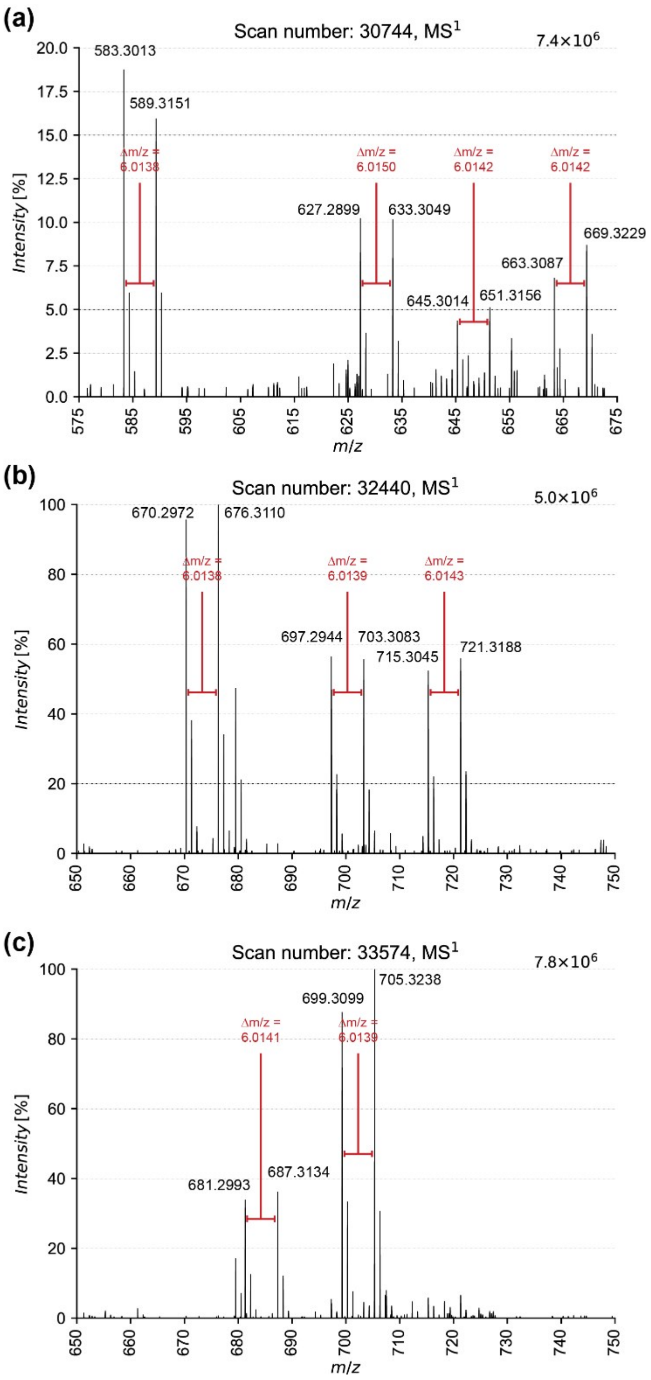

Figure S14. Representative MS¹ spectra illustrating the fragmentation of the Ar-F probe. Few peptides modified with the probe were detected in the proteomic analysis of cells treated with Ar-F, however, inspection of the mass spectra showed the presence of many ion pairs with mass difference close to 6.0138 m/z, corresponding to the difference between the 'heavy' and 'light' tags indicating the presence of fragmentation products of the probe-peptide adduct.

Figure S15

(a)

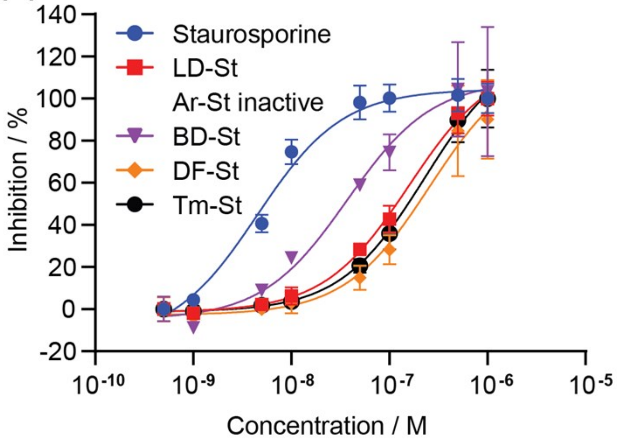

(b)

Compound	IC_{50} / nM	95 % CI
LD-St	158	122-208
Ar-St	ND	
BD-St	37	19-69
DF-St	259	144-524
Tm-St	231	163-338
Staurosporine	4.5	3.3-6.1

Figure S15. Probes incorporating staurosporine retain diminished inhibition of Protein Kinase A (PKA) catalytic subunit activity towards Kemptide, except for probe Ar-JQ which has negligible activity at the tested concentrations. A) Dose-response curves for cell viability in the presence of staurosporine-based probes and staurosporine itself. B) IC_{50} values and confidence intervals calculated from the dose-response curves.

Figure S16

(a)

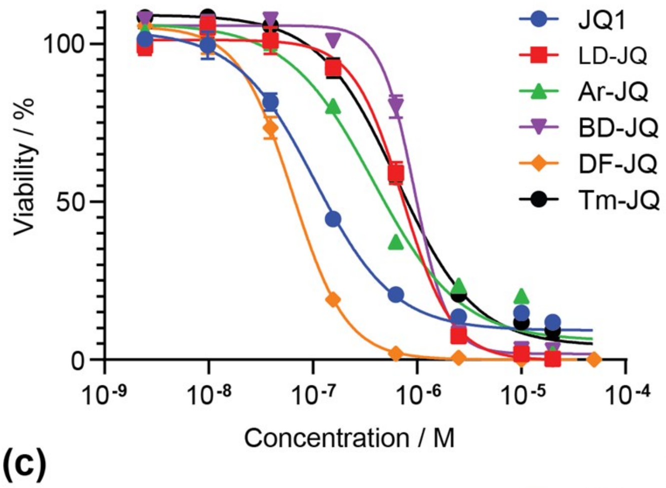

(b)

Compound	IC_{50} / nM	95 % CI
LD-JQ	731	670-799
Ar-JQ	383	287-534
BD-JQ	940	866-1015
DF-JQ	64	60-68
Tm-JQ	643	566-731
JQ1	104	84-128

(c)

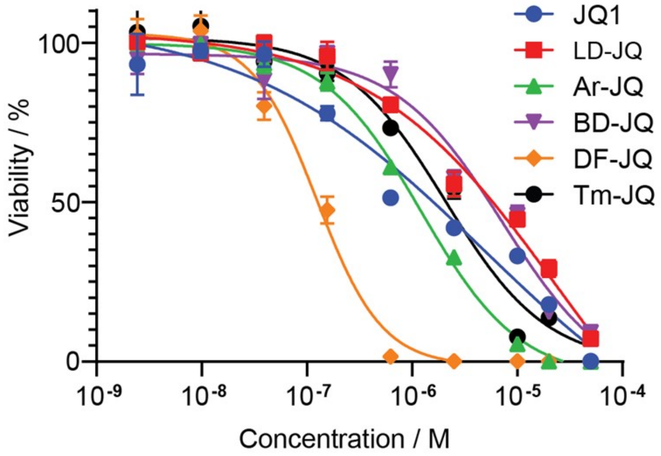

(d)

Compound	IC_{50} / µM	95 % CI
LD-JQ	24	>6
Ar-JQ	1.2	1.1-1.4
BD-JQ	7.2	3.7-33
DF-JQ	0.12	0.10-0.15
Tm-JQ	2.1	1.4-3.5
JQ1	4.8	>0.8

Figure S16. Incorporation of JQ1 into photoaffinity probes results in increased cell viability with the exception of probe DF-JQ. a) Dose-response curves for NCI-H1299 cell viability in the presence of JQ1-based probes and JQ1 itself (72 h). b) IC_{50} values and confidence intervals calculated from the NCI-H1299 dose-response curves. c) Dose-response curves for THP-1 cell viability in the presence of JQ1-based probes and JQ1 itself (48 h). b) IC_{50} values and confidence intervals calculated from the THP-1 dose-response curves.

Figure S17

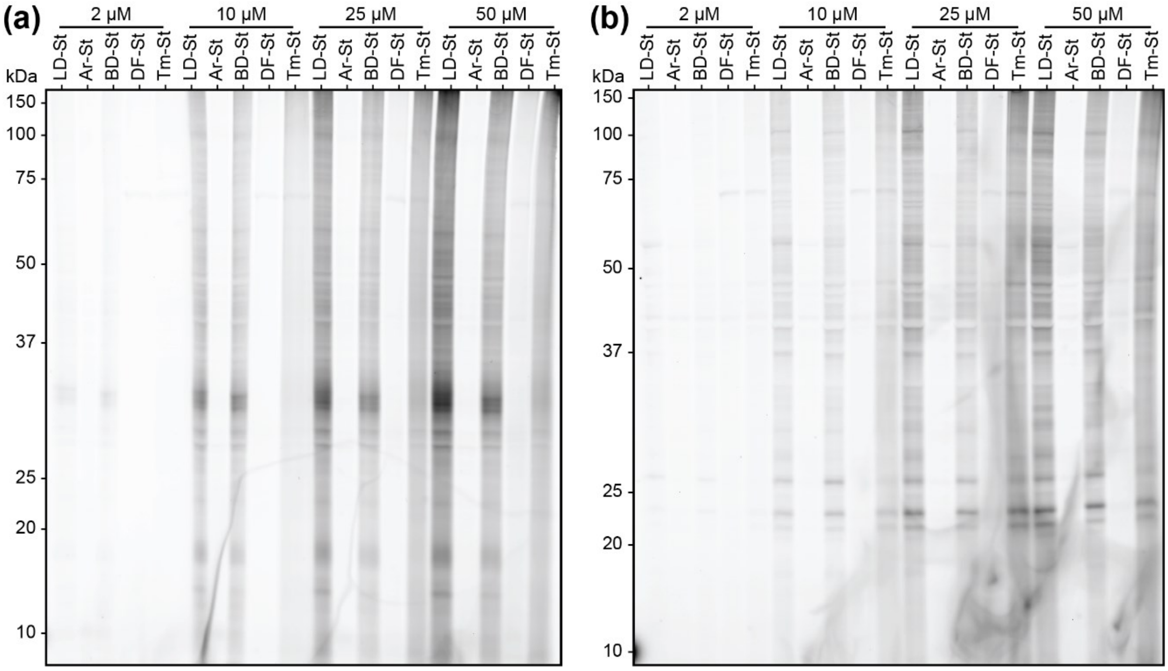

Figure S17. Fractionated K562 cell lysates were treated with staurosporine-based ‘St’ probes at a range of concentrations, irradiated with UV light (365 nm UV for probes LD-St, Ar-St, and BD-St, 302 nm UV for probes DF-St and Tm-St) for 15 min, and analyzed by in-gel fluorescence. a) Particulate fraction. b) Soluble fraction.

Figure S18

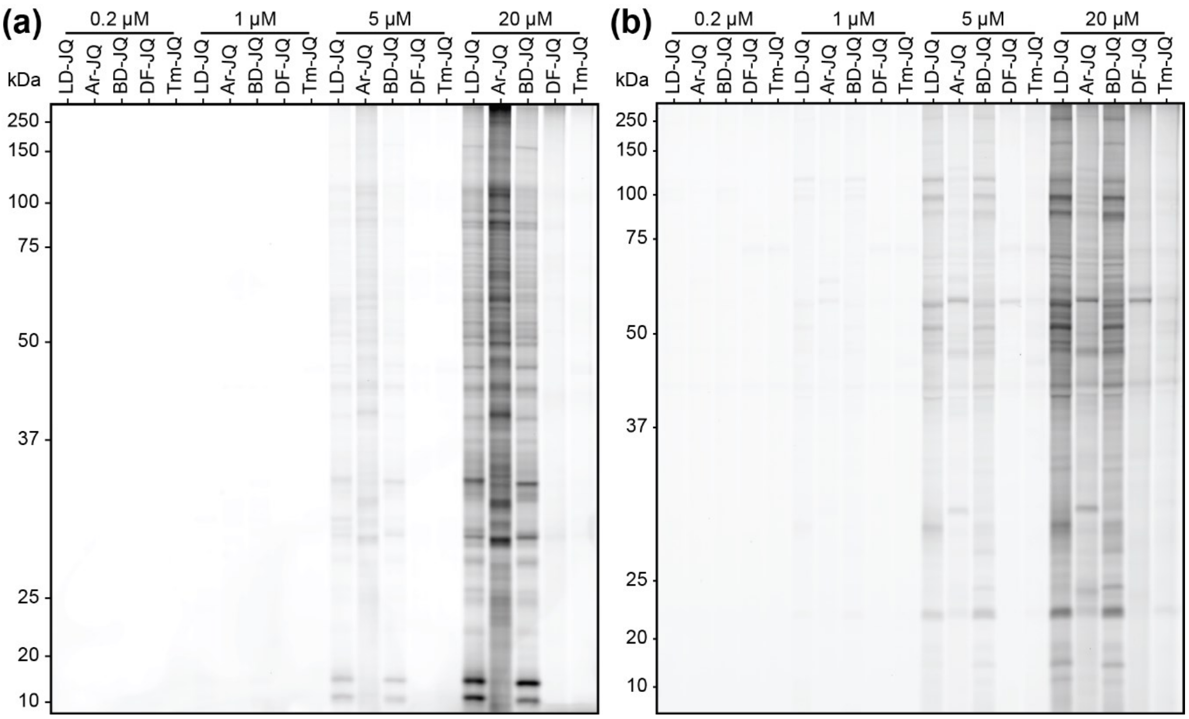

Figure S18. K562 cells were treated with JQ1-based 'JQ' probes at a range of concentrations, irradiated with UV light (365 nm UV for probes LD-JQ, Ar-JQ, and BD-JQ, 302 nm UV for probes DF-JQ and Tm-JQ) for 15 min, and analyzed by in-gel fluorescence. a) Particulate fraction. b) Soluble fraction.

Figure S19

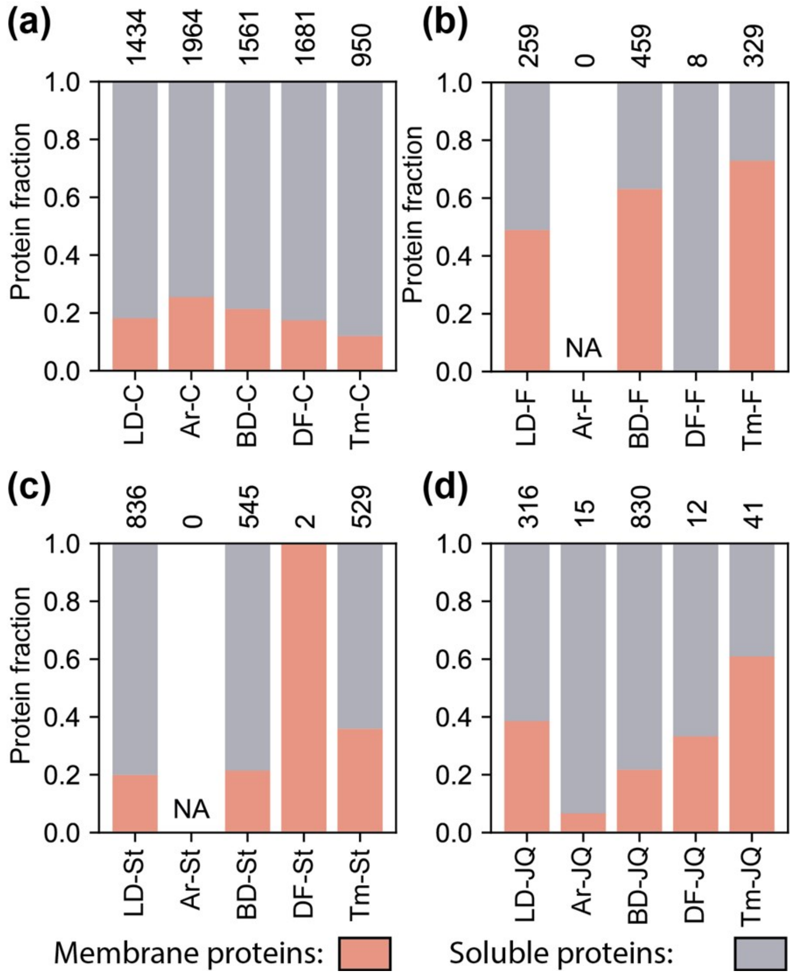

Figure S19. The proportion of significantly enriched membrane and non-membrane proteins (membrane proteins are defined as those which possess one or more trans-membrane domains as annotated by Uniprot) for each probe. a) ‘C’ control probes, b) ‘F’ fragment probes, c) ‘St’ staurosporine-based probes, and d) JQ1-based ‘JQ’ probes. Total ‘hits’ for each probe are shown at the top of each plot. Membrane proteins are proteins which have been annotated as having one or more transmembrane domains on Uniprot. Enriched proteins are defined as proteins with fold changes > 3 and p-values < 0.05 relative to the appropriate ‘C’ probes, with the exception of the ‘C’ probes themselves which are defined as enriched if their fold-changes relative to DMSO are > 10 and p-values < 0.01.

Figure S20

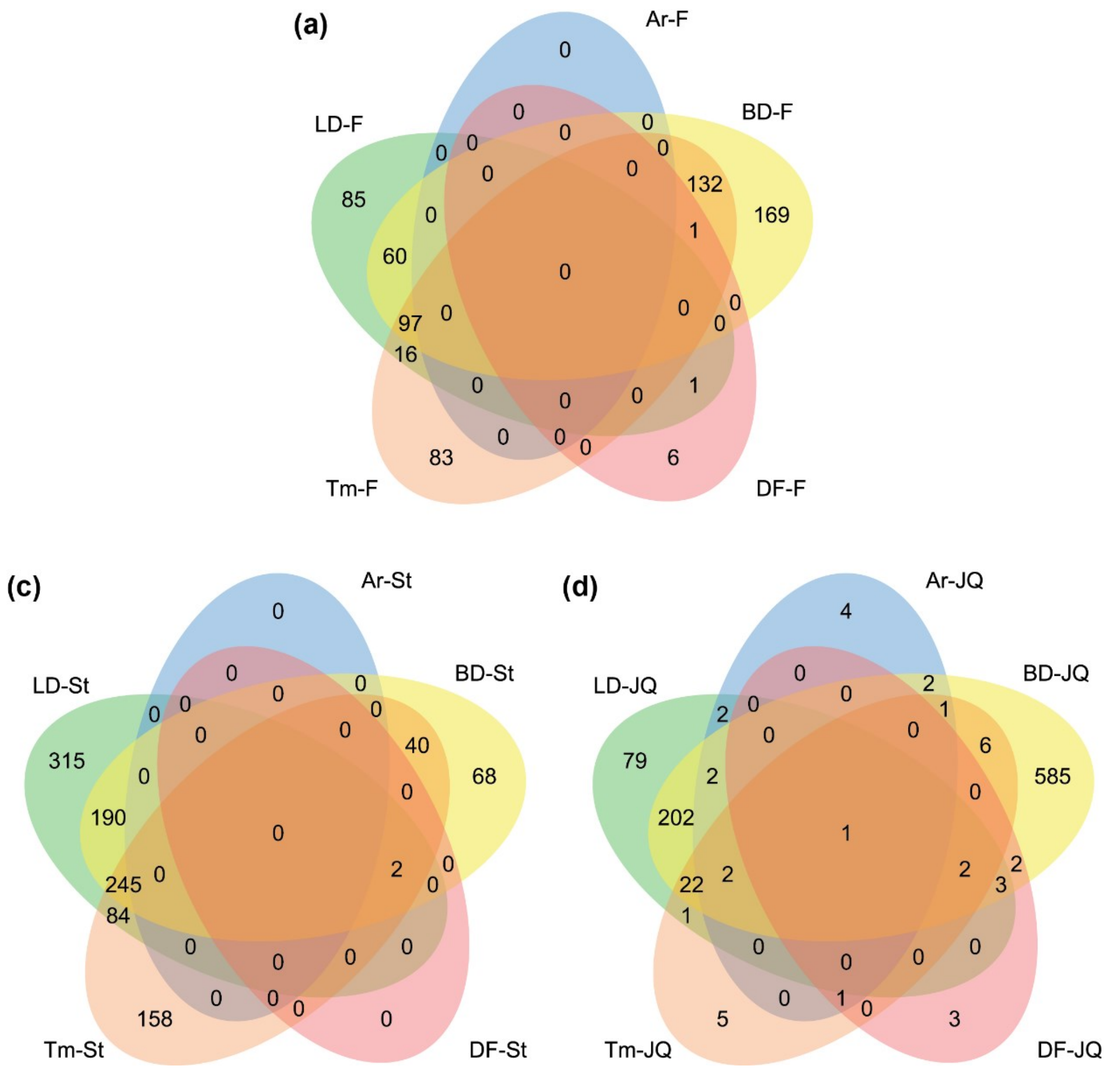

Figure 20. Venn diagrams illustrating the intersections of proteins enriched by each probe. Enriched proteins are defined as those with fold changes > 3 and p-values < 0.05 relative to the appropriate 'C' probes.

Biological Methods

UVP Crosslinker from Analytik Jena equipped with 5 × G8T5E UV bulbs (emission maximum 310 nm) or 5 × Hitachi F8T5 UV bulbs (emission maximum 365 nm)

a) Decomposition Kinetics Studies

Solutions of chemical probe (50 µL, 1-5 µM in isopropanol) were transferred in triplicate to a 96-well plate and irradiated with 365 or 302 nm UV light at 4 ºC. At each time point, a solution of *p*-nitroaniline (50 µL, 400 µM) was added to each well, mixed well by pipetting, and transferred to a HPLC vial for analysis. Samples were analyzed using a ThermoFisher UltiMate 3000 HPLC system fitted with a Thermo Scientific Syncronis C18 column (100 mm × 2.1 mm, 3 µm) and coupled to a ThermoFisher ISQ EC single quadrupole mass spectrometer. Buffer A was water with 0.1 % formic acid and buffer B was MeCN with 0.1% formic acid. The analytical method consisted of equilibration with 5 % buffer B for 4 min, before injection of the sample and a 12 min gradient (flow rate 0.5 mL/min, 40 °C), beginning with 5 % buffer B and rising to 95 % buffer B over 8 min, followed by isocratic elution for a further 4 min. The eluant was analyzed by mass spectrometry, selectively monitoring the $[M+H]^+$ signals of the chemical probe and *p*-nitroaniline.

b) Labeling of Cell Lysates with Photoaffinity Probes

For gel-based experiments, cells were grown in 15 cm plates ~90 % confluence at the time of treatment. For treatment of cell lysates, the cells were collected, washed with DPBS, and resuspended in 6 mL DPBS containing 1 × Halt protease inhibitor cocktail (Thermo Scientific). The cells were lysed by sonication (15 ms on, 40 ms off, 15 % amplitude, 1 s total on x 3) and centrifuged (100,000 × *g*, 45 min). The soluble fractions were retained and the pellets were resuspended in cold DPBS (2 ml supplemented with 1 × protease inhibitor cocktail) by sonication. Protein concentrations were normalized (2 mg / mL, final volume of 6 mL with DPBS 1X Halt Protease Inhibitor Cocktail) using the Lowry Protein Assay (Pierce). Samples of soluble and particulate cell lysate (250 µL) were combined with solutions of probes (250 µL in DPBS supplemented with protease inhibitor) in 12-well plates.

c) Labeling of Live Cells with Photoaffinity Probes

For the treatment of intact cells, the medium was aspirated, and the cells were incubated with 1 mL of serum-free media containing the appropriate probe for 30 min at 37 °C. The plates were then directly exposed to 365 nm UV light (Stratagene, UV Stratalinker 1800) for 15 min. For no-UV experiments, cells were incubated at 4 °C for 15 min protected from light. Following treatment and photo crosslinking, cells were harvested in cold DPBS by scraping, centrifuged (1700 g, 3 min, 4 °C), and pellets washed with cold DPBS and then aspirated (×2). Pellets were either directly processed or kept frozen at -80 °C until use. Cell pellets were lysed in cold DPBS (100-500 μL) using a Branson Sonifier probe sonicator (7 pulses, 15 % duty cycle, output setting = 4). For experiments requiring fractionation into particulate and soluble proteomes, cell lysates were centrifuged (100,000 × *g*, 45 min). The soluble fractions were retained and the pellets were resuspended in cold DPBS by sonication. Protein concentration was determined using the DC Protein Assay (Bio-Rad) and absorbance read using a Tecan, Infinite F500 plate reader following manufacturer's instructions.

d) Gel-based Analysis of Crosslinked Proteins

To each sample (1.5 mg/mL, 50 μL), 6 μL of freshly prepared 'click' mixture (3 μL of 1.7 mM TBTA in 4:1 *t*-BuOH:DMSO, 1 μL of 50 mM $CuSO_4$ in H_2O, 1 μL of 1.25 mM tetramethylrhodamine (TAMRA) azide in DMSO, 1 μL of freshly prepared 50 mM TCEP in DPBS) was added. The reaction mixture was mixed by pipetting and incubated at room temperature for 1 h before quenching with 4X SDS gel loading buffer (17 μL). Proteins (30 μg total protein loaded per gel lane) were resolved by SDS-PAGE (10 % acrylamide) made in-house and visualized by in-gel fluorescence on a Bio-Rad ChemiDoc MP Imaging System. The images were processed using Image Lab (version 5.2.1) software.

e) Fragment Probe Cell Treatments

For MS-based experiments, separate 10 cm dishes of HEK 293T cells were grown to 80-95% confluency with FBS-supplemented DMEM media. The growth medium was aspirated, and the cells were washed with Dulbecco's phosphate buffered saline (DPBS). The cells were incubated with serum-free media containing fragment probes

(200 μM) or control probes (200 μM) for 30 minutes at 37 °C under a 5 % CO_2 atmosphere. The cultures were irradiated under UV light (365 nm or 302 nm, 20 min), scraped, washed with cold DPBS, and collected into 15 mL centrifuge tubes, then transferred to 1.5 mL Eppendorf tubes. The cell suspensions were centrifuged (1,400 g, 3 min) and the pellets were stored at -80 °C until the next stage of processing.

f) Protein Kinase A Staurosporine Probe Activity Assay

Protein Kinase A (PKA) catalytic subunit inhibition was determined using the Kinase-Glo Luminescent Kinase Assay system (Promega) following manufacturer's guidelines. Briefly, PKA (0.5 units / well) was incubated with ATP (1 μM) and Kemptide substrate (5 μM) in a white-walled 96 well plate in triplicate. The solution was incubated at room temperature for 10 min containing different concentrations of St-series probes (0.05-10 μM) or unconjugated staurosporine (0.05-10 μM). The same volume of DMSO was used as a negative control. After treatment, inhibition was assayed; data represents the average and standard deviation of triplicates in measured luminescence.

g) Antiproliferation Assay of JQ1 Probe Bromodomain Inhibitors

Cell viability was determined using the CellTiter-Glo Luminescent Cell Viability Assay (Promega) following manufacturer's guidelines. NCI-H1299 cells were plated at 5000 cells/well in 96-well plates in RPMI 1640 supplemented with FBS, HEPES, and sodium pyruvate. THP-1 cells were plated in RPMI 1640 media supplemented with FBS. The cells were treated in triplicate with media containing different concentrations of JQ-series probes or unconjugated JQ1 (2.4 nM - 50 μM). Probes were applied via 20 mM DMSO stocks, of which the DMSO content never exceeded 1 % of the total media. The same volume of DMSO was used as a negative control. Fresh media containing each probe was reapplied to the NCI-H1299 cells after 48 hours. After the respective incubation time, cell proliferation was assayed; data represents the average and standard deviation of triplicates in measured luminescence.

h) JQ-1 Probe Cell Treatments

For MS-based experiments, K562 cells were grown in T-175 suspension flasks with FBS-supplemented RPMI-1640 media. Cells (3×10^8) were collected, washed twice with serum-free RPMI-1640, and resuspended in 30 mL serum-free RPMI-1640. Cell suspensions (3 mL per treatment) were aliquoted into 15 mL centrifuge tubes and JQ-1 probes, control probes, or DMSO was added (final concentration 10 µM). The suspensions were pipetted up and down to mix and placed in separate 10 cm dishes. The cells were incubated for 30 minutes at 37 °C under a 5% CO_2 atmosphere. The cultures were then irradiated under UV light (365 nm or 302 nm, 20 min), scraped, washed with cold DPBS, collected into 15 mL centrifuge tubes, and transferred to 1.5 mL Eppendorf tubes. The cell suspensions were centrifuged (1,400 g, 3 min) and the pellets were stored at -80 °C until the next stage of processing.

i) Staurosporine Probe Lysate Treatments

For MS-based experiments, K562 cells were grown in T-175 suspension flasks with FBS-supplemented RPMI-1640 media. Cells (3×10^8) were collected, washed with DPBS, and resuspended in 6 mL DPBS containing Halt Protease Inhibitor Cocktail. The cells were lysed by sonication (15 ms on, 40 ms off, 15 % amplitude, 1 s total on x 3). Protein concentrations were normalized (4 mg / mL, final volume of 6 mL with DPBS 1X Halt Protease Inhibitor Cocktail) using the Lowry Protein Assay (Pierce). Samples of cell lysate (500 µL) were combined with solutions of staurosporine probes or control probes (500 µL, 40 µM in DPBS supplemented with protease inhibitor) in 6-well plates. The lysates were irradiated under UV light (365 nm or 302 nm, 20 min), then 500 µL of each treatment were collected in 15 mL centrifuge tubes and directly proceeded to downstream processing..

j) Preparation of Samples for TMT Mass Spectrometric Proteomics

Cell pellets were resuspended in 500 µL DPBS and lysed by sonication (15 ms on, 40 ms off, 15% amplitude, 1 s total on x 3). Protein concentrations were normalized (2 mg/mL in 500 µL with cold DPBS) as previously described. To each sample was added solutions of tris((1-benzyl-4-triazolyl)methyl)amine (30 µL, 1.7 mM in DMSO/*t*-BuOH 1:4 v/v), tris(2-carboxyethyl)phosphine (10 µL, 50 mM), biotin-PEG3-azide (5 µL, 100 µM), and $CuSO_4$ (10 µL, 50 mM) and shaken at room temperature for 1 h. After

reaction, 2.5 mL of cold 4:1 MeOH/$CHCl_3$ solution and 1 mL cold DPBS 4 °C was added, the samples were vortexed then were centrifuged (4,700 x *g,* 10 min, 4 °C), forming a disc. The supernatant was removed, and the samples were washed three times with 4:1 MeOH/$CHCl_3$, resuspended by sonication and centrifuged as previously described. Pellets were resuspended in freshly prepared urea solution (500 µL DPBS, 6 M) and a solution of SDS (10 µL of 10 % *w/v*), followed by freshly prepared 1:1 solution (50 µL) TCEP (200 mM in DPBS) and K_2CO_3 (600 mM in DPBS) and incubated (30 min, 37 °C) while shaking. After reaction, a solution of freshly prepared iodoacetamide (70 µL, 400 mM in DPBS) was added and incubated for 30 min at room temperature while protected from light. A solution of SDS (130 µL, 10 % in DPBS *w/v*) was added, then diluted with DPBS (5.5 mL). A streptavidin-agarose slurry (100 µL, 50 %, Pierce) was added to each tube and rotated for 1.5 h at RT. The beads were pelleted by centrifugation (750 x *g*, 2 min, 4 °C), washed (1 x 5 mL 0.2% *w/v* SDS in DPBS, 1 x 5 mL DPBS, 1 x 5 mL 100 mM TEAB pH 8.5 in Milliq water) and transferred to 1.5 mL LoBind microcentrifuge tubes with TEAB (1 mL, 100 mM) and pelleted by centrifugation (750 x *g*, 2 min, 4 °C). The supernatant was aspirated, and sequencing-grade modified porcine trypsin (200 µL, 100 mM TEAB pH 8.5, 100 µM $CaCl_2$) was added to the beads and incubated at 37 °C for 14 h with shaking. The digest was separated by centrifugation (750 *x g*, 5 min, 4 °C) and placed in new LoBind centrifuge tubes. The beads were washed with TEAB (100 µL, 100 mM pH 8.5 in Milliq water), and the washing was separated and combined with the supernatant (300 µL total peptide mix per sample). MS-grade acetonitrile (120 µL) was added to each and samples were labeled with respective TMT 10 plex isotope (8 µL, 20 µg/ µL) for 1 h with occasional vortexing at RT. To quench, hydroxylamine (6 µL, 5% *v/v*) was added to each sample, vortexed, and incubated for 15 min at RT. Formic acid (4 µL) was added to each tube to acidify and the samples were dried under vacuum centrifugation. The samples were combined by redissolving the contents of one tube in a solution of trifluoroacetic acid (TFA, 200 µL, 0.1% in water) and transferred into each sample until all samples were redissolved. The step-wise process was repeated with a second volume of TFA solution (100 µL in water) for a final volume of 300 µL. The samples were fractionated using a fractionation kit (Pierce high pH Reversed-Phase Fractionation Kit, Thermo Fisher Scientific 84868) according to manufacturer's instructions. The peptide fractions were eluted from the spin column with consecutive solutions of 0.1% triethylamine combined with MeCN (5-95% MeCN). The fractions

were combined pairwise (fraction 1 and fraction 7, fraction 2 and fraction 8, etc.), dried via vacuum centrifugation, and stored at -80 °C until ready for mass spectrometer injection.

k) LC-MS Analysis of TMT samples

TMT labeled samples were redissolved in MS buffer A (20 µL, 0.1% formic acid in water). 3 µL of each sample was loaded onto an Acclaim PepMap 100 precolumn (75 µm x 2 mm) and eluted on an Acclaim PepMap RSLC analytical column (75 µm x 15 cm) using the UltiMate 3000 RSLCnano system (Thermo Fisher Scientific). Buffer A was prepared as described above and buffer B (0.1% formic acid in MeCN) were used in a 220 min gradient (flow rate 0.3 mL min, 35 °C) of 2 % buffer B for 10 min, followed by an incremental increase to 30 % buffer B over 192 min, 60 % buffer B for 5 min, 60-95 % buffer B for 1 min, hold at 95 % buffer B for 5 min, followed by descent to 2% buffer B for 1 min followed by re-equilibration at 2 % for 6 min. The elutions were analyzed with a Thermo Fisher Scientific Orbitrap Fusion Lumos mass spectrometer with a cycle time of 3 s and nano-LC electrospray ionization source applied voltage of 2.0 kV. MS^1 spectra were recorded at a resolution of 120,000 with an automatic gain control (AGC) value of $1x10^6$ ions, maximum injection time of 50 ms (dynamic exclusion enabled, repeat count 1, duration 20 s). The scan range was specified from 375 to 1,500 m/z. Peptide fragmentation MS^2 spectra was recorded via collision-induced diffusion (CID) and quadrupole ion trap analysis (AGC $1.8x10^4$, 30 % collision energy, maximum inject time 120 ms, isolation window 1.6). MS^3 spectra was generated by high-energy collision-induced dissociation (HCD) with collision energy of 65 %. Precursor selection included up to 10 MS^2 ions for the MS^3 spectrum.

l) TMT Proteomics Data Analysis

Proteomic analysis was performed with the processing software Proteome Discoverer 2.4 (Thermo Fisher Scientific). Peptide sequences were identified by matching proteome databases with experimental fragmentation patterns via the SEQUEST HT algorithm. Fragment tolerances were set to 0.6 Da, and precursor mass tolerances set to 10 ppm with one missed cleavage site allowed. Carbamidomethyl (C, +57.02146) and TMT-tag (K and N-terminal, +229.1629) were specified in the modifications

selection as static while oxidation (M, +15.994915) was defined as variable. For peptide localization experiments, the following dynamic modifications were included: for LD-F 545.3240 or 551.3378); for Ar-F 663.2907 or 669.3045; for BD-F 545.3240 or 551.3378; for DF-F 569.2688 or 575.2826; for Tm-F 517.2927 or 523.3065. Spectra were searched against the *Homo Sapiens* proteome database (42,358 sequences) using a false discovery rate of 1 % (Percolator). MS^3 peptide quantitation was performed with a mass tolerance of 20 ppm. Abundances in each channel were normalized to the naturally biotinylated protein ACACA. Identified proteins were required to have at least two unique peptides. TMT ratios obtained by Proteome Discoverer were transformed with $\log_2(x)$, and p-values were calculated via Student's two-tailed t-tests with two biological replicates. Quantitative data are listed in the supplementary proteomics data spreadsheet. The mass spectrometric datasets will be deposited after acceptance for publication.

I) Preparation of Samples for Site of Labeling Experiment

HEK293t cells were grown in 10-cm plates with DMEM medium supplemented with 10 % FBS to ~90 % confluence at the time of treatment. The medium was aspirated, and the cells were incubated with 1 mL of serum-free media containing the 'F' series fragment probe for 30 min at 37 °C in duplicate. The plates were then directly exposed to 365 nm UV light (Stratagene, UV Stratalinker 1800) for 20 min. Cells were harvested in cold DPBS by scraping, centrifuged (1700 g, 3 min, 4 °C), washed with cold DPBS and then aspirated (×2). Cell pellets were resuspended in 500 µL DPBS and lysed by sonication (15 ms on, 40 ms off, 15% amplitude, 1 s total on x 3). Protein concentrations were normalized (2 mg/mL in 500 µL with cold DPBS). To each sample was added solutions of tris((1-benzyl-4-triazolyl)methyl)amine (30 µL, 1.7 mM in DMSO/*t*-BuOH 1:4 v/v), tris(2-carboxyethyl)phosphine (10 µL, 50 mM), cleavable biotin azide light tag (L6) or cleavable biotin azide heavy tag (L6) (5 µL, 100 µM), and $CuSO_4$ (10 µL, 50 mM) and shaken at room temperature for 1 h. A cold MeOH/$CHCl_3$ solution (4:1, 2.5 mL) solution was added, followed by 1 mL cold DPBS. The samples were vortexed, then were centrifuged (3,000 x *g,* 10 min, 4 °C). The supernatant was removed, and the samples were washed with 4:1 MeOH/$CHCl_3$, resuspended by sonication and again centrifuged. Pellets were resuspended in freshly prepared urea solution (500 µL, 6 M in DPBS) and a solution of SDS (10 µL, 10 % *w/v*), followed by

a freshly prepared 1:1 solution of TCEP (200 mM in DPBS) and K_2CO_3 (600 mM in DPBS) (50 µL) and incubated (30 min, 37 °C) with shaking. A solution of freshly prepared iodoacetamide (70 µL, 400 mM in DPBS) was then added and incubated for 30 min at room temperature while protected from light. After addition of a solution of SDS (130 µL, 10 % in DPBS *w/v*), the 'heavy' and 'light" biotin probe-labeled proteomes were combined in a 1:1 ratio, then diluted with DPBS (11 mL). A streptavidin-agarose slurry (200 µL, 50 %, Pierce) was added to each tube and the tubes were rotated for 1.5 h at RT. The beads were pelleted by centrifugation (500 x *g*, 2 min, 4 °C), washed (1 x 5 mL 0.2 % *w/v* SDS in DPBS, 1 x 5 mL DPBS, 1 x 5 mL 100 mM TEAB pH 8.5 in Milli-Q water) and transferred to 1.5 mL LoBind microcentrifuge tubes with TEAB (1 mL, 100 mM) and pelleted by centrifugation (500 x *g*, 2 min, 4 °C). The supernatant was aspirated, and sequencing-grade modified porcine trypsin (100 µL, 100 mM TEAB pH 8.5, 100 µM $CaCl_2$, 1 M urea) was added to the beads and incubated at 37 °C for 14 h with shaking. The beads were transferred to tube filters (Corning, SPINX8160), and the digested peptides were removed by centrifugation (400 × *g*, 5 min). The beads were washed with SDS in DPBS (0.2 % *w/v*, 0.5 mL), NaCl in DPBS (2 × 0.5 mL, 150 mM) and H_2O (2 × 0.5 mL) by centrifugation (400 × *g*, 5 min). The beads were transferred to LoBind microcentrifuge tubes with water, and the water was removed by centrifugation (500 x *g*, 10 min). The beads were incubated with a solution of formic acid/water (200 µL, 2 % *v/v*) for 1 h with gentle shaking. The supernatant was collected and the remaining beads were again subjected to this treatment. The supernatants were combined. The beads were then washed with acetonitrile/water (50 % v/v with 1 % formic acid, 400 µL). The wash was combined with the supernatants, and solvents were removed with vacuum centrifugation. The residue was redissolved in TFA solution (0.1 % in water *v/v*) and desalted with a C-18 desalting column (Thermo Fisher Scientific, 89870). The solvents were removed from the eluant by vacuum centrifugation and the residue was stored at -80 °C until ready for mass spectrometric analysis.

m) LC-MS Analysis of Site of Labeling Samples

Site of labeling samples were redissolved in MS buffer A (10 µL, 0.1% formic acid in water). 4 µL of each sample was loaded onto an Acclaim PepMap 100 precolumn (75 µm x 2 mm) and eluted on an Acclaim PepMap RSLC analytical column (75 µm x 15

cm) using the UltiMate 3000 RSLCnano system (Thermo Fisher Scientific). Buffer A was prepared as described above and buffer B (0.1% formic acid in MeCN) were used in a 120 min gradient (flow rate 0.3 mL / min, 35 °C) consisting of 2 % buffer B for 10 min, followed by an incremental increase to 35 % buffer B over 90 min, 60 % buffer B for 8 min, 60-95 % buffer B over 2 min, hold at 95 % buffer B for 8 min, followed by descent to 2% buffer B for 2 min followed by re-equilibration at 2 % for 10 min. The eluants were analyzed with a Thermo Fisher Scientific Orbitrap Fusion Lumos mass spectrometer with a cycle time of 3 s and nano-LC electrospray ionization source applied voltage of 2.0 kV. MS^1 spectra were recorded at a resolution of 60,000 with an automatic gain control (AGC) value of $4x10^5$ ions, maximum injection time of 50 ms (dynamic exclusion enabled, repeat count 1, duration 30 s), with a scan range of 375 to 2000 m/z. Peptide fragmentation MS^2 spectra was generated by high-energy collision-induced dissociation (HCD) with a collision energy of 28 %. The detector type was orbitrap, with a 15,000 resolution (AGC $5x10^4$, maximum inject time 110 ms, isolation window 1.6).

n) Site of Labeling Data Analysis

Photoaffinity probe binding site analysis was performed with the processing software Proteome Discoverer 2.4 (Thermo Fisher Scientific). Peptide sequences were identified by matching proteome databases with experimental fragmentation patterns via the SEQUEST HT algorithm. Fragment tolerances were set to 0.6 Da, and precursor mass tolerances set to 10 ppm with two missed cleavage sites allowed. Carbamidomethyl (C, +57.02146) was specified as static modification. Oxidation (M, +15.994915) was defined as variable modifications. Photoaffinity probe binding sites were assigned as variable modifications on any amino acid (LD-F, L: +545.3240, H: +551.3378; Ar-F, L: +663.2907, H: + 669.3045; BD-F, L: +545.3240, H: +551.3378; DF-F, L: +569.2688, H: +575.2826; Tm-F, L: +517.2927, H: +523.3065; L: probe with light tag, H: probe with heavy tag). For each probe, separate analyses were performed for the light and heavy modifications. Spectra were searched against the *Homo sapiens* proteome database (42,358 sequences) using a false discovery rate of 1 % (Percolator). Peptides were considered to be probe-modified with high confidence if the peptides were found to carry the probe modification in both the ‘heavy’ and ‘light’

analyses regardless of the assigned position of the modification or the presence of any other modifications.

The mass spectrometry proteomics data have been deposited to the ProteomeXchange Consortium via the PRIDE[2] partner repository with the dataset identifier PXD025652

Synthetic Methods

Chemicals and reagents were purchased from commercial vendors, including Sigma-Aldrich, Fisher Scientific, Combi-Blocks, MedChemExpress, Alfa Aesar and AstaTech, and were used as received without further purification, unless otherwise noted. Anhydrous solvents were purchased from Sigma-Aldrich in Sure/Seal™ formulations. All reactions were monitored by thin-layer chromatography (TLC, Merck silica gel 60 F-254 plates). The plates were stained either with *p*-anisaldehyde (2.5 % *p*-anisaldehyde, 1 % AcOH, 3.5 % H_2SO_4 (conc.) in 95 % EtOH), ninhydrin (0.3 % ninhydrin in 97:3 EtOH-AcOH (w/*v*)), $KMnO_4$ (1.5 g $KMnO_4$, 10 g K_2CO_3, and 1.25 mL 10 % NaOH in 200 mL water), iodine or directly visualized with UV light. Reaction purification was carried out using Flash chromatography (230 – 400 mesh silica gel), Biotage® or preparative thin layer chromatography (pTLC, Analtech, 500-2000 µm thickness). NMR spectra were recorded on Bruker DPX-400 or Bruker AV-500 spectrometers in the indicated solvent. Multiplicities are reported with the following abbreviations: s singlet; d doublet; t triplet; q quartet; p pentet; m multiplet; br broad; dd doublet of doublets; dt doublet of triplets; td triplet of doublets; Chemical shifts are reported in ppm relative to the residual solvent peak and *J* values are reported in Hz. Mass spectrometry data were collected on an Agilent 6120 single-quadrupole LC/MS instrument (ESI, low resolution) or an Agilent ESI-TOF instrument (ESI-TOF, HRMS).

a) Synthesis of terminal diazirine alkyne tag *N*-((3H-diazirin-3-yl)methyl)prop-2-yn-1-amine (Tm)

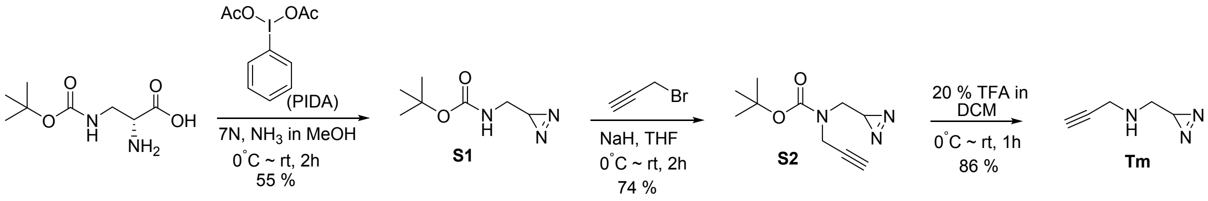

Tert-butyl ((3H-diazirin-3-yl)methyl)carbamate (S1) was synthesized according to literature procedures.[3] To a solution of *N*-β-Boc-D-2,3-diaminopropionic acid (1.0 g, 4.9 mmol) in a methanolic solution of ammonia (12.2 mL, 7 N, 85.7 mmol), (diacetoxyiodo)benzene (4.73 g, 14.7 mmol) was added in one portion at 0 °C under

argon. After 30 min at 0 °C, the mixture stirred at room temperature for 1 h. After completion (monitored by TLC), methanol was evaporated under reduced pressure and the crude residue was purified by Biotage® SNAP Cartridge KP-Sil 10 g with a 0-20 % linear gradient of ethyl acetate in hexane over 20 column volumes (CV), to obtain S1 as a light brown oil (461 mg, 55 %). ^{1}H NMR (400 MHz, $CDCl_3$) δ 4.55 (s, 1H), 3.07 (t, *J* = 5.0 Hz, 2H), 1.47 (s, 9H), 1.10 (t, *J* = 4.0 Hz, 1H); ^{13}C NMR (100 MHz, $CDCl_3$): δ 155.7, 80.0, 40.6, 28.3, 20.6.

Tert-butyl ((3H-diazirin-3-yl)methyl)(prop-2-yn-1-yl)carbamate (S2): A solution of tert-butyl ((3H-diazirin-3-yl)methyl)carbamate (400 mg 1.5 mmol) in dry THF (5 mL) was dropwise added to a stirred solution of sodium hydride (41 mg, 1.7 mmol) in dry THF (5 mL) at 0 °C, over 10 min an atmosphere of argon and the resulting solution was stirred for 20 min. Propargyl bromide (202 mg, 129 µL, 1.70 mmol) was added dropwise at 0 °C and the resulting mixture was stirred at room temperature for an additional 2 h. After completion (monitored by TLC), the resulting solution was evaporated and redissolved in water (10 mL) and extracted with diethyl ether (50 mL ×3). The combined extracts were washed with saturated aqueous $NaHCO_3$ solution (10 mL), dried over anhydrous Na_2SO_4, and evaporated under reduced pressure. The crude product was purified by Biotage® SNAP Cartridge KP-Sil Snap 10 g with a 0-20 % linear gradient of ethyl acetate in hexane over 20 column volumes (CV) to obtain S2 as a light brown oil (362 mg, 74 %). ^{1}H NMR (400 MHz, $CDCl_3$) δ 4.13 (s, 1H), 3.26 (d, *J* = 4.0 Hz, 2H), 2.26 (d, *J* = 4.0 Hz, 1H), 1.51 (s, 9H), 1.12 (t, *J* = 4.0 Hz, 1H); ^{13}C NMR (100 MHz, $CDCl_3$): δ 154.6, 81.1, 78.9, 72.3, 71.2, 46.0, 37.2, 36.6, 28.4, 28.3, 20.0.

N-((3H-diazirin-3-yl)methyl)prop-2-yn-1-amine (Tm): To a solution of tert-butyl ((3H-diazirin-3-yl)methyl)(prop-2-yn-1-yl)carbamate (**S2**) (350 mg, 0.113 mmol) in DCM (3 mL), a solution of 20 % TFA in DCM (1 mL) was added at 0 °C and resulting mixture was stirred at room temperature for 2 h. After completion (monitored by TLC), the reaction mixture was evaporated under reduced pressure to obtained the TFA salt of *N*-((3H-diazirin-3-yl)methyl)prop-2-yn-1-amine **Tm** as a dark brown oil (286 mg, 86 %). 1H NMR (400 MHz, $CDCl_3$) δ 3.96 (d, *J* = 4.0 Hz, 2H), 3.01 (d, *J* = 4.0 Hz, 2H), 2.52 (t, *J* = 4.0 Hz, 1H), 1.27 (t, *J* = 4.0 Hz, 1H); ^{13}C NMR (100 MHz, $CDCl_3$): δ 78.7, 71.6, 47.5, 36.4, 16.0.

b) Synthesis of N-(2-(3-methyl-3H-diazirin-3-yl)ethyl)prop-2-yn-1-amine:

Prepared according to a literature procedures.[4] Analytical data are identical to previously reported data.

c) Synthesis of (3-(1,1-difluoro-3-(triisopropylsilyl)prop-2-yn-1-yl)-3H-diazirin-3-yl)methanol: Prepared according to literature procedures.[5] Analytical data are identical to previously reported data.

d) General procedures for the synthesis of diazirine containing tags:

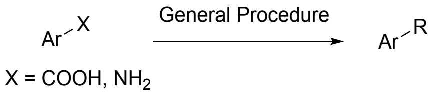

General Procedure 1: To a solution of the appropriate carboxylic acid (0.113 mmol) in DMC (3 mL), the corresponding diazirine amine (0.118 mmol), DIPEA (45.8 mg, 61.7 µl, 0.354 mmol), EDC-HCl (33.9 mg, 0.177 mmol), and HOBt (23.9 mg, 0.177 mmol) were added. The reaction mixture was stirred at room temperature for 14 to 16 h. After completion (monitored by TLC) the crude reaction mixture was diluted with DCM (20 mL) and washed first with saturated aqueous NH_4Cl (10 mL) and saturated aqueous $NaHCO_3$ (10 mL) solution, dried over anhydrous Na_2SO_4 and solvents were were removed by rotary evaporation. Crude products were purified by PTLC or Biotage® SNAP Cartridge KP-Sil Snap 10 g with a 0-40 % linear gradient of ethyl acetate in hexane over 20 column volumes (CV).

General Procedure 2: To a solution of the carboxylic acid (0.118 mmol) in DMF (2 mL), HATU (49.4 mg, 0.130 mmol) and DIPEA (45.8 mg, 61.7 µl, 0.354 mmol) were added at 0 °C and resulting mixture was stirred for 5 min. The corresponding diazirine-containing amine was added, and the resulting mixture was stirred at room temperature for 1 h. The crude mixture was diluted with cold water (10 mL) and extracted with ethyl acetate (20 mL × 2). The combined organic extracts were then dried over anhydrous Na_2SO_4 and solvents were removed by rotary evaporation. Crude products were purified using a Biotage® SNAP Cartridge KP-Sil Snap 10 g with 0-40 % linear gradient of ethyl acetate in hexane over 20 column volumes (CV).

General Procedure 3: To a solution of amine (0.113 mmol) in DCM (3 mL), the corresponding diazirine-containing acid (0.118 mmol), DIPEA (45.8 mg, 61.7 µl, 0.354 mmol), EDC-HCl (33.9 mg, 0.177 mmol), and HOBt (23.9 mg, 0.177 mmol) were added. The reaction mixtures were stirred at room temperature for 14 to 16 h. After completion (monitored by TLC) the crude samples were diluted with DCM (20 mL) and washed first with saturated aqueous NH_4Cl (10 mL) and saturated aqueous $NaHCO_3$ (10 mL), then dried over anhydrous Na_2SO_4 and solvents were removed by rotary evaporation. Crude products were purified by Biotage® SNAP Cartridge KP-Sil Snap 10 g with a 0-40 % linear gradient of ethyl acetate in hexane over 20 column volumes (CV).

General Procedure 4: Carbamates were prepared based on the procedure reported by Chang, et al.[5] Carbonyldiimidazole (162 mg, 1.38 mmol) was added to a stirred solution of (3-(1,1-difluoro-3-(triisopropylsilyl)prop-2-yn-1-yl)-3H-diazirin-3-yl)methanol (34 mg, 0.082 mmol) in tetrahydrofuran (2.0 mL). The reaction mixture was stirred for 12 h at 24 ˚C. The mixture was concentrated by rotary evaporation and the crude carbamate intermediate was dissolved in anhydrous dichloromethane (2.0 mL). The corresponding amine (0.113 mmol) was added to the reaction mixture, followed by 4-dimethylaminopyridine (2.3 mg, 19 µmol) and the resulting solution was stirred for 48 h at room temperature. The crude product was dried by rotary evaporation, dissolved in anhydrous tetrahydrofuran (2.0 mL) and a solution of tetra-*N*-butylammonium fluoride in THF (52.7 µL, 1 M, 52.7 mmol) and acetic acid (3.2 mg, 3.0 µL, 52.7 mmol) were added in sequence at –78 ˚C. The reaction was stirred continuously for 20 min at –78 ˚C allowed to rise to room temperature over an additional 10 min. The reaction mixture was quenched by saturated aqueous ammonium chloride solution (1 mL) and extracted with dichloromethane (2 × 20 mL). The combined organic extracts were washed with brine, dried over anhydrous Na_2SO_4, and solvents were removed by rotary evaporation. The crude products were purified by Biotage® SNAP Cartridge KP-Sil 10 g with a 0-40 % linear gradient of ethyl acetate in hexane over 20 column volumes (CV) to afford the probe.

e) Procedure for the synthesis of staurosporine amine linker:

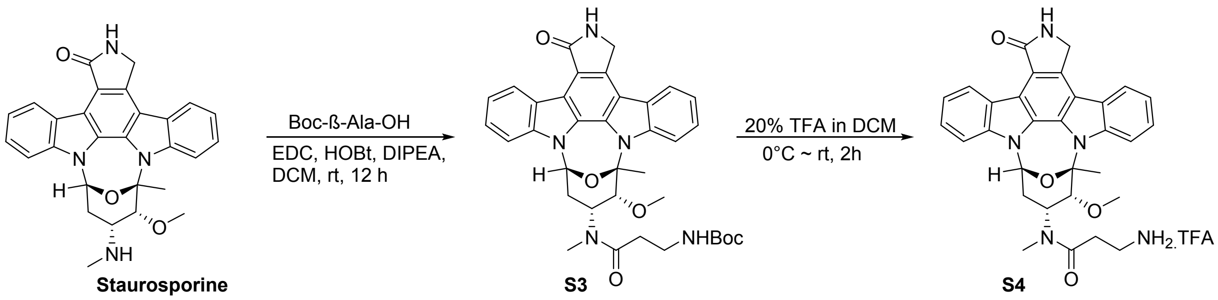

Synthesis of Staurosporine intermediate (S3): To a solution of staurosporine (10 mg, 21 μmol) in dichloromethane (2 mL), Boc-β-Ala-OH (4.5 mg, 23 μmol), DIPEA (12 μL, 64 μmol), EDC-HCl (6.2 mg, 32 μmol), and HOBt (4.4 mg, 32 μmol) were added at 0 °C and resulting mixture was stirred at room temperature for 12 h. After completion (monitored by TLC) a solution of TFA in water (2 mL, 0.1 % *v/v*) and the mixture was extracted with dichloromethane (2 × 20 mL). The combined extracts were dried over anhydrous Na_2SO_4 and volatiles removed by rotary evaporation. The crude products were purified by Biotage® SNAP Cartridge KP-Sil 10 g with a 0-5 % linear gradient of methanol in dichloromethane over 20 column volumes to afford the corresponding staurosporine intermediate (**S8**) as sticky liquid (10 mg, 76 %). ^{1}H NMR (600 MHz, $CDCl_3$) δ 9.36 (d, *J* = 7.9 Hz, 1H), 7.80 (dd, *J* = 7.8, 1.3 Hz, 1H), 7.64 (d, *J* = 8.4 Hz, 1H), 7.40 (t, *J* = 7.6 Hz, 1H), 7.36 (ddd, *J* = 8.4, 7.0, 1.3 Hz, 1H), 7.29 (t, *J* = 7.5 Hz, 1H), 7.25 (t, *J* = 7.4 Hz, 1H), 7.13 (d, *J* = 8.0 Hz, 1H), 6.77 (s, 1H), 6.61 – 6.54 (m, 1H), 5.27 (t, *J* = 6.4 Hz, 1H), 5.15 – 5.07 (m, 1H), 4.97 – 4.84 (m, 2H), 3.91 (d, *J* = 2.2 Hz, 1H), 3.47 – 3.31 (m, 2H), 2.72 (s, 3H), 2.52 – 2.44 (m, 4H), 2.39 (s, 3H), 2.36 (s, 3H), 1.38 (s, 9H). ^{13}C NMR (151 MHz, $CDCl_3$) δ 172.29, 171.55, 154.95, 137.51, 135.44, 131.44, 129.39, 125.64, 125.19, 124.38, 123.92, 123.58, 122.52, 120.41, 119.46, 119.00, 118.02, 115.17, 113.47, 111.25, 106.62, 93.50, 83.73, 81.36, 78.21, 59.35, 54.18, 47.44, 44.91, 35.17, 33.33, 30.11, 28.63, 28.05, 27.38, 26.96.

General Procedure 5: Synthesis of staurosporine amine linker intermediate (S4): To a stirred solution of intermediate **S3** (10 mg, 15 μmol) in dichloromethane (2 mL), a solution of trifluoroacetic acid in dichloromethane (20 % *v/v*, 2 mL) was added at 0 °C and the resulting mixture was stirred at room temperature for 3 h. Solvents were removed by rotary evaporation to obtain the TFA salt of **S4** as a light brown sticky liquid (9.4 mg, 95 %); ^{1}H NMR (600 MHz, DMSO) δ 9.34 – 9.25 (m, 1H), 8.64 (s, 1H),

8.06 (dd, J = 8.0, 1.3 Hz, 1H), 8.00 (dd, J = 8.3, 2.6 Hz, 1H), 7.66 (d, J = 8.2 Hz, 1H), 7.49 (ddd, J = 8.4, 7.1, 1.3 Hz, 2H), 7.36 (t, J = 7.5 Hz, 1H), 7.30 (td, J = 7.4, 6.9, 1.0 Hz, 1H), 7.03 (dd, J = 8.6, 6.6 Hz, 1H), 5.08 – 5.01 (m, 1H), 5.04 – 4.96 (m, 2H), 4.26 (dd, J = 2.7, 1.6 Hz, 1H), 2.83 – 2.77 (m, 4H), 2.76 (s, 4H), 2.35 (s, 3H). ^{13}C NMR (151 MHz, DMSO) δ 179.83, 177.58, 177.12, 144.08, 141.47, 137.89, 137.85, 134.39, 130.90, 130.54, 130.24, 130.20, 128.97, 127.85, 126.67, 125.52, 124.68, 124.61, 120.40, 119.33, 114.21, 99.88, 88.50, 87.48, 65.68, 53.04, 50.65, 43.47, 43.11, 42.40, 36.14, 34.66, 32.01, 30.50.

f) Synthetic procedures of JQ1 amine linker:

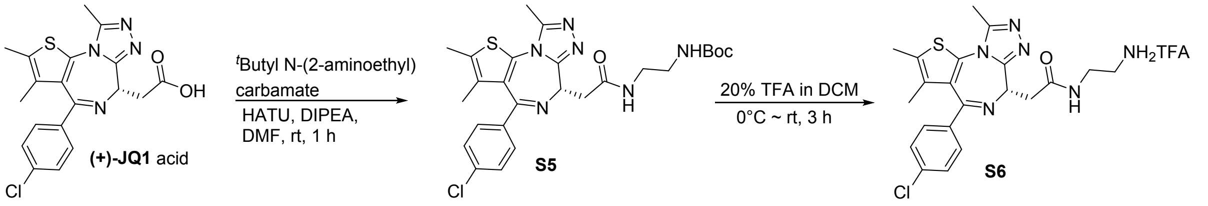

Synthesis of JQ1 intermediate (S5): To a solution of (+)-JQ1 acid (30 mg, 74 μmol) in DMF (2 mL), HATU (32 mg, 83 μmol) and DIPEA (39 μL, 0.22 mmol) were added at 0 °C and the resulting mixture was stirred for 5 min. tert-Butyl *N*-(2-aminoethyl)carbamate (14 mg, 82 μmol) was then added and resulting mixture was stirred at 0 °C for 1 h. The crude mixture was diluted with cold water (10 mL) and extracted with ethyl acetate (20 mL × 2). The combined extracts were dried over anhydrous Na_2SO_4 and volatiles removed by rotary evaporation. The crude products were purified by Biotage® SNAP Cartridge KP-Sil Snap 10 g column with a 0-60 % linear gradient of ethyl acetate and hexane over 20 column volumes (CV) to obtain corresponding intermediate **S5** as a sticky liquid (29 mg, 72 %); ^{1}H NMR (400 MHz, $CDCl_3$) δ 7.70 (t, J = 5.5 Hz, 1H), 7.39 (d, J = 8.6 Hz, 2H), 7.31 (d, J = 8.7 Hz, 2H), 5.71 (d, J = 5.5 Hz, 1H), 4.71 (t, J = 7.1 Hz, 1H), 3.58 (dd, J = 14.7, 7.4 Hz, 1H), 3.54 – 3.41 (m, 2H), 3.41 – 3.21 (m, 3H), 2.69 (s, 3H), 2.41 (s, 3H), 1.68 (s, 3H), 1.42 (s, 9H). ^{13}C NMR (101 MHz, $CDCl_3$) δ 171.08, 163.88, 156.38, 155.80, 149.94, 136.75, 136.56, 132.00, 130.98, 130.90, 130.63, 129.99, 129.89, 128.69, 79.13, 76.82, 54.31, 40.80, 39.98, 38.93, 28.47, 14.40, 13.09, 11.82.

Synthesis of staurosporine amine linker intermediate (S6): Synthesized according to general procedure 5, to obtain TFA salt of **S6** as a light brown sticky liquid (27 mg,

95 %); ^{1}H NMR (400 MHz, $CDCl_3$) δ 8.58 (s, 1H), 7.40 (d, *J* = 8.5 Hz, 2H), 7.31 (d, *J* = 8.7 Hz, 2H), 4.66 (dd, *J* = 8.4, 6.0 Hz, 1H), 3.77 (s, 1H), 3.71 – 3.57 (m, 2H), 3.45 (s, 1H), 3.43 – 3.30 (m, 2H), 3.27 – 3.14 (m, 2H), 2.64 (s, 3H), 2.40 (s, 3H), 1.67 (s, 3H).

g) Procedures for the synthesis of JQ1 acid linker:

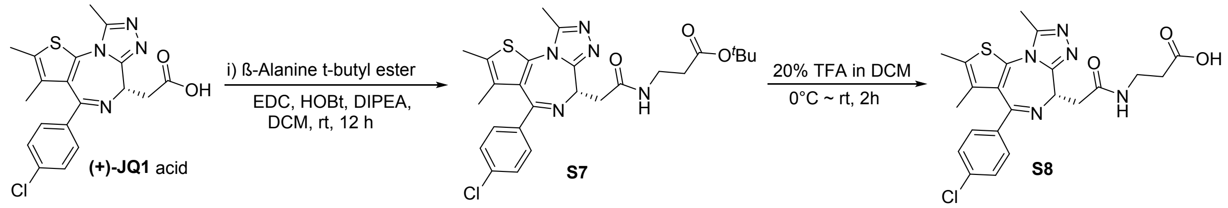

Synthesis of intermediate S7: To a solution of JQ1-acid (30 mg, 74 μmol) in DCM (3 mL), β-alanine t-butyl ester (15 mg, 82 μmol), DIPEA (29 mg, 39 μl, 0.22 mmol), EDC•HCl (21 mg, 0.11 mmol), and HOBt (16 mg, 0.11 mmol) were added. The reaction mixtures were stirred at room temperature for 12 h. After completion (monitored by TLC) the crude samples were diluted with DCM (20 mL) and washed with saturated aqueous NH_4Cl (10 mL) and saturated aqueous $NaHCO_3$ (10 mL), then dried over anhydrous Na_2SO_4 and volatiles were removed by rotary evaporation. Crude products were purified by Biotage® SNAP Cartridge KP-Sil Snap 10 g with a 0-40 % linear gradient of ethyl acetate in hexane over 20 column volumes (CV) to obtain **S7** as a sticky liquid (29 mg, 74 %), ^{1}H NMR (400 MHz, $CDCl_3$) δ 7.41 (d, *J* = 8.0, 2H), 7.32 (dt, *J* = 8.4, 2.5 Hz, 2H), 6.90 – 6.86 (m, 1H), 4.64 (t, *J* = 6.9 Hz, 1H), 3.56 – 3.48 (m, 3H), 3.41 – 3.35 (m, 1H), 2.66 (s, 3H), 2.55 – 2.44 (m, 2H), 2.40 (s, 3H), 1.67 (s, 3H), 1.45 (s, 9H). ^{13}C NMR (101 MHz, $CDCl_3$) δ 171.47, 170.43, 163.83, 155.68, 149.85, 136.72, 136.64, 132.17, 130.91, 130.74, 130.46, 129.86, 128.69, 80.98, 54.28, 39.20, 35.32, 35.27, 28.12, 14.40, 13.09, 11.84.

Synthesis of intermediate (S8): Synthesized according to general procedure 5, to obtain the TFA salt of **S8** as a sticky liquid (26 mg, 95 %) ^{1}H NMR (400 MHz, $CDCl_3$) δ 7.97 (s, 1H), 7.44 (d, *J* = 8.4 Hz, 2H), 7.40 (d, *J* = 8.7 Hz, 2H), 4.91 (t, *J* = 7.1 Hz, 1H), 3.66 – 3.43 (m, 4H), 2.85 (s, 3H), 2.59 – 2.47 (m, 2H), 2.46 (s, 3H), 1.69 (s, 3H). ^{13}C NMR (101 MHz, $CDCl_3$) δ 175.32, 170.72, 167.57, 160.39, 160.00, 154.33, 151.31,

139.49, 134.30, 133.21, 132.14, 130.75, 130.03, 129.33, 116.68, 113.82, 52.93, 36.12, 35.58, 33.42, 14.21, 13.15, 11.02.

h) Isotopically labeled dialkoxydiphenylsilane (DADPS) tag :

(1) Cleavable biotin azide light tag (**L6**):

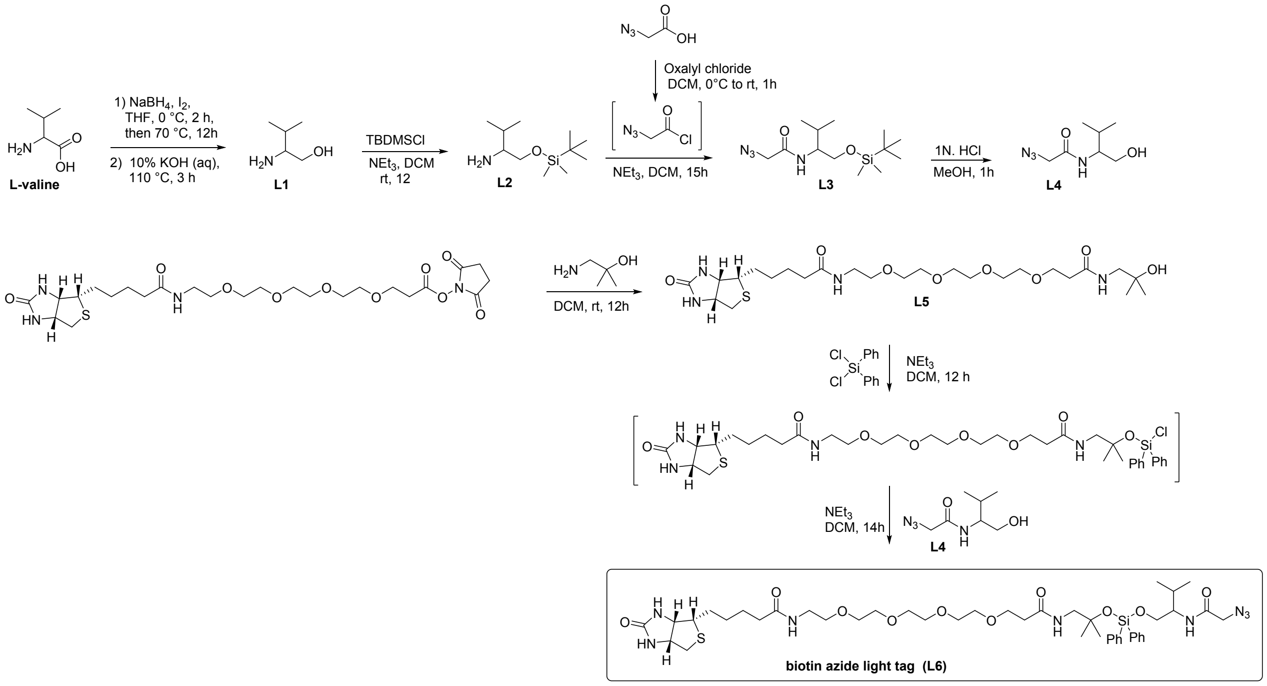

(2) Cleavable biotin azide heavy tag (**H6**):

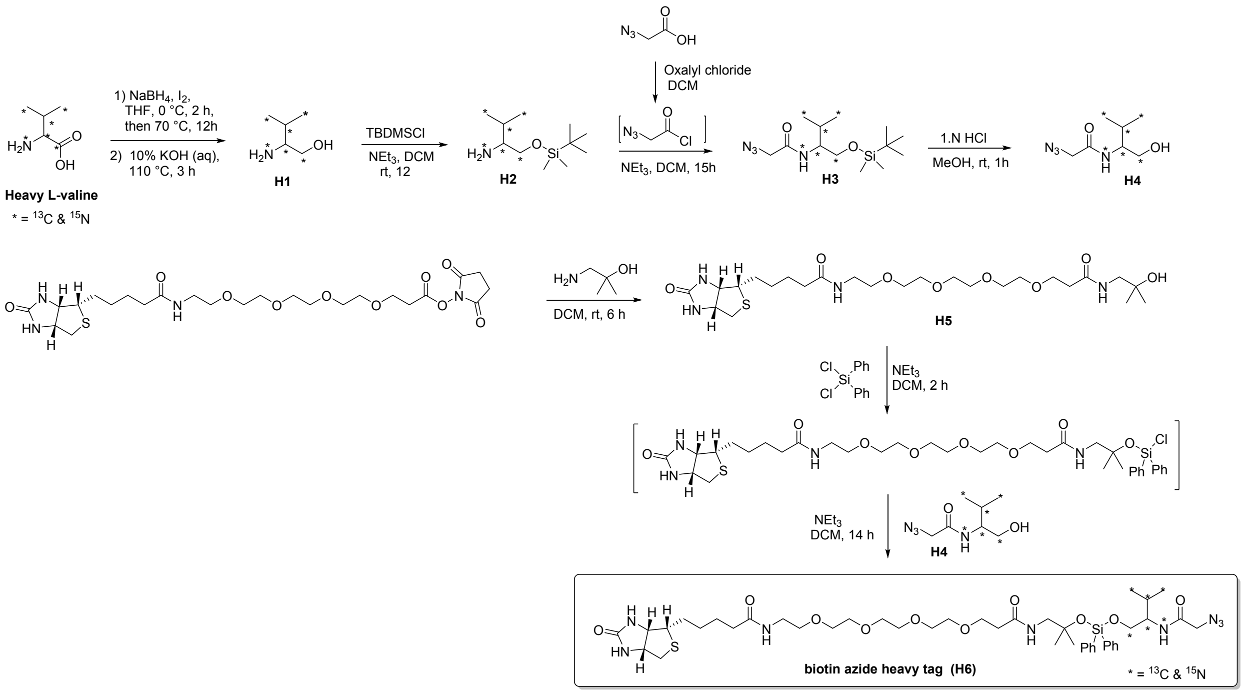

i) General procedure for synthesis of the cleavable biotin azide light valine and heavy valine tag

Step 1: Synthesis of 2-amino-3-methylbutan-1-ol **L1** and **H1** was synthesized with light L-valine and heavy L-valine according to previously published procedures.[6]

Step 2: Synthesis of **L2** and **H2** intermediate was carried out according to reported procedure.[7]

Step 3: General procedure for synthesis of **L3** and **H3**: A solution of 1-((tert-butyldimethylsilyl)oxy)-3-methylbutan-2-amine **L2** (214 mg, 0.99 mmol) and triethylamine (276 uL, 1.98 mmol) in dichloromethane (3 mL) was added to the freshly prepared solution of azidoacetyl chloride[8] (118 mg, 0.99 mmol) in dichloromethane (3 mL) at 0 °C, over 10 min and the resultant mixture was stirred at room temperature for 15 h. After completion monitored by TLC ($KMnO_4$ stains) the resulting reaction mixture was diluted with water and extracted in dichloromethane (3 × 30 mL). The combined organic layer was dried over Na_2SO_4, filtered, and concentrated. The resulting oil was purified by column chromatography (hexanes/ ethyl acetate/ triethylamine, 84:15:1) to afford **(L3)**.

2-Azido-N-(1-((tert-butyldimethylsilyl)oxy)-3-methylbutan-2-yl)acetamide (L3)

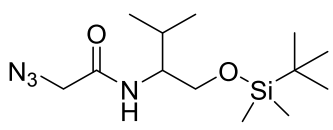

L3

^{1}H NMR (400 MHz, $CDCl_3$) δ 6.51 (d, *J* = 9.3 Hz, 1H), 4.01 – 3.88 (m, 2H), 3.74 – 3.61 (m, 2H), 3.51 (dd, *J* = 10.0, 3.7 Hz, 1H), 1.94 – 1.80 (m, 1H), 1.62 – 1.56 (m, 1H), 0.89 (d, *J* = 6.7 Hz, 3H), 0.87 (d, *J* = 6.8 Hz, 3H), 0.85 (s, 9H), -0.00 (s, 6H), (163 mg, 55 % yield) as a colorless oil.

2-Azido-N-(1-((tert-butyldimethylsilyl)oxy)-3-methylbutan-2-yl)acetamide (H3)

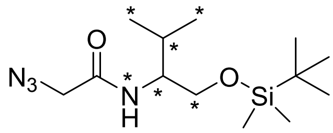

H3 (* = ^{13}C & ^{15}N)

^{1}H NMR (400 MHz, $CDCl_3$) δ 6.69 – 6.43 (m, 1H), 4.03 – 3.96 (m, 2H), 3.96 – 3.33 (m, 3H), 2.19 – 1.62 (m, 1H), 1.18 – 1.00 (m, 3H), 0.89 (s, 9H), 0.85 – 0.72 (m, 3H), 0.05 (s, 6H). ^{13}C NMR (101 MHz, $CDCl_3$) δ 165.91, 63.50 (d, *J*cc = 40.4 Hz), 55.58 (ddd, *J*cc = 40.7, 37.0, 9.7 Hz), 52.94, 28.97 (q, *J*cc = 35.6 Hz), 25.75, 19.25 (dd, *J*cc = 41.3, 34.9 Hz), 18.17, 5.54, 5.60. (502 mg, 52% yield) as a colorless oil.

Step 4: General procedure of synthesis of **L4** and **H4**: To a solution of **L3** (400 mg, 1.33 mmol) in methanol (5 mL) was added hydrochloric acid solution (1 M, 5 mL) at 0 ºC. The reaction mixture was stirred at room temperature until completion (40 min, monitored by TLC, $KMnO_4$ stain). The methanol solution was evaporated and the resulting residue was diluted with water (10 mL) and extracted with ethyl acetate (3 × 20 mL) the organic layer was washed with saturated sodium bicarbonate, brine, and dried over Na_2SO_4. Concentration under vacuum gave the product **L4**.

2-Azido-N-(1-hydroxy-3-methylbutan-2-yl)acetamide (L4)

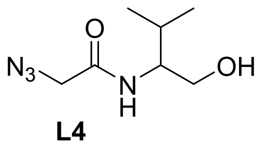

L4

^{1}H NMR (400 MHz, $CDCl_3$) δ 6.58 (d, J = 8.8 Hz, 1H), 4.03 (s, 2H), 3.83 – 3.56 (m, 3H), 3.13 (s, 1H), 2.00 – 1.85 (m, 1H), 0.97 (d, J = 6.8 Hz, 3H), 0.94 (d, J = 6.8 Hz, 3H). (218 mg, 88%) as a colorless oil.

2-Azido-N-(1-hydroxy-3-methylbutan-2-yl)acetamide (H4)

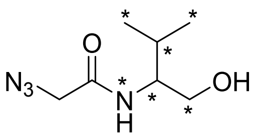

H4 (* = ^{13}C & ^{15}N)

^{1}H NMR (400 MHz, $CDCl_3$) δ 6.68 – 6.29 (m, 1H), 4.05 (s, 2H), 3.99 – 3.41 (m, 3H), 2.44 (s, 1H), 2.20 – 1.73 (m, 1H), 1.21 – 1.05 (m, 3H), 0.89 – 0.75 (m, 3H). ^{13}C NMR (101 MHz, $CDCl_3$) δ 167.52, 63.62 (d, Jcc = 38.9 Hz), 57.12 (ddd, Jcc = 39.0, 36.0, 9.3 Hz), 52.77, 28.91 (q, cc$J$ = 35.5 Hz), 19.47 (d, Jcc = 35.3 Hz), 18.72 (d, Jcc = 36.0 Hz). (167 mg, 82 % yield) as a colorless oil.

Step 5: **General procedure for the synthesis of cleavable biotin azide light valine tag (L6) and heavy valine tag (H6):[9]**

***N*-(10-Azido-7-isopropyl-2,2-dimethyl-9-oxo-4,4-diphenyl-3,5-dioxa-8-aza-4-siladecyl)-1-(5-((3aS,4S,6aR)-2-oxohexahydro-1H-thieno[3,4-d]imidazol-4-yl)pentanamido)-3,6,9,12-tetraoxapentadecan-15-amide (L6)**

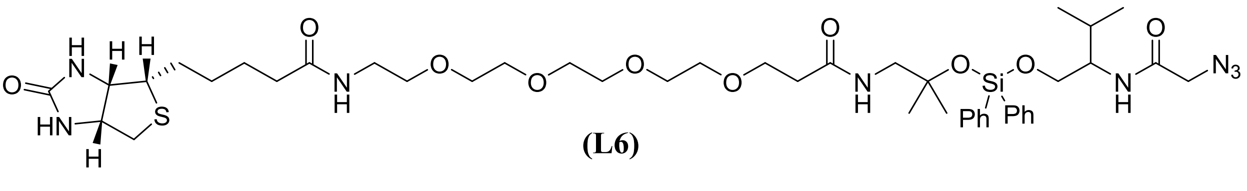

(L6)

To a stirred solution of biotin–PEG_4–alcohol[10] **L5** (20 mg, 35 µmol) in anhydrous dichloromethane (1 mL), triethylamine (75 µL, 0.533 mmol) and dichlorodiphenylsilane (38 µL, 178 mmol) were added in sequence at 0 °C and the resulting solution was stirred for 2 h at 24 °C. A solution of 2-azido-N-(1-hydroxy-3-methylbutan-2-yl)acetamide **L4** (66 mg, 0.355 mmol) in anhydrous dichloromethane (1 mL) was added to the stirred solution. The resulting solution was stirred for an additional 14 h at 24 °C. The product mixture was diluted with dichloromethane (10 mL) and saturated aqueous sodium bicarbonate solution (5 mL). The resulting mixture was extracted with dichloromethane (3 × 20 mL), and the combined organic layers were dried over Na_2SO_4 and filtered. The filtrate was concentrated by rotary evaporation and the residue obtained was purified with a Biotage® SNAP Cartridge KP-Sil 10 g with a linear gradient of 100 % dichoromethane rising to 8 : 0.8 : 1 dichloromethane : methanol : triethylamine, to afford the cleavable biotin azide light tag (**L6**) as a clear colorless sticky material (18 mg, 54 %). ^{1}H NMR (600 MHz, $CDCl_3$) δ 7.53 (d, *J* = 8.1 Hz, 4H), 7.42 – 7.35 (m, 2H), 7.32 (d, *J* = 7.4 Hz, 4H), 6.73 (d, *J* = 5.6 Hz, 1H), 6.51 (d, *J* = 9.9 Hz, 2H), 6.29 (s, 1H), 5.35 (s, 1H), 4.38 (dd, *J* = 7.8, 4.9 Hz, 1H), 4.21 (dd, *J* = 7.9, 4.6 Hz, 1H), 3.85 (d, *J* = 16.3 Hz, 1H), 3.80 – 3.74 (m, 2H), 3.73 – 3.67 (m, 2H), 3.64 (t, *J* = 6.1 Hz, 2H), 3.53 – 3.52 (m, 6H), 3.51 – 3.46 (m, 6H), 3.36 – 3.32 (m, 2H), 3.29 – 3.22 (m, 2H), 3.05 (td, *J* = 7.4, 4.5 Hz, 1H), 2.80 (dd, *J* = 12.8, 4.9 Hz, 1H), 2.64 (d, *J* = 12.8 Hz, 1H), 2.35 (t, *J* = 6.1 Hz, 2H), 2.18 – 2.09 (m, 4H), 1.93 – 1.88 (m, 1H), 1.67 – 1.55 (m, 4H), 1.40 – 1.33 (m, 2H), 1.18 (d, *J* = 3.9 Hz, 3H), 0.83 (d, *J* = 6.8 Hz, 3H). ^{13}C NMR (151 MHz, $CDCl_3$) δ 172.28, 170.35, 165.38, 162.74, 133.77, 133.75, 133.50, 133.37, 132.70, 132.59, 129.42, 129.39, 126.93, 126.63, 74.78, 69.39, 69.35, 69.29, 69.17, 69.00, 68.91, 66.28, 61.90, 60.69, 59.09, 54.73, 54.48, 51.45, 49.31, 39.45, 38.09, 35.96, 34.81, 27.74, 27.10, 27.01, 26.57, 26.55, 24.51, 18.43, 17.82. LCMS *m/z* (M+H) 929.

***N*-(10-Azido-7-isopropyl-2,2-dimethyl-9-oxo-4,4-diphenyl-3,5-dioxa-8-aza-4-siladecyl)-1-(5-((3aS,4S,6aR)-2-oxohexahydro-1H-thieno[3,4-d]imidazol-4-yl)pentanamido)-3,6,9,12-tetraoxapentadecan-15-amide (H6)**

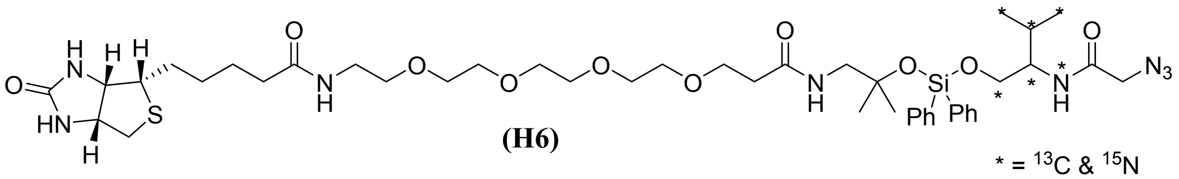

(H6)

* = ^{13}C & ^{15}N

^{1}H NMR (400 MHz, $CDCl_3$) δ 7.63 – 7.58 (m, 4H), 7.48 – 7.41 (m, 2H), 7.38 (dd, *J* = 7.8, 6.4 Hz, 4H), 6.91 (t, *J* = 5.6 Hz, 1H), 6.75 – 6.45 (m, 3H), 5.67 (s, 1H), 4.46 (dd, *J* = 7.8, 4.8 Hz, 1H), 4.28 (ddd, *J* = 7.8, 4.6, 1.4 Hz, 1H), 4.07 – 3.76 (m, 4H), 3.71 (t, *J* = 6.0 Hz, 2H), 3.64 – 3.52 (m, 15H), 3.46 – 3.38 (m, 2H), 3.33 (dd, *J* = 7.4, 6.2 Hz, 2H), 3.12 (td, *J* = 7.3, 4.5 Hz, 1H), 2.87 (dd, *J* = 12.8, 4.9 Hz, 1H), 2.72 (d, *J* = 12.8 Hz, 1H), 2.54 (s, 1H), 2.43 (t, *J* = 6.0 Hz, 2H), 2.21 (t, *J* = 7.5 Hz, 2H), 1.78 – 1.61 (m, 4H), 1.43 (q, *J* = 7.5 Hz, 2H), 1.26 (s, 6H), 1.06 (t, *J* = 5.5 Hz, 3H), 0.75 (t, *J* = 5.4 Hz, 3H). ^{13}C NMR (101 MHz, $CDCl_3$) δ 173.41, 171.42, 166.53, 166.37, 164.02, 134.82, 134.67, 133.76, 133.65, 130.48, 130.45, 127.99, 127.68, 75.84, 70.45, 70.40, 70.35, 70.23, 70.05, 69.99, 67.34, 62.96 (d, *J*cc = 40.7 Hz), 61.75, 60.18, 55.76 (ddd, *J*cc = 40.8, 36.6, 9.6 Hz), 52.52, 52.45, 50.36, 40.52, 39.15, 37.02, 35.93, 28.77 (q, *J*cc = 35.7 Hz), 27.62, 25.63. 19.48 (d, *J*cc = 35.1 Hz), 18.88 (d, *J*cc = 35.4 Hz). LCMS *m/z* (M+H) 935.

Characterization data for diazirine probes

***N*-(2-(3-(But-3-yn-1-yl)-3H-diazirin-3-yl)ethyl)benzamide (LD-C)**

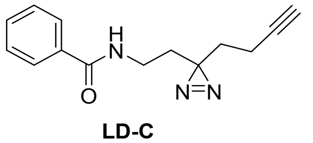

LD-C

Synthesized according to general procedure 1. Purified by PTLC (hexanes/EtOAc, 9:1) to afford **LD-C** as a colorless liquid (12 mg, 74 %). ^{1}H NMR (400 MHz, $CDCl_3$) δ 7.83 – 7.76 (m, 2H), 7.55 – 7.48 (m, 1H), 7.48 – 7.39 (m, 2H), 6.38 (s, 1H), 3.32 (td, *J* = 6.6, 5.8 Hz, 2H), 2.04 (td, *J* = 7.2, 2.4 Hz, 2H), 1.99 (t, *J* = 2.6 Hz, 1H), 1.82 (t, *J* =

6.7 Hz, 2H), 1.69 (t, *J* = 7.2 Hz, 2H). ^{13}C NMR (101 MHz, $CDCl_3$) δ 167.58, 134.40, 131.59, 128.63, 126.92, 82.70, 69.46, 34.91, 32.50, 32.13, 26.95, 13.21. HRMS (ESI-TOF) *calcd for* $C_{14}H_{16}N_3O$, 242.1288 (M+H$^+$), *found* 242.1292.

N-Phenyl-2-(prop-2-yn-1-yloxy)-4-(3-(trifluoromethyl)-3H-diazirin-3-yl)benzamide (Ar-C)

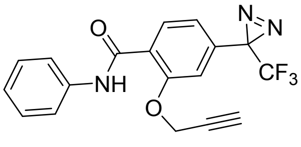

Ar-C

Synthesized according to general procedure 2. Purified by PTLC (hexanes/EtOAc, 8:2) to afford **Ar-C** as a white solid (10 mg, 82 %).^{1}H NMR (600 MHz, $CDCl_3$) δ 9.60 (s, 1H), 8.32 (d, *J* = 8.3 Hz, 1H), 7.68 (dd, *J* = 8.3, 1.1 Hz, 2H), 7.42 – 7.35 (m, 2H), 7.15 (dd, *J* = 7.4, 1.1 Hz, 1H), 7.02 – 6.92 (m, 1H), 6.85 (d, *J* = 1.7 Hz, 1H), 4.91 (d, *J* = 2.4 Hz, 2H), 2.73 (t, *J* = 2.4, 1H). ^{13}C NMR (151 MHz, $CDCl_3$) δ 161.63, 155.17, 138.04, 134.04, 133.39, 129.05, 124.53, 123.80, 120.38 (d, *J* = 13.2 Hz), 111.05, 77.89, 76.24, 57.65, 28.35 (q, *J* = 40.4 Hz). HRMS (ESI-TOF) *calcd for* $C_{18}H_{13}F_3N_3O_2$, 360.0955 (M+H$^+$), *found* 360.0962.

N-(2-(3-Methyl-3H-diazirin-3-yl)ethyl)-N-(prop-2-yn-1-yl)benzamide (BD-C)

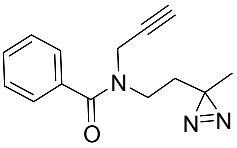

BD-C

Synthesized according to general procedure 1. Purified by PTLC (hexanes/EtOAc, 7:3) to afford BD-C as a light-yellow liquid (8 mg, 68 %). ^{1}H NMR (400 MHz, DMSO-d_6 at 100°C) δ 7.48 – 7.42 (m, 3H), 7.42 – 7.36 (m, 2H), 4.09 (d, *J* = 2.5 Hz, 2H), 3.49 – 3.35 (m, 2H), 3.08 (t, *J* = 2.5 Hz, 1H), 1.71 – 1.59 (m, 2H), 0.96 (s, 3H). ^{13}C NMR (151 MHz, $CDCl_3$) δ 168.99, 133.15, 127.71, 126.25, 124.43, 76.12, 70.93, 41.24,

38.50, 37.24, 30.04, 22.15, 17.17. Note: rotomeric isomers observed. HRMS (ESI-TOF) *calcd for* $C_{14}H_{16}N_3O$, 242.1288 (M+H$^+$), *found* 242.1297.

(3-(1,1-Difluoroprop-2-yn-1-yl)-3H-diazirin-3-yl)methyl phenylcarbamate (DF-C)

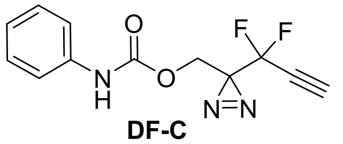

DF-C

Synthesized according to general procedure 4. Purified by PTLC (hexanes/EtOAc, 8:2) to afford **DF-C** as a sticky colorless liquid (6 mg, 56 %).^{1}H NMR (600 MHz, $CDCl_3$) δ 7.30 (d, *J* = 7.9 Hz, 2H), 7.25 (t, *J* = 8.0 Hz, 2H), 7.02 (t, *J* = 7.1 Hz, 1H), 6.64 (s, 1H), 4.30 (s, 2H), 2.93 (q, *J* = 4.8 Hz, 1H). ^{13}C NMR (151 MHz, $CDCl_3$) δ 152.05, 137.4, 129.65, 127.06, 124.03, 118.75, 110.08 (t, *J* = 235.5 Hz), 80.51 (t, *J* = 6.5 Hz), 72.70 (t, *J* = 40.4 Hz), 60.57, 29.73 (t, *J* = 36.0 Hz).^{19}F NMR (565 MHz, $CDCl_3$) δ -86.53. HRMS (ESI-TOF) *calcd for* $C_{12}H_8F_3N_2O_2$, 264.0590 (M-H$^+$), *found* 264.0587

N-((3H-Diazirin-3-yl)methyl)-N-(prop-2-yn-1-yl)benzamide (Tm-C)

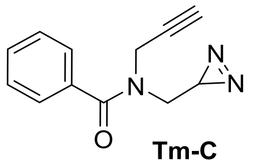

Tm-C

Synthesized according to general procedure 1. Purified by PTLC (hexanes/EtOAc, 7:3) to afford Tm-C as a light brown sticky liquid (8 mg, 64 %).^{1}H NMR (600 MHz, $CDCl_3$)δ 7.55 – 7.38 (m, 10H), 4.46 (s, 1H), 4.08 (s, 3H), 3.54 (s, 4H), 2.35 (s, 2H), 1.65 (s, 1H), 1.28 (s, 1H). ^{13}C NMR (151 MHz, $CDCl_3$) δ 171.58, 134.69, 130.39, 128.67, 127.06, 78.02, 73.53, 44.98, 40.27, 19.47. Note: rotomeric isomers observed. HRMS (ESI-TOF) *calcd for* $C_{12}H_{12}N_3O$, 214.0975 (M+H$^+$), found 214.0980.

1-Benzhydryl-N-(2-(3-(but-3-yn-1-yl)-3H-diazirin-3-yl)ethyl)azetidine-3-carboxamide (LD-F)

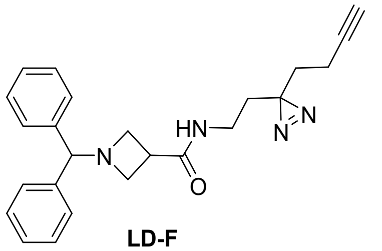

LD-F

Synthesized according to general procedure 1. Purified by PTLC (Hexanes/EtOAc, 7:3) to afford **LD-F** as a colorless sticky liquid (8 mg, 64%). ^{1}H NMR (400 MHz, $CDCl_3$) δ 7.46 – 7.39 (m, 4H), 7.32 – 7.26 (m, 4H), 7.23 – 7.17 (m, 2H), 6.48 (s, 1H), 4.45 (s, 1H), 3.44 – 3.29 (m, 4H), 3.15 (q, J = 6.4 Hz, 2H), 3.11 – 3.00 (m, 1H), 2.03 (td, J = 7.3, 2.6 Hz, 2H), 1.98 (t, J = 2.6 Hz, 1H), 1.75 (t, J = 6.6 Hz, 2H), 1.66 (t, J = 7.3 Hz, 2H). ^{13}C NMR (101 MHz, $CDCl_3$) δ 173.40, 141.51, 128.55, 127.47, 127.29, 82.65, 77.63, 77.28, 69.50, 56.41, 35.83, 34.18, 32.42, 32.27, 26.91, 13.26. HRMS (ESI-TOF) *calcd for* $C_{24}H_{27}N_4O$, 387.2180 (M+H$^+$), *found* 387.2186.

N-(1-Benzhydrylazetidin-3-yl)-2-(prop-2-yn-1-yloxy)-4-(3-(trifluoromethyl)-3H-diazirin-3-yl)benzamide (Ar-F)

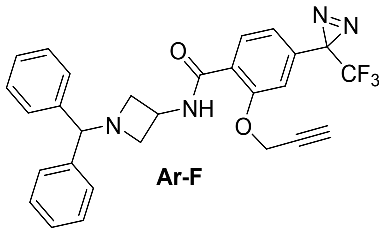

Ar-F

Synthesized according to general procedure 1. Purified by PTLC (hexanes/EtOAc, 6:4) to afford **Ar-F** as a white solid (8 mg, 78%). ^{1}H NMR (600 MHz, $CDCl_3$) δ 8.18 (dd, J = 8.2, 1.1 Hz, 1H), 8.03 (d, J = 7.1 Hz, 1H), 7.44 – 7.38 (m, 4H), 7.30 – 7.24 (m, 5H), 7.22 – 7.16 (m, 2H), 6.94 – 6.88 (m, 1H), 6.81 (s, 1H), 4.85 (dd, J = 2.5, 1.1 Hz, 2H), 4.70 (q, J = 6.6 Hz, 1H), 4.37 (s, 1H), 3.63 (t, J = 7.5 Hz, 2H), 2.98 (dd, J = 8.1, 5.9 Hz, 2H), 2.68 (d, J = 1.0 Hz, 1H). ^{13}C NMR (151 MHz, $CDCl_3$) δ 163.27, 155.42, 141.87, 133.68, 133.09, 128.46, 127.40, 127.21, 123.28, 121.80 (d, J = 274.7 Hz), 110.96, 78.14, 77.59, 76.46, 60.92, 57.46, 40.57, 28.32 (q, J = 40.8 Hz). HRMS (ESI-TOF) *calcd for* $C_{28}H_{24}F_3N_4O_2$, 505.1846 (M+H$^+$), *found* 505.1849.

1-Benzhydryl-N-(2-(3-methyl-3H-diazirin-3-yl)ethyl)-N-(prop-2-yn-1-yl)azetidine-3-carboxamide (BD-F)

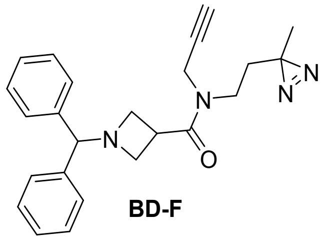

BD-F

Synthesized according to general procedure 1. Purified by PTLC (hexanes/EtOAc, 7:3) to afford **BD-F** as a colorless sticky liquid (11 mg, 65 %). ^{1}H NMR (600 MHz, $CDCl_3$) δ 7.40 (dt, *J* = 8.1, 1.4 Hz, 7H), 7.31 – 7.23 (m, 7H), 7.20 – 7.16 (m, 3H), 4.40 (d, *J* = 5.8 Hz, 2H), 4.17 (d, *J* = 2.6 Hz, 1H), 3.84 (d, *J* = 2.5 Hz, 2H), 3.53 (q, *J* = 7.7 Hz, 1H), 3.51 – 3.40 (m, 6H), 3.34 – 3.24 (m, 3H), 3.23 – 3.14 (m, 1H), 2.23 (t, *J* = 2.4 Hz, 1H), 2.20 (t, *J* = 2.5 Hz, 1H), 1.63 – 1.52 (m, 3H), 1.05 (s, 3H), 1.02 (s, 2H). ^{13}C NMR (151 MHz, $CDCl_3$) δ 171.96, 171.50, 141.74, 141.65, 128.50, 128.47, 127.49, 127.22, 127.17, 78.50, 78.05, 77.72, 73.09, 72.33, 55.82, 55.73, 41.66, 41.64, 37.21, 34.20, 33.69, 32.60, 32.53, 32.14, 24.32, 23.75, 19.79, 19.55. Note: rotomeric isomers observed. HRMS (ESI-TOF) *calcd* for $C_{24}H_{27}N_4O$, 387.2180 (M+H$^+$), *found* 387.2185.

(3-(1,1-Difluoroprop-2-yn-1-yl)-3H-diazirin-3-yl)methyl(1-benzhydrylazetidin-3-yl)carbamate (DF-F)

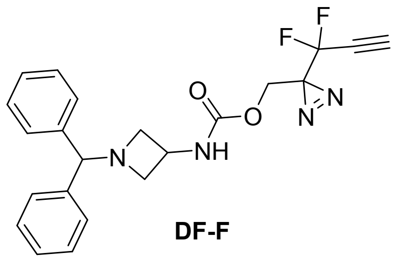

DF-F

Synthesized according to general procedure 4. Purified by PTLC (hexanes/EtOAc, 7:3) to afford **DF-F** as a colorless sticky liquid (7 mg, 58 %). ^{1}H NMR (600 MHz, $CDCl_3$) ^{1}H NMR (600 MHz, $CDCl_3$) δ 7.38 (d, *J* = 7.6 Hz, 4H), 7.27 (d, *J* = 7.6 Hz, 4H), 7.19 (d, *J* = 7.3 Hz, 2H), 5.21 (d, *J* = 8.4 Hz, 1H), 4.30 (s, 2H), 4.24 (s, 3H), 3.53 – 3.47 (m,

2H), 2.96 (t, *J* = 5.1 Hz, 1H), 2.89 – 2.86 (m, 1H). ^{13}C NMR (151 MHz, $CDCl_3$) δ 154.22, 141.69, 128.51, 127.37, 110.07 (t, *J* = 235.8 Hz), 80.40 (t, *J* = 6.6 Hz), 78.05, 72.82 (d, *J* = 40.4 Hz), 60.82, 60.52, 41.80, 29.60 (d, *J* = 35.4 Hz). ^{19}F NMR (376 MHz, $CDCl_3$) δ -86.58, -86.60. HRMS (ESI-TOF) *calcd for* $C_{22}H_{21}F_2N_4O_2$, 411.1627 (M+H$^+$), *found* 411.1632.

***N*-((3H-Diazirin-3-yl)methyl)-1-benzhydryl-*N*-(prop-2-yn-1-yl)azetidine-3-carboxamide (Tm-F)**

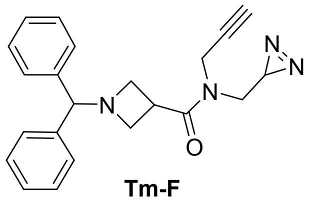

Tm-F

Synthesized according to general procedure 1. Purified by PTLC (hexanes/EtOAc, 7:3) to afford **Tm-F** as a light brown liquid (9 mg, 64 %). ^1H NMR (400 MHz, $CDCl_3$) δ 7.40 (dt, *J* = 8.0, 1.7 Hz, 6H), 7.32 – 7.23 (m, 6H), 7.23 – 7.13 (m, 3H), 4.39 (d, *J* = 2.0 Hz, 1H), 4.25 (d, *J* = 2.5 Hz, 1H), 3.90 (d, *J* = 2.5 Hz, 2H), 3.58 – 3.37 (m, 5H), 3.36 (d, *J* = 3.9 Hz, 2H), 3.33 – 3.19 (m, 4H), 2.27 (t, *J* = 2.4 Hz, 1H), 2.22 (t, *J* = 2.5 Hz, 1H), 1.11 (t, *J* = 3.8 Hz, 1H), 1.03 (t, *J* = 3.6 Hz, 1H). ^{13}C NMR (101 MHz, $CDCl_3$) δ 172.31, 171.73, 141.70, 141.64, 128.52, 127.51, 127.24, 78.08, 77.73, 77.48, 73.48, 55.76, 45.80, 45.52, 37.80, 34.93, 32.34, 32.25, 19.51, 19.45. Note: rotomeric isomers observed. HRMS (ESI-TOF) *calcd for* $C_{22}H_{23}N_4O$, 359.1866 (M+H$^+$), *found* 359.1874.

***N^1^*-(2-(3-(But-3-yn-1-yl)-3H-diazirin-3-yl)ethyl)-N^4-((5R,7R,8R,9S)-8-methoxy-9-methyl-16-oxo-6,7,8,9,15,16-hexahydro-5H,14H-17-oxa-4b,9a,15-triaza-5,9 methanodibenzo[b,h]cyclonona[jkl]cyclopenta[e]-as-indacen-7-yl)-N^4-methylsuccinamide (LD-St)**

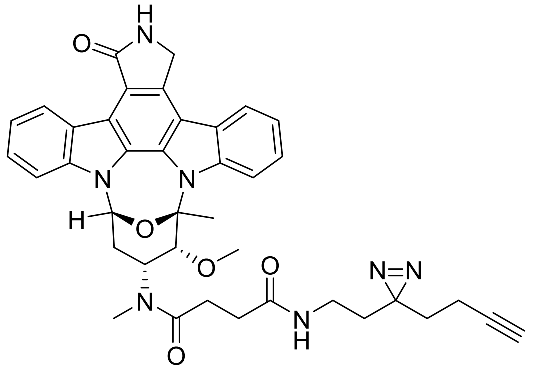

LD-St

Synthesized according to general procedure 1. Purified by PTLC (DCM/methanol, 19:1) to afford **LD-St** as a colorless sticky liquid (7.4 mg, 64 %). ^{1}H NMR (600 MHz, $CDCl_3$) δ 9.44 (dd, *J* = 8.0, 2.5 Hz, 1H), 7.89 (dd, *J* = 7.8, 2.5 Hz, 1H), 7.72 (dd, *J* = 8.5, 2.5 Hz, 1H), 7.49 (td, *J* = 7.4, 2.4 Hz, 1H), 7.47 – 7.42 (m, 1H), 7.38 (td, *J* = 7.6, 2.5 Hz, 1H), 7.33 (td, *J* = 7.5, 2.7 Hz, 1H), 7.28 – 7.22 (m, 2H), 6.69 (q, *J* = 6.0, 4.3 Hz, 2H), 6.20 – 6.11 (m, 1H), 5.24 – 5.14 (m, 1H), 5.01 (s, 2H), 3.99 (d, *J* = 2.7 Hz, 1H), 3.14 – 3.18 (m, 2H), 2.86 (s, 3H), 2.73 – 2.70 (m, 1H), 2.68 – 2.66 (m, 1H), 2.61 – 2.54 (m, 4H), 2.49 (s, 3H), 2.46 (s, 3H), 2.04 – 2.06 (m, 2H), 1.73 – 1.67 (m, 3H). ^{13}C NMR (151 MHz, $CDCl_3$) δ 173.23, 172.83, 172.20, 138.63, 136.53, 132.43, 130.44, 126.73, 126.22, 125.48, 124.94, 124.65, 123.58, 121.43, 120.49, 120.12, 119.08, 116.30, 114.52, 112.38, 107.67, 94.60, 84.73, 82.71, 82.48, 69.46, 60.48, 48.71, 45.91, 34.42, 32.58, 32.12, 31.16, 31.12, 29.42, 29.19, 28.01, 26.93, 13.25. HRMS (ESI-TOF) *calcd for* $C_{39}H_{40}N_7O_5$, 686.3086 (M+H$^+$), *found* 686.3085.

***N*-(3-(((5R,7R,8R,9S)-8-Methoxy-9-methyl-16-oxo-6,7,8,9,15,16-hexahydro-5H,14H-17-oxa-4b,9a,15-triaza-5,9-methanodibenzo[b,h]cyclonona[jkl]cyclopenta[e]-as-indacen-7-yl)(methyl)amino)-3-oxopropyl)-2-(prop-2-yn-1-yloxy)-4-(3-(trifluoromethyl)-3H-diazirin-3-yl)benzamide (Ar-St)**

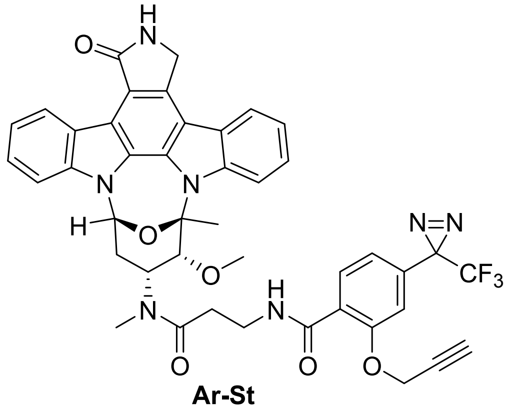

Ar-St

Synthesized according to general procedure 2. Purified by PTLC (DCM/methanol, 19:1) to afford **Ar-St** as an off-white solid (7 mg, 64 %). ^{1}H NMR (600 MHz, $CDCl_3$) δ 9.46 (d, *J* = 7.8 Hz, 1H), 8.42 (t, *J* = 6.1 Hz, 1H), 8.21 (d, *J* = 8.2 Hz, 1H), 7.95 – 7.90 (m, 1H), 7.76 – 7.71 (m, 1H), 7.52 – 7.46 (m, 1H), 7.47 – 7.42 (m, 1H), 7.41 – 7.32 (m, 2H), 7.26 – 7.20 (m, 1H), 6.97 (d, *J* = 1.7 Hz, 1H), 6.94 – 6.89 (m, 1H), 6.72 (dd, *J* = 7.7, 6.3 Hz, 1H), 6.50 (s, 1H), 5.26 – 5.22 (m, 1H), 5.03 – 4.99 (m, 2H), 4.97 (dd, *J* = 8.8, 2.4 Hz, 3H), 4.01 (d, *J* = 2.2 Hz, 1H), 3.85 – 3.76 (m, 2H), 2.83 (s, 3H), 2.71 (t, *J* = 2.4 Hz, 1H), 2.69 – 2.63 (m, 2H), 2.61 – 2.56 (m, 2H), 2.50 (s, 3H), 2.46 (s, 3H). ^{13}C NMR (151 MHz, $CDCl_3$) δ 173.15, 172.53, 163.89, 155.37, 138.61, 136.58, 133.51, 132.92, 132.42, 130.51, 126.86, 126.30, 125.53, 124.96, 124.75, 123.74 (d, *J* = 26.6 Hz), 121.53, 120.58, 120.10 (d, *J* = 30.1 Hz), 119.20, 116.41, 114.58, 112.36, 111.42, 107.61, 94.61, 84.87, 82.53, 77.66, 77.24, 77.03, 76.82, 60.48, 57.02, 53.44, 48.53, 45.87, 35.45, 33.89, 31.21, 29.71, 29.13, 28.35 (q, *J* = 39.3 Hz). HRMS (ESI-TOF) *calcd for* $C_{43}H_{37}F_3N_7O_6$, 804.2752 (M+H$^+$), *found* 804.2753.

***N^1^*-((5R,7R,8R,9S)-8-Methoxy-9-methyl-16-oxo-6,7,8,9,15,16-hexahydro-5H,14H-17-oxa-4b,9a,15-triaza-5,9-methanodibenzo[b,h]cyclonona[jkl]cyclopenta[e]-as-indacen-7-yl)-N^1-methyl-N^4-(2-(3-methyl-3H-diazirin-3-yl)ethyl)-N^4-(prop-2-yn-1-yl)succinimide (BD-St)**

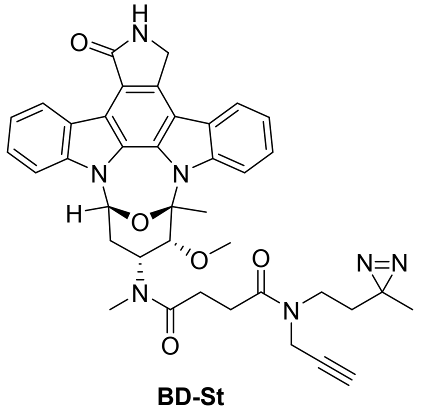

BD-St

Synthesized according to general procedure 1. Purified by PTLC (DCM/methanol, 19:1) to afford **BD-St** as a colorless sticky liquid (6 mg, 62 %). ^{1}H NMR (400 MHz, $CDCl_3$) δ 9.46 (d, *J* = 7.9 Hz, 2H), 7.90 (dd, *J* = 8.0, 1.3 Hz, 2H), 7.71 (d, *J* = 8.4 Hz, 2H), 7.54 – 7.41 (m, 4H), 7.41 – 7.30 (m, 4H), 7.24 (d, *J* = 8.1 Hz, 2H), 6.72 (s, 2H), 6.69 – 6.65 (m, 2H), 5.24 – 5.13 (m, 2H), 5.00 (s, 4H), 4.30 – 4.16 (m, 2H), 4.14 (d, *J* = 2.5 Hz, 2H), 4.00 (d, *J* = 2.3 Hz, 2H), 3.57 – 3.37 (m, 4H), 2.89 (d, *J* = 2.9 Hz, 6H), 2.85 – 2.75 (m, 3H), 2.73 – 2.67(m, 4H), 2.58 – 2.52 (m, 9H), 2.44 (d, *J* = 2.8 Hz, 6H), 2.32 (t, *J* = 2.4 Hz, 1H), 2.24 (t, *J* = 2.5 Hz, 1H), 1.80 – 1.73 (m, 3H), 1.62 (t, *J* = 7.6 Hz, 2H), 1.12 (s, 3H), 1.07 (s, 3H). ^{13}C NMR (101 MHz, $CDCl_3$) δ 173.25, 172.81, 172.76, 171.78, 171.24, 138.75, 138.71, 136.57, 132.47, 130.46, 126.79, 126.22, 125.47, 124.91, 124.88, 124.70, 123.63, 121.44, 120.45, 120.10, 119.16, 116.37, 114.54, 112.52, 112.45, 107.67, 94.67, 84.68, 82.52, 78.84, 78.39, 73.01, 72.20, 60.58, 60.53, 48.74, 48.69, 45.91, 42.16, 42.10, 37.76, 34.56, 33.55, 32.78, 31.21, 29.28, 29.23, 28.95, 28.35, 28.03, 24.35, 23.98, 19.87, 19.63. Note: rotomeric isomers observed. HRMS (ESI-TOF) *calcd for* $C_{39}H_{40}N_7O_5$, 686.3086 (M+H$^+$), *found* 686.3081.

(3-(1,1-Difluoroprop-2-yn-1-yl)-3H-diazirin-3-yl)methyl (3-(((5R,7R,8R,9S)-8-methoxy-9-methyl-16-oxo-6,7,8,9,15,16-hexahydro-5H,14H-17-oxa-4b,9a,15-triaza-5,9-methanodibenzo[b,h]cyclonona[jkl]cyclopenta[e]-as-indacen-7-yl)(methyl)amino)-3-oxopropyl)carbamate (DF-St)

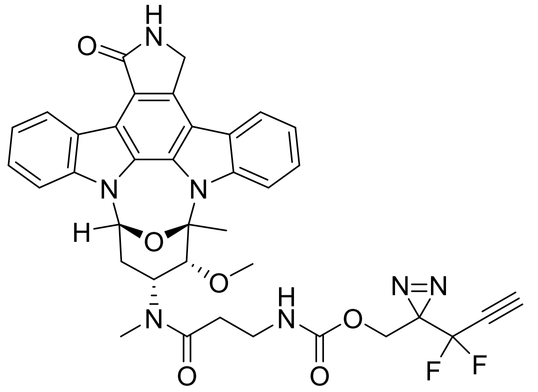

DF-St

Synthesized according to general procedure 4. Purified by PTLC (DCM/methanol, 19:1) to afford **DF-St** as a colorless sticky liquid (5 mg, 57 %). ^{1}H NMR (400 MHz, $CDCl_3$) δ 9.45 (d, *J* = 7.9 Hz, 1H), 7.97 – 7.87 (m, 1H), 7.74 (d, *J* = 8.5 Hz, 1H), 7.53 – 7.42 (m, 2H), 7.40 – 7.33 (m, 2H), 7.26 – 7.22 (m, 1H), 6.71 (t, *J* = 6.9 Hz, 1H), 6.59 (s, 1H), 5.70 (t, *J* = 6.3 Hz, 1H), 5.20 (t, *J* = 9.2 Hz, 1H), 5.04 – 4.99 (m, 2H), 4.29 (d, *J* = 2.8 Hz, 2H), 4.00 (d, *J* = 2.3 Hz, 1H), 3.53 (d, *J* = 6.0 Hz, 2H), 3.00 (t, *J* = 5.1 Hz, 1H), 2.81 (s, 3H), 2.62 – 2.52 (m, 4H), 2.49 (s, 3H), 2.47 (s, 3H). ^{13}C NMR (151 MHz, $CDCl_3$) δ 173.26, 173.23, 172.26, 155.15 (d, *J* = 3.8 Hz), 138.65, 136.59, 132.46, 130.52, 126.85, 126.28, 125.55, 125.52, 125.05, 124.70, 123.66, 121.50, 120.59, 120.56, 120.20, 119.17, 116.39, 114.60, 112.40, 110.21 (t, *J* = 235.8 Hz), 107.66 (d, *J* = 3.5 Hz), 94.60 (d, *J* = 3.9 Hz), 84.83 (d, *J* = 3.5 Hz), 82.47 (d, *J* = 3.5 Hz), 80.55 (d, *J* = 7.4 Hz), 77.05, 72.73 (t, *J* = 41.4 Hz), 60.50 (d, *J* = 3.9 Hz), 59.97, 48.61, 48.58, 45.93, 45.91, 36.83, 34.01, 31.19, 31.16, 29.73, 29.20, 29.18, 28.06, 28.03. ^{19}F NMR (376 MHz, $CDCl_3$) δ -86.53 (d, *J* = 5.4 Hz). HRMS (ESI-TOF) *calcd for* $C_{37}H_{34}F_2N_7O_6$, 710.2533 (M+H^{+}), *found* 710.2528.

***N^{1}*-((3H-Diazirin-3-yl)methyl)-N^{4}-((5R,7R,8R,9S)-8-methoxy-9-methyl-16-oxo-6,7,8,9,15,16-hexahydro-5H,14H-17-oxa-4b,9a,15-triaza-5,9-methanodibenzo[b,h]cyclonona[jkl]cyclopenta[e]-as-indacen-7-yl)-N^{4}-methyl-N^{1}-(prop-2-yn-1-yl)succinimide (Tm-St)**

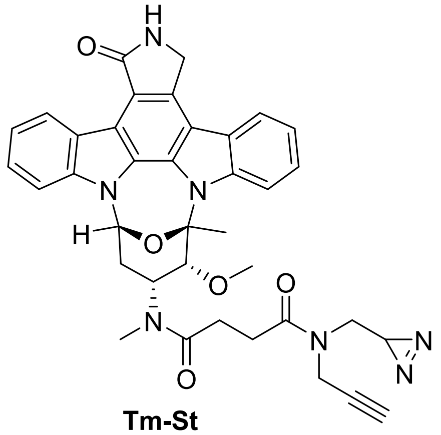

Tm-St

Synthesized according to general procedure 1. Purified by PTLC (DCM/methanol, 9.5:0.5) to afford **Tm-St** as a light yellow solid (6 mg, 54 %). ^{1}H NMR (400 MHz, $CDCl_3$) δ 9.46 (d, *J* = 7.9 Hz, 1H), 7.90 (dd, *J* = 7.7, 1.3 Hz, 1H), 7.74 – 7.68 (m, 1H), 7.53 – 7.30 (m, 6H), 7.24 (d, *J* = 8.1 Hz, 1H), 6.72 – 6.67 (m, 2H), 5.19 (td, *J* = 9.5, 8.3, 4.3 Hz, 1H), 5.00 (d, *J* = 1.8 Hz, 3H), 4.29 (dd, *J* = 9.2, 2.5 Hz, 1H), 4.21 (d, *J* = 2.5 Hz, 1H), 4.01 (dd, *J* = 6.9, 2.3 Hz, 1H), 3.55 – 3.48 (m, 1H), 3.53 – 3.42 (m, 2H), 2.89 (d, *J* = 1.9 Hz, 4H), 2.83 (d, *J* = 6.2 Hz, 1H), 2.71 (t, *J* = 3.0 Hz, 3H), 2.59 – 2.52 (m, 6H), 2.44 (d, *J* = 4.7 Hz, 4H), 2.37 (t, *J* = 2.4 Hz, 1H), 2.27 (t, *J* = 2.5 Hz, 1H), 1.25 (t, *J* = 3.8 Hz, 1H), 1.14 (t, *J* = 3.9 Hz, 1H). ^{13}C NMR (101 MHz, $CDCl_3$) δ 173.27, 172.70, 172.66, 172.15, 171.62, 138.74, 136.57, 132.49, 130.47, 126.81, 126.22, 125.49, 124.91, 124.70, 123.64, 121.45, 120.47, 120.12, 119.17, 116.37, 114.55, 112.49, 107.69, 94.68, 84.66, 82.53, 78.37, 77.81, 73.40, 72.66, 60.58, 48.77, 46.35, 46.06, 45.93, 38.31, 35.38, 31.24, 29.28, 29.24, 28.94, 28.88, 28.31, 28.10, 28.03, 19.66. Note: rotomeric isomers observed. HRMS (ESI-TOF) *calcd for* $C_{37}H_{36}N_7O_5$, 658.2773 (M+H$^+$), *found* 658.2772.

(S)-N-(2-(3-(But-3-yn-1-yl)-3H-diazirin-3-yl)ethyl)-3-(2-(4-(4-chlorophenyl)-2,3,9-trimethyl-6H-thieno[3,2-f][1,2,4]triazolo[4,3-a][1,4]diazepin-6-yl)acetamido)propenamide (LD-JQ)

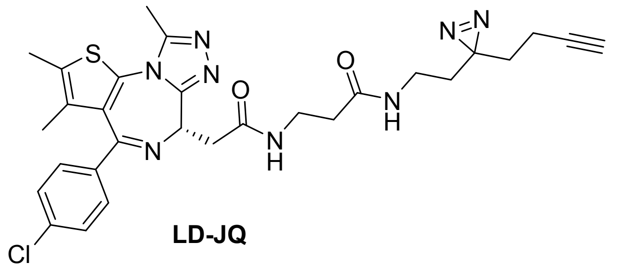

LD-JQ

Synthesized according to general procedure 1. Purified by PTLC (DCM/methanol, 9.6:0.4) to afford **LD-JQ** as a colorless sticky liquid (5 mg, 57 %). ^{1}H NMR (400 MHz, $CDCl_3$) δ 7.43 (d, *J* = 8.5 Hz, 2H), 7.34 (d, *J* = 8.7 Hz, 2H), 7.15 (t, *J* = 5.7 Hz, 1H), 4.70 (dd, *J* = 9.6, 5.4 Hz, 1H), 3.82 (dq, *J* = 13.0, 6.3, 5.8 Hz, 1H), 3.60 (dd, *J* = 14.8, 9.6 Hz, 1H), 3.36 – 3.28 (m, 2H), 3.14 – 3.05 (m, 1H), 2.84 (t, *J* = 7.8 Hz, 1H), 2.67 (s, 3H), 2.42 (s, 3H), 2.35 (q, *J* = 4.9 Hz, 2H), 2.07 – 2.02 (m, 2H), 2.00 – 1.90 (m, 4H), 1.73 – 1.67 (m, 6H). ^{13}C NMR (151 MHz, $CDCl_3$) δ 171.94, 170.65, 164.22, 155.72, 149.93, 136.95, 136.46, 132.03, 131.08, 130.90, 130.50, 129.88, 128.78, 82.75, 69.32, 54.46, 39.14, 37.04, 36.40, 34.63, 32.01, 31.69, 27.51, 14.40, 13.25, 13.13, 11.83. HRMS (ESI-TOF) *calcd for* $C_{29}H_{32}ClN_8O_2S$, 591.2052 (M+H$^+$), *found* 591.2053.

(S)-*N*-(2-(2-(4-(4-Chlorophenyl)-2,3,9-trimethyl-6H-thieno[3,2-f][1,2,4]triazolo[4,3-a][1,4]diazepin-6-yl)acetamido)ethyl)-2-(prop-2-yn-1-yloxy)-4-(3-(trifluoromethyl)-3H-diazirin-3-yl)benzamide

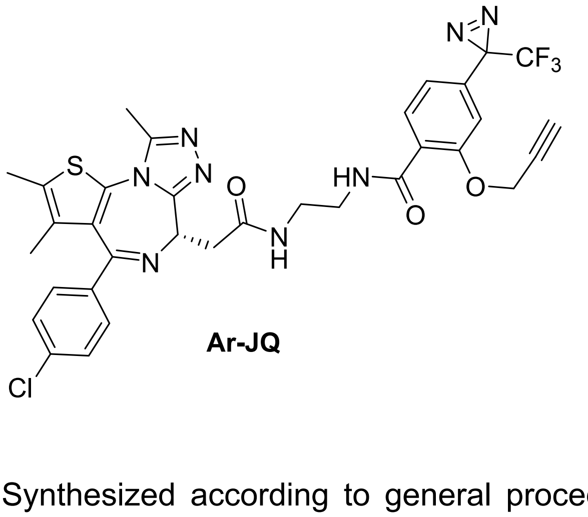

Ar-JQ

Synthesized according to general procedure 3. Purified by PTLC (DCM/methanol, 19:1) to afford **Ar-JQ** as an off white solid (8 mg, 65 %). ^{1}H NMR (400 MHz, $CDCl_3$) δ 8.15 (d, *J* = 8.1 Hz, 1H), 8.06 (s, 1H), 7.36 (dd, *J* = 8.5, 1.6 Hz, 2H), 7.32 – 7.27 (m, 2H), 7.03 (s, 1H), 6.89 – 6.87 (s, 2H), 4.90 (s, 2H), 4.62 – 4.60 (m, 1H), 3.68 – 3.64

(m, 1H), 3.57 – 3.53 (m, 4H), 3.39 – 3.36 (m, 1H), 2.62 (s, 4H), 2.40 (s, 3H), 1.63 (s, 3H).^{13}C NMR (151 MHz, $CDCl_3$) δ 171.29, 164.56, 164.06, 155.61, 155.47, 149.92, 136.89, 136.50, 133.45, 132.91, 132.12, 130.91 (d, *J* = 10.0 Hz), 130.40, 129.77, 128.76, 123.61, 119.82, 111.28, 77.47, 57.06, 54.40, 40.39, 39.33, 29.71, 28.36 (q, *J* = 40.4 Hz), 14.34, 13.09, 11.79. ^{19}F NMR (376 MHz, $CDCl_3$) δ -64.86. HRMS (ESI-TOF) *calcd for* $C_{33}H_{29}ClF_3N_8O_3S$, 709.1719 (M+H$^+$), *found* 709.1722.

(S)-3-(2-(4-(4-Chlorophenyl)-2,3,9-trimethyl-6H-thieno[3,2-f][1,2,4]triazolo[4,3-a][1,4]diazepin-6-yl)acetamido)-N-(2-(3-methyl-3H-diazirin-3-yl)ethyl)-N-(prop-2-yn-1-yl)propenamide (BD-JQ)

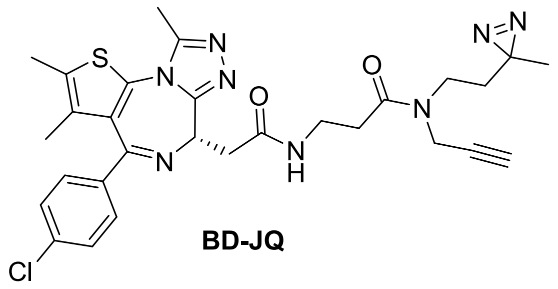

BD-JQ

Synthesized according to general procedure 1. Purified by PTLC (DCM/methanol, 19:1) to afford **BD-JQ** as a light brown liquid (7 mg, 56 %). ^{1}H NMR (600 MHz, $CDCl_3$) δ 7.44 – 7.39 (m, 4H), 7.34 – 7.32 (m, 4H), 6.98 (t, *J* = 6.0 Hz, 2H), 4.65 – 4.63 (m, 2H), 4.27 (dt, *J* = 17.6, 2.3 Hz, 1H), 4.15 (dt, *J* = 17.7, 2.4 Hz, 1H), 3.99 (dd, *J* = 5.5, 2.7 Hz, 2H), 3.73 – 3.64 (m, 3H), 3.63 – 3.54 (m, 3H), 3.51 – 3.44 (m, 5H), 3.39 – 4.33 (m, 4H), 2.66 (d, *J* = 2.0 Hz, 7H), 2.57 (d, *J* = 5.1 Hz, 2H), 2.40 (d, *J* = 2.0 Hz, 6H), 2.30 (q, *J* = 2.3 Hz, 1H), 2.24 (d, *J* = 2.4 Hz, 1H), 1.68 (s, 7H), 1.66 – 1.59 (m, 4H), 1.07 (d, *J* = 2.1 Hz, 3H), 1.06 (d, *J* = 2.1 Hz, 3H). ^{13}C NMR (151 MHz, $CDCl_3$) δ 171.44, 170.81, 170.59, 170.54, 163.86, 155.64, 149.81, 136.74, 136.63, 132.21, 130.89, 130.72, 130.43, 129.87, 128.70, 78.62, 78.13, 73.16, 72.36, 54.27, 54.23, 41.99, 41.78, 39.10, 37.64, 35.22, 34.18, 33.42, 33.21, 32.87, 32.70, 24.38, 23.86, 19.78, 19.59, 14.42, 13.10, 11.85. Note: rotomeric isomers observed. HRMS (ESI-TOF) *calcd for* $C_{29}H_{32}ClN_8O_2S$, 591.2052 (M+H$^+$), *found* 591.2051.

(3-(1,1-Difluoroprop-2-yn-1-yl)-3H-diazirin-3-yl)methyl (S)-(2-(2-(4-(4-chlorophenyl)-2,3,9-trimethyl-6H-thieno[3,2-f][1,2,4]triazolo[4,3-a][1,4]diazepin-6-yl)acetamido)ethyl)carbamate (DF-JQ)

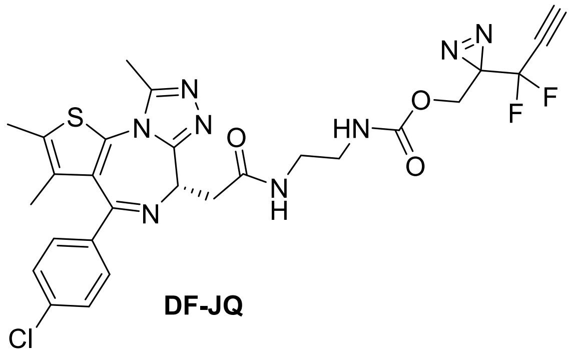

DF-JQ

Synthesized according to general procedure 4. Purified by PTLC (DCM/methanol, 19:1) to afford **DF-JQ** as a colorless sticky liquid (6.4 mg, 58 %). ^{1}H NMR (400 MHz, $CDCl_3$) δ 8.19 (t, *J* = 5.9 Hz, 1H), 7.38 (d, *J* = 8.6 Hz, 2H), 7.30 (d, *J* = 8.8 Hz, 2H), 6.84 (t, *J* = 5.4 Hz, 1H), 4.75 (dd, *J* = 7.8, 6.5 Hz, 1H), 4.23 (s, 2H), 3.63 – 3.57 (m, 2H), 3.49 – 3.33 (m, 4H), 3.06 (t, *J* = 5.1 Hz, 1H), 2.74 (s, 3H), 2.42 (s, 3H), 1.69 (s, 3H). ^{13}C NMR (101 MHz, $CDCl_3$) δ 171.01, 164.08, 155.85, 155.52, 150.18, 136.83, 136.45, 131.87, 131.12, 131.03, 130.75, 129.91, 128.69, 109.86 (t, *J* = 236.3 Hz), 80.42 (t, *J* = 6.5 Hz), 77.25, 72.76 (t, *J* = 40.4 Hz), 60.03, 54.21, 41.59, 39.40, 38.84, 29.88 (t, *J* = 35.3 Hz), 14.41, 13.12, 11.71. ^{19}F NMR (376 MHz, $CDCl_3$) δ -86.84 (d, *J* = 5.5 Hz). HRMS (ESI-TOF) *calcd for* $C_{27}H_{26}ClF_2N_8O_3S$, 615.1500 (M+H$^+$), *found* 615.1499.

(S)-N-((3H-Diazirin-3-yl)methyl)-3-(2-(4-(4-chlorophenyl)-2,3,9-trimethyl-6H-thieno[3,2-f][1,2,4]triazolo[4,3-a][1,4]diazepin-6-yl)acetamido)-N-(prop-2-yn-1-yl)propenamide (Tm-JQ)

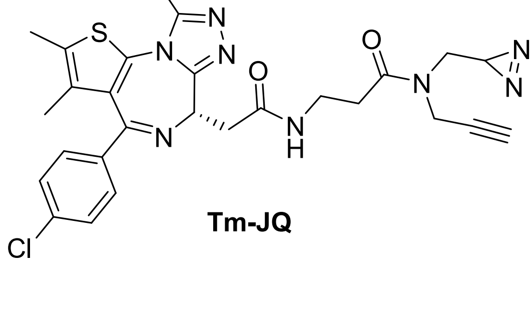

Tm-JQ

Synthesized according to general procedure 1. Purified by PTLC (DCM/methanol, 19:1) to afford **Tm-JQ** as a light yellow solid (8 mg, 57 %). ^{1}H NMR (600 MHz, $CDCl_3$) δ 7.35 (d, *J* = 8.3 Hz, 4H), 7.27 (d, *J* = 8.6 Hz, 4H), 7.00 – 6.88 (m, 2H), 4.57 – 4.51 (m, 2H), 4.22 – 4.09 (m, 2H), 3.98 (t, *J* = 2.1 Hz, 2H), 3.64 – 6.68 (m, 4H), 3.63 – 3.56 (m, 2H), 3.55 – 3.48 (m, 2H), 3.45 – 3.39 (m, 2H), 3.32 – 3.28 (m, 6H), 3.12 (q, *J* = 7.4 Hz, 4H), 2.59 (s, 7H), 2.51 – 2.42 (m, 2H), 2.33 (s, 5H), 2.26 (t, *J* = 2.5 Hz, 1H), 2.19 (t, *J* = 2.5 Hz, 1H), 1.61 (s, 7H), 1.41 – 1.38 (m, 4H). ^{13}C NMR (151 MHz, $CDCl_3$) δ 171.74, 171.17, 170.73, 170.66, 164.02, 155.63, 149.91, 136.85, 136.56, 132.14, 130.94, 130.90, 130.48, 129.90, 128.75, 73.52, 72.73, 55.79, 54.23, 45.84, 43.75, 39.06, 38.24, 35.13, 32.86, 29.72, 19.55, 18.62, 17.23, 14.42, 13.12, 12.56, 11.83. Note: rotomeric isomers observed. HRMS (ESI-TOF) *calcd for* $C_{27}H_{29}ClN_8O_2S$, 563.1739 ($M+H^+$), *found* 563.1741.

References

1. J. G. K. O'Brien, A. Jemas, P. N. Asare-Okai, C. W. Am Ende and J. M. Fox, *Org Lett*, 2020, **22**, 9415-9420.
2. Y. Perez-Riverol, A. Csordas, J. W. Bai, M. Bernal-Llinares, S. Hewapathirana, D. J. Kundu, A. Inuganti, J. Griss, G. Mayer, M. Eisenacher, E. Perez, J. Uszkoreit, J. Pfeuffer, T. Sachsenberg, S. Yilmaz, S. Tiwary, J. Cox, E. Audain, M. Walzer, A. F. Jarnuczak, T. Ternent, A. Brazma and J. A. Vizcaino, *Nucleic Acids Research*, 2019, **47**, D442-D450.
3. T. Glachet, H. Marzag, N. Saraiva Rosa, J. F. P. Colell, G. Zhang, W. S. Warren, X. Franck, T. Theis and V. Reboul, *J Am Chem Soc*, 2019, **141**, 13689-13696.
4. T. Kambe, B. E. Correia, M. J. Niphakis and B. F. Cravatt, *Journal of the American Chemical Society*, 2014, **136**, 10777-10782.
5. C. F. Chang, A. Mfuh, J. X. Gao, H. Y. Wu and C. M. Woo, *Tetrahedron*, 2018, **74**, 3273-3277.
6. K. Qin, Y. Zhu, W. Qin, J. Gao, X. Shao, Y. L. Wang, W. Zhou, C. Wang and X. Chen, *ACS Chem Biol*, 2018, **13**, 1983-1989.
7. C. Palomo, J. M. Aizpurua, E. Balentova, A. Jimenez, J. Oyarbide, R. M. Fratila and J. I. Miranda, *Organic Letters*, 2007, **9**, 101-104.
8. J. Fernandez-Valparis, P. Romea, F. Urpi and M. Font-Bardia, *Organic Letters*, 2017, **19**, 6400-6403.
9. J. X. Gao, A. Mfuh, Y. Amako and C. M. Woo, *Journal of the American Chemical Society*, 2018, **140**, 4259-4268.
10. J. Szychowski, A. Mahdavi, J. J. L. Hodas, J. D. Bagert, J. T. Ngo, P. Landgraf, D. C. Dieterich, E. M. Schuman and D. A. Tirrell, *Journal of the American Chemical Society*, 2010, **132**, 18351-18360.
