# Supplementary material for: Evaluation of fully-functionalized diazirine tags for chemical proteomic applications
Source: Chem Sci. 2021 May 7;12(22):7839–47. doi: 10.1039/d1sc01360b (PMC8188597; doi:10.1039/d1sc01360b)
Supplement: SC-012-D1SC01360B-s003 [file SC-012-D1SC01360B-s003.pdf]

Electronic Supplementary Material (ESI) for Chemical Science.
This journal is © The Royal Society of Chemistry 2021

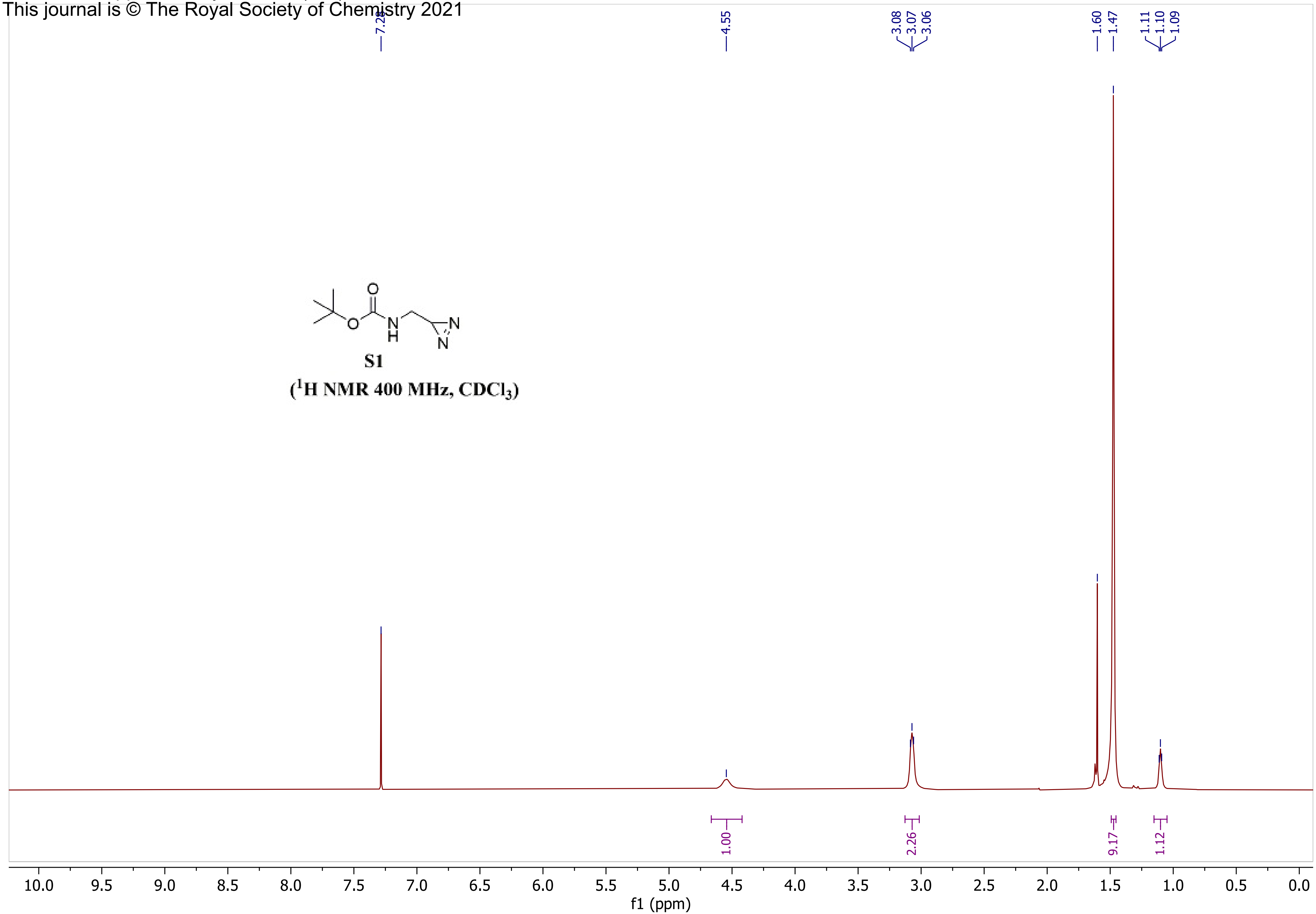

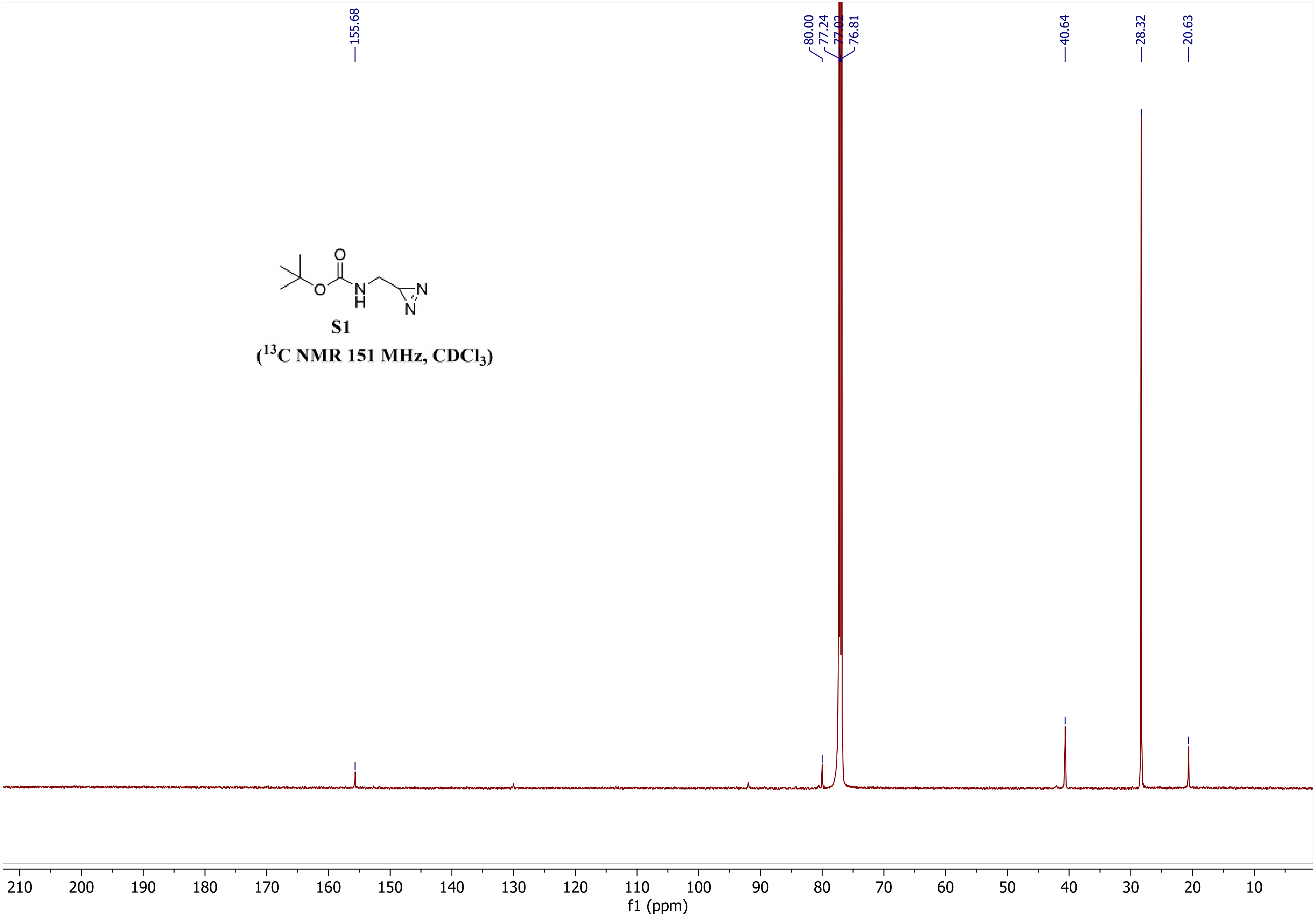
155.68
80.00
77.24
77.02
76.81
40.64
28.32
20.63
O
O
N
H
N
N
S1
(13C NMR 151 MHz, CDCl3)
210 200 190 180 170 160 150 140 130 120 110 100 90 80 70 60 50 40 30 20 10
f1 (ppm)

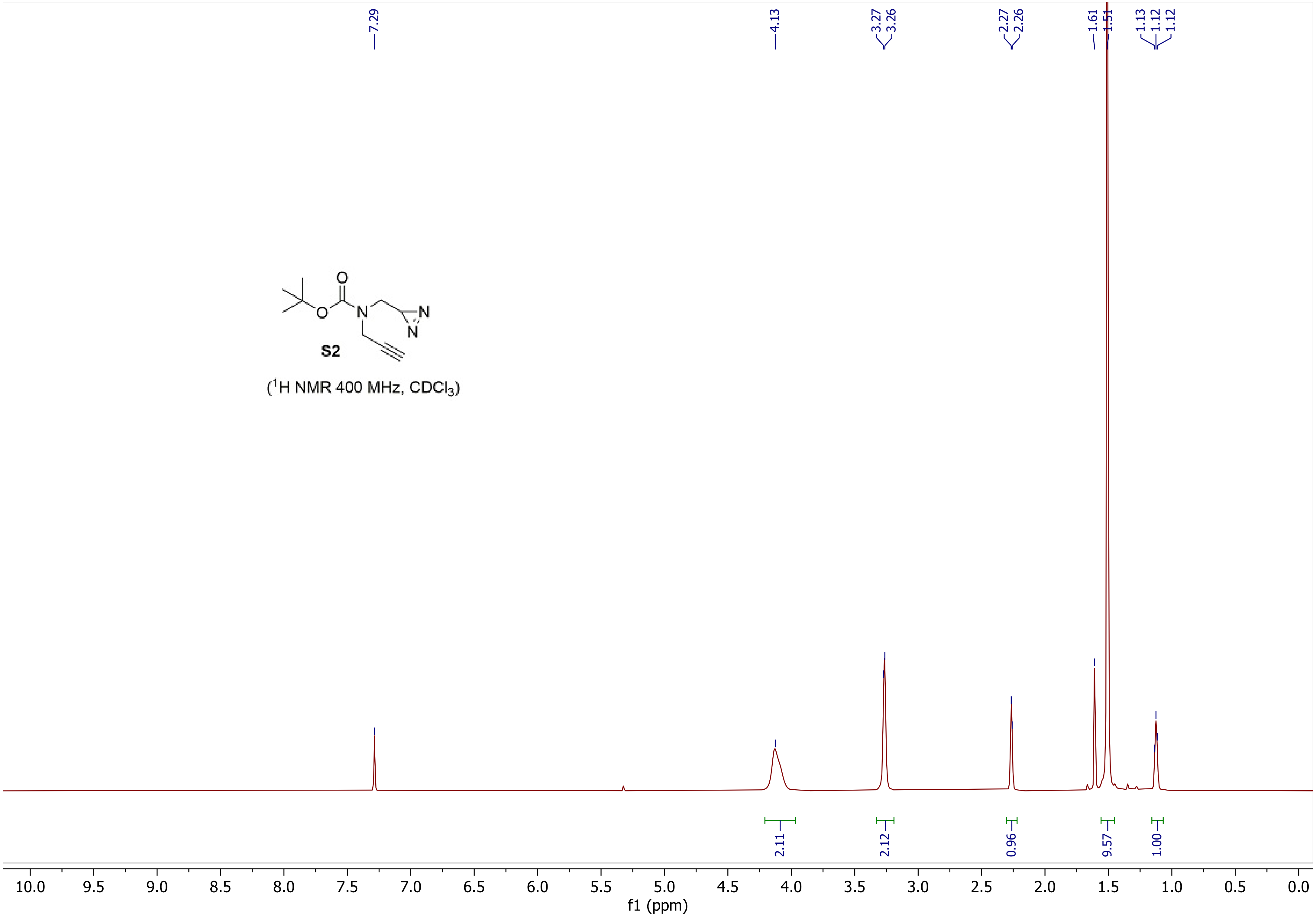
7.29
4.13
3.27
3.26
2.27
2.26
1.61
1.51
1.13
1.12
1.12
O
O
N
N
N
S2
(^{1}H NMR 400 MHz, $CDCl_3$)
2.11
2.12
0.96
9.57
1.00
10.0 9.5 9.0 8.5 8.0 7.5 7.0 6.5 6.0 5.5 5.0 4.5 4.0 3.5 3.0 2.5 2.0 1.5 1.0 0.5 0.0
f1 (ppm)

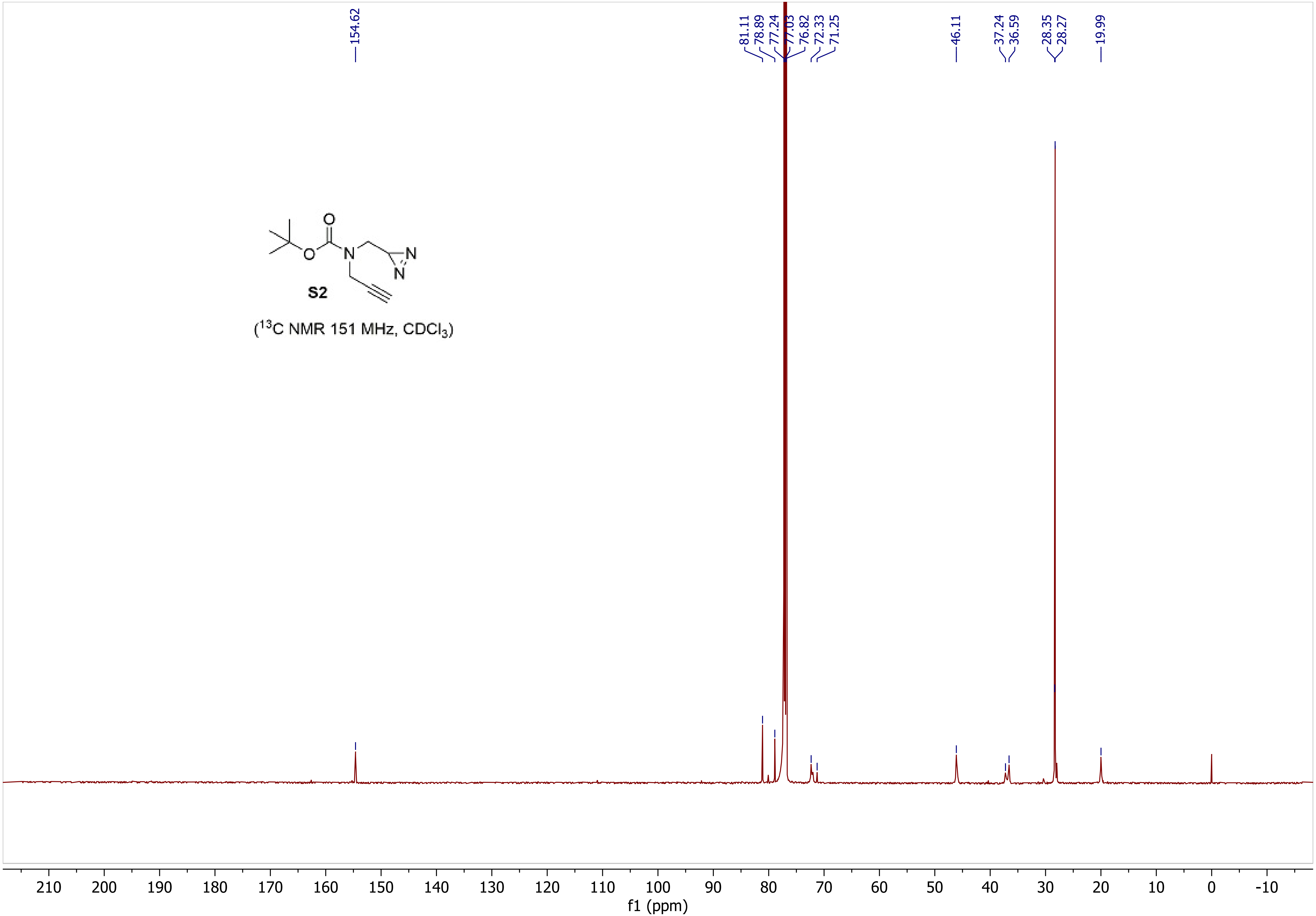
154.62
81.11
78.89
77.24
77.03
76.82
72.33
71.25
46.11
37.24
36.59
28.35
28.27
19.99
O
O
N
N
N
S2
(^{13}C NMR 151 MHz, CDCl$_3$)
210
200
190
180
170
160
150
140
130
120
110
100
90
80
70
60
50
40
30
20
10
0
-10
f1 (ppm)

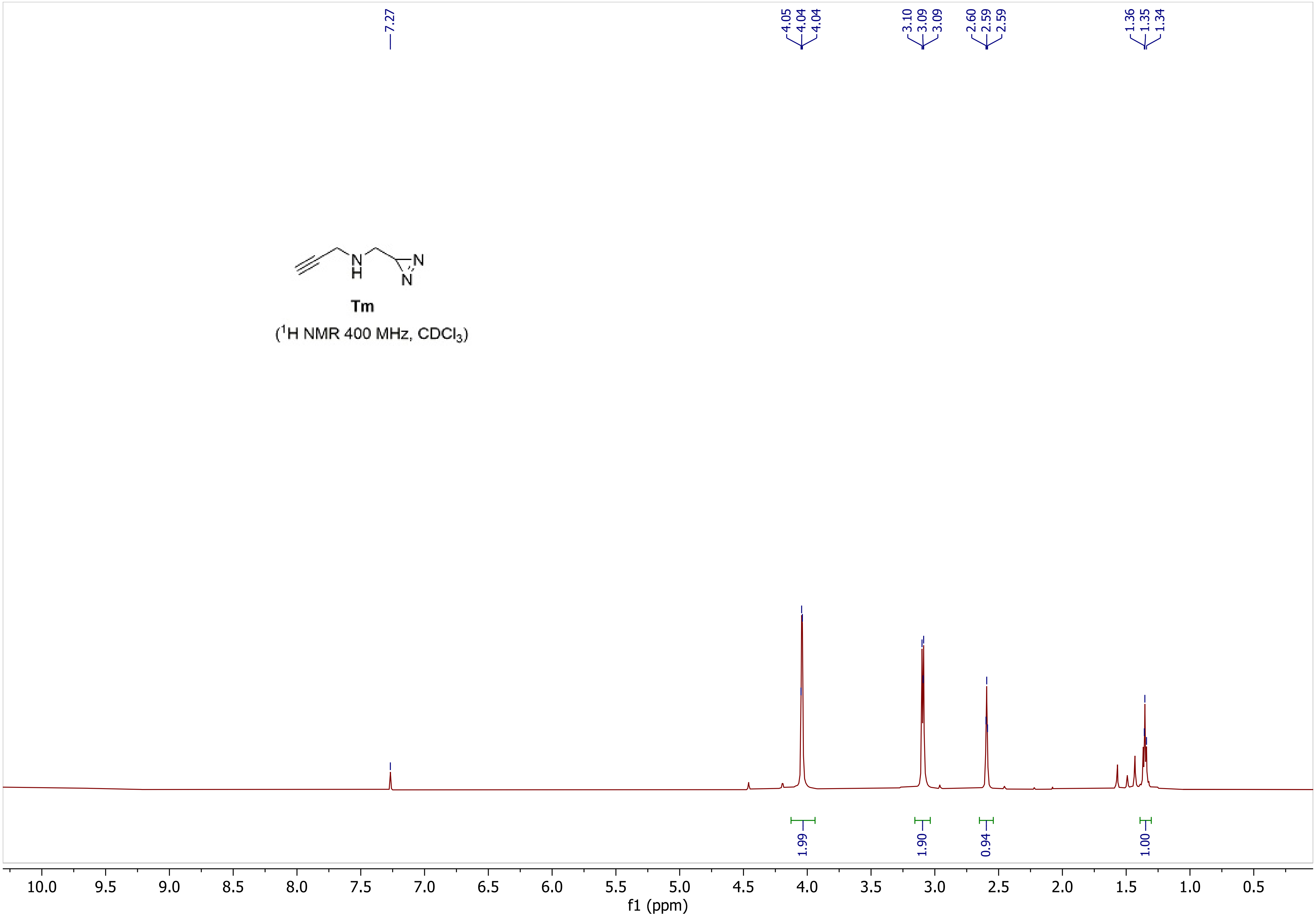
7.27
4.05
4.04
4.04
3.10
3.09
3.09
2.60
2.59
2.59
1.36
1.35
1.34
Tm
(1H NMR 400 MHz, CDCl3)
1.99
1.90
0.94
1.00
10.0
9.5
9.0
8.5
8.0
7.5
7.0
6.5
6.0
5.5
5.0
4.5
4.0
3.5
3.0
2.5
2.0
1.5
1.0
0.5
f1 (ppm)

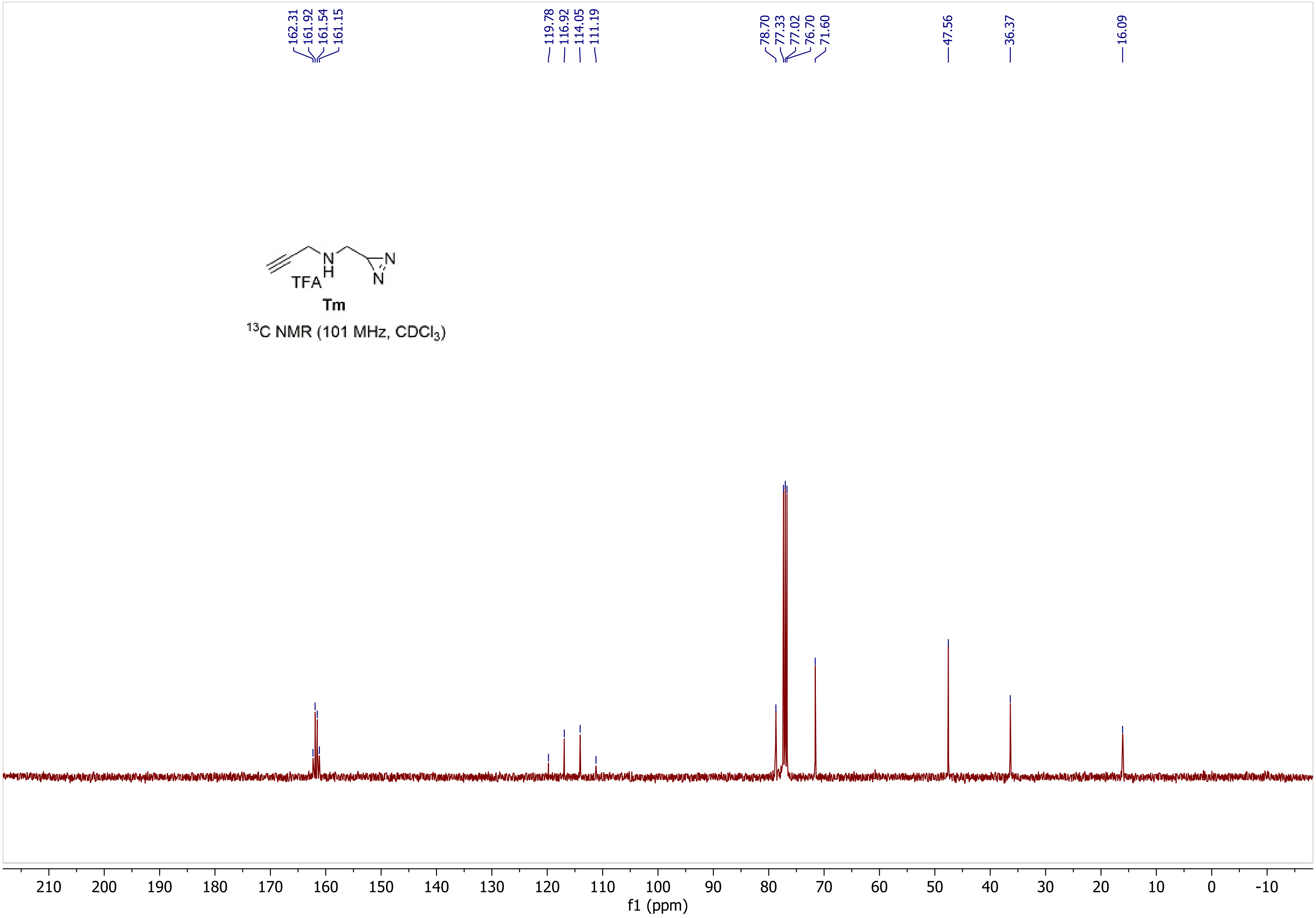
162.31
161.92
161.54
161.15
119.78
116.92
114.05
111.19
78.70
77.33
77.02
76.70
71.60
47.56
36.37
16.09
TFA
H
N
N
N
Tm
13C NMR (101 MHz, CDCl3)
210 200 190 180 170 160 150 140 130 120 110 100 90 80 70 60 50 40 30 20 10 0 -10
f1 (ppm)

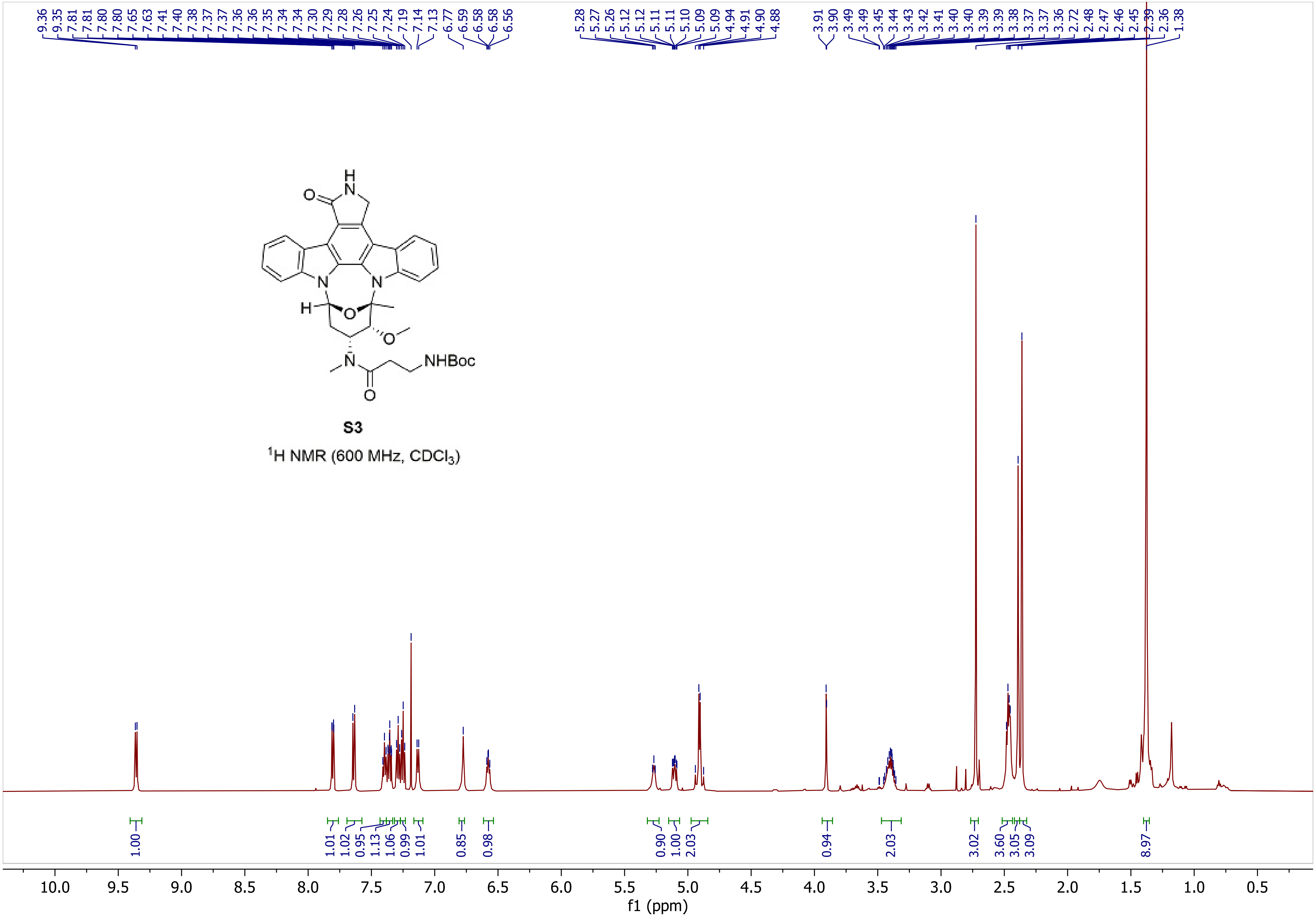

S3

^{1}H NMR (600 MHz, $CDCl_3$)

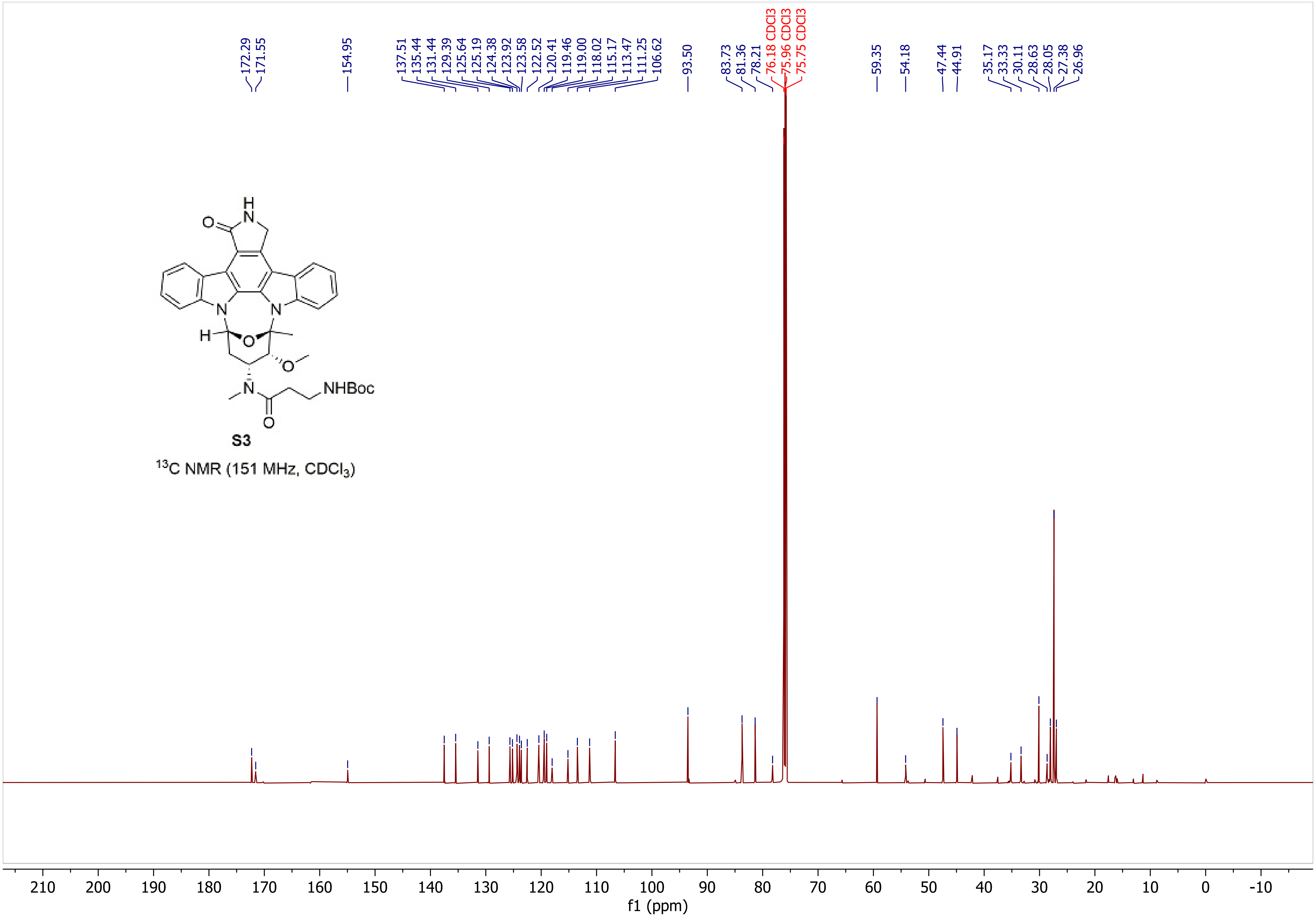
172.29
171.55
154.95
137.51
135.44
131.44
129.39
125.64
125.19
124.38
123.92
123.58
122.52
120.41
119.46
119.00
118.02
115.17
113.47
111.25
106.62
93.50
83.73
81.36
78.21
76.18 CDCl3
75.96 CDCl3
75.75 CDCl3
59.35
54.18
47.44
44.91
35.17
33.33
30.11
28.63
28.05
27.38
26.96
H
O
N
N
N
H
O
O
N
NHBoc
O
S3
^{13}C NMR (151 MHz, CDCl$_3$)
210
200
190
180
170
160
150
140
130
120
110
100
90
80
70
60
50
40
30
20
10
0
-10
f1 (ppm)

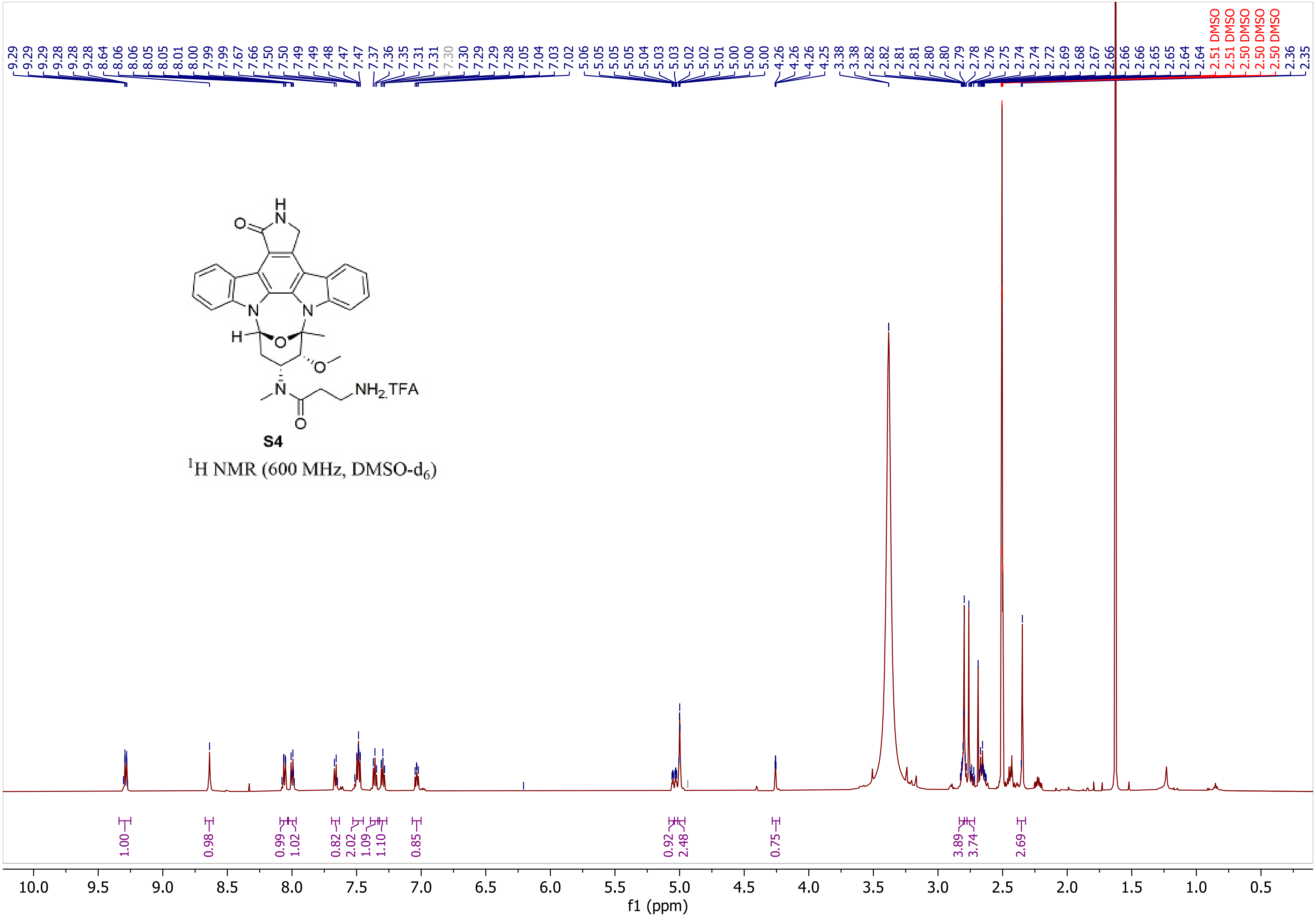

S4
^{1}H NMR (600 MHz, DMSO-d$_6$)
NH2.TFA
f1 (ppm)

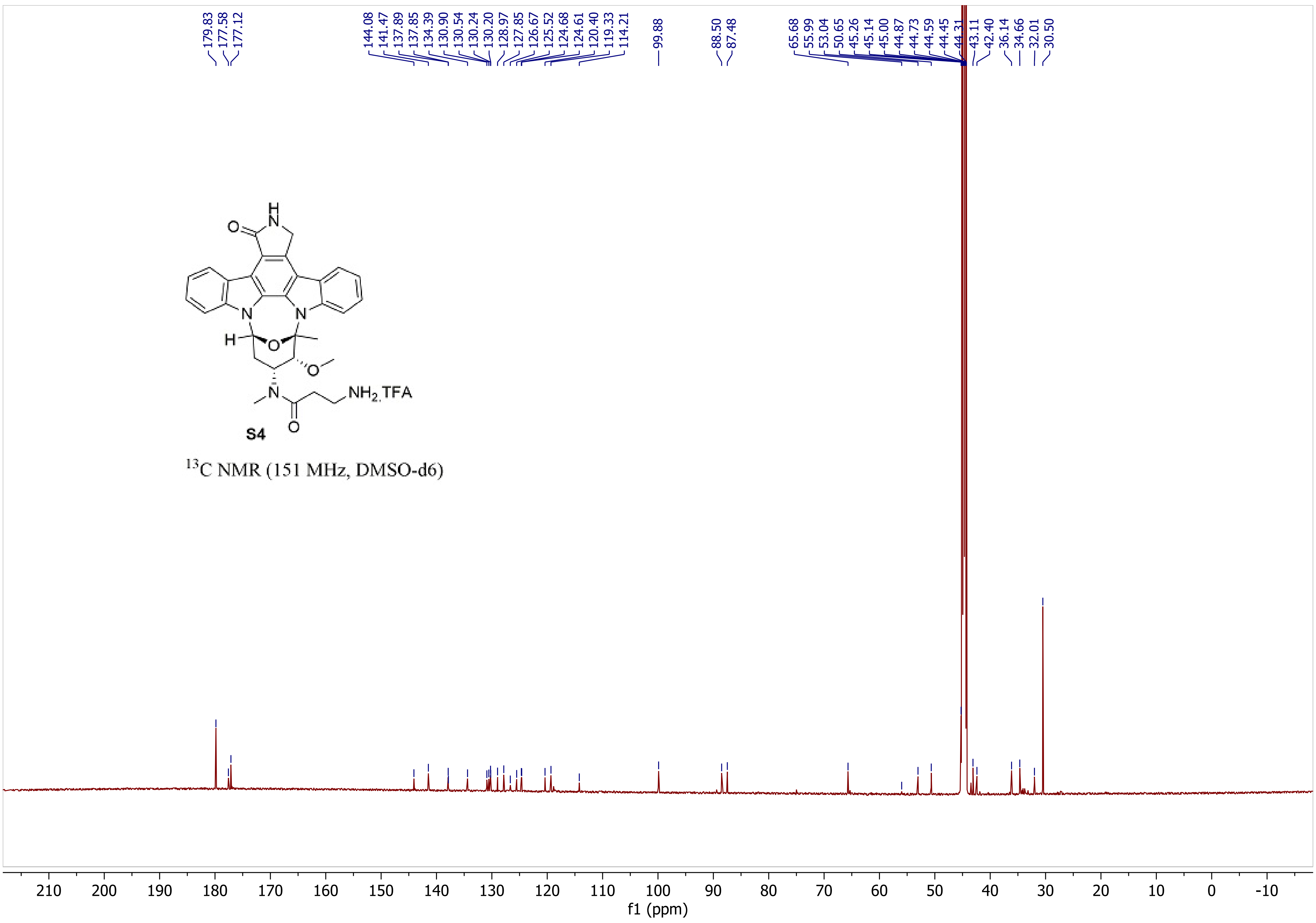
179.83
177.58
177.12
144.08
141.47
137.89
137.85
134.39
130.90
130.54
130.24
130.20
128.97
127.85
126.67
125.52
124.68
124.61
120.40
119.33
114.21
99.88
88.50
87.48
65.68
55.99
53.04
50.65
45.26
45.14
45.00
44.87
44.73
44.59
44.45
44.31
43.11
42.40
36.14
34.66
32.01
30.50
H
N
O
H
O
N
N
O
N
NH2.TFA
O
S4
13C NMR (151 MHz, DMSO-d6)
210 200 190 180 170 160 150 140 130 120 110 100 90 80 70 60 50 40 30 20 10 0 -10
f1 (ppm)

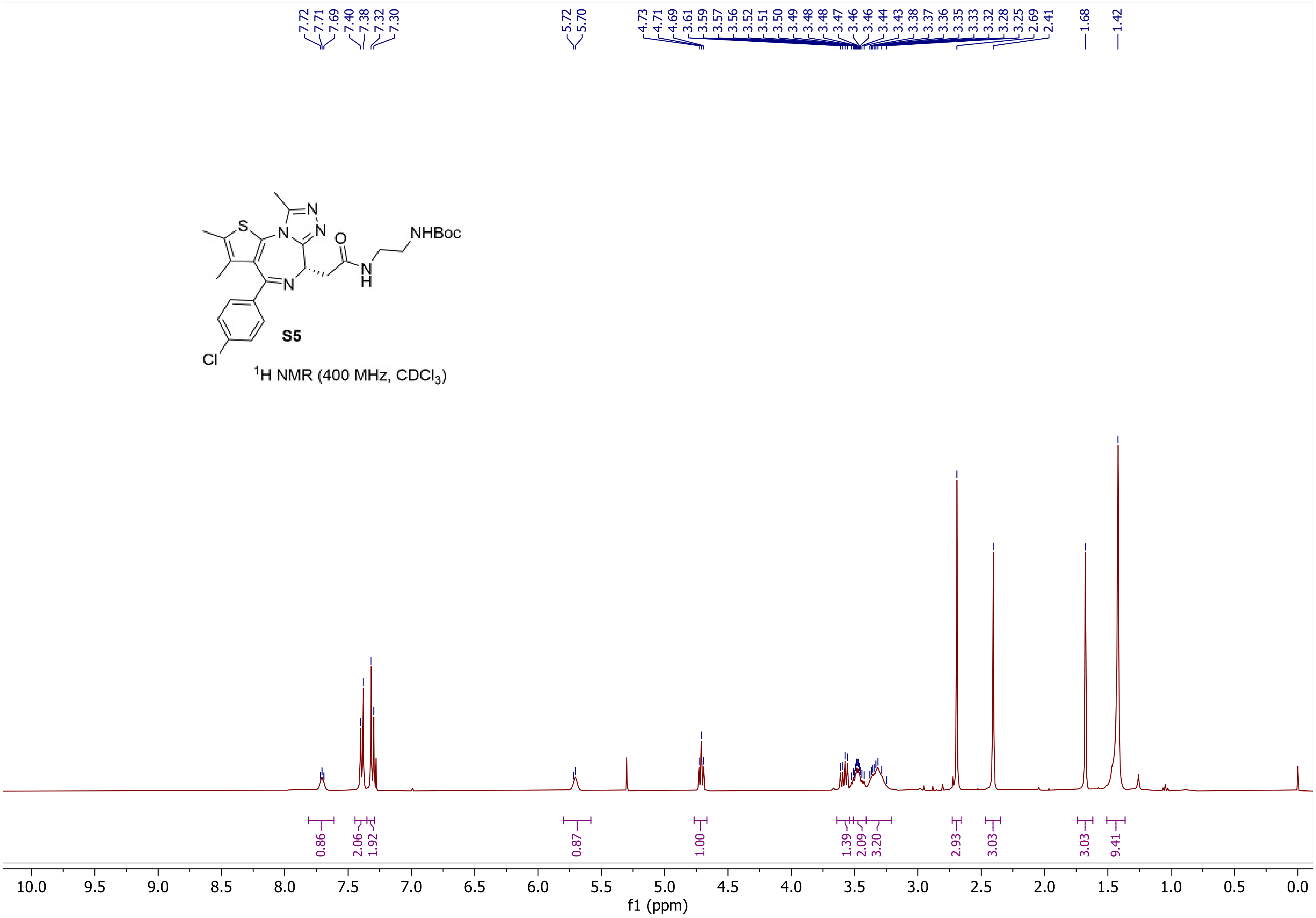
7.72 7.71 7.69 7.40 7.38 7.32 7.30
5.72 5.70
4.73 4.71 4.69 3.61 3.59 3.57 3.56 3.52 3.51 3.50 3.49 3.48 3.48 3.47 3.46 3.46 3.44 3.43 3.38 3.37 3.36 3.35 3.33 3.32 3.28 3.25 2.69 2.41
1.68
1.42
NHBoc
S5
1H NMR (400 MHz, CDCl3)
0.86 2.06 1.92 0.87 1.00 1.39 2.09 3.20 2.93 3.03 3.03 9.41
10.0 9.5 9.0 8.5 8.0 7.5 7.0 6.5 6.0 5.5 5.0 4.5 4.0 3.5 3.0 2.5 2.0 1.5 1.0 0.5 0.0
f1 (ppm)

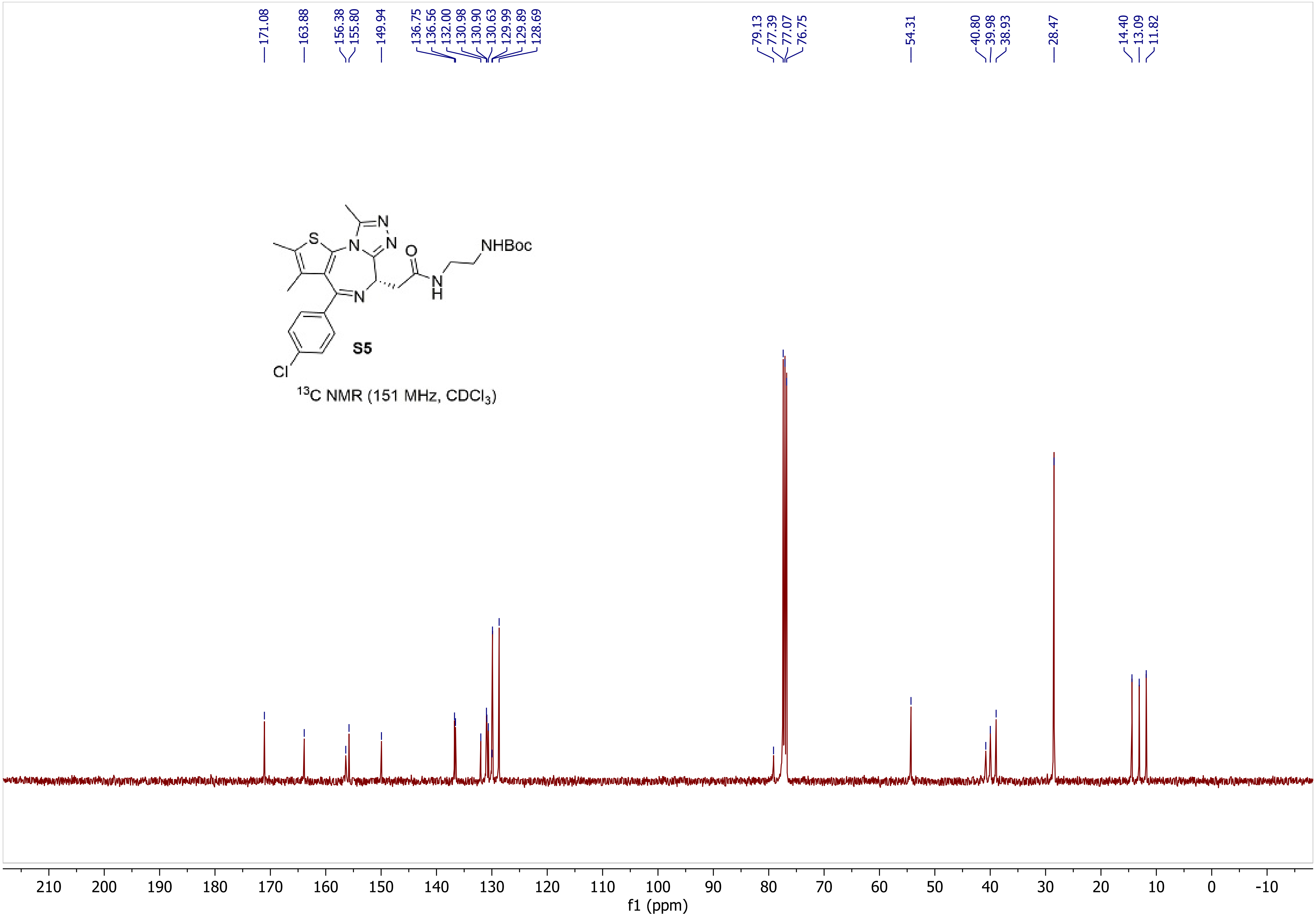
171.08
163.88
156.38
155.80
149.94
136.75
136.56
132.00
130.98
130.90
130.63
129.99
129.89
128.69
79.13
77.39
77.07
76.75
54.31
40.80
39.98
38.93
28.47
14.40
13.09
11.82
S
N
N
N
O
NHBoc
N
H
N
Cl
S5
13C NMR (151 MHz, CDCl3)
210
200
190
180
170
160
150
140
130
120
110
100
90
80
70
60
50
40
30
20
10
0
-10
f1 (ppm)

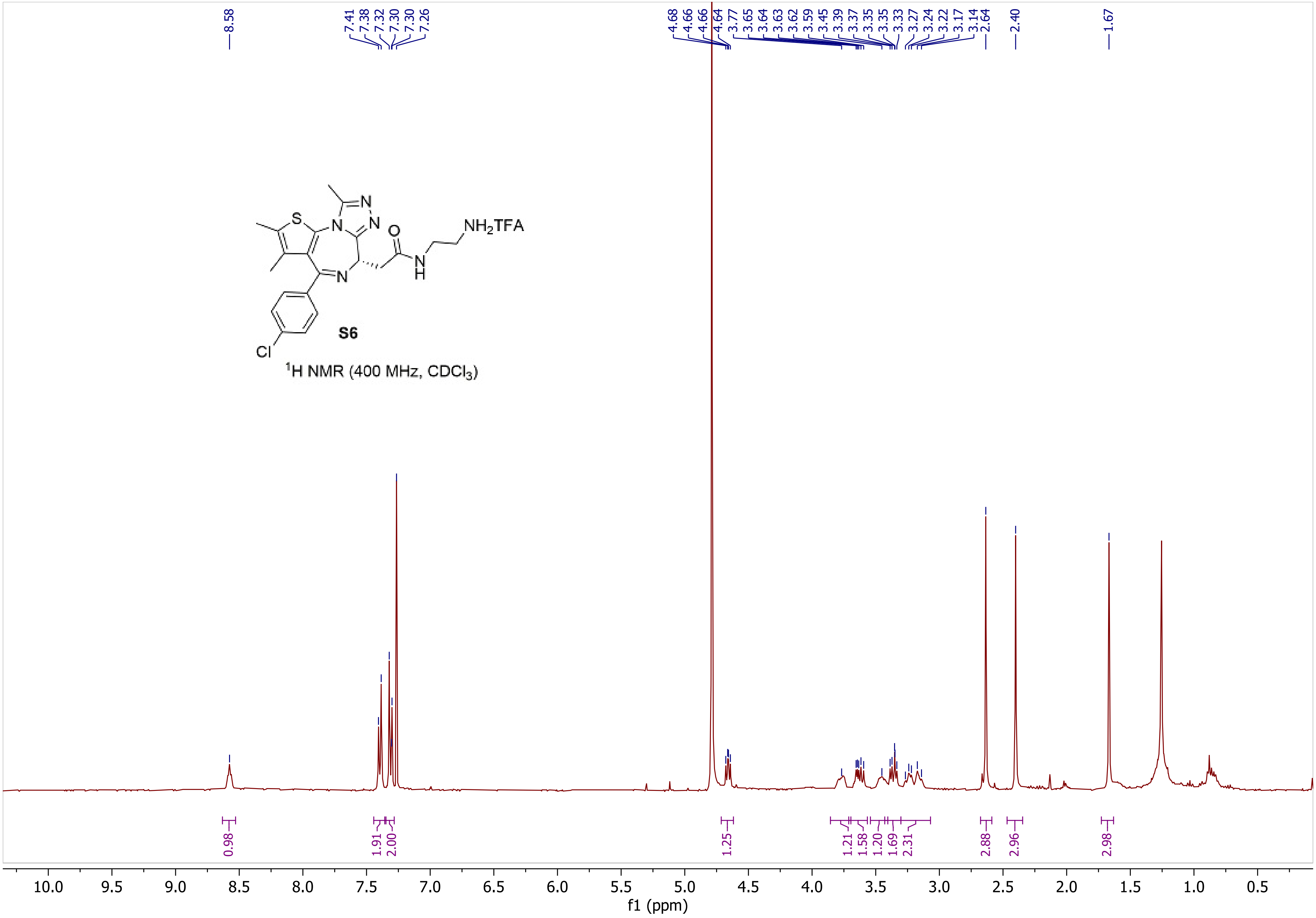
8.58
7.41
7.38
7.32
7.30
7.30
7.26
4.68
4.66
4.66
4.64
3.77
3.65
3.64
3.63
3.62
3.59
3.45
3.39
3.37
3.35
3.35
3.33
3.27
3.24
3.22
3.17
3.14
2.64
2.40
1.67
NH2TFA
S6
1H NMR (400 MHz, CDCl3)
0.98
1.91
2.00
1.25
1.21
1.58
1.20
1.69
2.31
2.88
2.96
2.98
10.0
9.5
9.0
8.5
8.0
7.5
7.0
6.5
6.0
5.5
5.0
4.5
4.0
3.5
3.0
2.5
2.0
1.5
1.0
0.5
f1 (ppm)

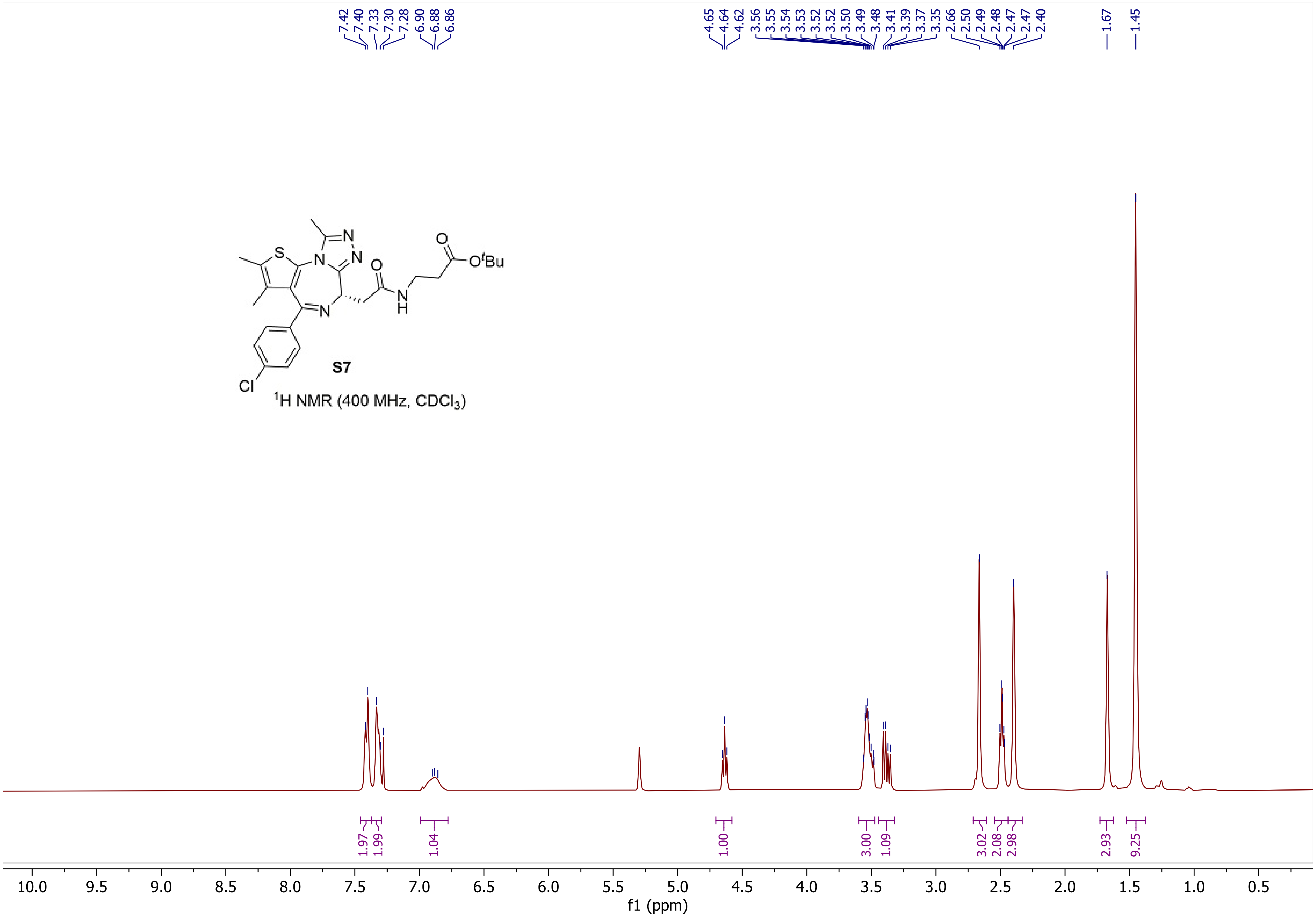

7.42
7.40
7.33
7.30
7.28
6.90
6.88
6.86
4.65
4.64
4.62
3.56
3.55
3.54
3.53
3.52
3.52
3.50
3.49
3.48
3.41
3.39
3.37
3.35
2.66
2.50
2.49
2.48
2.47
2.47
2.40
1.67
1.45
S
N
N
N
O
O
O^{t}Bu
N
H
N
Cl
S7
^{1}H NMR (400 MHz, $CDCl_3$)
1.97
1.99
1.04
1.00
3.00
1.09
3.02
2.08
2.98
2.93
9.25
10.0
9.5
9.0
8.5
8.0
7.5
7.0
6.5
6.0
5.5
5.0
4.5
4.0
3.5
3.0
2.5
2.0
1.5
1.0
0.5
f1 (ppm)

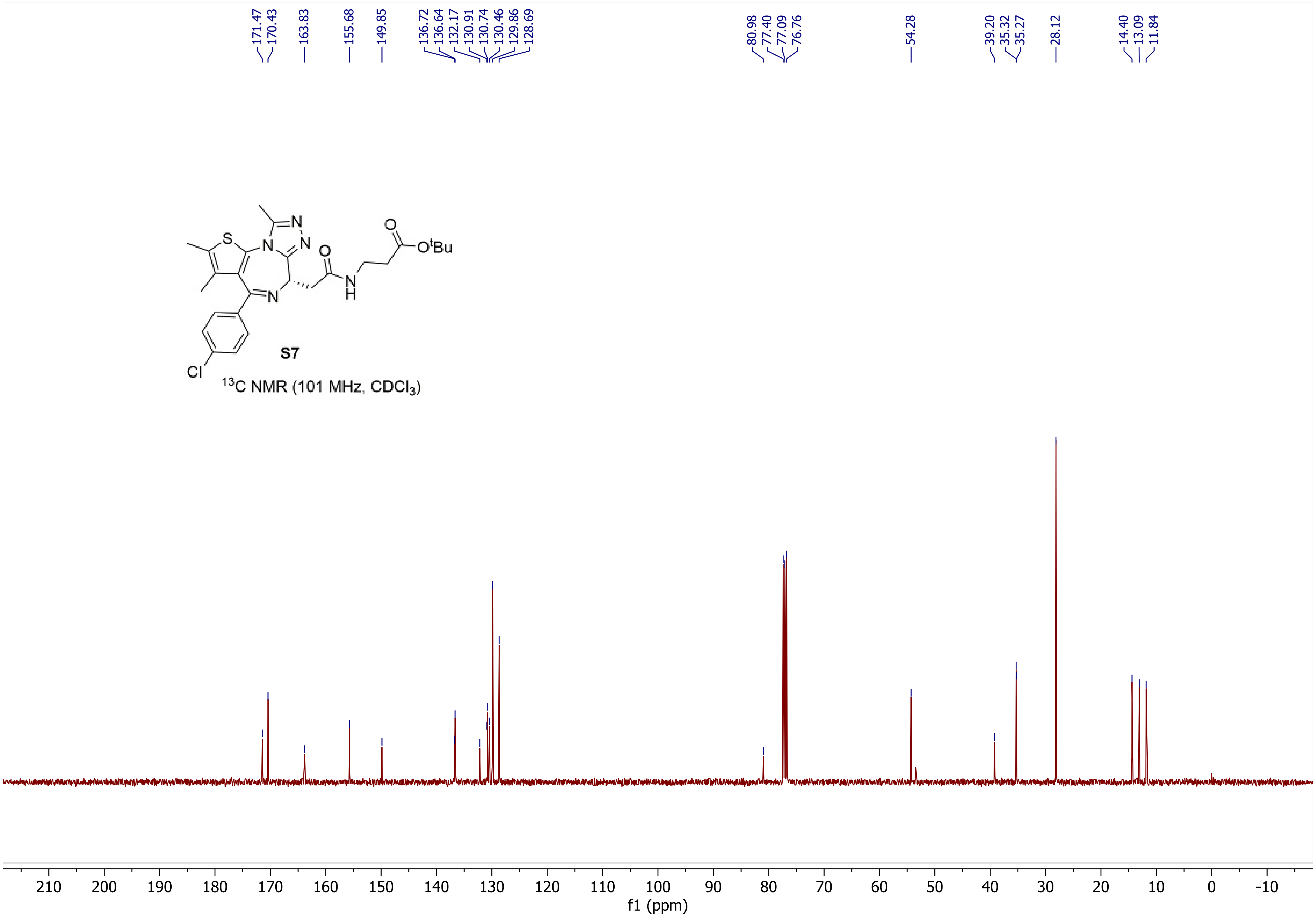
171.47
170.43
163.83
155.68
149.85
136.72
136.64
132.17
130.91
130.74
130.46
129.86
128.69
80.98
77.40
77.09
76.76
54.28
39.20
35.32
35.27
28.12
14.40
13.09
11.84
S
N
N
N
O
N
H
O
O^{t}Bu
N
Cl
S7
^{13}C NMR (101 MHz, $CDCl_3$)
210
200
190
180
170
160
150
140
130
120
110
100
90
80
70
60
50
40
30
20
10
0
-10
f1 (ppm)

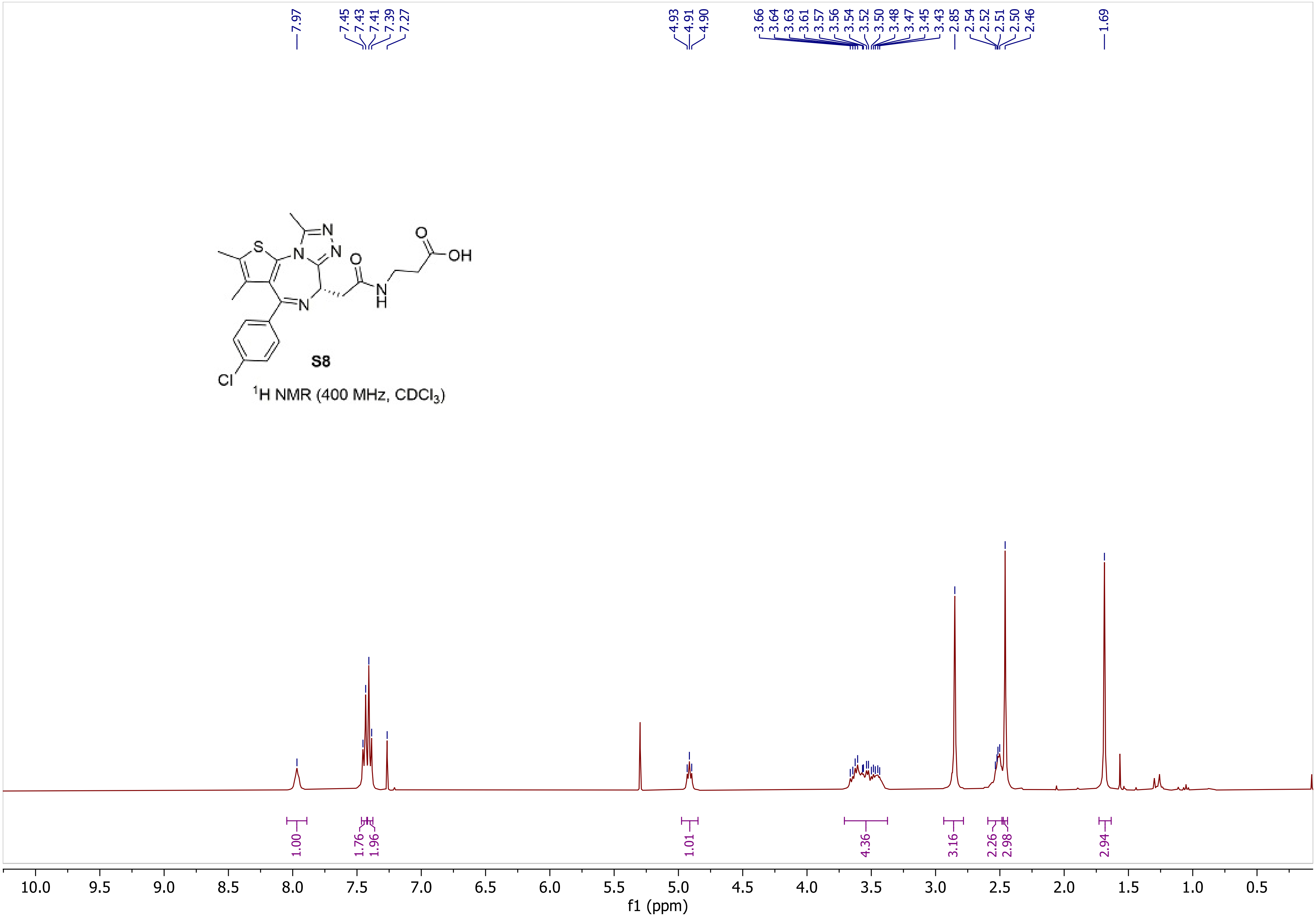

7.97
7.45 7.43 7.41 7.39 7.27
4.93 4.91 4.90
3.66 3.64 3.63 3.61 3.57 3.56 3.54 3.52 3.50 3.48 3.47 3.45 3.43
2.85
2.54 2.52 2.51 2.50 2.46
1.69
S
N
O
OH
H
Cl
S8
1H NMR (400 MHz, CDCl3)
1.00 1.76 1.96 1.01 4.36 3.16 2.26 2.98 2.94
10.0 9.5 9.0 8.5 8.0 7.5 7.0 6.5 6.0 5.5 5.0 4.5 4.0 3.5 3.0 2.5 2.0 1.5 1.0 0.5
f1 (ppm)

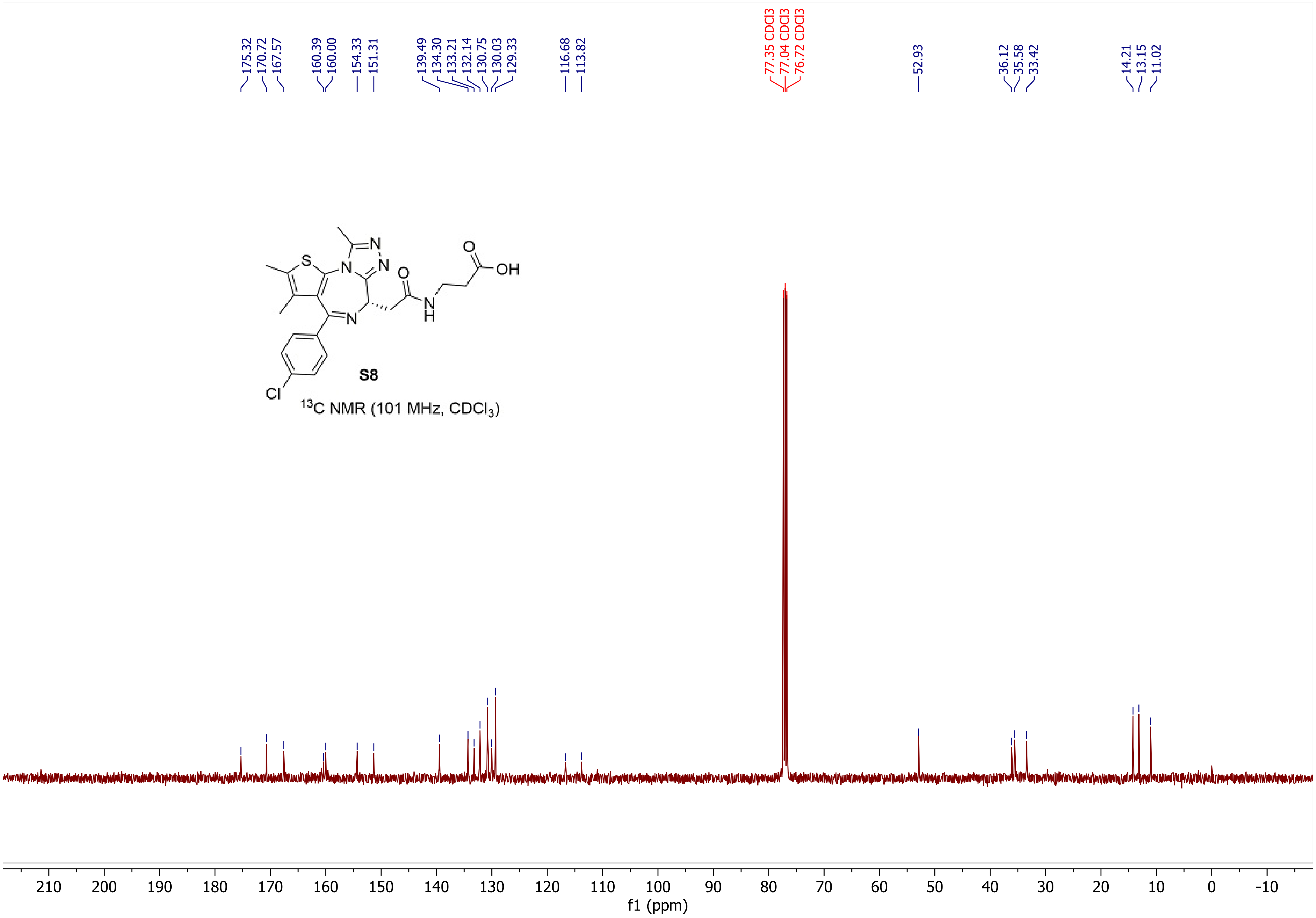
175.32
170.72
167.57
160.39
160.00
154.33
151.31
139.49
134.30
133.21
132.14
130.75
130.03
129.33
116.68
113.82
77.35 CDCl3
77.04 CDCl3
76.72 CDCl3
52.93
36.12
35.58
33.42
14.21
13.15
11.02
S8
^{13}C NMR (101 MHz, $CDCl_3$)
f1 (ppm)

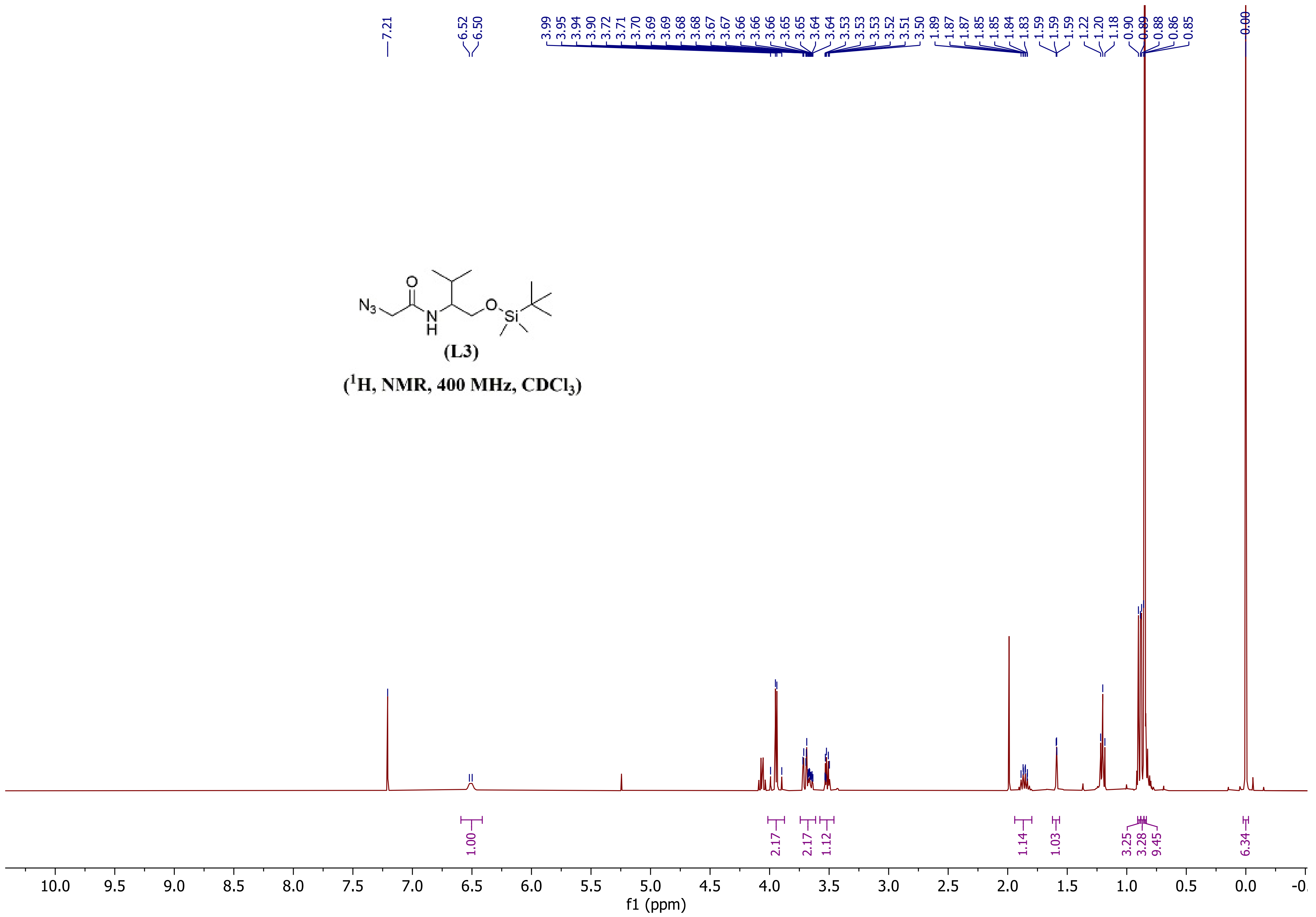
7.21
6.52
6.50
3.99 3.95 3.94 3.90 3.72 3.71 3.70 3.69 3.69 3.68 3.68 3.67 3.67 3.66 3.66 3.66 3.65 3.65 3.64 3.64 3.53 3.53 3.53 3.52 3.51 3.50
1.89 1.87 1.87 1.85 1.85 1.84 1.83
1.59 1.59 1.59
1.22 1.20 1.18
0.90 0.89 0.88 0.86 0.85
0.00
N_3
O
N
H
O
Si
(L3)
(^{1}H, NMR, 400 MHz, $CDCl_3$)
1.00
2.17
2.17
1.12
1.14
1.03
3.25
3.28
9.45
6.34
10.0 9.5 9.0 8.5 8.0 7.5 7.0 6.5 6.0 5.5 5.0 4.5 4.0 3.5 3.0 2.5 2.0 1.5 1.0 0.5 0.0
f1 (ppm)

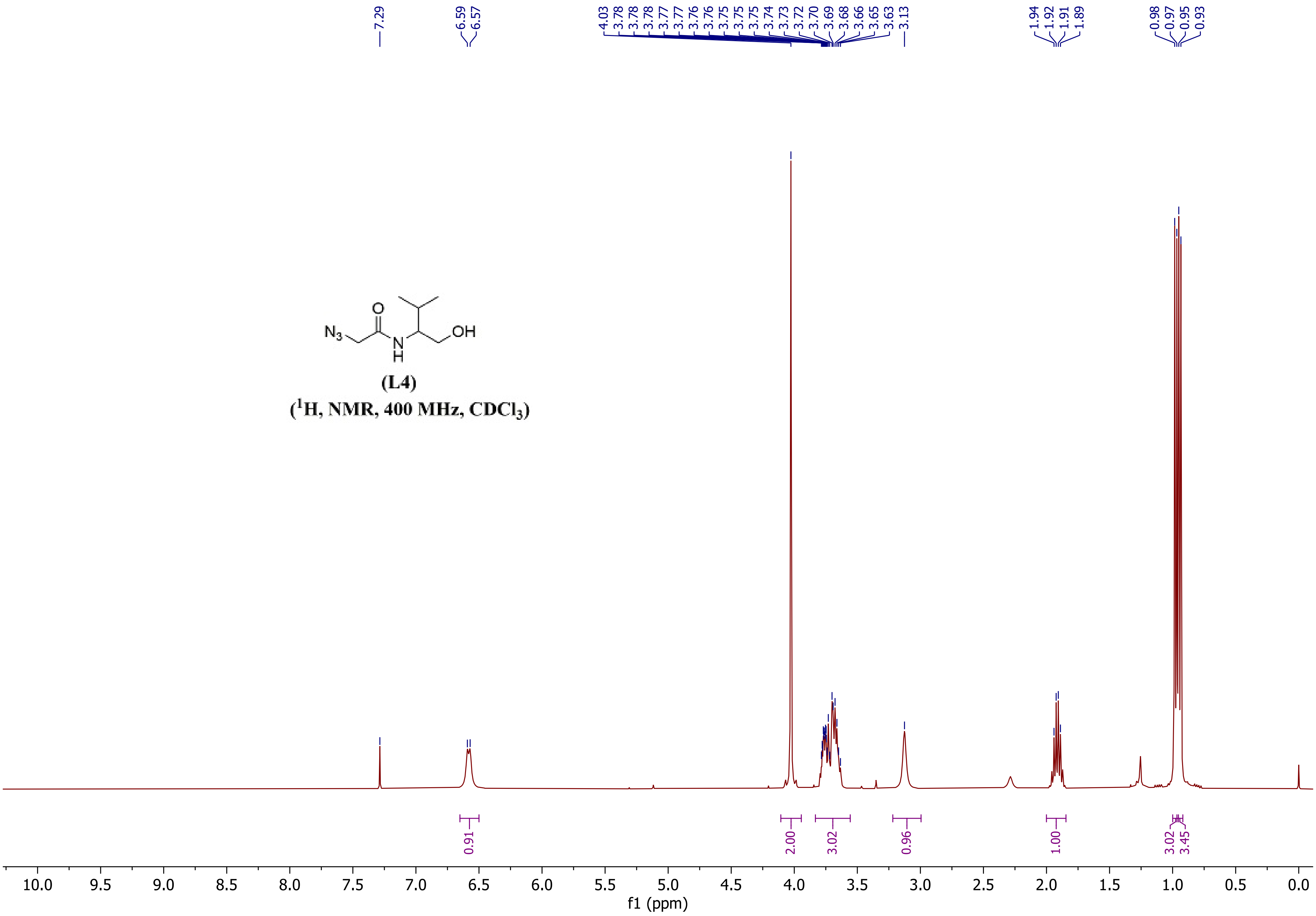
7.29
6.59
6.57
4.03
3.78
3.78
3.78
3.77
3.77
3.76
3.76
3.75
3.75
3.75
3.74
3.73
3.72
3.70
3.69
3.68
3.66
3.65
3.63
3.13
1.94
1.92
1.91
1.89
0.98
0.97
0.95
0.93
N3
O
N
H
OH
(L4)
(1H, NMR, 400 MHz, CDCl3)
0.91
2.00
3.02
0.96
1.00
3.02
3.45
10.0
9.5
9.0
8.5
8.0
7.5
7.0
6.5
6.0
5.5
5.0
4.5
4.0
3.5
3.0
2.5
2.0
1.5
1.0
0.5
0.0
f1 (ppm)

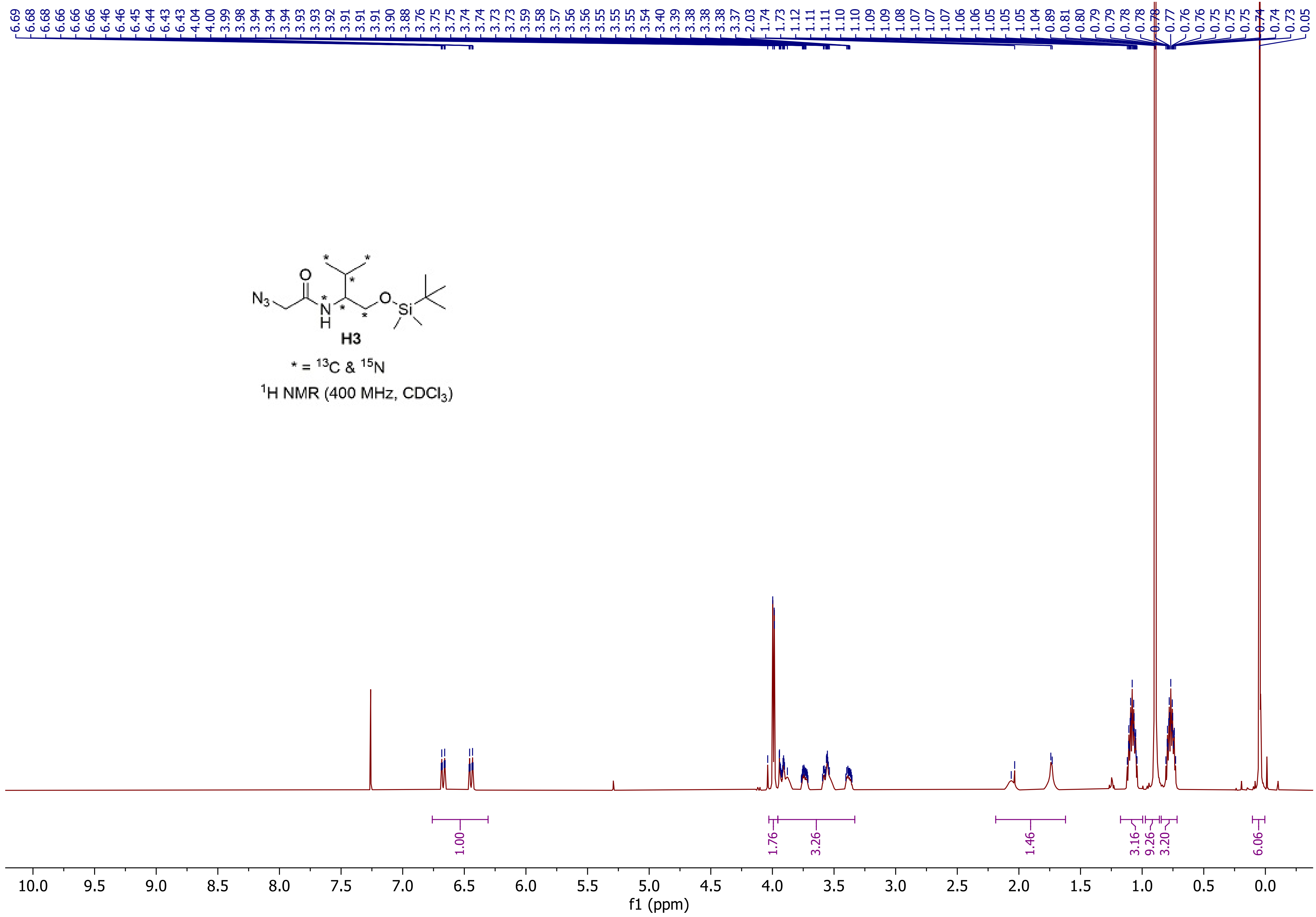

6.69 6.68 6.68 6.66 6.66 6.66 6.46 6.46 6.45 6.44 6.43 6.43 4.04 4.00 3.99 3.98 3.94 3.94 3.93 3.93 3.92 3.91 3.91 3.91 3.90 3.88 3.76 3.75 3.75 3.74 3.74 3.73 3.73 3.59 3.58 3.57 3.56 3.56 3.55 3.55 3.55 3.54 3.40 3.39 3.38 3.38 3.38 3.37 2.03 1.74 1.73 1.12 1.11 1.11 1.10 1.10 1.09 1.09 1.08 1.07 1.07 1.07 1.06 1.06 1.05 1.05 1.05 1.04 0.89 0.81 0.80 0.79 0.79 0.78 0.78 0.78 0.77 0.76 0.76 0.75 0.75 0.75 0.74 0.74 0.73 0.05
N3
O
N
H
O
Si
H3
* = 13C & 15N
1H NMR (400 MHz, CDCl3)
1.00
1.76
3.26
1.46
3.16
9.26
3.20
6.06
10.0 9.5 9.0 8.5 8.0 7.5 7.0 6.5 6.0 5.5 5.0 4.5 4.0 3.5 3.0 2.5 2.0 1.5 1.0 0.5 0.0
f1 (ppm)

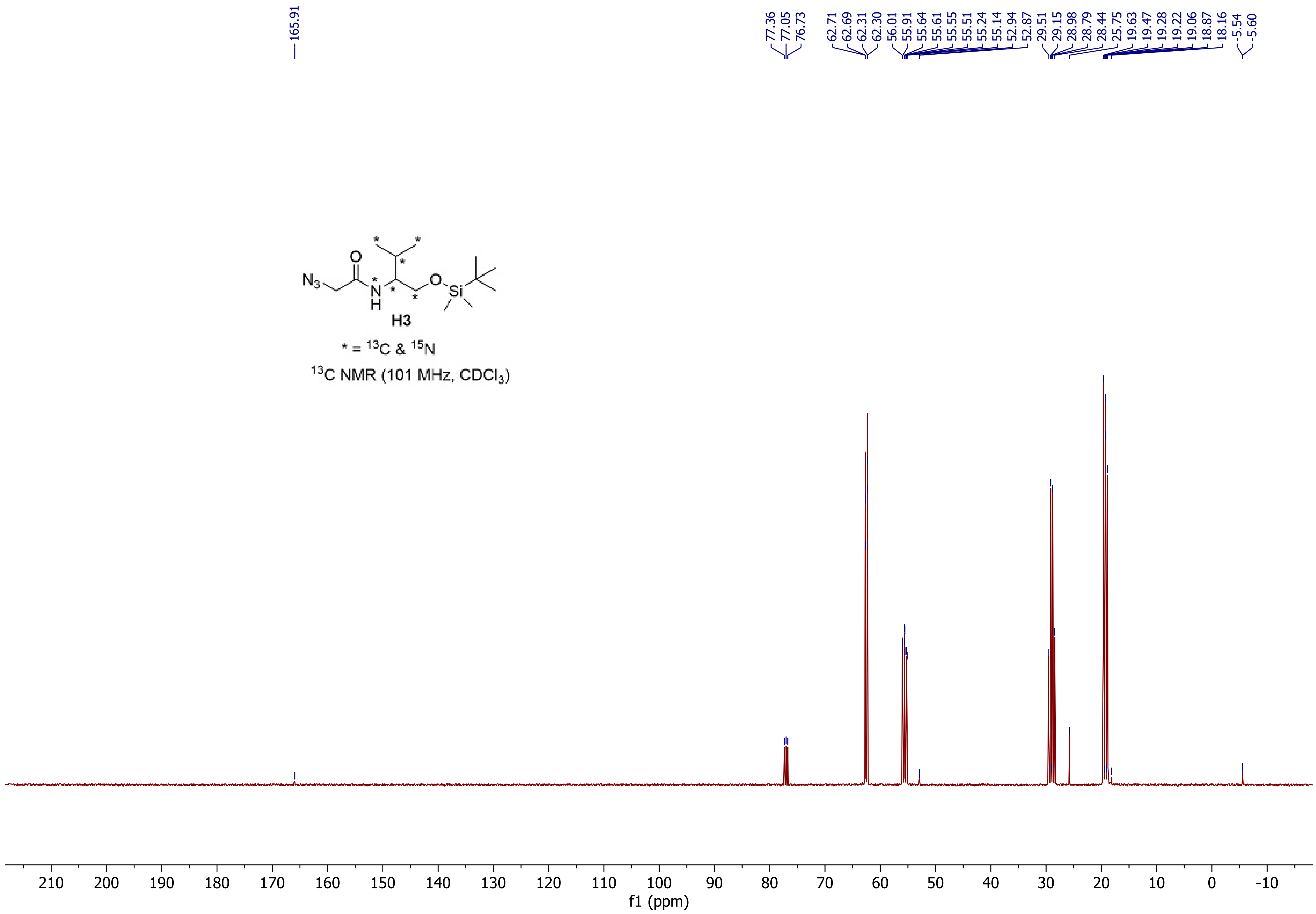
165.91
77.36
77.05
76.73
62.71
62.69
62.31
62.30
56.01
55.91
55.64
55.61
55.55
55.51
55.24
55.14
52.94
52.87
29.51
29.15
28.98
28.79
28.44
25.75
19.63
19.47
19.28
19.22
19.06
18.87
18.16
-5.54
-5.60
N3
O
N
H
O
Si
H3
* = 13C & 15N
13C NMR (101 MHz, CDCl3)
210
200
190
180
170
160
150
140
130
120
110
100
90
80
70
60
50
40
30
20
10
0
-10
f1 (ppm)

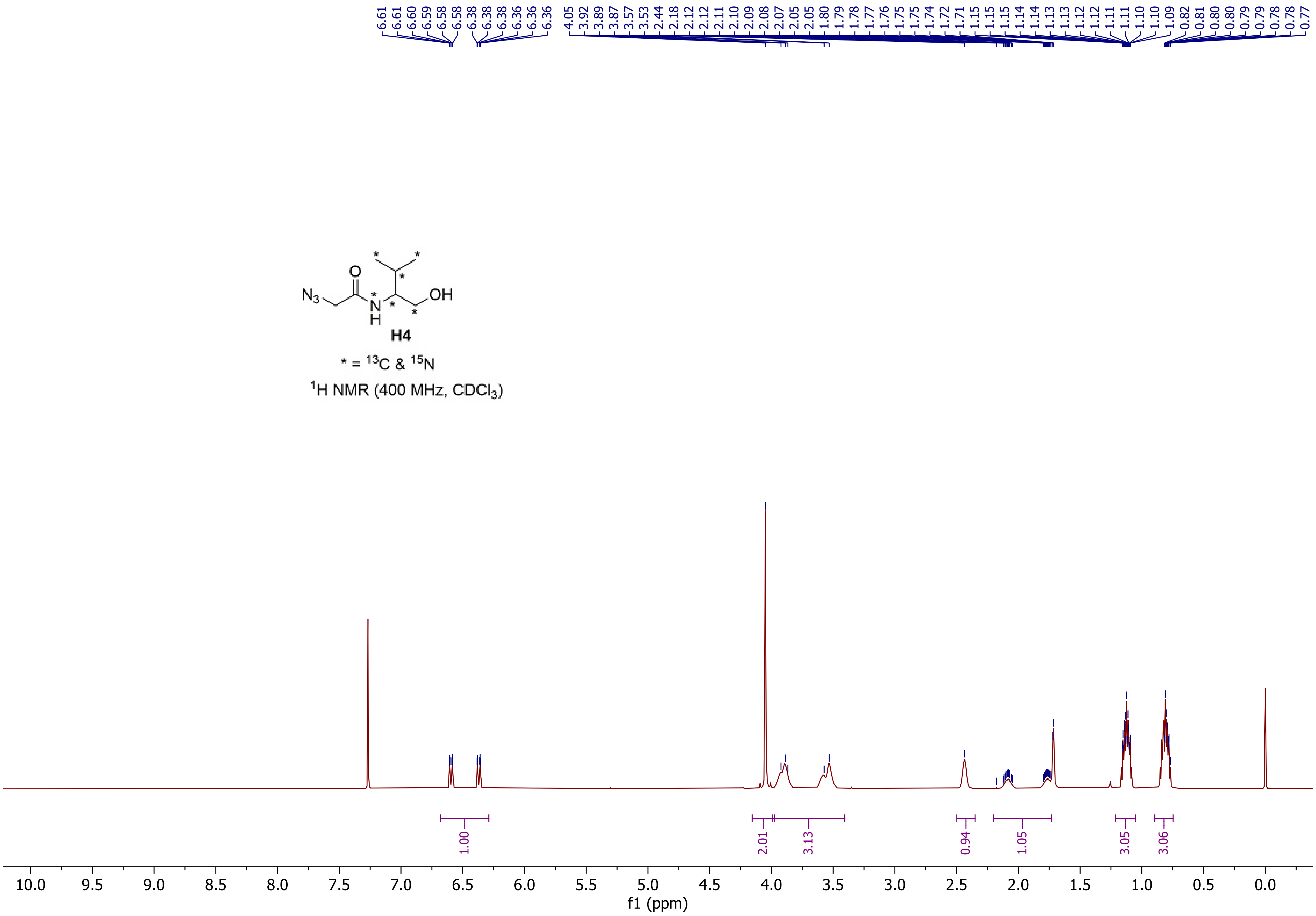

H4

* = ^{13}C & ^{15}N

^1H NMR (400 MHz, $CDCl_3$)

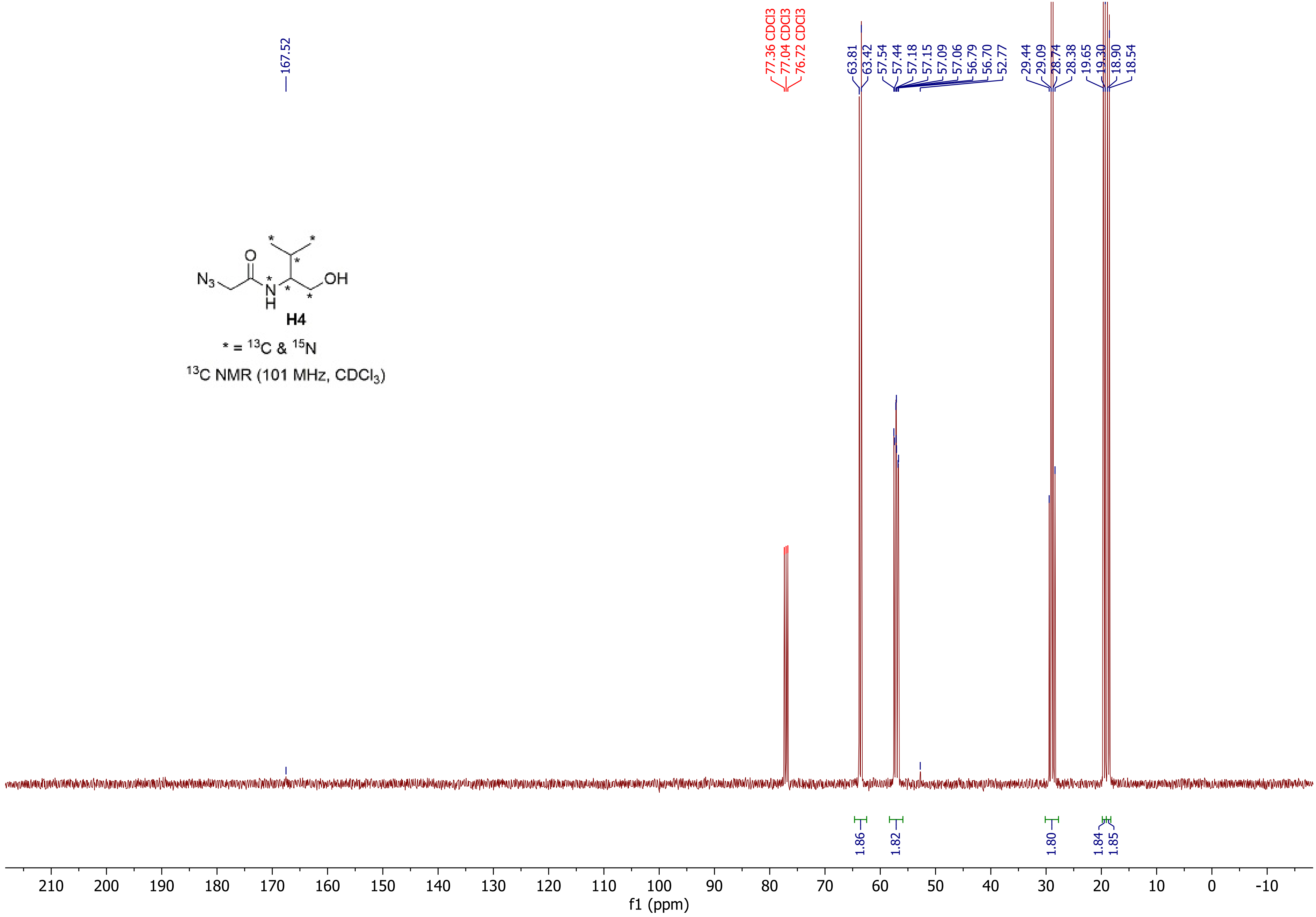
167.52
77.36 CDCl3
77.04 CDCl3
76.72 CDCl3
63.81
63.42
57.54
57.44
57.18
57.15
57.09
57.06
56.79
56.70
52.77
29.44
29.09
28.74
28.38
19.65
19.30
18.90
18.54
N3
O
N
H
OH
H4
* = 13C & 15N
13C NMR (101 MHz, CDCl3)
1.86
1.82
1.80
1.84
1.85
210 200 190 180 170 160 150 140 130 120 110 100 90 80 70 60 50 40 30 20 10 0 -10
f1 (ppm)

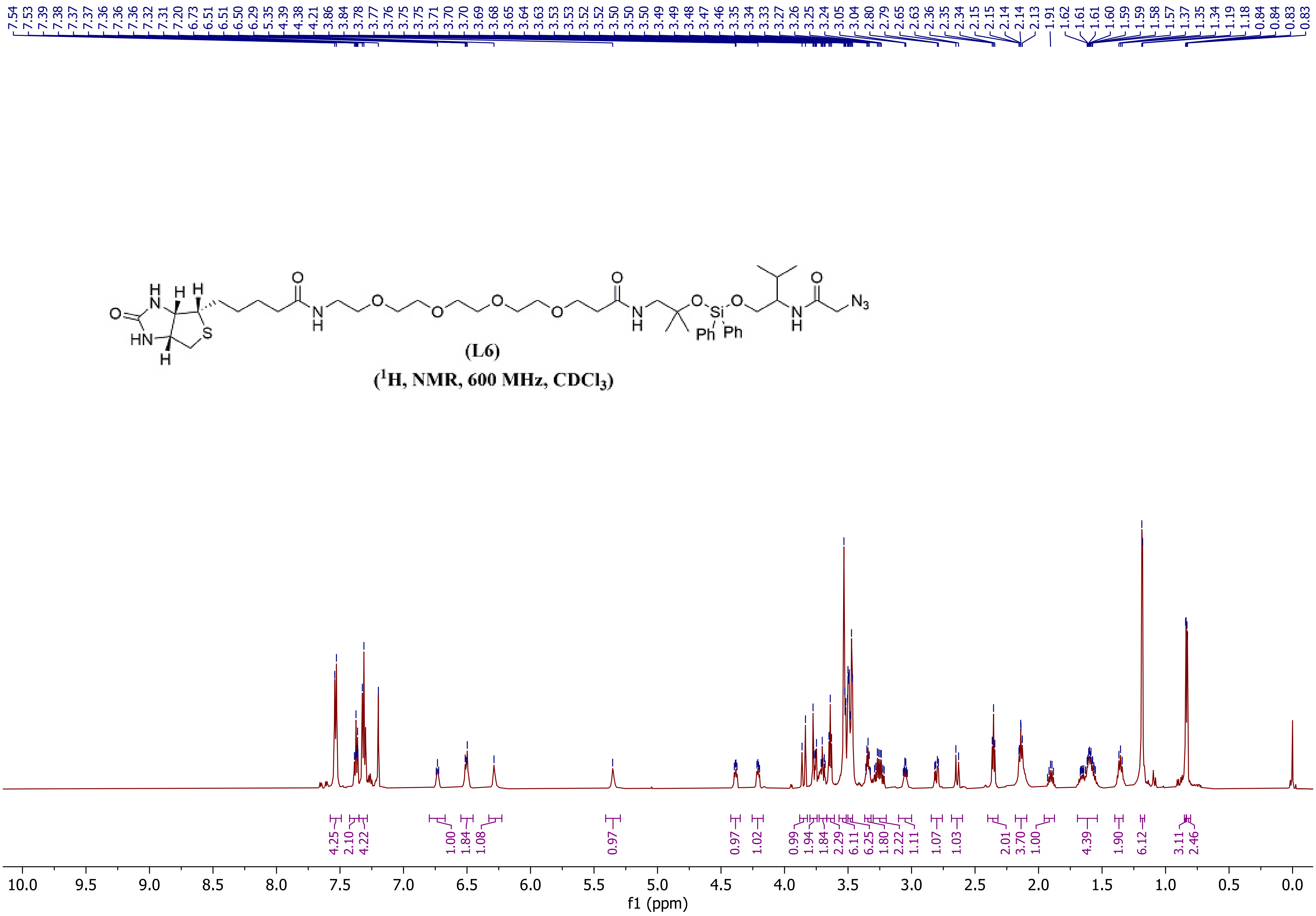

(L6)

(^{1}H, NMR, 600 MHz, $CDCl_3$)

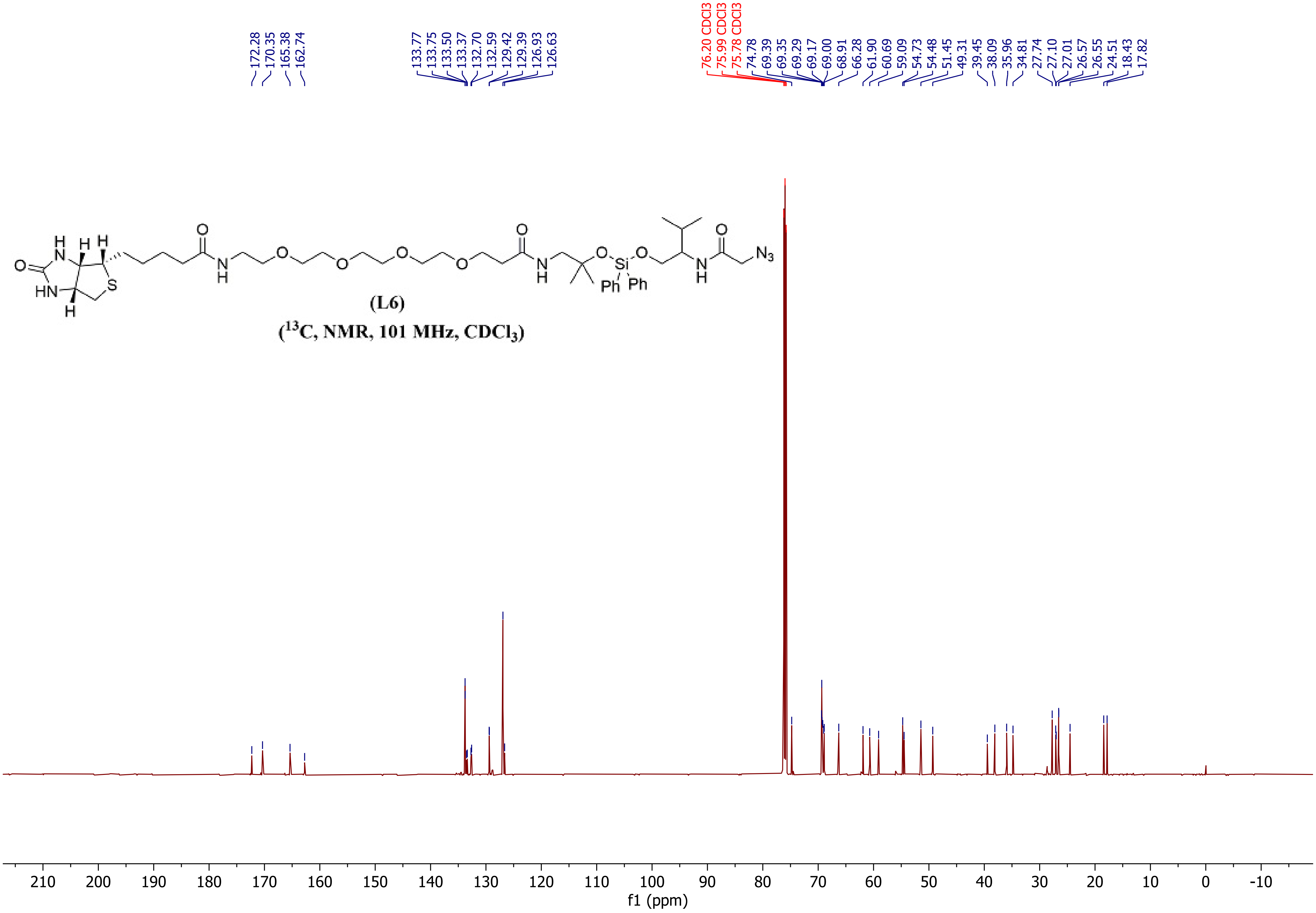
172.28
170.35
165.38
162.74
133.77
133.75
133.50
133.37
132.70
132.59
129.42
129.39
126.93
126.63
76.20 CDCl3
75.99 CDCl3
75.78 CDCl3
74.78
69.39
69.35
69.29
69.17
69.00
68.91
66.28
61.90
60.69
59.09
54.73
54.48
51.45
49.31
39.45
38.09
35.96
34.81
27.74
27.10
27.01
26.57
26.55
24.51
18.43
17.82
(L6)
(13C, NMR, 101 MHz, CDCl3)
f1 (ppm)

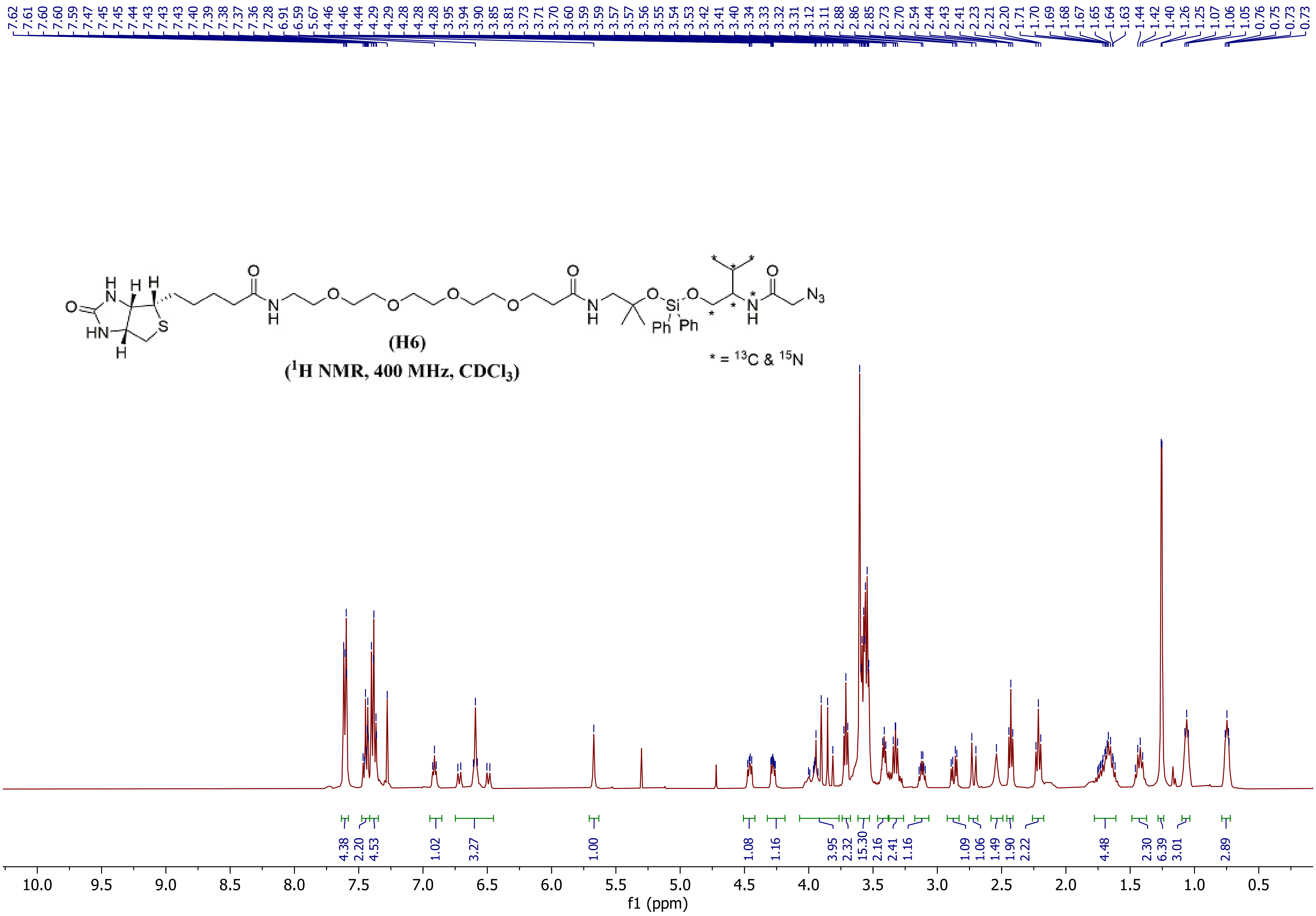

7.62 7.61 7.60 7.60 7.59 7.47 7.45 7.45 7.44 7.43 7.43 7.43 7.40 7.39 7.38 7.37 7.36 7.28 6.91 6.59 5.67 4.46 4.46 4.44 4.29 4.29 4.28 4.28 4.28 3.95 3.94 3.90 3.85 3.81 3.73 3.71 3.70 3.60 3.59 3.59 3.57 3.57 3.56 3.55 3.54 3.53 3.42 3.41 3.40 3.34 3.33 3.32 3.31 3.12 3.11 2.88 2.86 2.85 2.73 2.70 2.54 2.44 2.43 2.41 2.23 2.21 2.20 1.71 1.70 1.69 1.68 1.67 1.65 1.64 1.63 1.44 1.42 1.40 1.26 1.25 1.07 1.06 1.05 0.76 0.75 0.73 0.73
(H6)
(^{1}H NMR, 400 MHz, CDCl$_3$)
* = ^{13}C & ^{15}N
4.38 2.20 4.53 1.02 3.27 1.00 1.08 1.16 3.95 2.32 15.30 2.16 2.41 1.16 1.09 1.06 1.49 1.90 2.22 4.48 2.30 6.39 3.01 2.89
10.0 9.5 9.0 8.5 8.0 7.5 7.0 6.5 6.0 5.5 5.0 4.5 4.0 3.5 3.0 2.5 2.0 1.5 1.0 0.5
f1 (ppm)

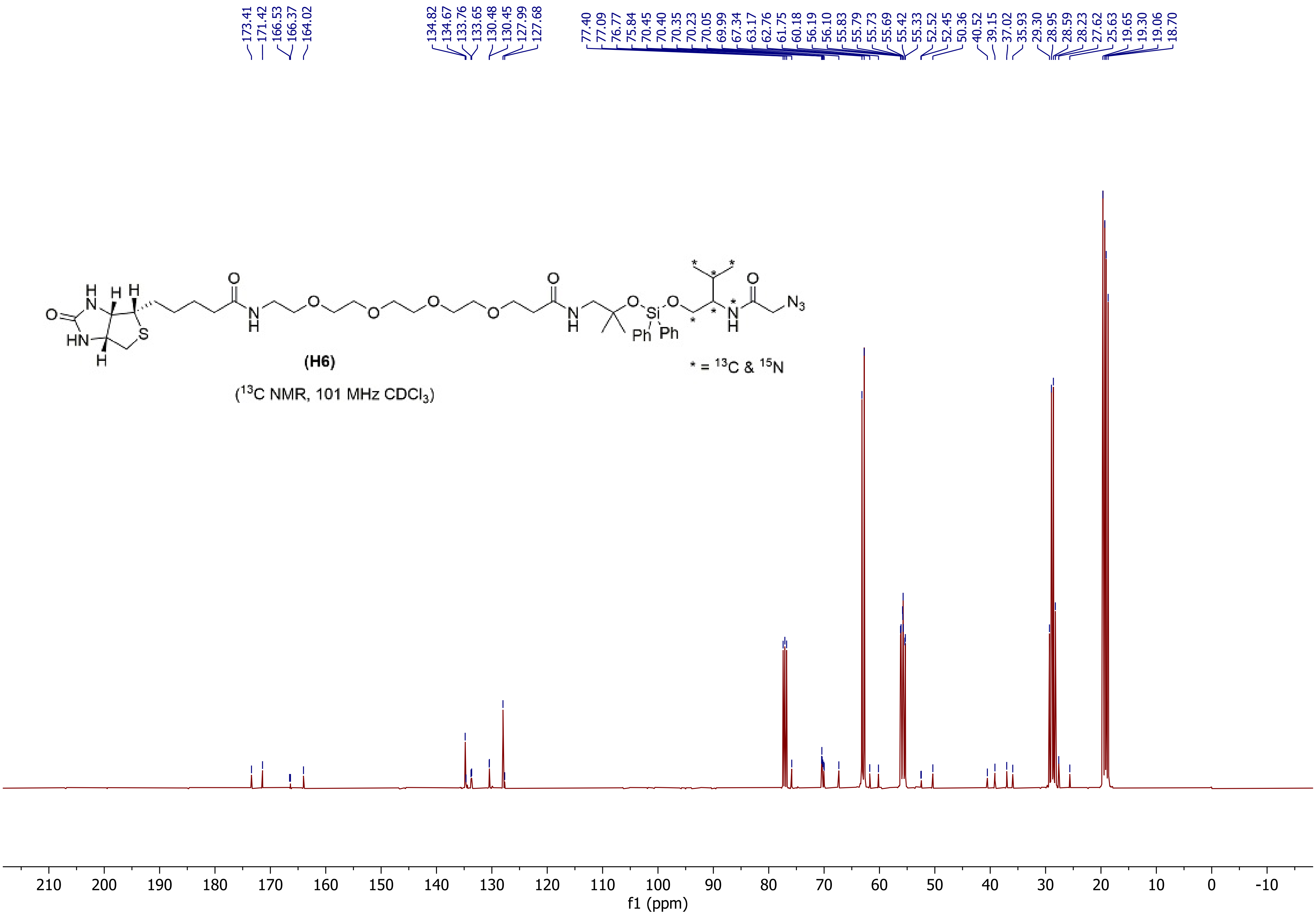
173.41
171.42
166.53
166.37
164.02
134.82
134.67
133.76
133.65
130.48
130.45
127.99
127.68
77.40
77.09
76.77
75.84
70.45
70.40
70.35
70.23
70.05
69.99
67.34
63.17
62.76
61.75
60.18
56.19
56.10
55.83
55.79
55.73
55.69
55.42
55.33
52.52
52.45
50.36
40.52
39.15
37.02
35.93
29.30
28.95
28.59
28.23
27.62
25.63
19.65
19.30
19.06
18.70
(H6)
(13C NMR, 101 MHz CDCl3)
* = 13C & 15N
210 200 190 180 170 160 150 140 130 120 110 100 90 80 70 60 50 40 30 20 10 0 -10
f1 (ppm)

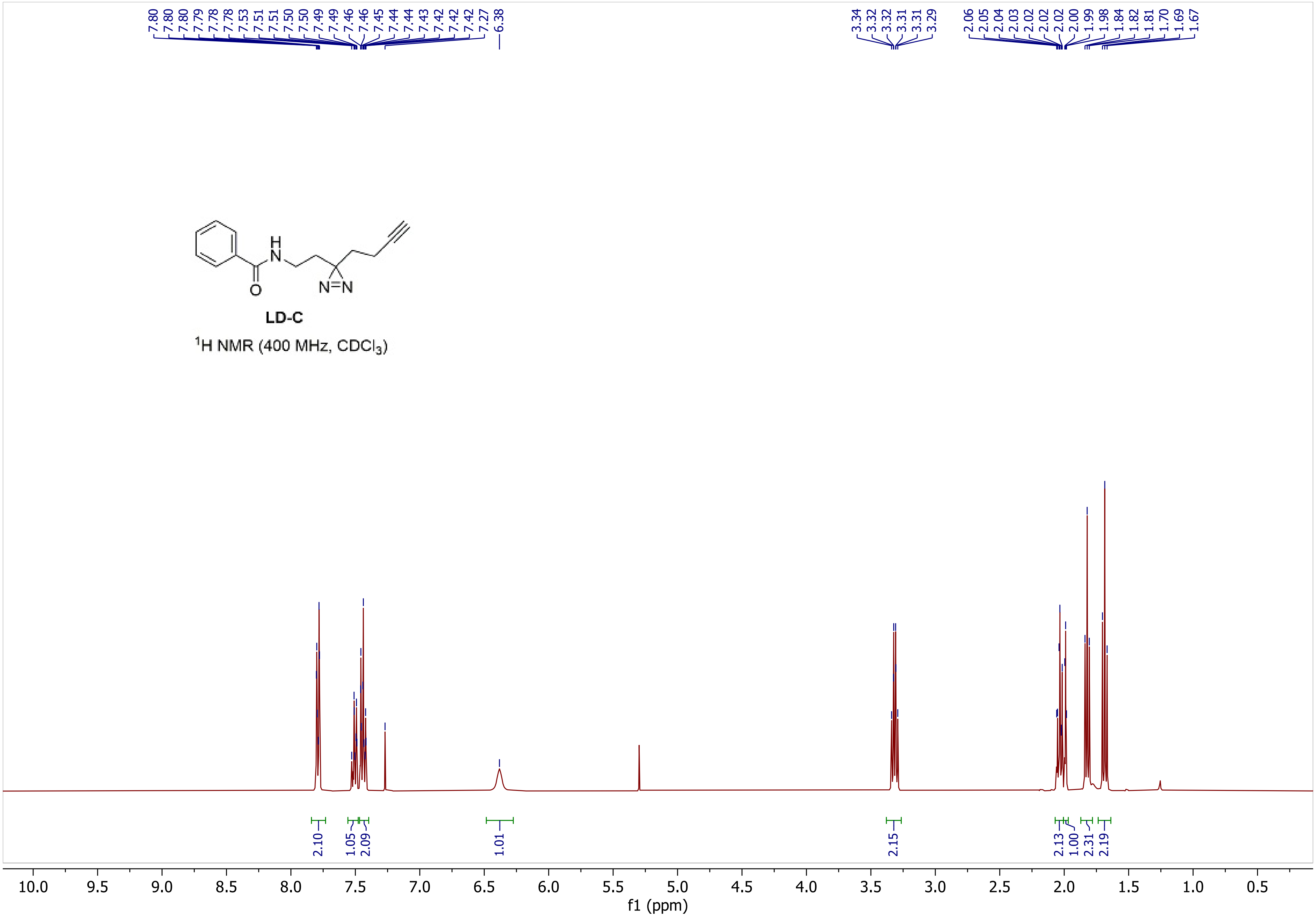
7.80 7.80 7.80 7.79 7.78 7.78 7.53 7.51 7.51 7.50 7.50 7.49 7.49 7.46 7.46 7.45 7.44 7.44 7.43 7.42 7.42 7.42 7.27 6.38
3.34 3.32 3.32 3.31 3.31 3.29
2.06 2.05 2.04 2.03 2.02 2.02 2.02 2.00 1.99 1.98 1.84 1.82 1.81 1.70 1.69 1.67
LD-C
^{1}H NMR (400 MHz, $CDCl_3$)
2.10 1.05 2.09 1.01 2.15 2.13 1.00 2.31 2.19
10.0 9.5 9.0 8.5 8.0 7.5 7.0 6.5 6.0 5.5 5.0 4.5 4.0 3.5 3.0 2.5 2.0 1.5 1.0 0.5
f1 (ppm)

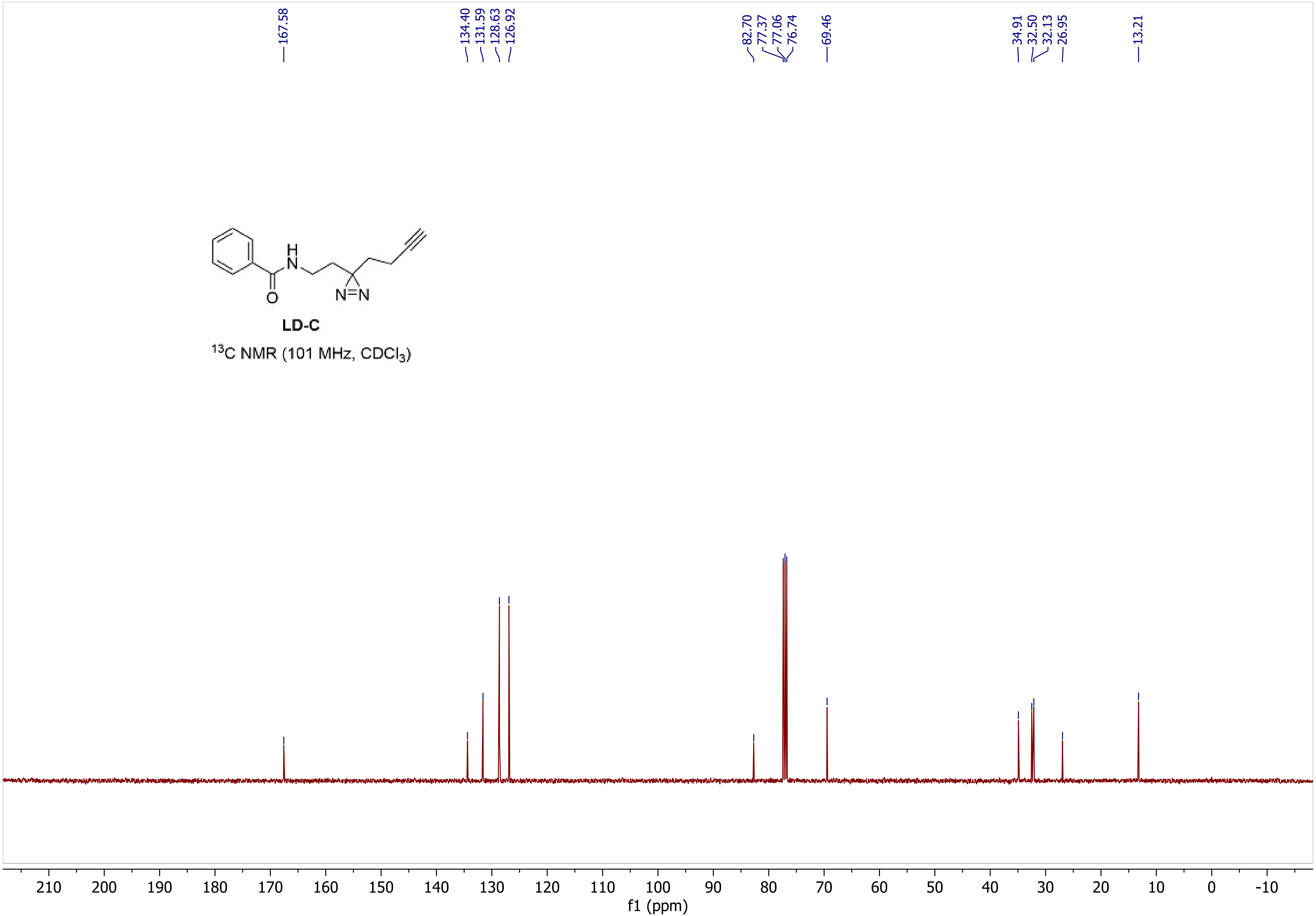

LD-C

^{13}C NMR (101 MHz, $CDCl_3$)

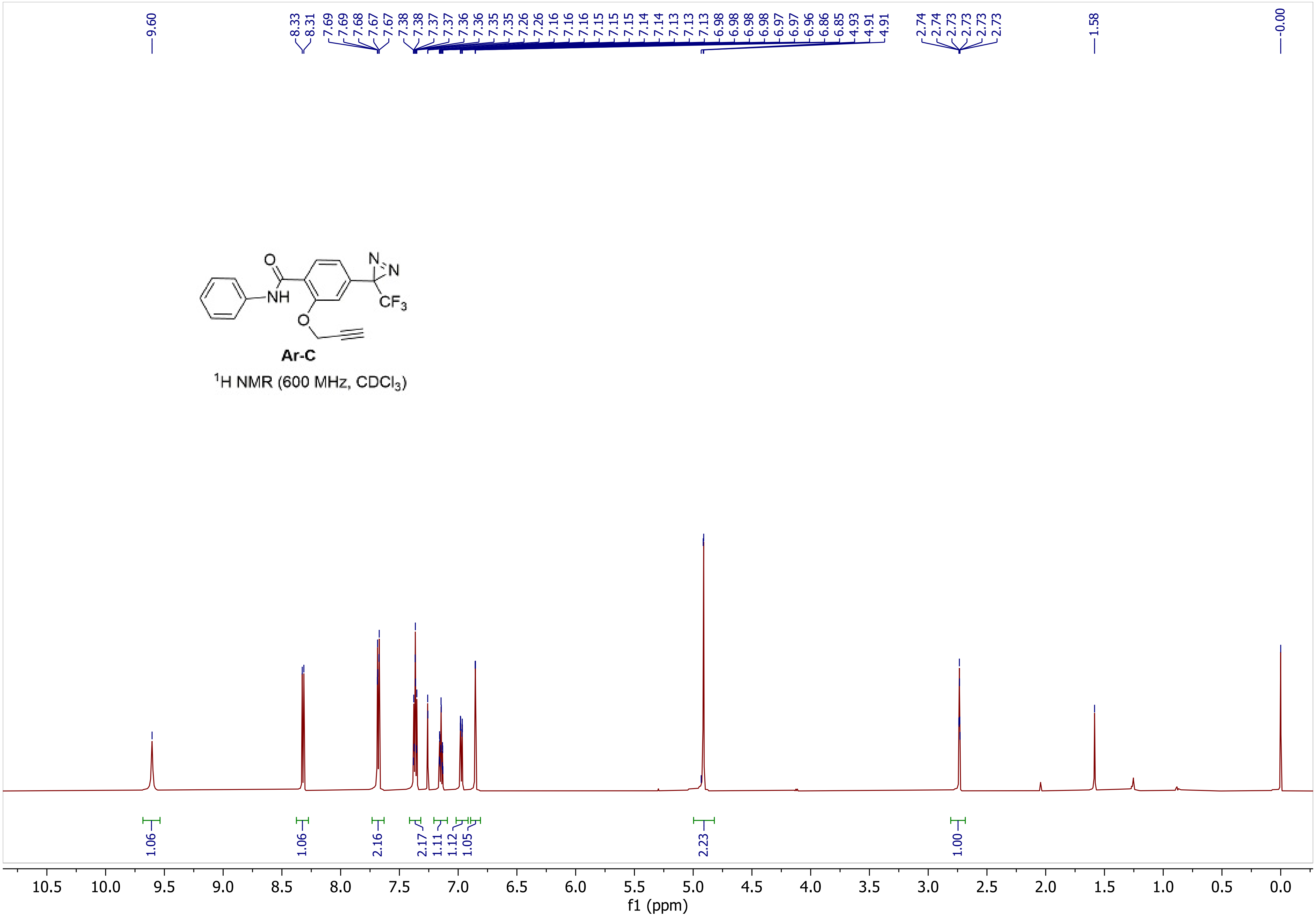
9.60
8.33
8.31
7.69
7.69
7.68
7.67
7.67
7.38
7.38
7.37
7.37
7.36
7.36
7.35
7.35
7.26
7.26
7.16
7.16
7.16
7.15
7.15
7.15
7.14
7.14
7.13
7.13
7.13
6.98
6.98
6.98
6.98
6.97
6.97
6.96
6.86
6.85
4.93
4.91
4.91
2.74
2.74
2.73
2.73
2.73
2.73
1.58
0.00
O
NH
N
N
CF3
O
Ar-C
1H NMR (600 MHz, CDCl3)
1.06
1.06
2.16
2.17
1.11
1.12
1.05
2.23
1.00
10.5
10.0
9.5
9.0
8.5
8.0
7.5
7.0
6.5
6.0
5.5
5.0
4.5
4.0
3.5
3.0
2.5
2.0
1.5
1.0
0.5
0.0
f1 (ppm)

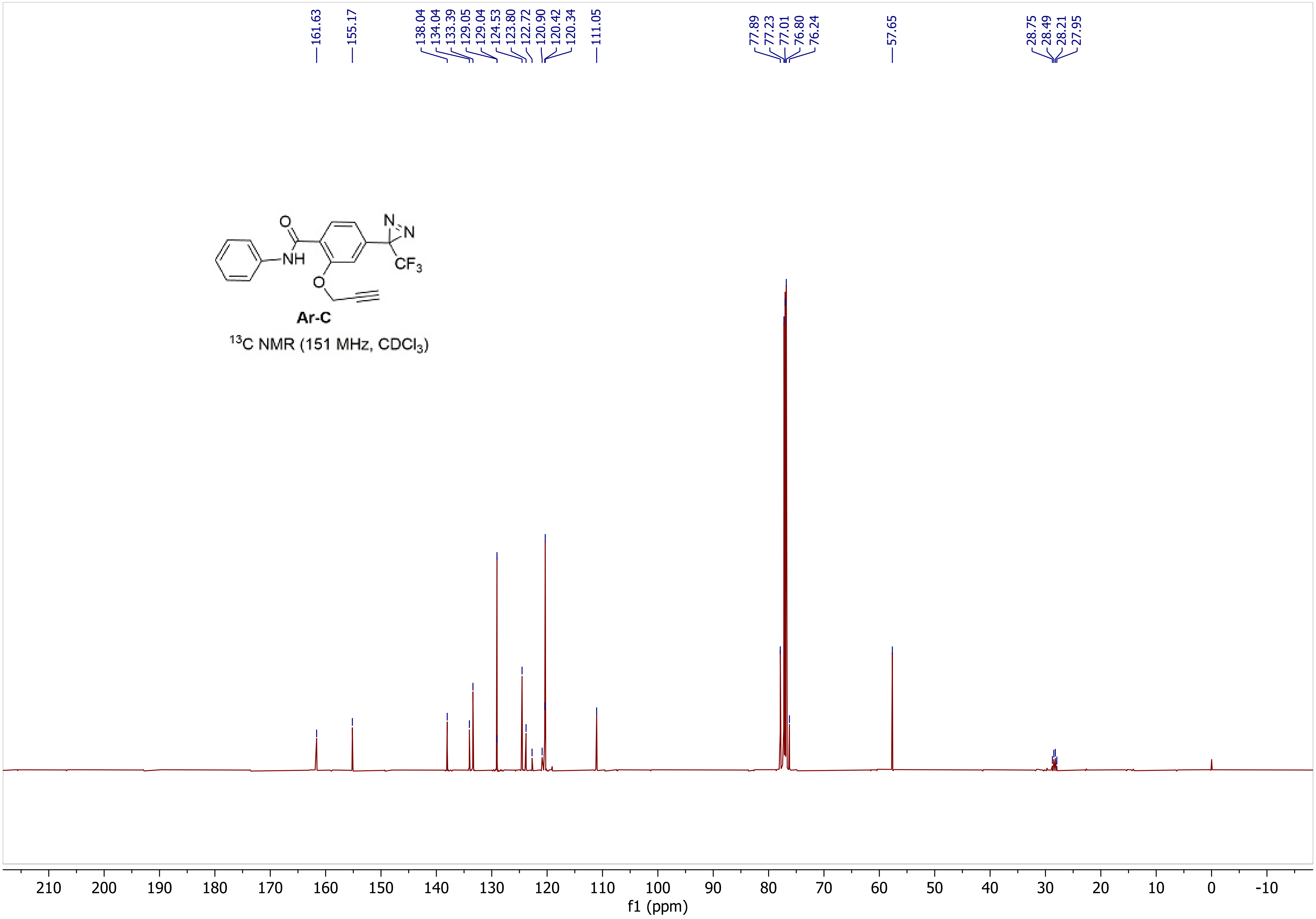
161.63
155.17
138.04
134.04
133.39
129.05
129.04
124.53
123.80
122.72
120.90
120.42
120.34
111.05
77.89
77.23
77.01
76.80
76.24
57.65
28.75
28.49
28.21
27.95
O
NH
N
N
CF3
O
Ar-C
13C NMR (151 MHz, CDCl3)
210 200 190 180 170 160 150 140 130 120 110 100 90 80 70 60 50 40 30 20 10 0 -10
f1 (ppm)

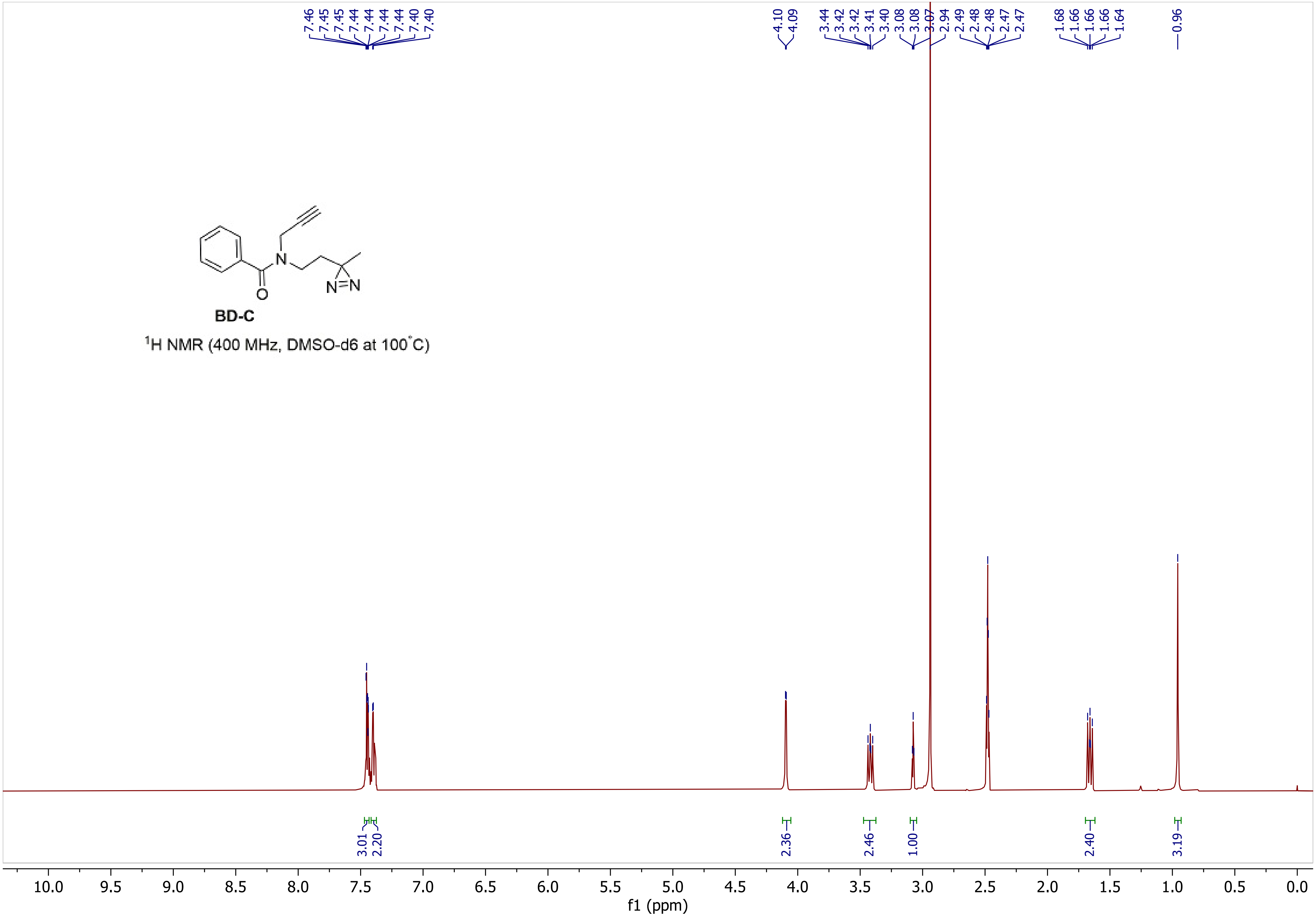
BD-C
¹H NMR (400 MHz, DMSO-d6 at 100˚C)
7.46 7.45 7.45 7.44 7.44 7.44 7.44 7.40 7.40
4.10 4.09
3.44 3.42 3.42 3.41 3.40 3.08 3.08 3.07 2.94 2.49 2.48 2.48 2.47 2.47
1.68 1.66 1.66 1.66 1.64
0.96
3.01 2.20 2.36 2.46 1.00 2.40 3.19
10.0 9.5 9.0 8.5 8.0 7.5 7.0 6.5 6.0 5.5 5.0 4.5 4.0 3.5 3.0 2.5 2.0 1.5 1.0 0.5 0.0
f1 (ppm)

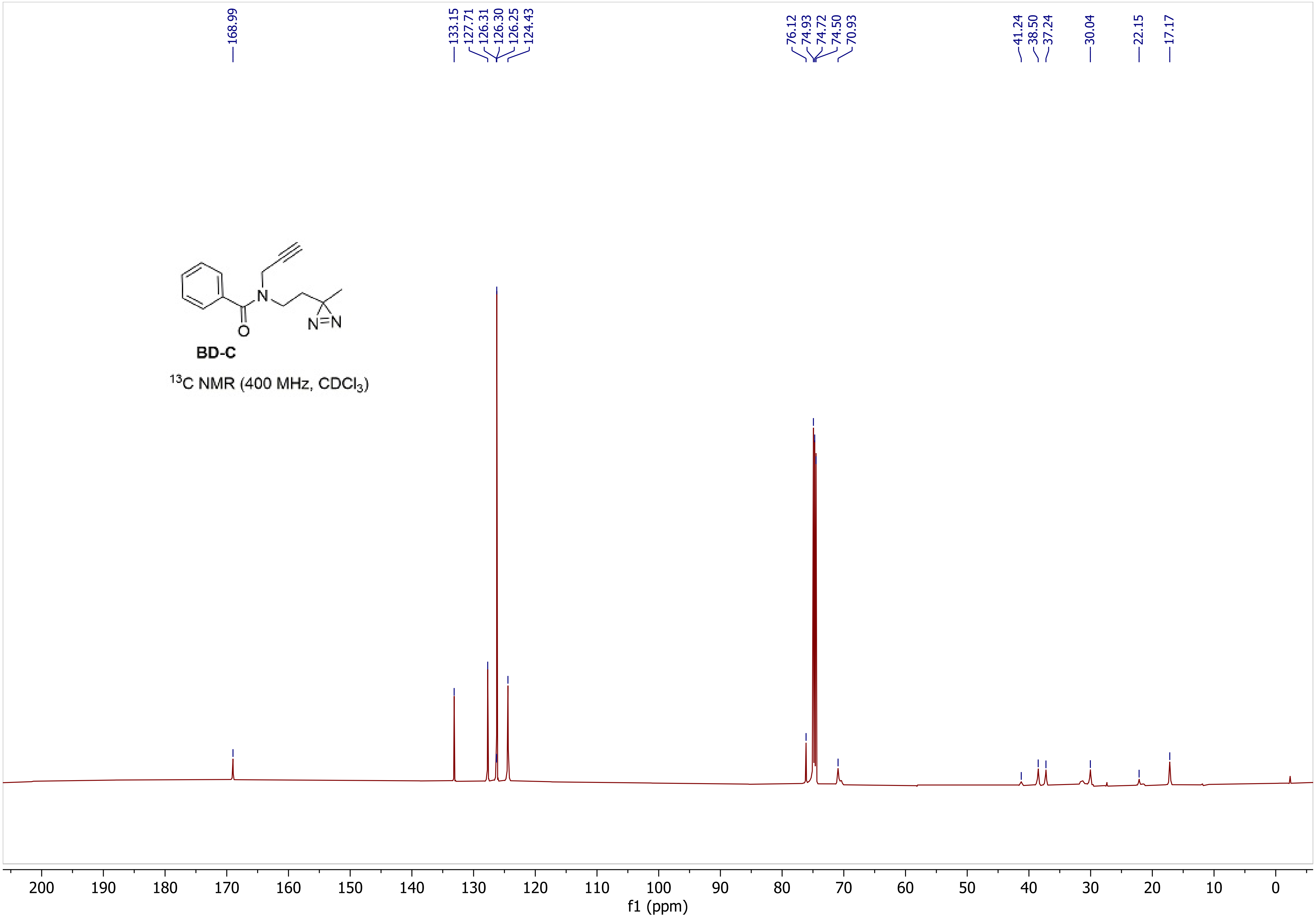
168.99
133.15
127.71
126.31
126.30
126.25
124.43
76.12
74.93
74.72
74.50
70.93
41.24
38.50
37.24
30.04
22.15
17.17
O
N
N=N
BD-C
13C NMR (400 MHz, CDCl3)
200
190
180
170
160
150
140
130
120
110
100
90
80
70
60
50
40
30
20
10
0
f1 (ppm)

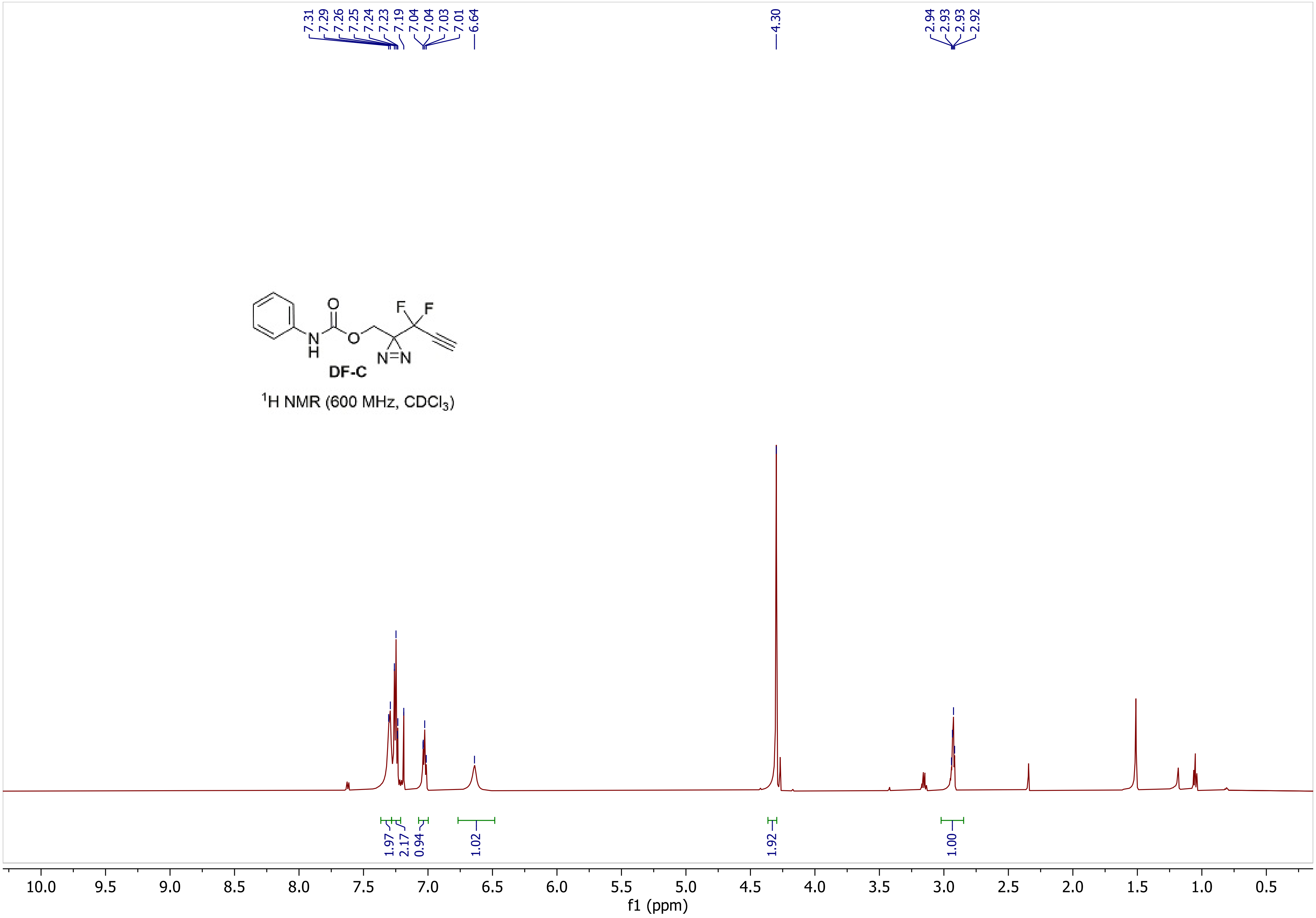
7.31
7.29
7.26
7.25
7.24
7.23
7.19
7.04
7.04
7.03
7.01
6.64
4.30
2.94
2.93
2.93
2.92
DF-C
^{1}H NMR (600 MHz, $CDCl_3$)
1.97
2.17
0.94
1.02
1.92
1.00
10.0
9.5
9.0
8.5
8.0
7.5
7.0
6.5
6.0
5.5
5.0
4.5
4.0
3.5
3.0
2.5
2.0
1.5
1.0
0.5
f1 (ppm)

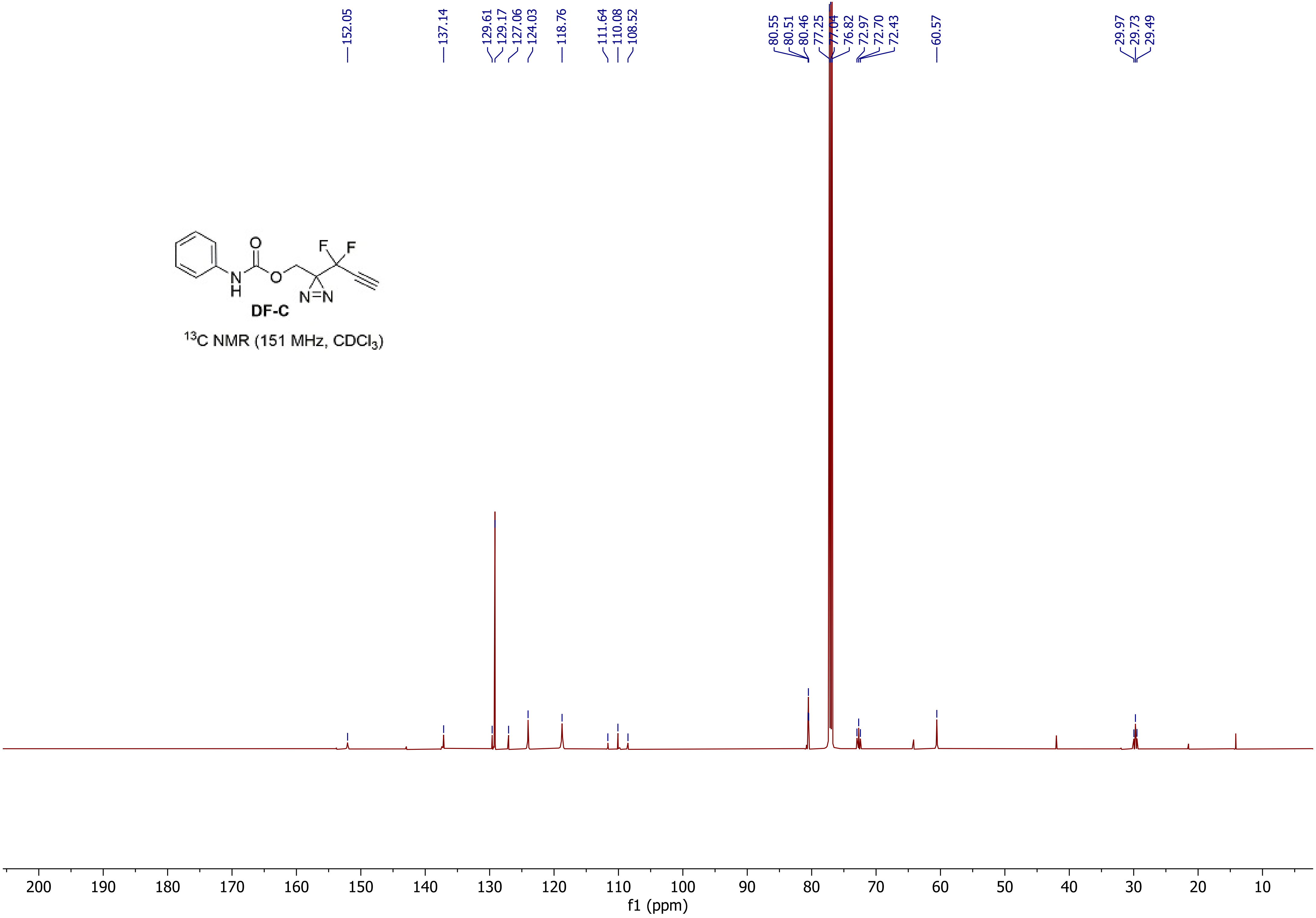
152.05
137.14
129.61
129.17
127.06
124.03
118.76
111.64
110.08
108.52
80.55
80.51
80.46
77.25
77.04
76.82
72.97
72.70
72.43
60.57
29.97
29.73
29.49
O
F
F
N
H
O
N=N
DF-C
^{13}C NMR (151 MHz, $CDCl_3$)
200
190
180
170
160
150
140
130
120
110
100
90
80
70
60
50
40
30
20
10
f1 (ppm)

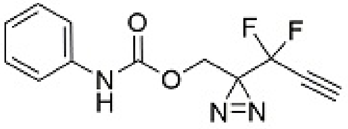

DF-C

^{19}F NMR (565 MHz, $CDCl_3$)

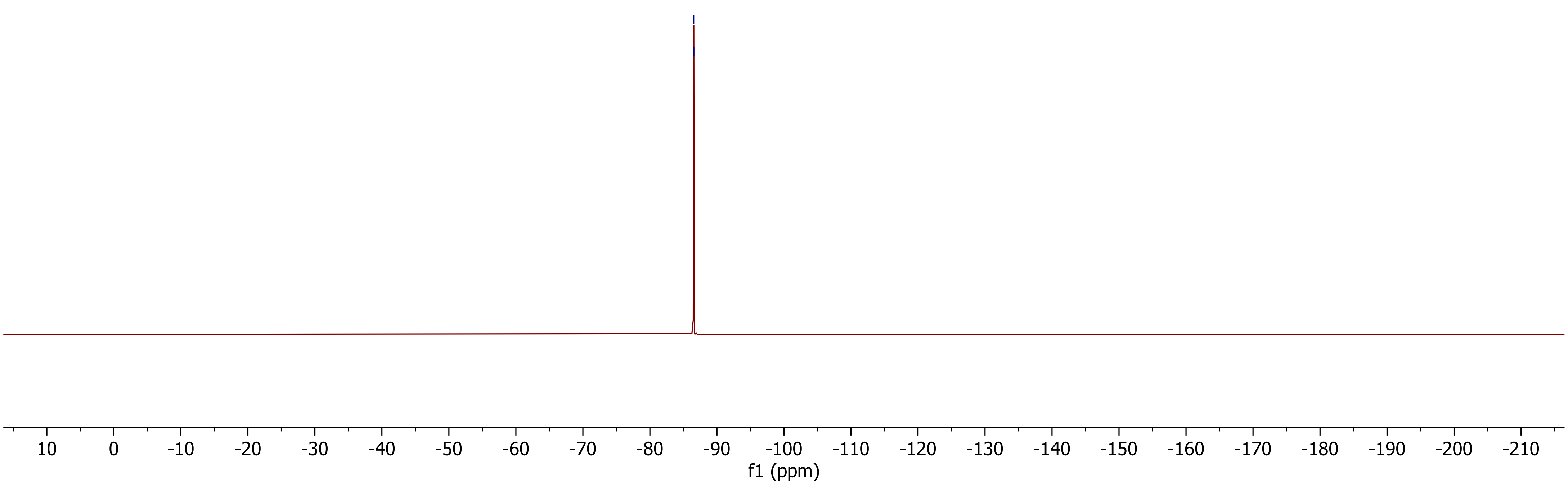

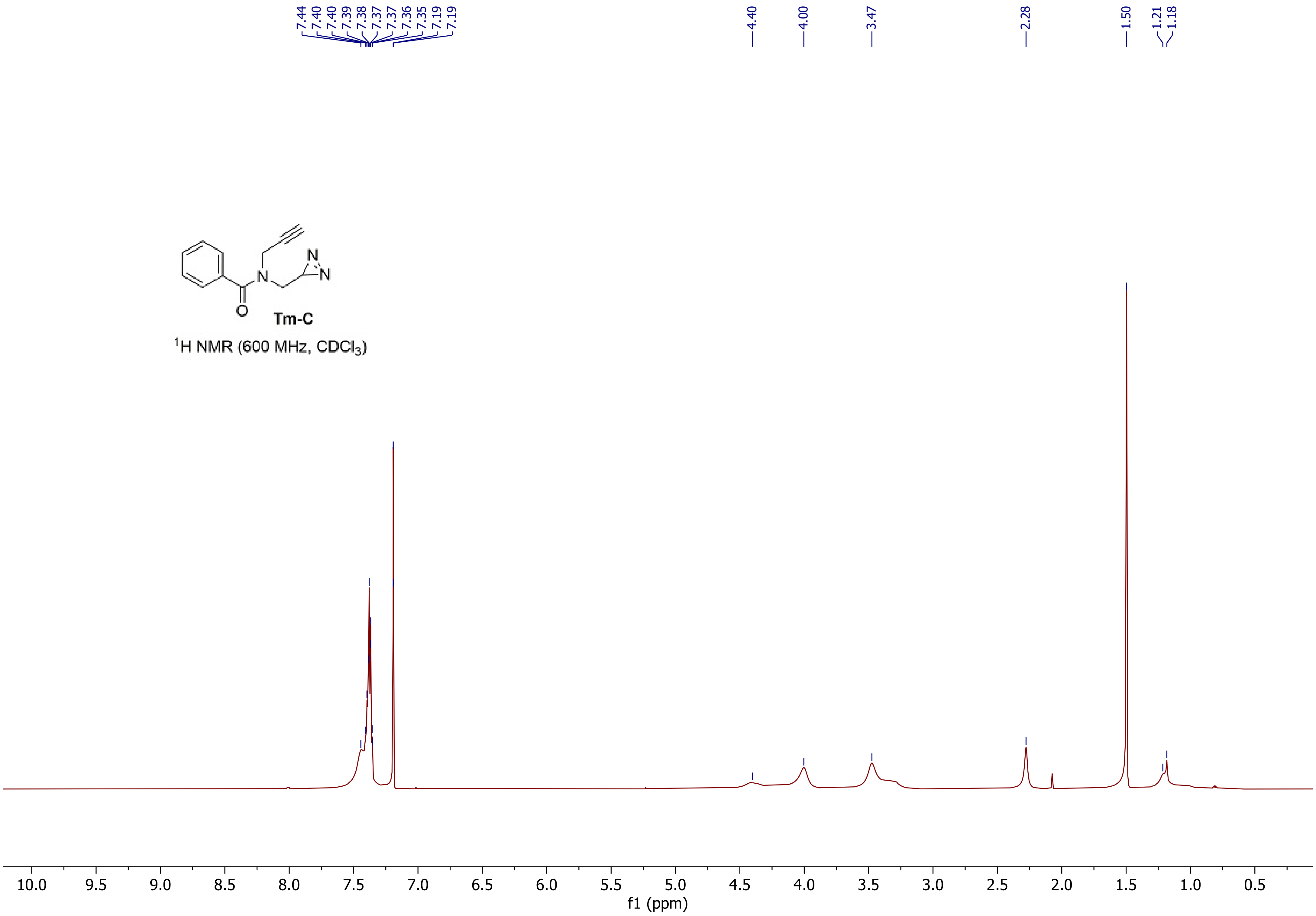

7.44
7.40
7.40
7.39
7.38
7.37
7.37
7.36
7.35
7.19
7.19
4.40
4.00
3.47
2.28
1.50
1.21
1.18
Tm-C
1H NMR (600 MHz, CDCl3)
10.0
9.5
9.0
8.5
8.0
7.5
7.0
6.5
6.0
5.5
5.0
4.5
4.0
3.5
3.0
2.5
2.0
1.5
1.0
0.5
f1 (ppm)

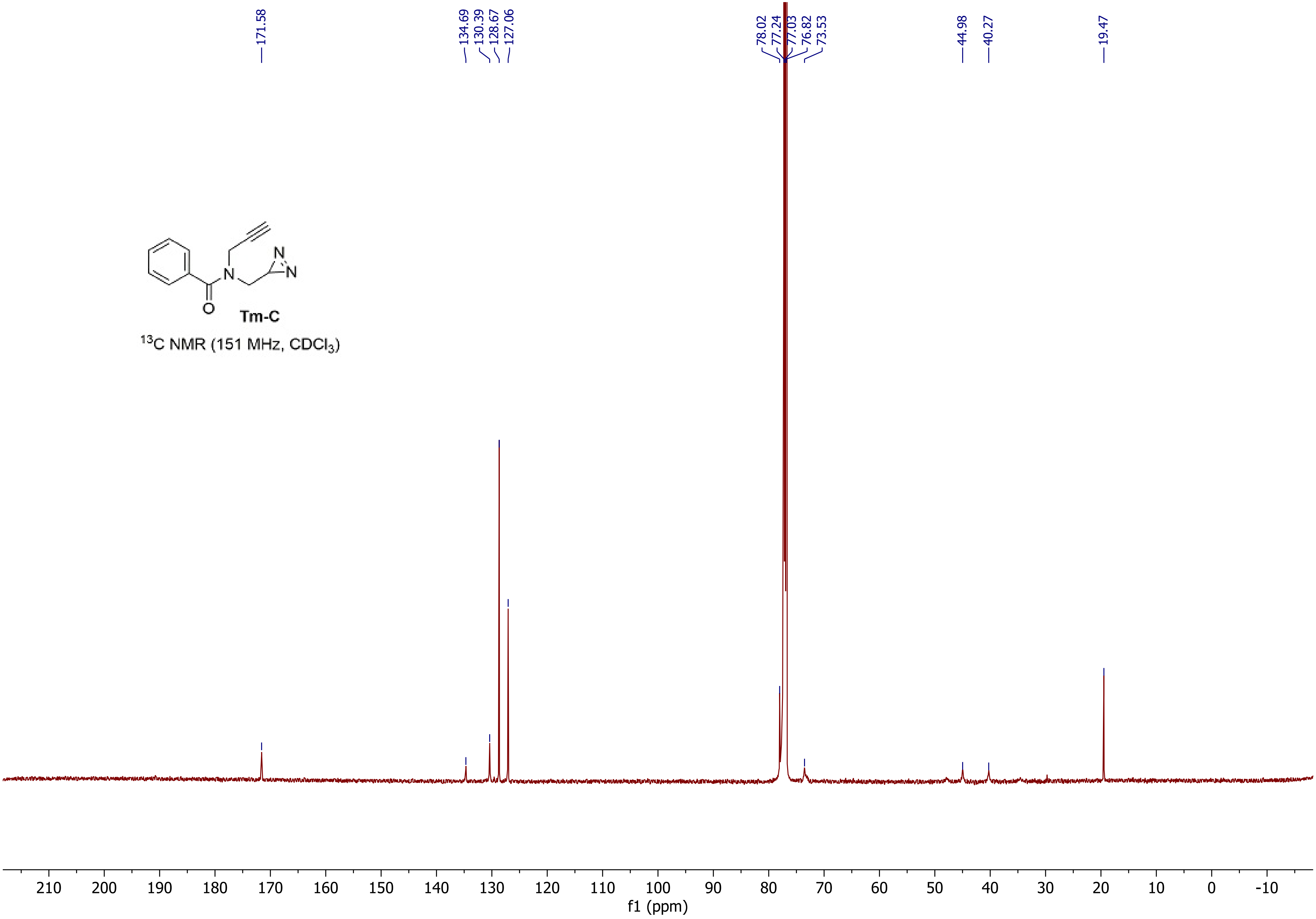
171.58
134.69
130.39
128.67
127.06
78.02
77.24
77.03
76.82
73.53
44.98
40.27
19.47
N
N
N
O
Tm-C
^{13}C NMR (151 MHz, $CDCl_3$)
210 200 190 180 170 160 150 140 130 120 110 100 90 80 70 60 50 40 30 20 10 0 -10
f1 (ppm)

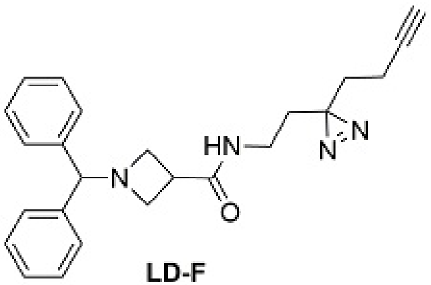

LD-F

^{1}H NMR (400 MHz, $CDCl_3$)

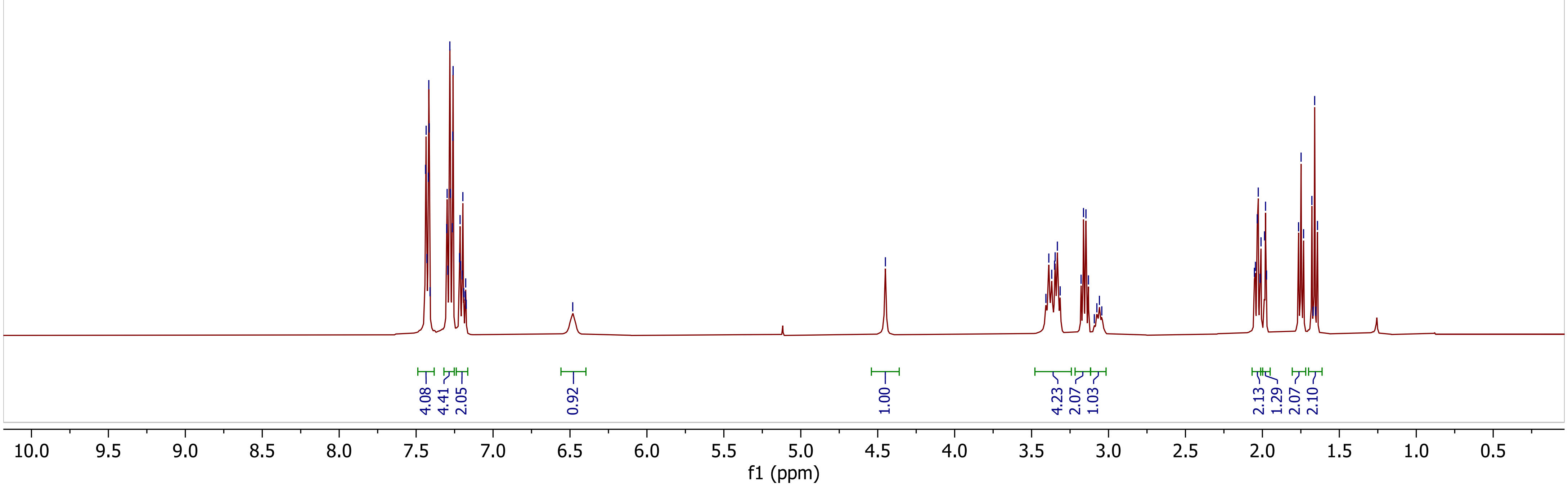

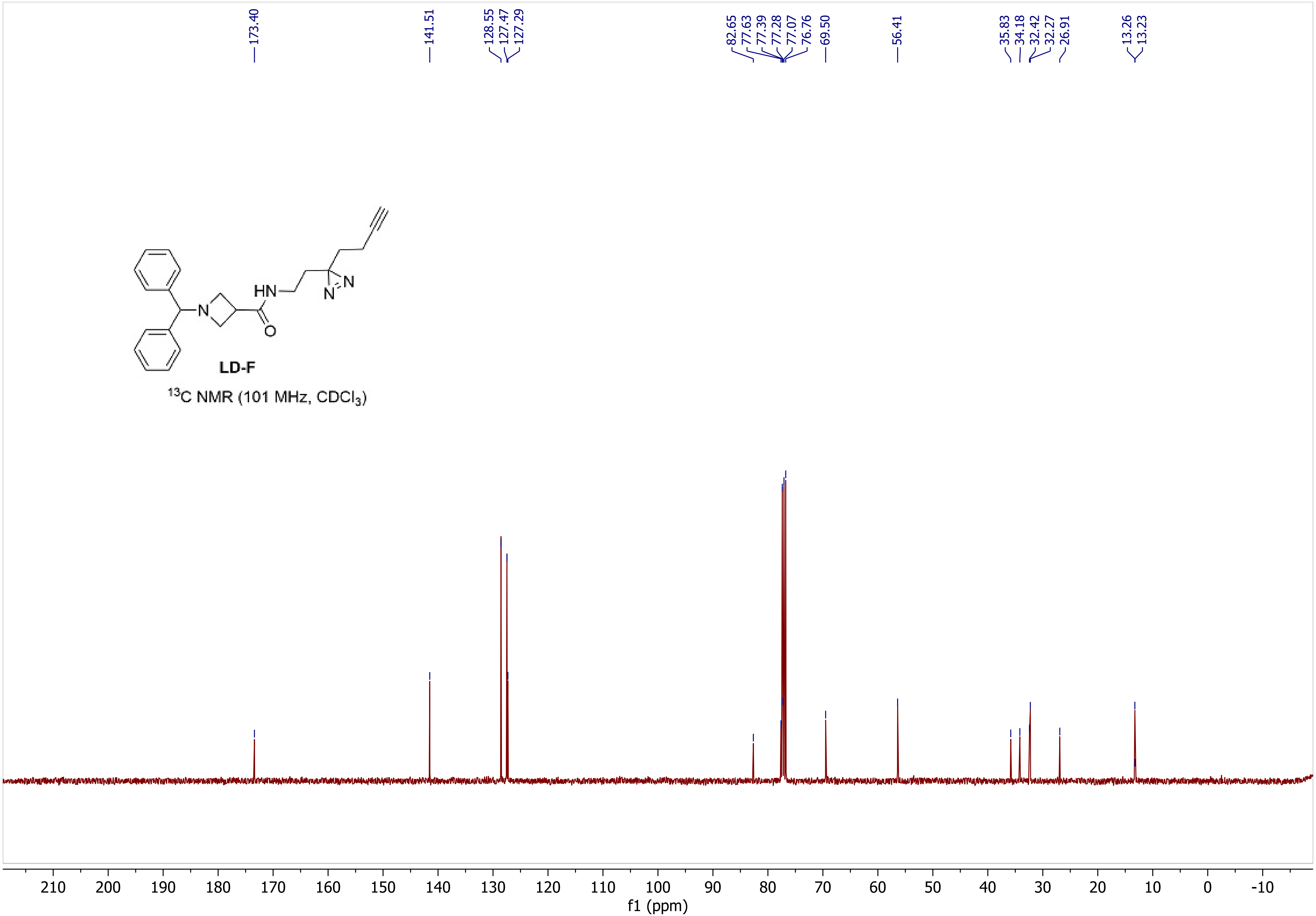
173.40
141.51
128.55
127.47
127.29
82.65
77.63
77.39
77.28
77.07
76.76
69.50
56.41
35.83
34.18
32.42
32.27
26.91
13.26
13.23
HN
N
N
N
O
LD-F
13C NMR (101 MHz, CDCl3)
210 200 190 180 170 160 150 140 130 120 110 100 90 80 70 60 50 40 30 20 10 0 -10
f1 (ppm)

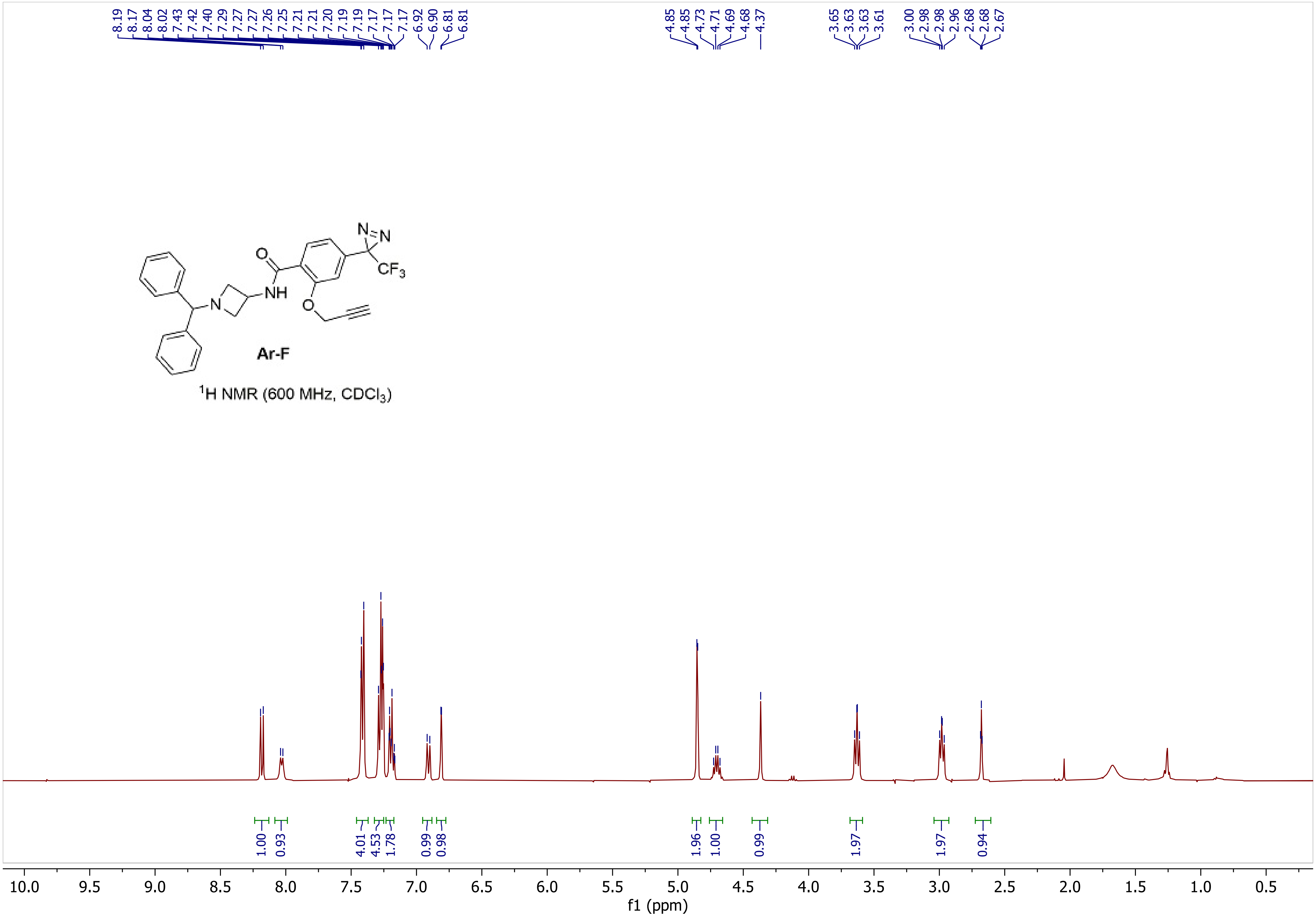

8.19 8.17 8.04 8.02 7.43 7.42 7.40 7.29 7.27 7.27 7.26 7.25 7.21 7.21 7.20 7.19 7.19 7.17 7.17 7.17 6.92 6.90 6.81 6.81
4.85 4.85 4.73 4.71 4.69 4.68 4.37
3.65 3.63 3.63 3.61
3.00 2.98 2.98 2.96 2.68 2.68 2.67
N=N
O
CF3
NH
N
O
Ar-F
1H NMR (600 MHz, CDCl3)
1.00 0.93 4.01 4.53 1.78 0.99 0.98 1.96 1.00 0.99 1.97 1.97 0.94
10.0 9.5 9.0 8.5 8.0 7.5 7.0 6.5 6.0 5.5 5.0 4.5 4.0 3.5 3.0 2.5 2.0 1.5 1.0 0.5
f1 (ppm)

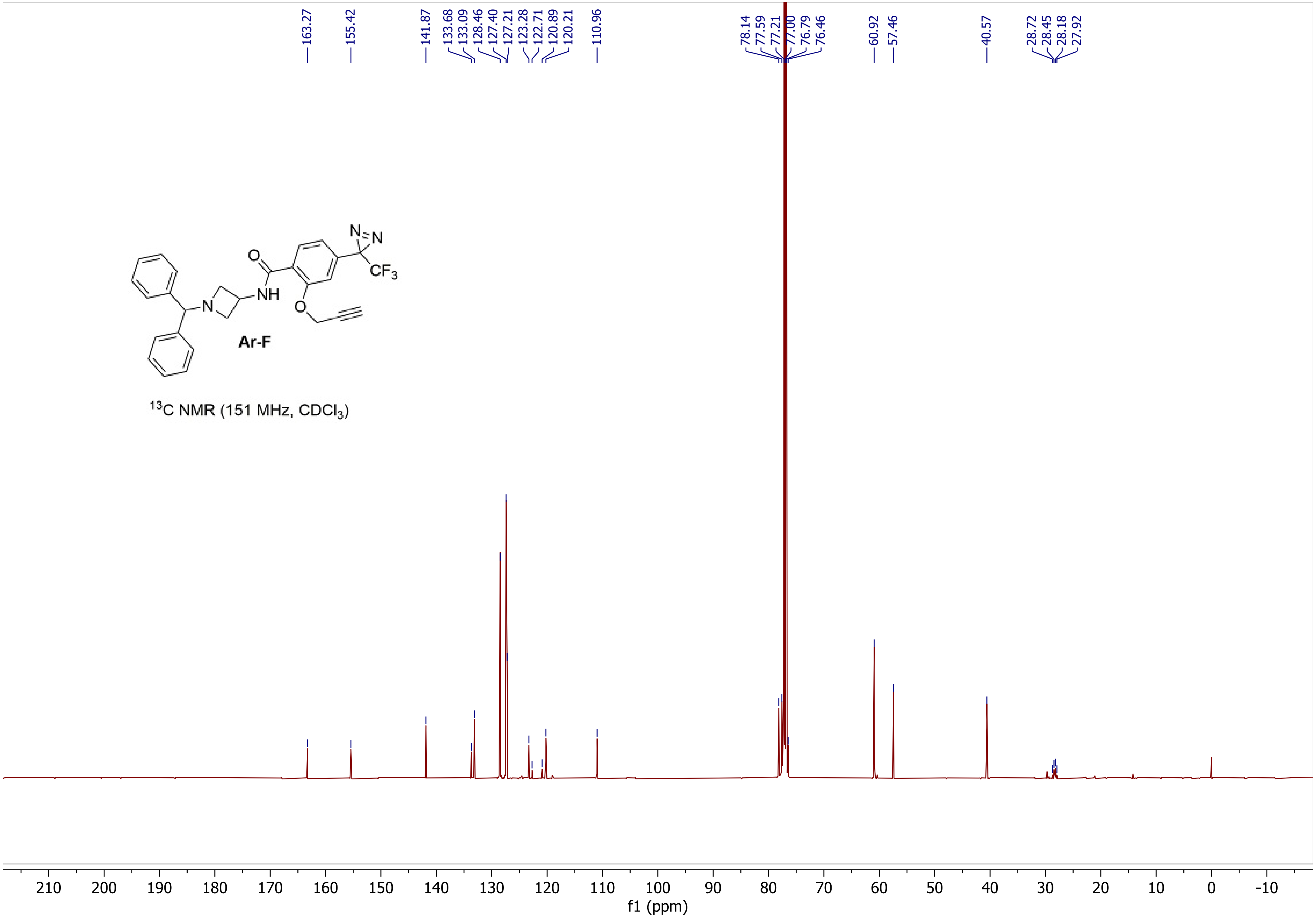
163.27
155.42
141.87
133.68
133.09
128.46
127.40
127.21
123.28
122.71
120.89
120.21
110.96
78.14
77.59
77.21
77.00
76.79
76.46
60.92
57.46
40.57
28.72
28.45
28.18
27.92
N
N
O
NH
N
O
CF3
Ar-F
13C NMR (151 MHz, CDCl3)
210 200 190 180 170 160 150 140 130 120 110 100 90 80 70 60 50 40 30 20 10 0 -10
f1 (ppm)

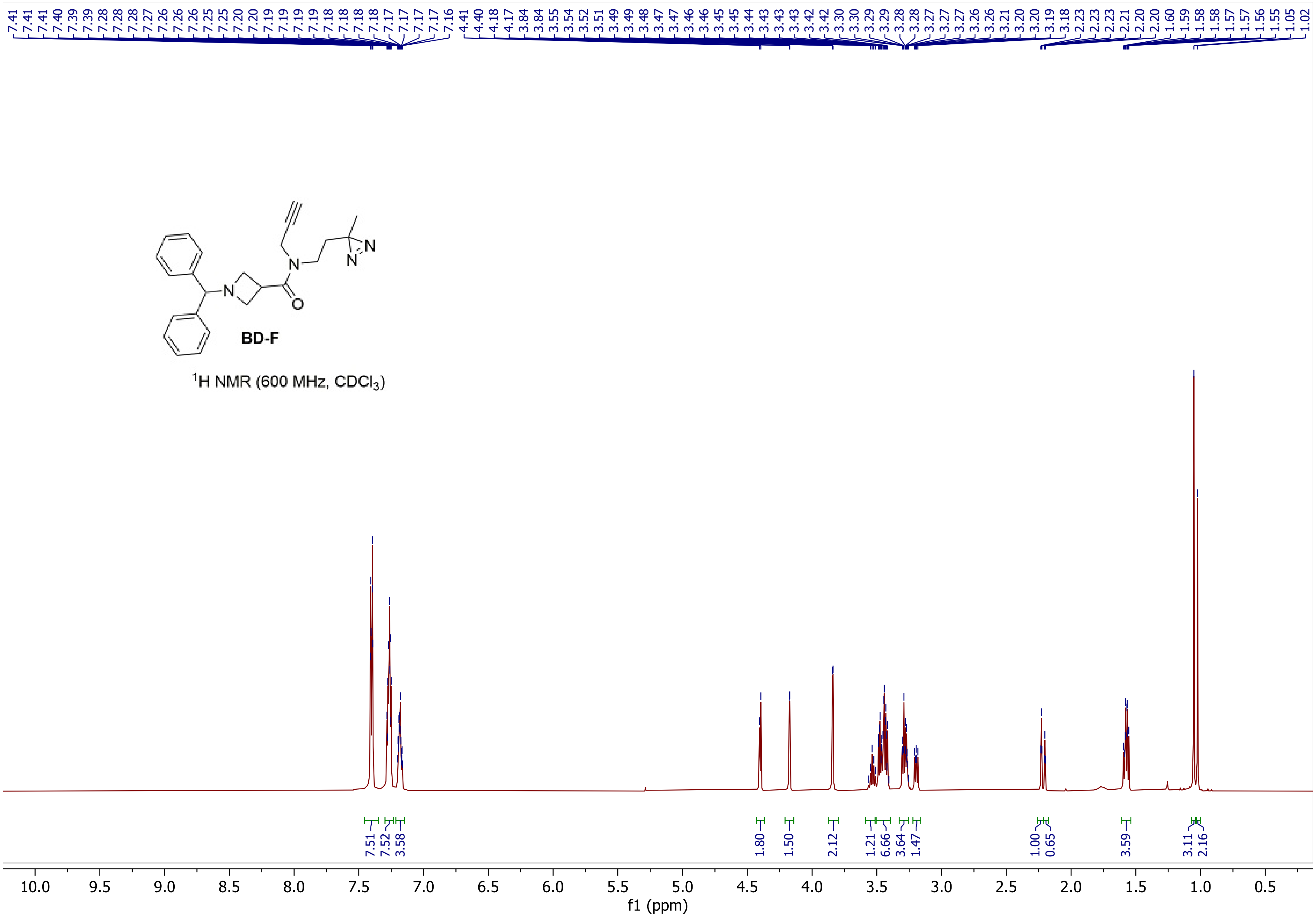
BD-F
1H NMR (600 MHz, CDCl3)
f1 (ppm)

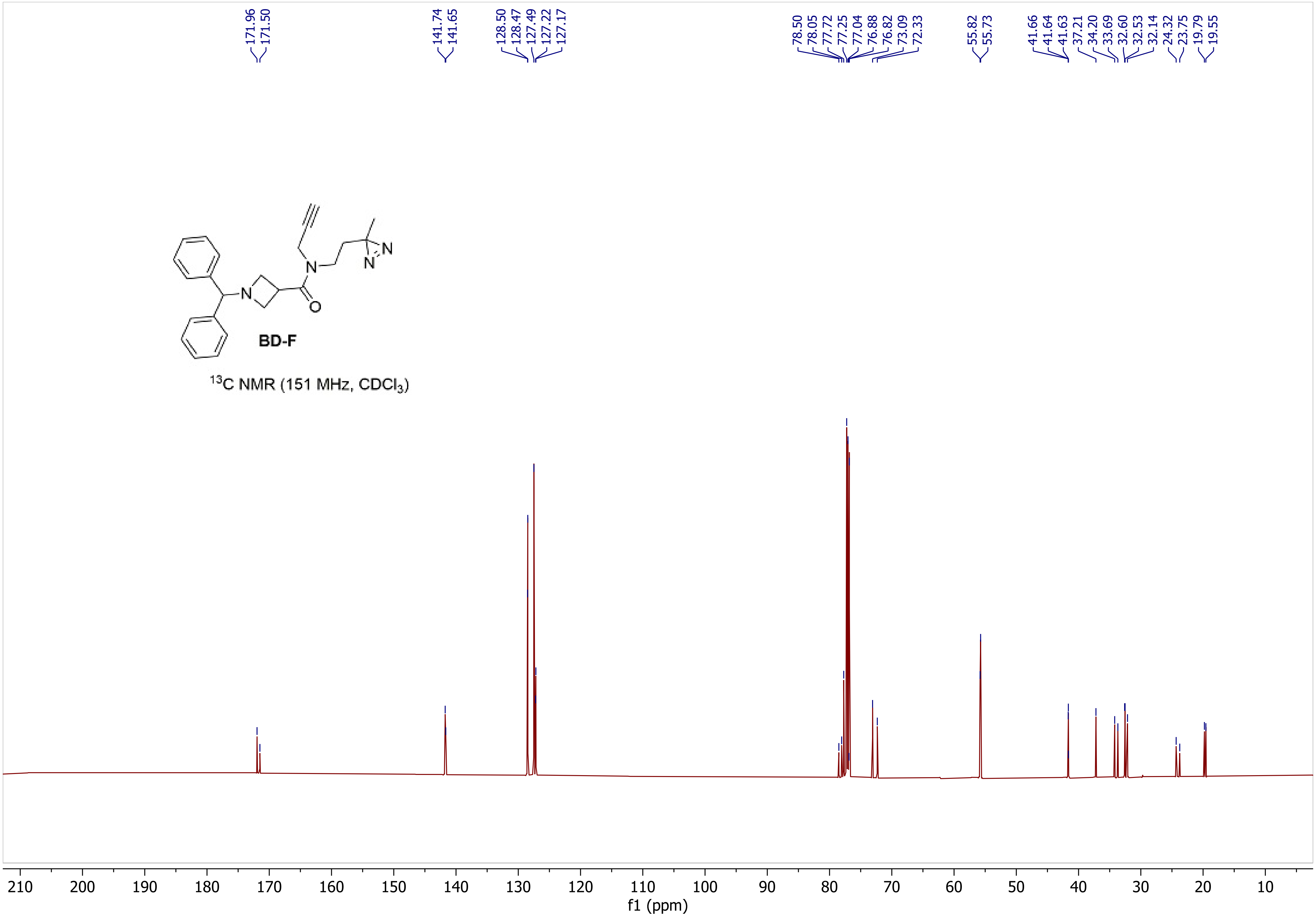
171.96
171.50
141.74
141.65
128.50
128.47
127.49
127.22
127.17
78.50
78.05
77.72
77.25
77.04
76.88
76.82
73.09
72.33
55.82
55.73
41.66
41.64
41.63
37.21
34.20
33.69
32.60
32.53
32.14
24.32
23.75
19.79
19.55
BD-F
^{13}C NMR (151 MHz, $CDCl_3$)
210 200 190 180 170 160 150 140 130 120 110 100 90 80 70 60 50 40 30 20 10
f1 (ppm)

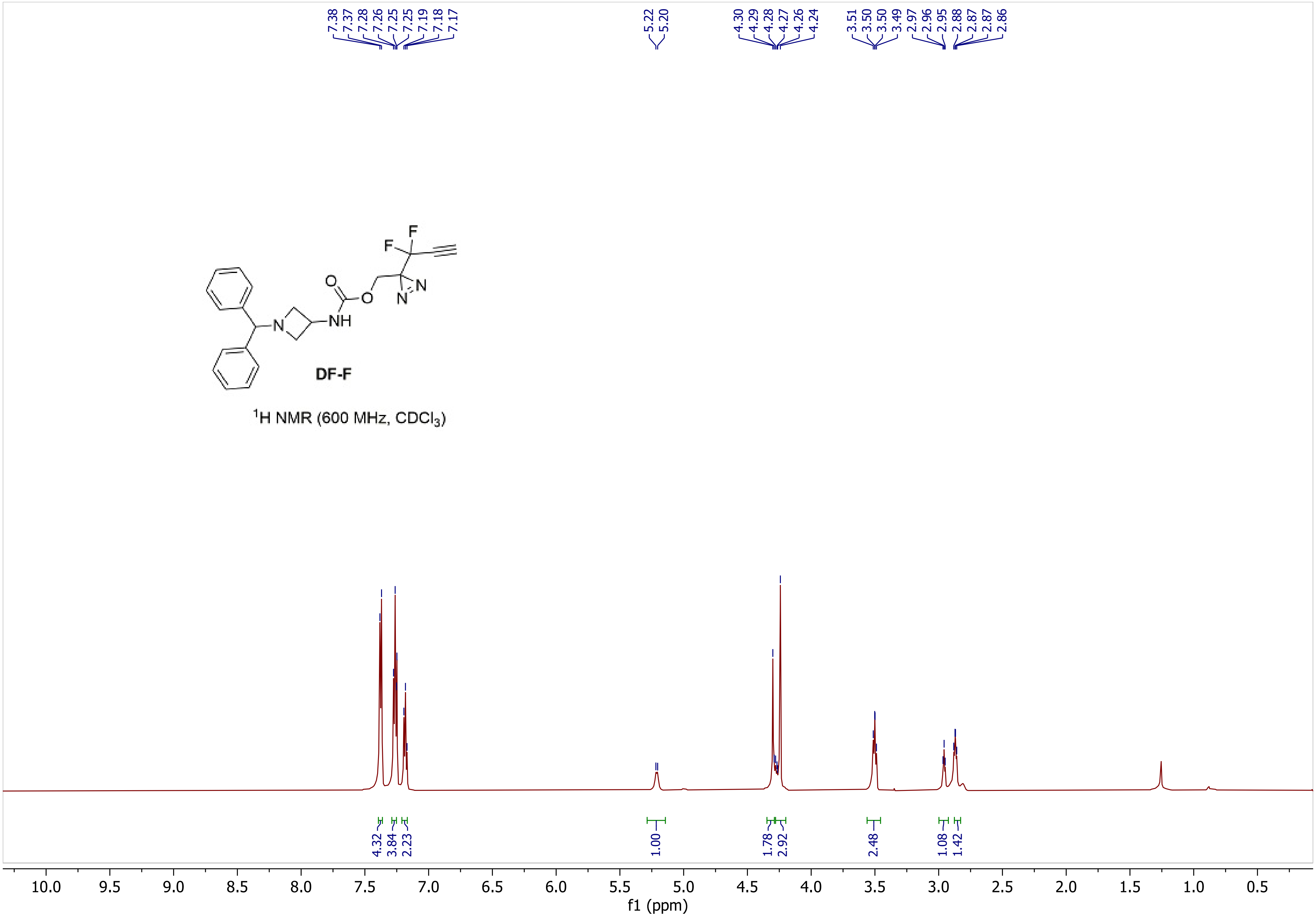
7.38
7.37
7.28
7.26
7.25
7.25
7.19
7.18
7.17
5.22
5.20
4.30
4.29
4.28
4.27
4.26
4.24
3.51
3.50
3.50
3.49
2.97
2.96
2.95
2.88
2.87
2.87
2.86
F
F
O
O
N
N
N
NH
DF-F
1H NMR (600 MHz, CDCl3)
4.32
3.84
2.23
1.00
1.78
2.92
2.48
1.08
1.42
10.0
9.5
9.0
8.5
8.0
7.5
7.0
6.5
6.0
5.5
5.0
4.5
4.0
3.5
3.0
2.5
2.0
1.5
1.0
0.5
f1 (ppm)

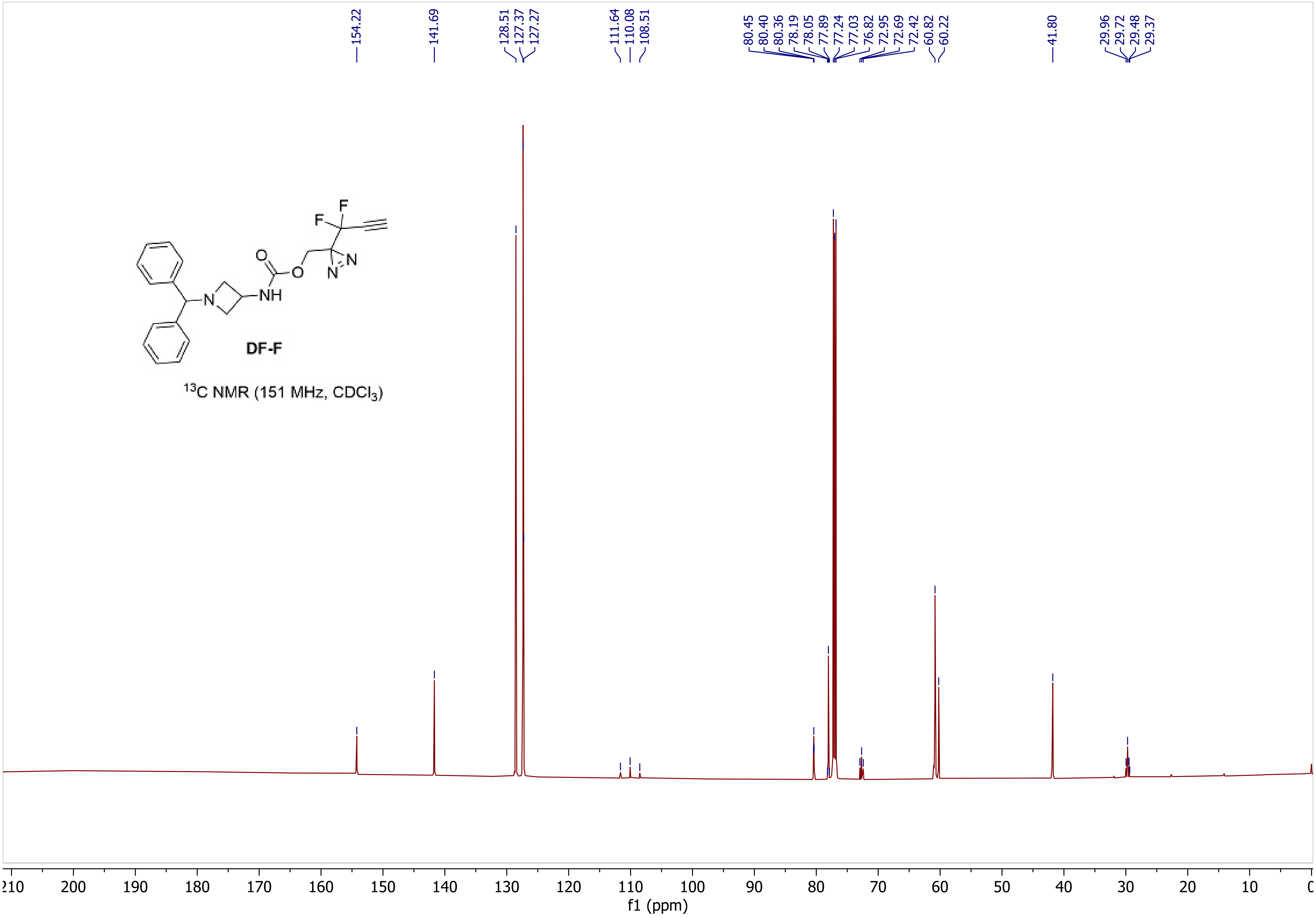
154.22
141.69
128.51
127.37
127.27
111.64
110.08
108.51
80.45
80.40
80.36
78.19
78.05
77.89
77.24
77.03
76.82
72.95
72.69
72.42
60.82
60.22
41.80
29.96
29.72
29.48
29.37
DF-F
^{13}C NMR (151 MHz, $CDCl_3$)
f1 (ppm)

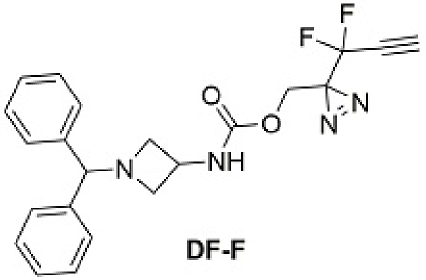

DF-F

^{19}F NMR (376 MHz, $CDCl_3$)

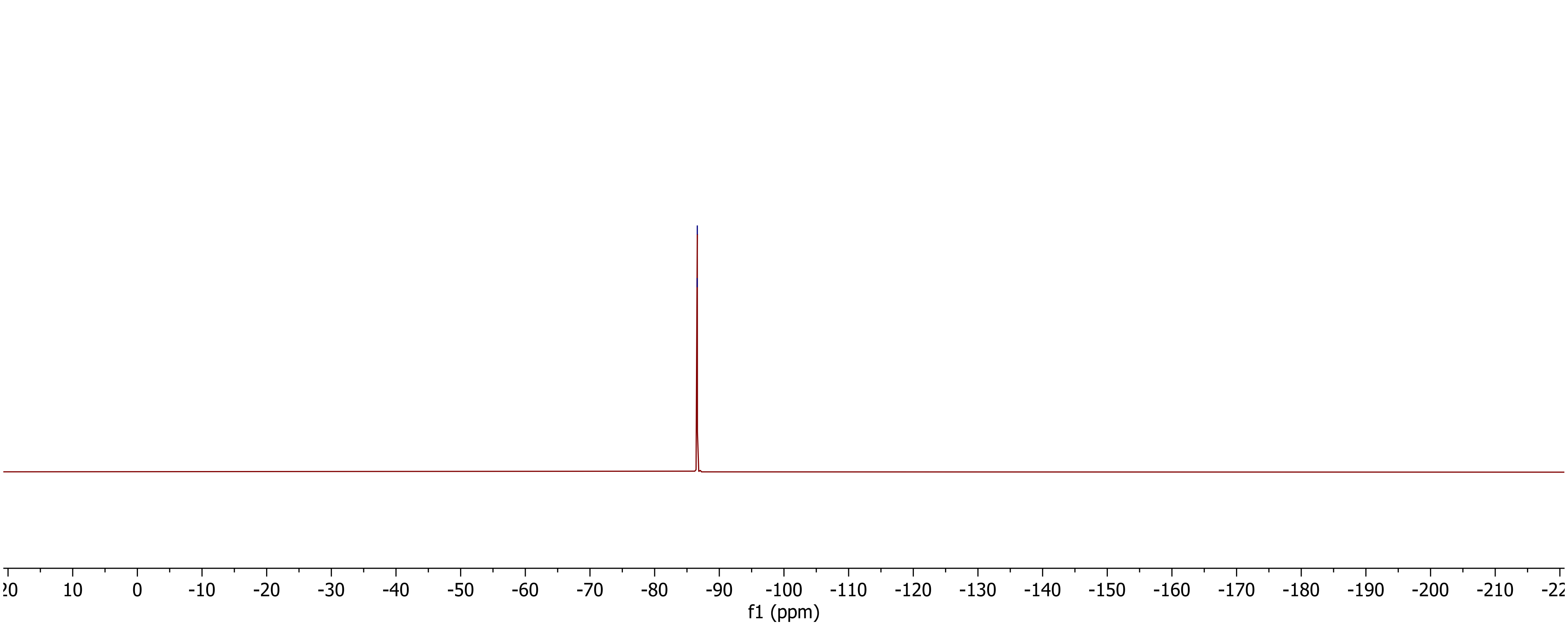

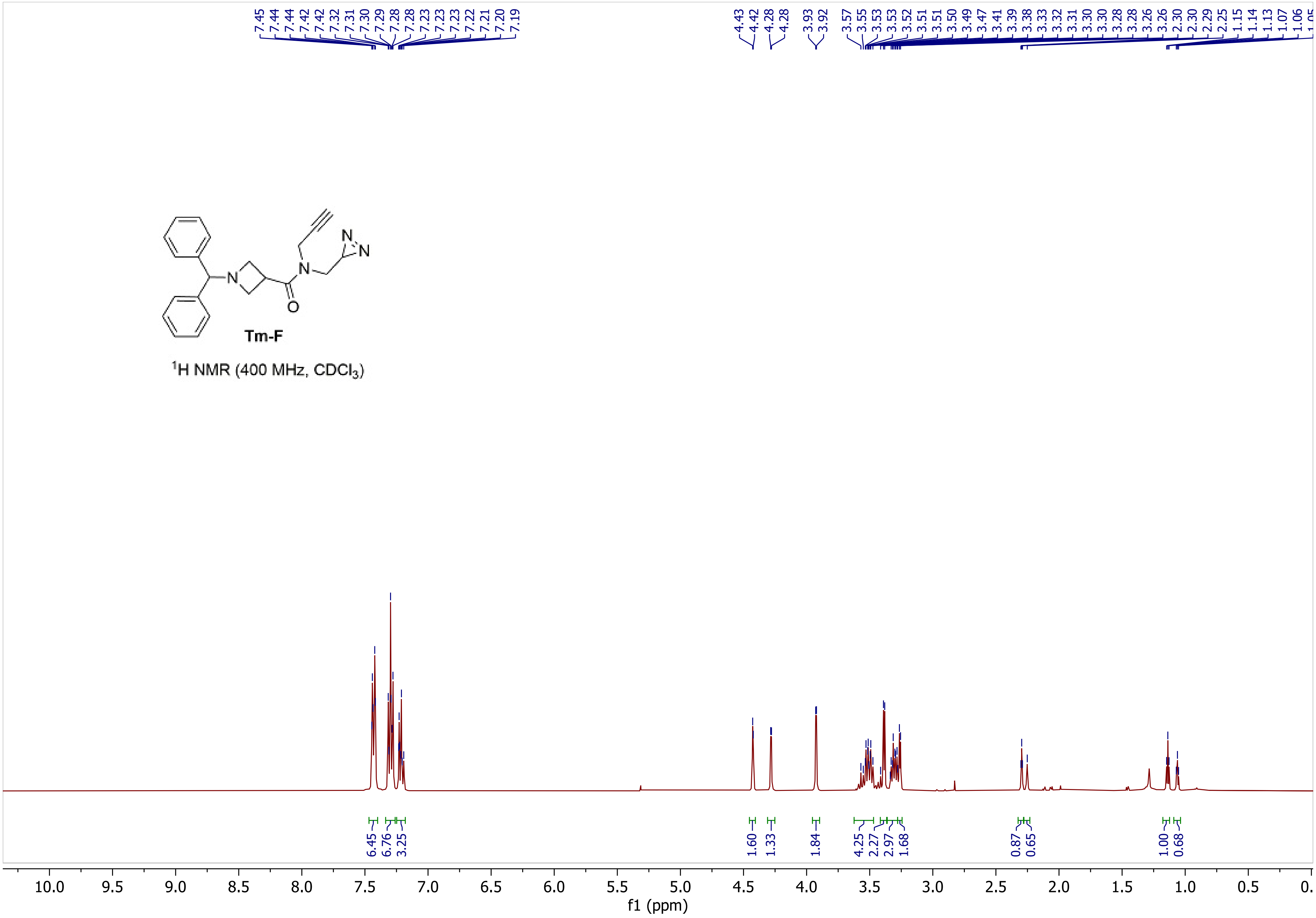

7.45 7.44 7.44 7.42 7.42 7.32 7.31 7.30 7.29 7.28 7.28 7.23 7.23 7.23 7.22 7.21 7.20 7.19
4.43 4.42 4.28 4.28 3.93 3.92 3.57 3.55 3.53 3.53 3.52 3.51 3.51 3.50 3.49 3.47 3.41 3.39 3.38 3.33 3.32 3.31 3.30 3.30 3.28 3.28 3.26 3.26 2.30 2.30 2.29 2.25 1.15 1.14 1.13 1.07 1.06
Tm-F
^{1}H NMR (400 MHz, CDCl$_3$)
6.45 6.76 3.25 1.60 1.33 1.84 4.25 2.27 2.97 1.68 0.87 0.65 1.00 0.68
10.0 9.5 9.0 8.5 8.0 7.5 7.0 6.5 6.0 5.5 5.0 4.5 4.0 3.5 3.0 2.5 2.0 1.5 1.0 0.5
f1 (ppm)

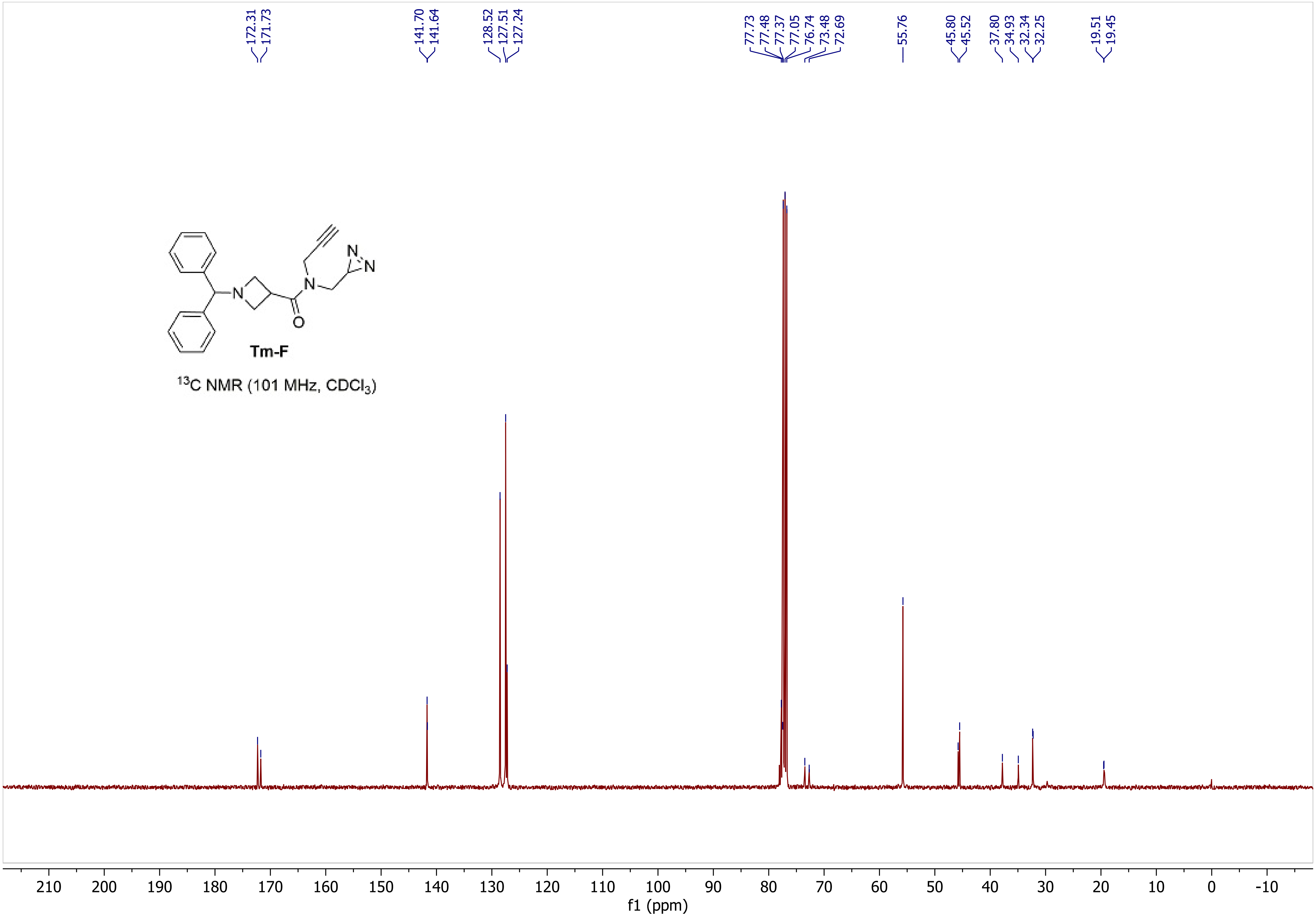
172.31
171.73
141.70
141.64
128.52
127.51
127.24
77.73
77.48
77.37
77.05
76.74
73.48
72.69
55.76
45.80
45.52
37.80
34.93
32.34
32.25
19.51
19.45
N
N
N
N
O
Tm-F
13C NMR (101 MHz, CDCl3)
210
200
190
180
170
160
150
140
130
120
110
100
90
80
70
60
50
40
30
20
10
0
-10
f1 (ppm)

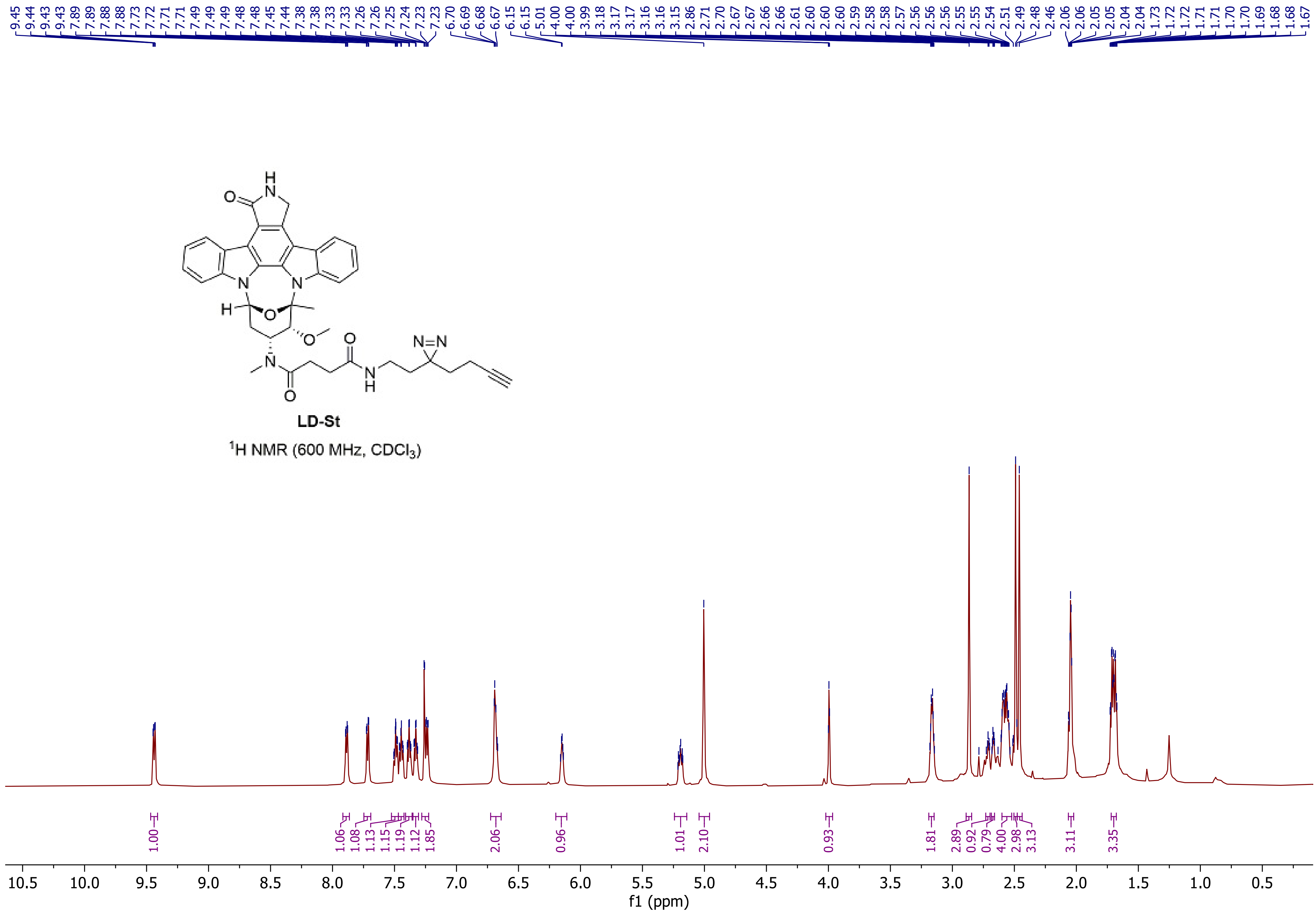

LD-St

^{1}H NMR (600 MHz, $CDCl_3$)

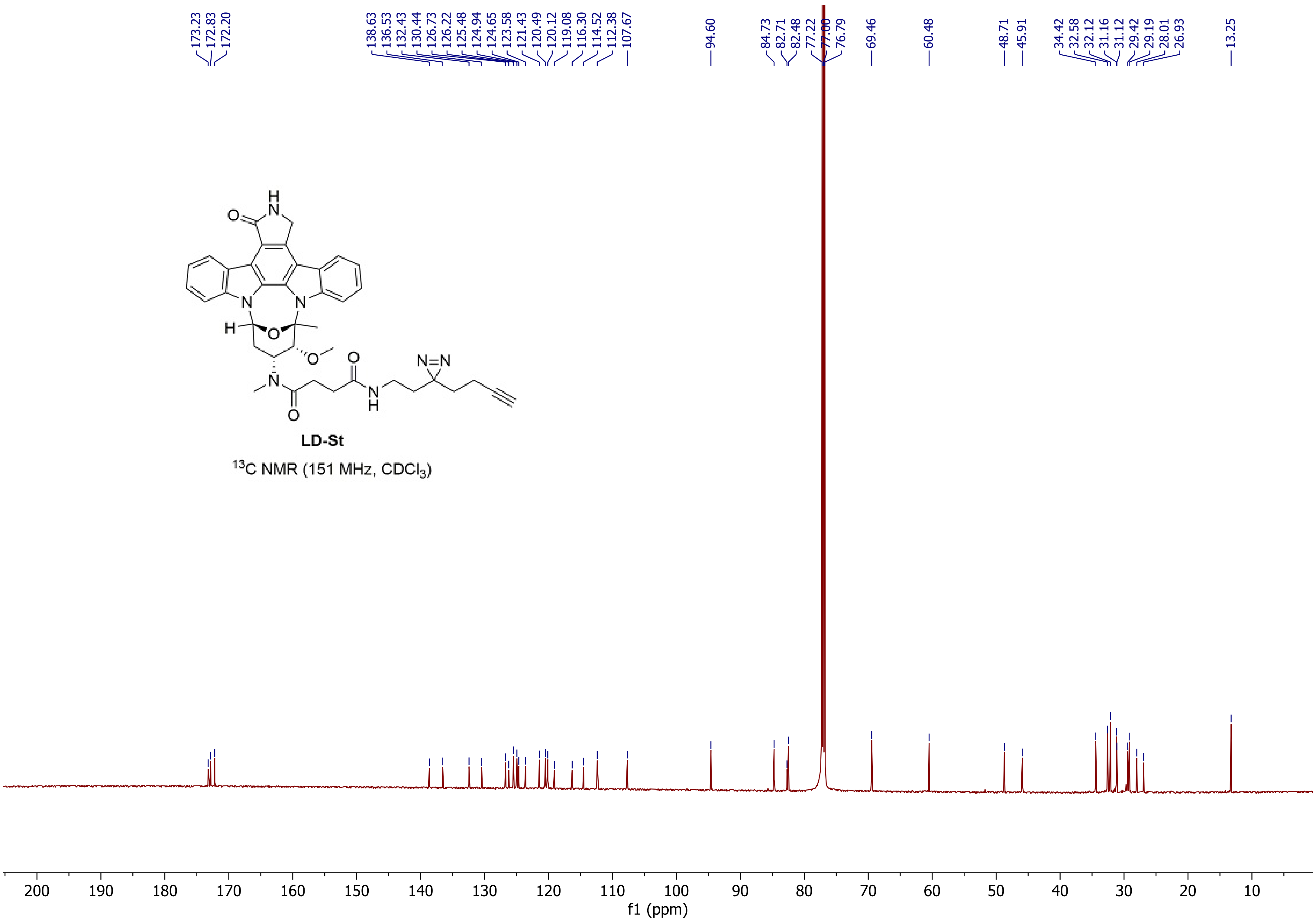
173.23
172.83
172.20
138.63
136.53
132.43
130.44
126.73
126.22
125.48
124.94
124.65
123.58
121.43
120.49
120.12
119.08
116.30
114.52
112.38
107.67
94.60
84.73
82.71
82.48
77.22
77.00
76.79
69.46
60.48
48.71
45.91
34.42
32.58
32.12
31.16
31.12
29.42
29.19
28.01
26.93
13.25
LD-St
^{13}C NMR (151 MHz, $CDCl_3$)
200
190
180
170
160
150
140
130
120
110
100
90
80
70
60
50
40
30
20
10
f1 (ppm)

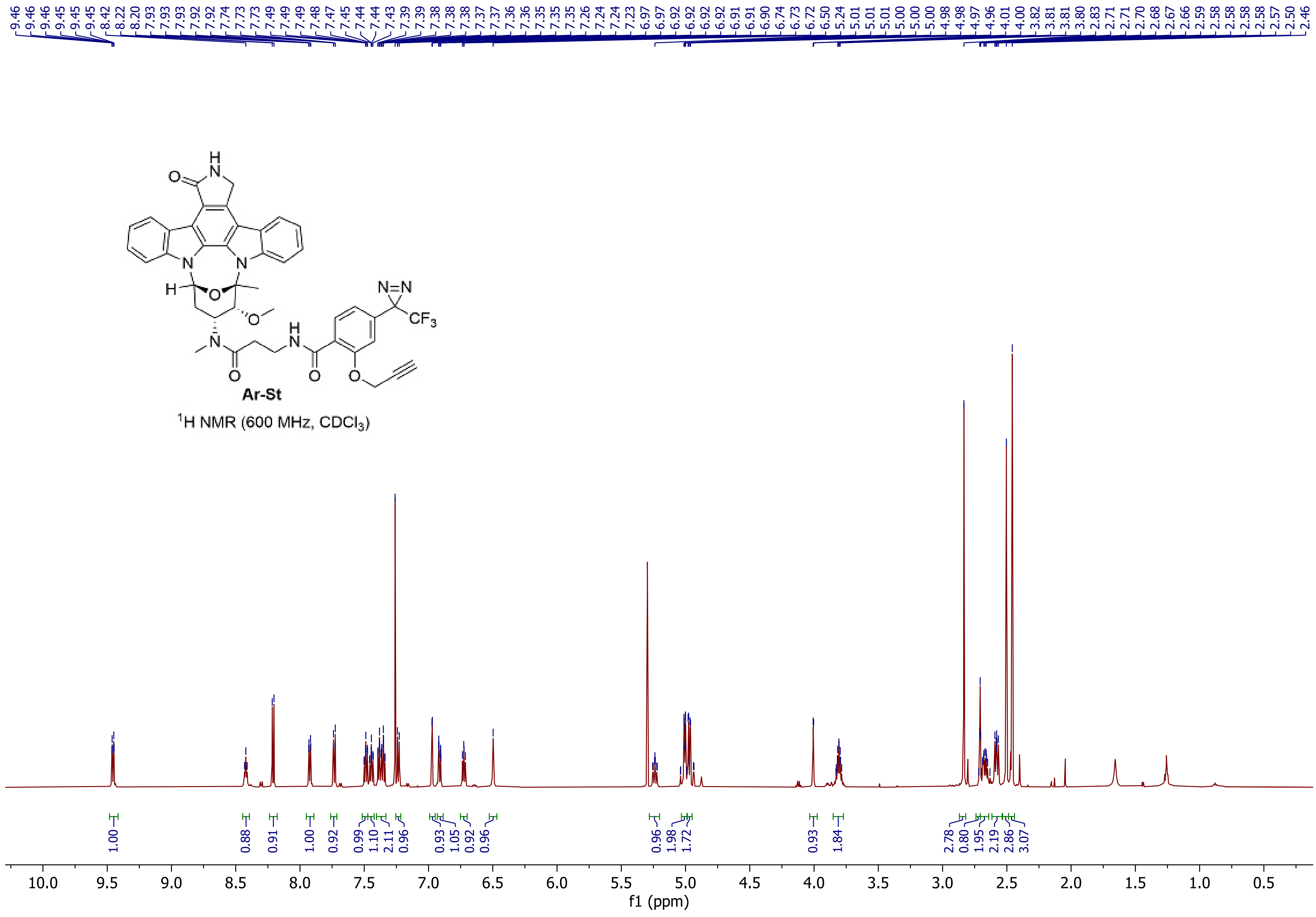
9.46 9.46 9.46 9.45 9.45 9.45 8.42 8.22 8.20 7.93 7.93 7.93 7.92 7.92 7.74 7.73 7.73 7.49 7.49 7.49 7.48 7.47 7.45 7.44 7.44 7.43 7.39 7.39 7.38 7.38 7.38 7.37 7.37 7.36 7.36 7.35 7.35 7.26 7.24 7.24 7.23 6.97 6.97 6.92 6.92 6.92 6.92 6.91 6.91 6.90 6.74 6.73 6.72 6.50 5.24 5.01 5.01 5.00 5.00 5.00 4.98 4.98 4.97 4.96 4.01 4.00 3.82 3.81 3.81 3.80 2.83 2.71 2.71 2.70 2.68 2.67 2.66 2.59 2.58 2.58 2.58 2.58 2.57 2.50 2.46
Ar-St
^{1}H NMR (600 MHz, CDCl$_3$)
1.00 0.88 0.91 1.00 0.92 0.99 1.10 2.11 0.96 0.93 1.05 0.92 0.96 0.96 1.98 1.72 0.93 1.84 2.78 0.80 1.95 2.19 2.86 3.07
10.0 9.5 9.0 8.5 8.0 7.5 7.0 6.5 6.0 5.5 5.0 4.5 4.0 3.5 3.0 2.5 2.0 1.5 1.0 0.5
f1 (ppm)

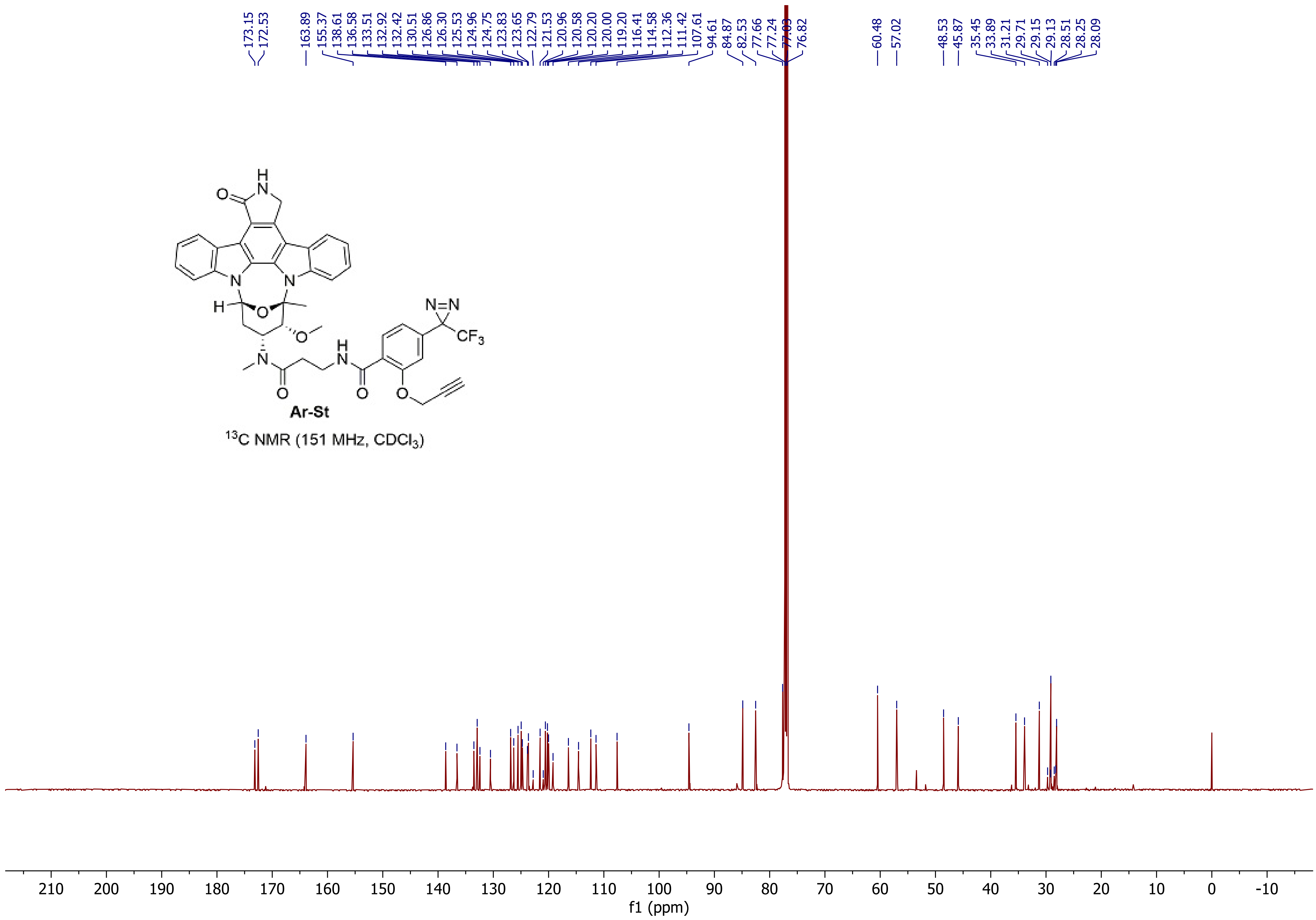
173.15
172.53
163.89
155.37
138.61
136.58
133.51
132.92
132.42
130.51
126.86
126.30
125.53
124.96
124.75
123.83
123.65
122.79
121.53
120.96
120.58
120.20
120.00
119.20
116.41
114.58
112.36
111.42
107.61
94.61
84.87
82.53
77.66
77.24
77.03
76.82
60.48
57.02
48.53
45.87
35.45
33.89
31.21
29.71
29.15
29.13
28.51
28.25
28.09
Ar-St
^{13}C NMR (151 MHz, $CDCl_3$)
210 200 190 180 170 160 150 140 130 120 110 100 90 80 70 60 50 40 30 20 10 0 -10
f1 (ppm)

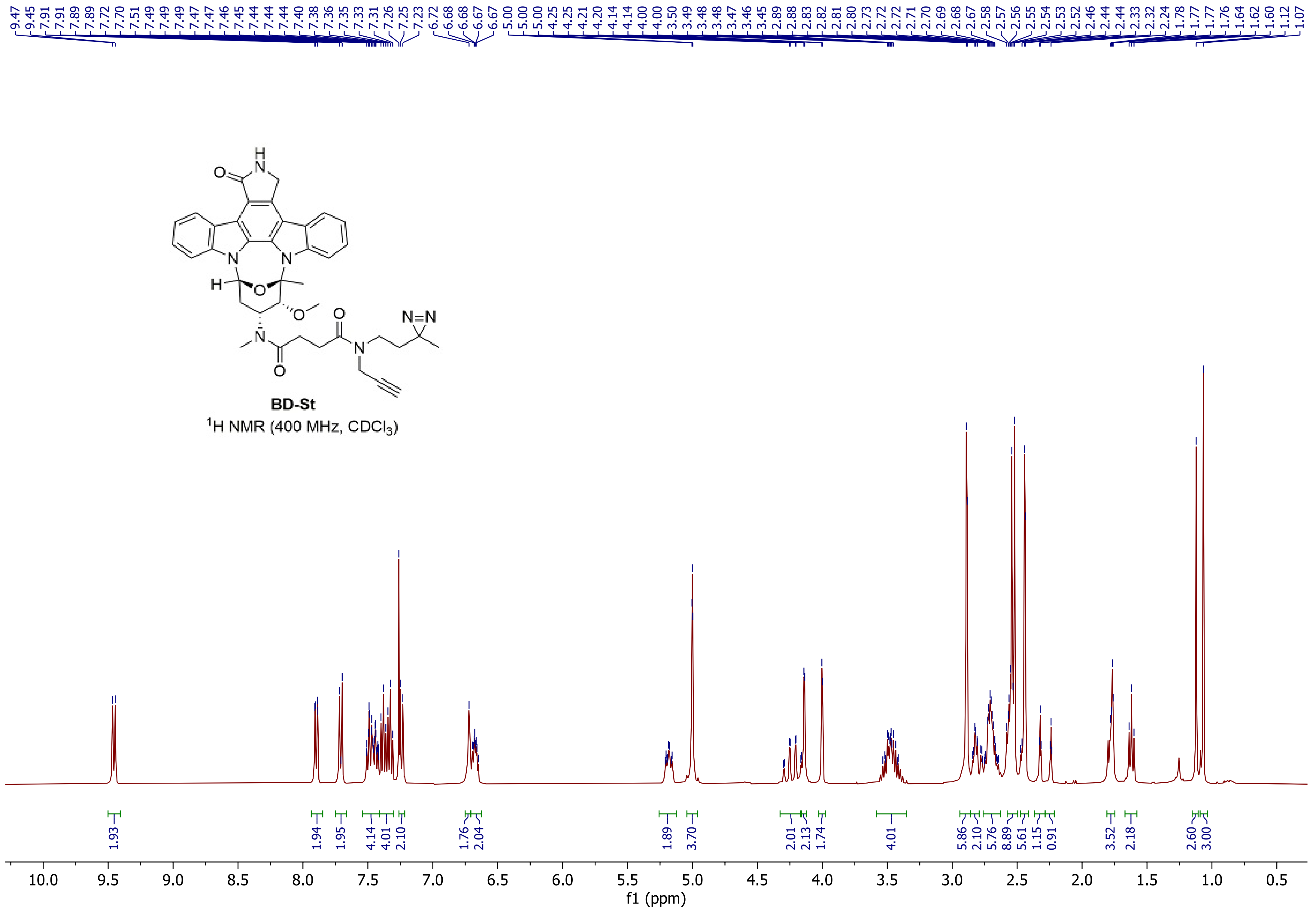
9.47 9.45 7.91 7.91 7.89 7.89 7.72 7.70 7.51 7.49 7.49 7.49 7.47 7.47 7.46 7.45 7.44 7.44 7.44 7.40 7.38 7.36 7.35 7.33 7.31 7.26 7.25 7.23 6.72 6.68 6.68 6.67 6.67 5.00 5.00 4.25 4.25 4.21 4.20 4.14 4.14 4.00 4.00 3.50 3.49 3.48 3.48 3.47 3.46 3.45 2.89 2.88 2.83 2.82 2.81 2.80 2.73 2.72 2.72 2.71 2.70 2.69 2.68 2.67 2.58 2.57 2.56 2.55 2.54 2.53 2.52 2.46 2.44 2.44 2.33 2.32 2.24 1.78 1.77 1.77 1.76 1.64 1.62 1.60 1.12 1.07
BD-St
1H NMR (400 MHz, CDCl3)
1.93 1.94 1.95 4.14 4.01 2.10 1.76 2.04 1.89 3.70 2.01 2.13 1.74 4.01 5.86 2.10 5.76 8.89 5.61 1.15 0.91 3.52 2.18 2.60 3.00
10.0 9.5 9.0 8.5 8.0 7.5 7.0 6.5 6.0 5.5 5.0 4.5 4.0 3.5 3.0 2.5 2.0 1.5 1.0 0.5
f1 (ppm)

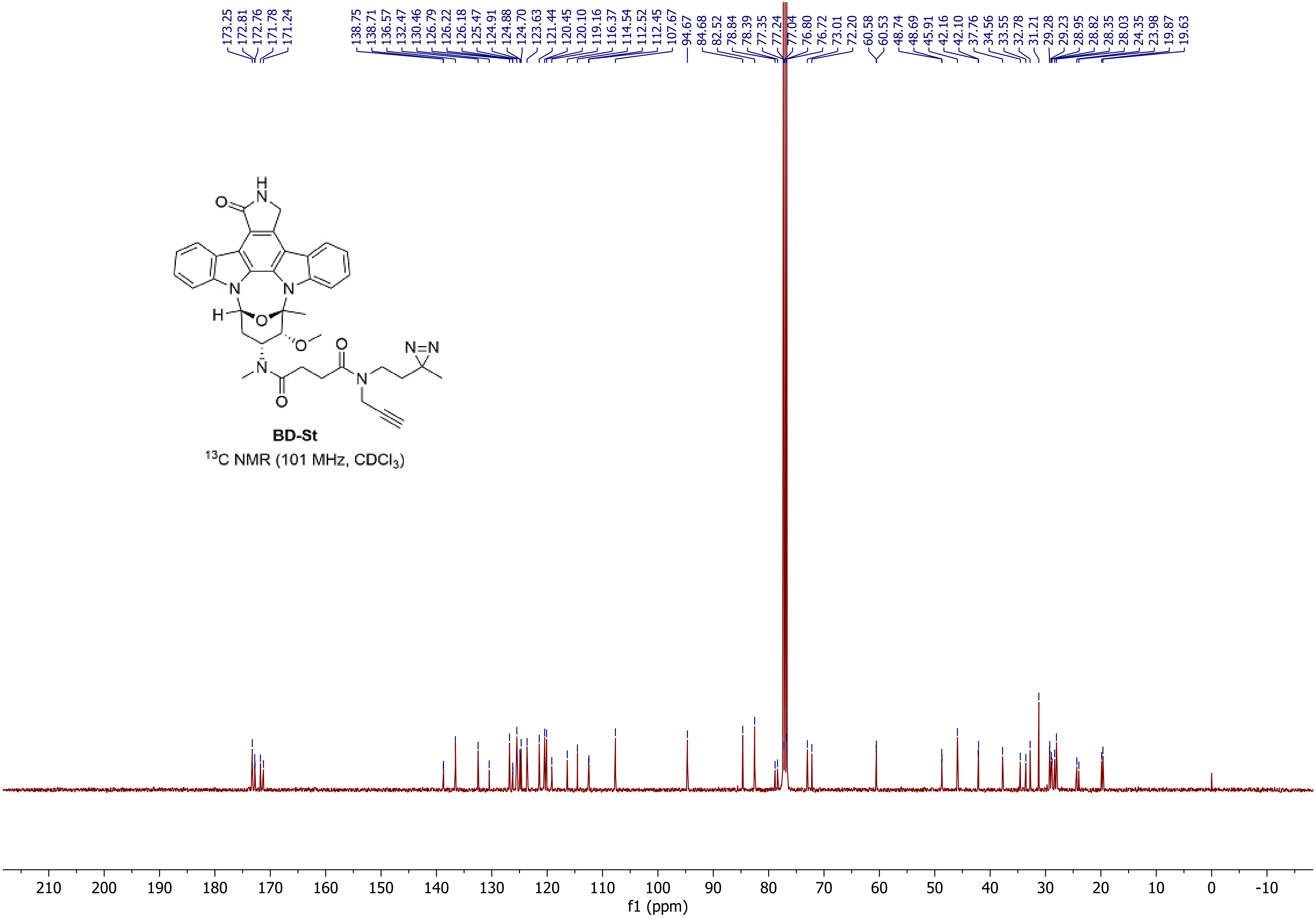
173.25
172.81
172.76
171.78
171.24
138.75
138.71
136.57
132.47
130.46
126.79
126.22
126.18
125.47
124.91
124.88
124.70
123.63
121.44
120.45
120.10
119.16
116.37
114.54
112.52
112.45
107.67
94.67
84.68
82.52
78.84
78.39
77.35
77.24
77.04
76.80
76.72
73.01
72.20
60.58
60.53
48.74
48.69
45.91
42.16
42.10
37.76
34.56
33.55
32.78
31.21
29.28
29.23
28.95
28.82
28.35
28.03
24.35
23.98
19.87
19.63
BD-St
^{13}C NMR (101 MHz, CDCl$_3$)
210 200 190 180 170 160 150 140 130 120 110 100 90 80 70 60 50 40 30 20 10 0 -10
f1 (ppm)

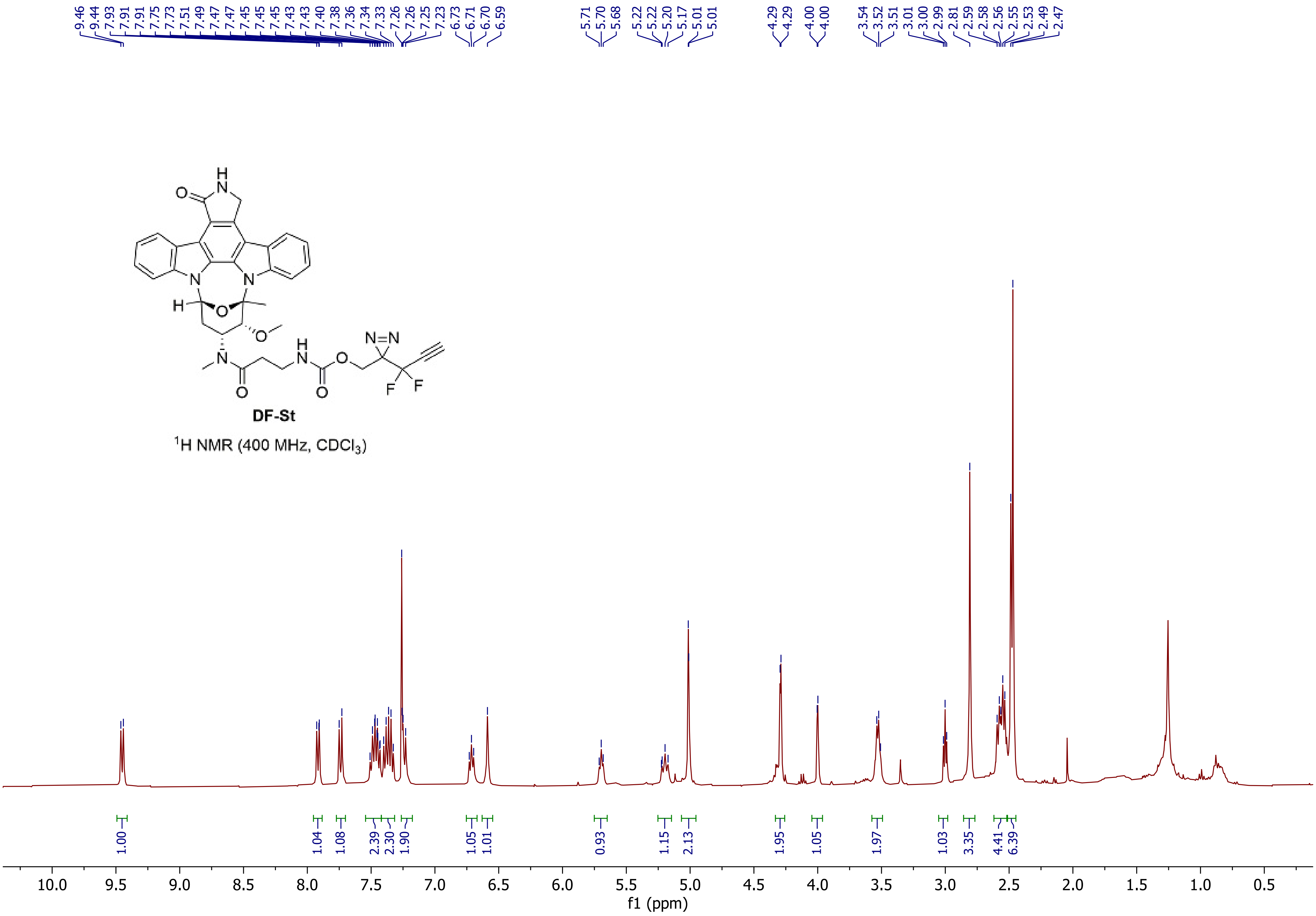

DF-St

^{1}H NMR (400 MHz, $CDCl_3$)

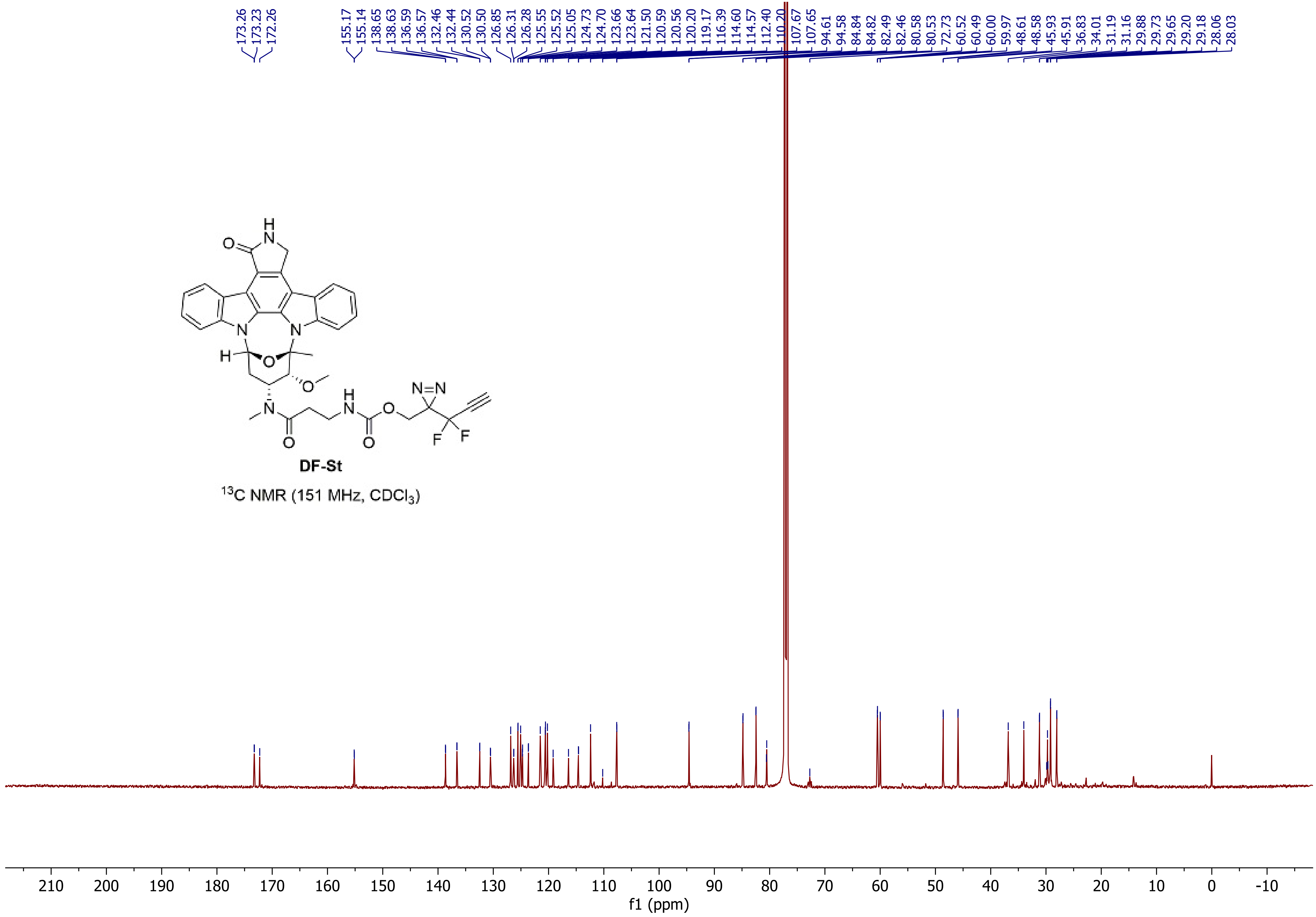
173.26
173.23
172.26
155.17
155.14
138.65
138.63
136.59
136.57
132.46
132.44
130.52
130.50
126.85
126.31
126.28
125.55
125.52
125.05
124.73
124.70
123.66
123.64
121.50
120.59
120.56
120.20
119.17
116.39
114.60
114.57
112.40
110.20
107.67
107.65
94.61
94.58
84.84
84.82
82.49
82.46
80.58
80.53
72.73
60.52
60.49
60.00
59.97
48.61
48.58
45.93
45.91
36.83
34.01
31.19
31.16
29.88
29.73
29.65
29.20
29.18
28.06
28.03
DF-St
13C NMR (151 MHz, CDCl3)
210 200 190 180 170 160 150 140 130 120 110 100 90 80 70 60 50 40 30 20 10 0 -10
f1 (ppm)

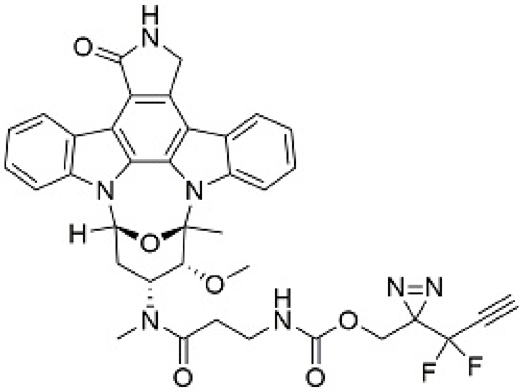

DF-St

^{19}F NMR (376 MHz, $CDCl_3$)

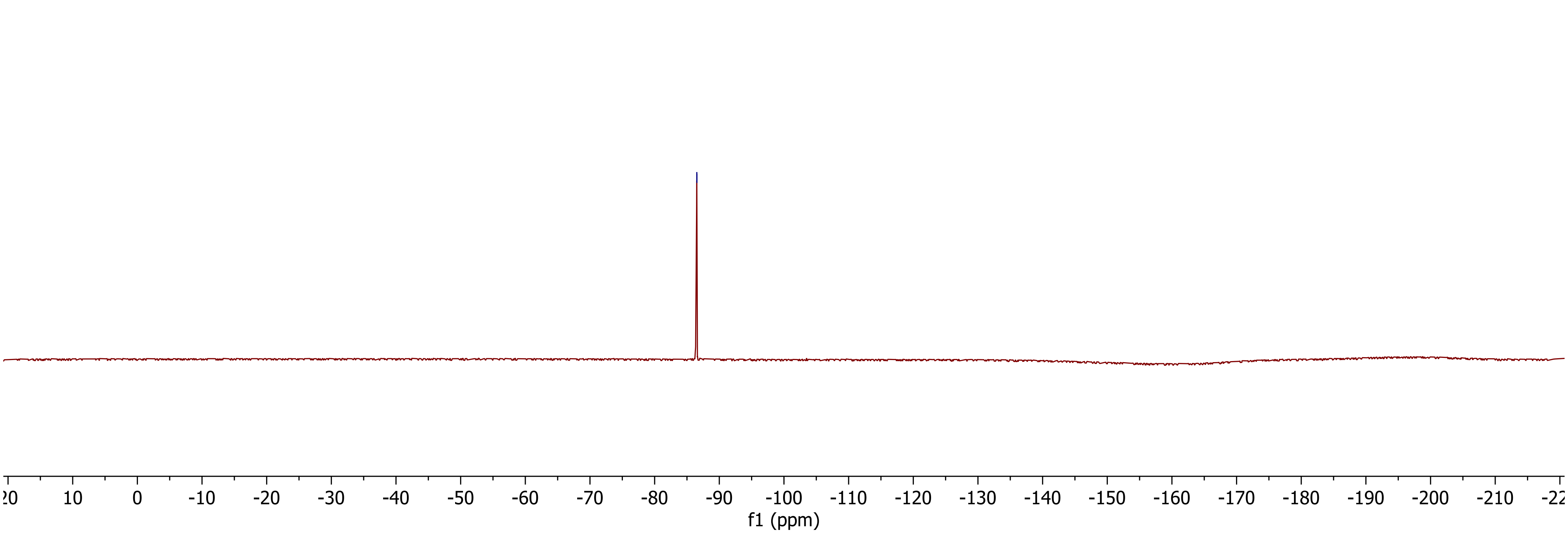

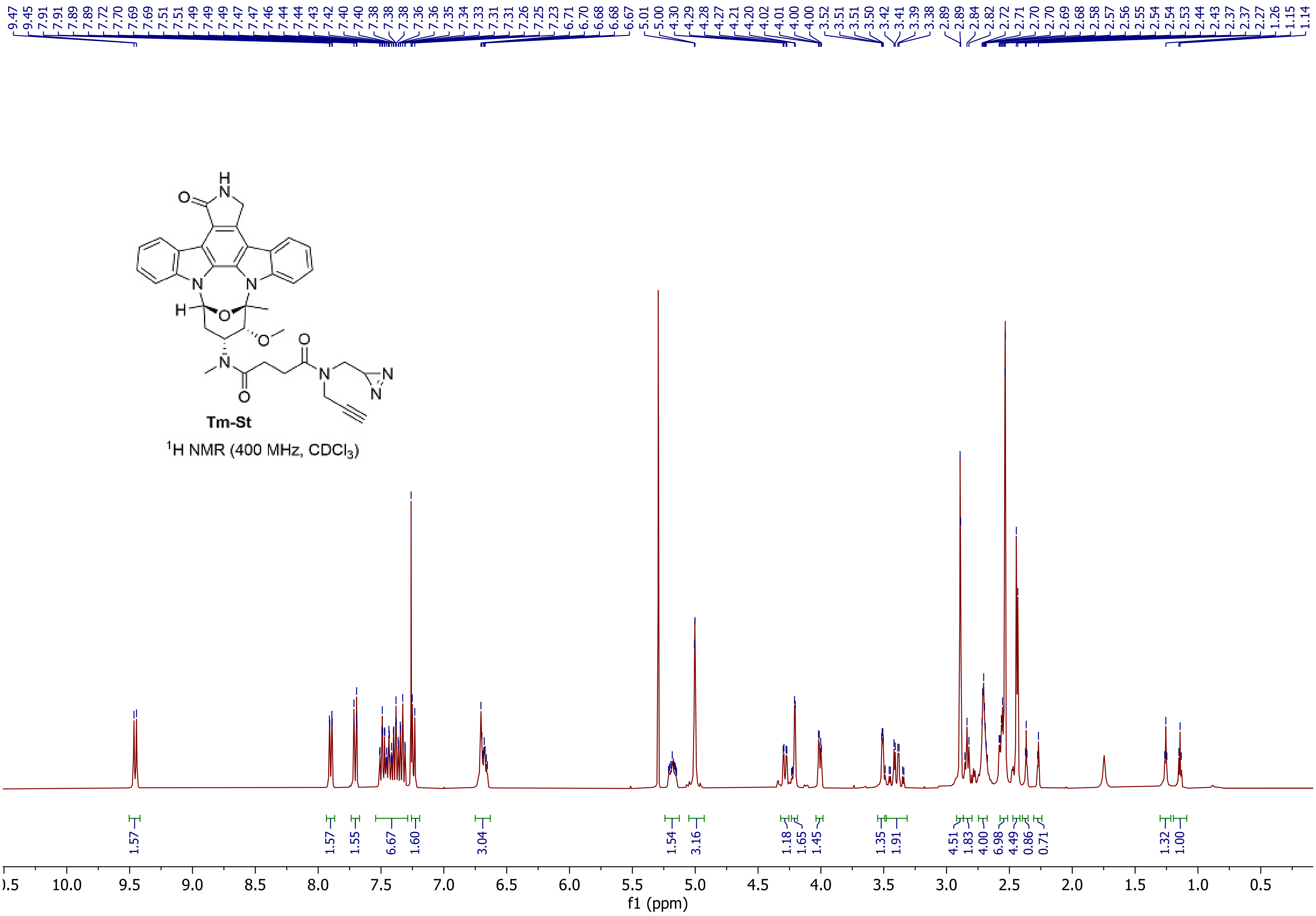
9.47 9.45 7.91 7.89 7.89 7.72 7.70 7.69 7.69 7.51 7.51 7.49 7.49 7.47 7.46 7.44 7.44 7.43 7.42 7.40 7.40 7.38 7.38 7.38 7.36 7.36 7.35 7.34 7.33 7.31 7.31 7.26 7.25 7.23 6.71 6.70 6.68 6.68 6.67 5.01 5.00 4.30 4.29 4.28 4.27 4.21 4.20 4.02 4.01 4.00 4.00 3.52 3.51 3.51 3.50 3.42 3.41 3.39 3.38 2.89 2.89 2.84 2.82 2.72 2.71 2.70 2.70 2.69 2.68 2.58 2.57 2.56 2.55 2.54 2.54 2.53 2.44 2.43 2.37 2.37 2.27 1.26 1.15 1.14
Tm-St
^{1}H NMR (400 MHz, $CDCl_3$)
1.57 1.57 1.55 6.67 1.60 3.04 1.54 3.16 1.18 1.65 1.45 1.35 1.91 4.51 1.83 4.00 6.98 4.49 0.86 0.71 1.32 1.00
.5 10.0 9.5 9.0 8.5 8.0 7.5 7.0 6.5 6.0 5.5 5.0 4.5 4.0 3.5 3.0 2.5 2.0 1.5 1.0 0.5
f1 (ppm)

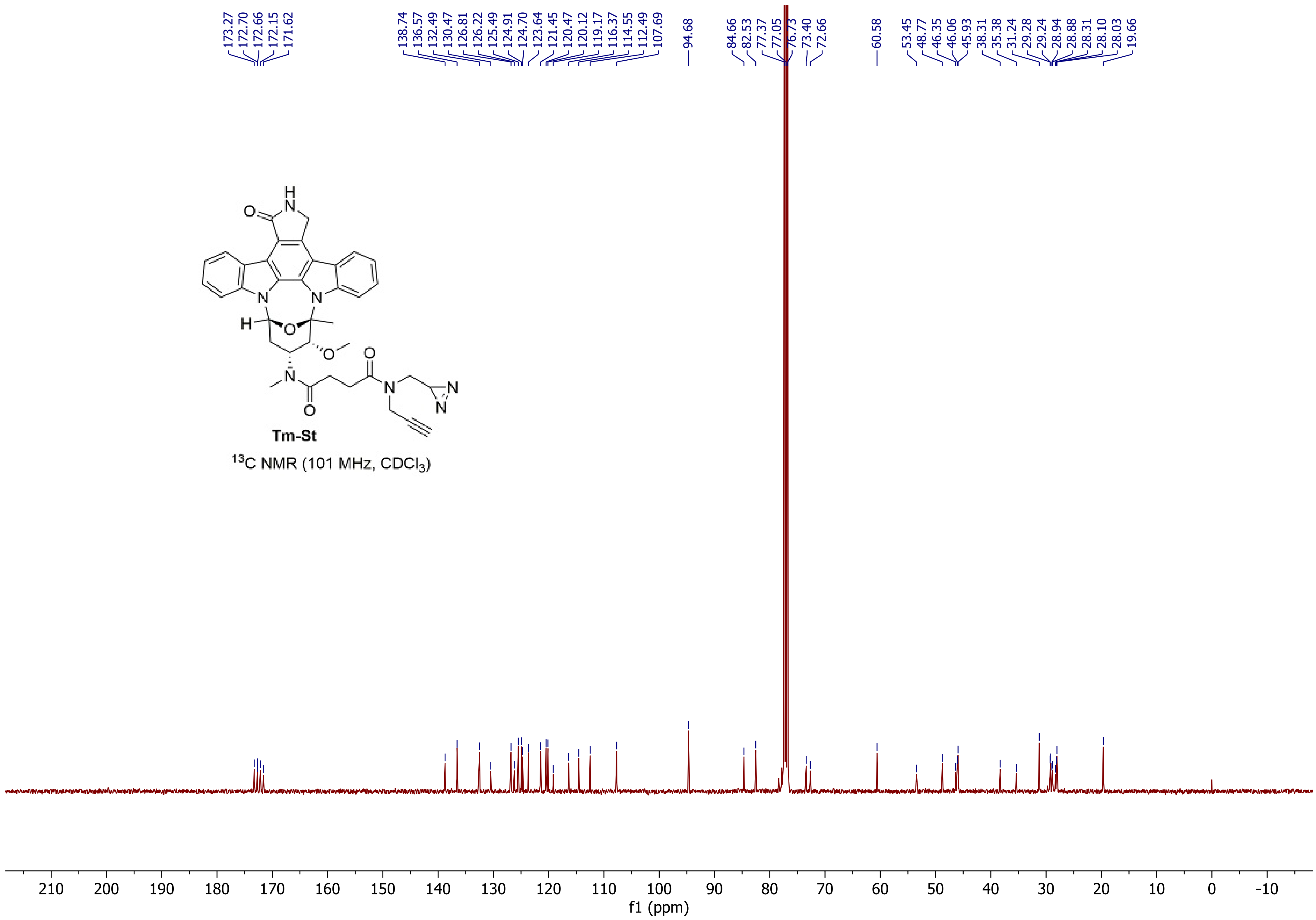
173.27
172.70
172.66
172.15
171.62
138.74
136.57
132.49
130.47
126.81
126.22
125.49
124.91
124.70
123.64
121.45
120.47
120.12
119.17
116.37
114.55
112.49
107.69
94.68
84.66
82.53
77.37
77.05
76.73
73.40
72.66
60.58
53.45
48.77
46.35
46.06
45.93
38.31
35.38
31.24
29.28
29.24
28.94
28.88
28.31
28.10
28.03
19.66
Tm-St
^{13}C NMR (101 MHz, $CDCl_3$)
210 200 190 180 170 160 150 140 130 120 110 100 90 80 70 60 50 40 30 20 10 0 -10
f1 (ppm)

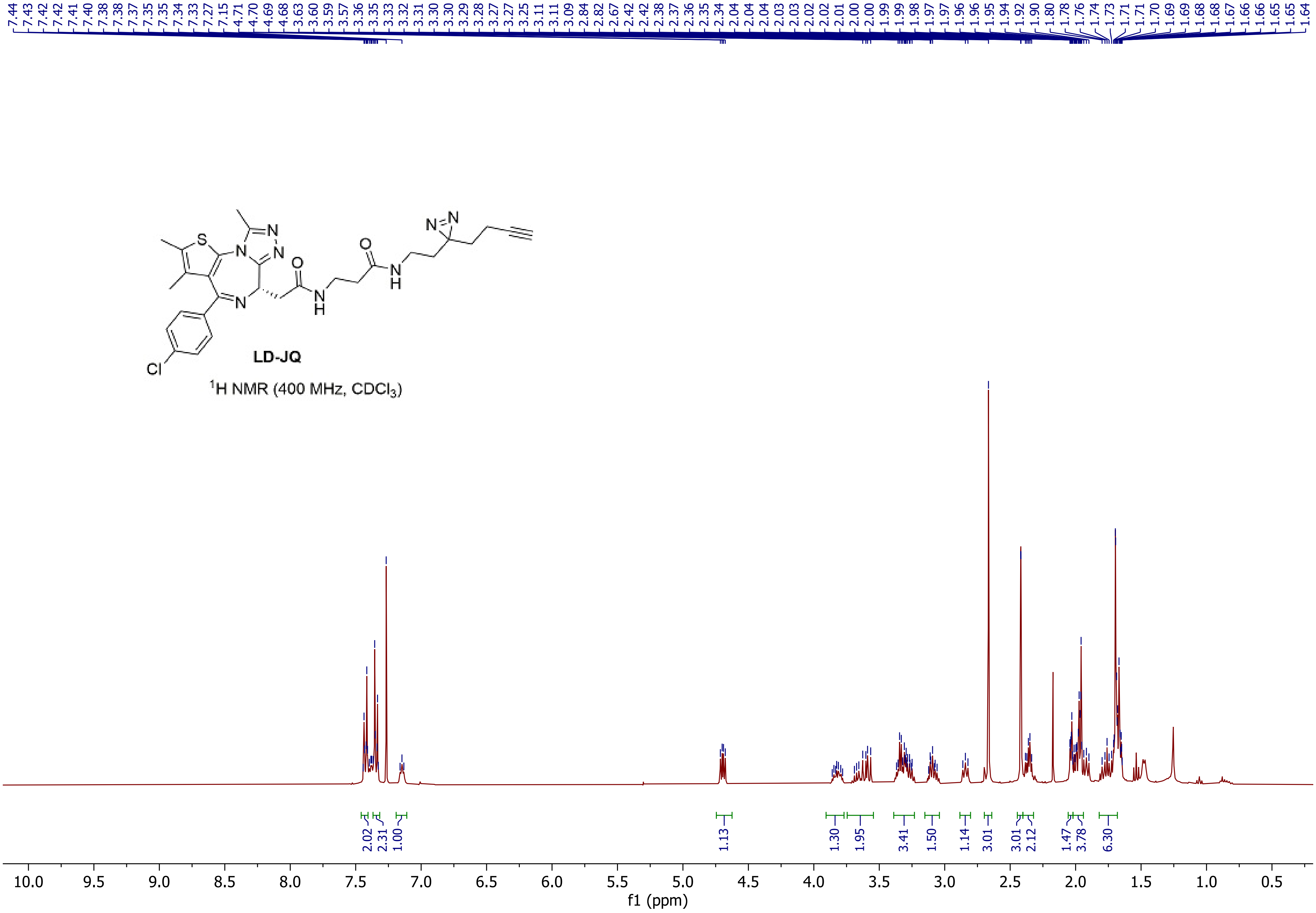
7.44 7.43 7.42 7.42 7.41 7.40 7.38 7.38 7.37 7.35 7.35 7.34 7.33 7.27 7.15 4.71 4.70 4.69 4.68 3.63 3.60 3.59 3.57 3.36 3.35 3.33 3.32 3.31 3.30 3.30 3.29 3.28 3.27 3.27 3.25 3.11 3.11 3.09 2.84 2.82 2.67 2.42 2.42 2.38 2.37 2.36 2.35 2.34 2.04 2.04 2.04 2.03 2.03 2.02 2.02 2.01 2.00 2.00 1.99 1.99 1.98 1.97 1.97 1.96 1.96 1.95 1.94 1.92 1.90 1.80 1.78 1.76 1.74 1.73 1.71 1.71 1.70 1.69 1.69 1.68 1.68 1.67 1.66 1.66 1.65 1.65 1.64
LD-JQ
^{1}H NMR (400 MHz, $CDCl_3$)
2.02 2.31 1.00 1.13 1.30 1.95 3.41 1.50 1.14 3.01 3.01 2.12 1.47 3.78 6.30
10.0 9.5 9.0 8.5 8.0 7.5 7.0 6.5 6.0 5.5 5.0 4.5 4.0 3.5 3.0 2.5 2.0 1.5 1.0 0.5
f1 (ppm)

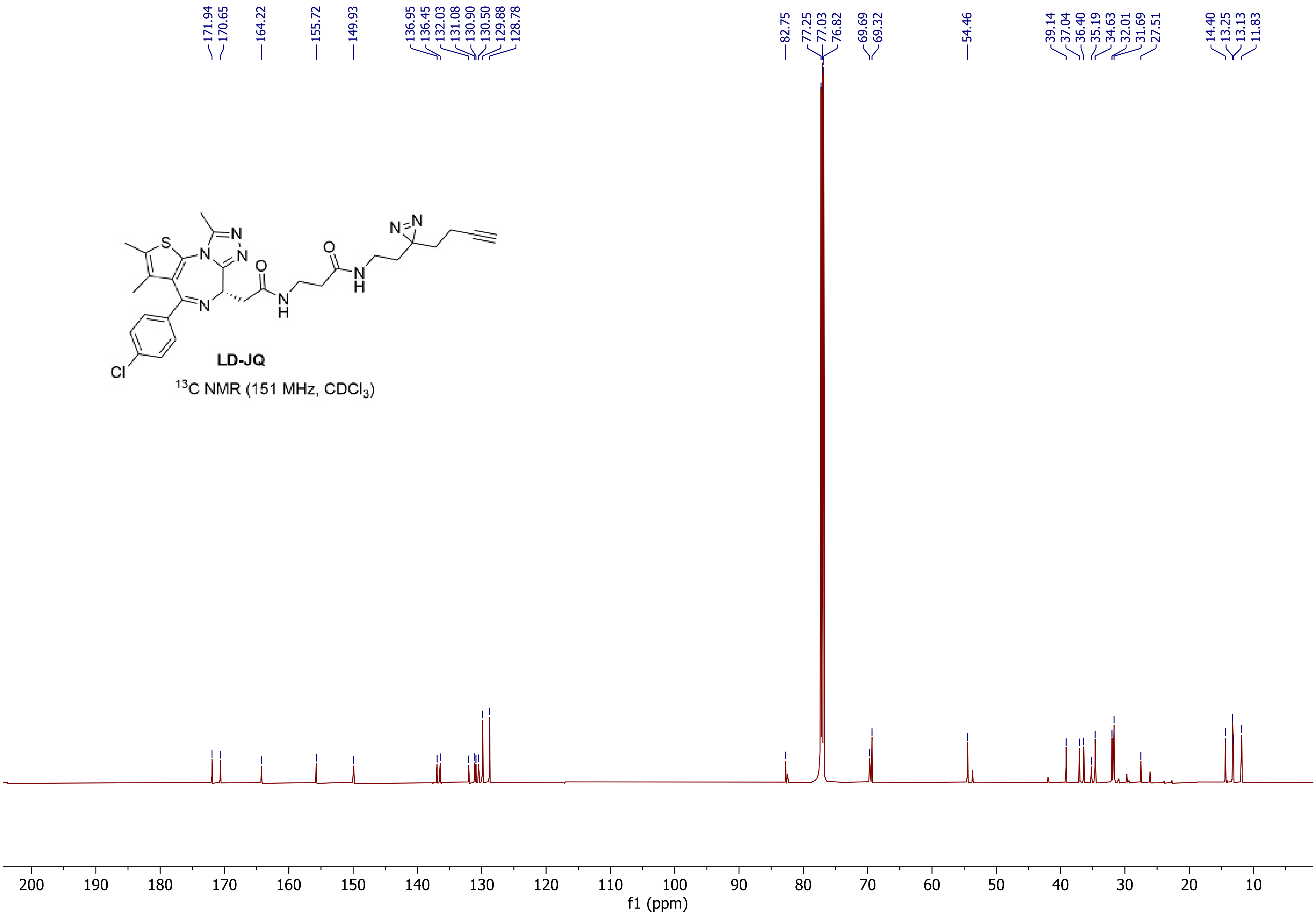

LD-JQ

^{13}C NMR (151 MHz, $CDCl_3$)

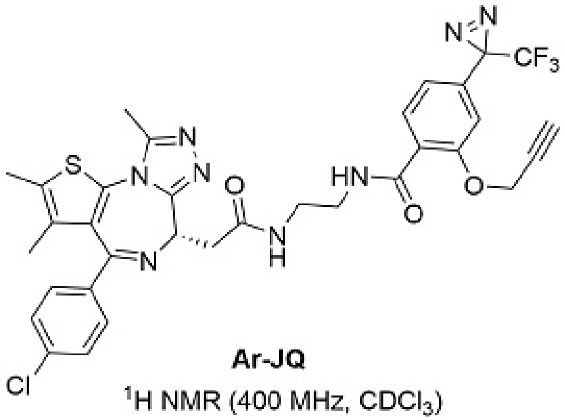

Ar-JQ

^{1}H NMR (400 MHz, $CDCl_3$)

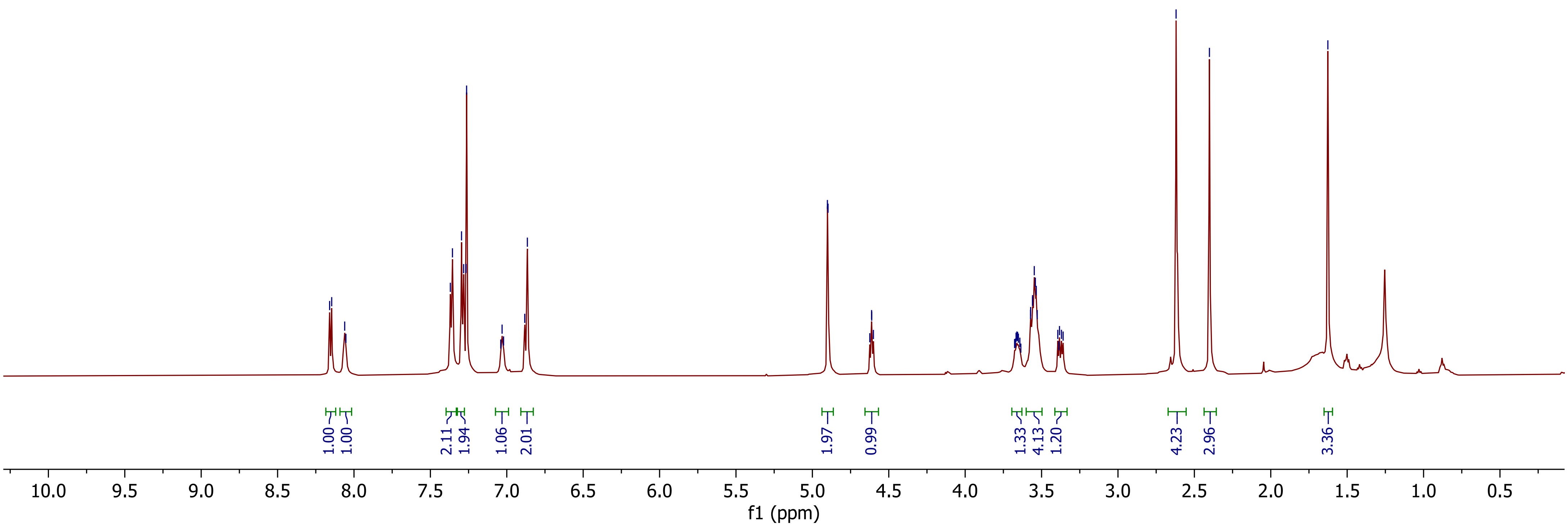

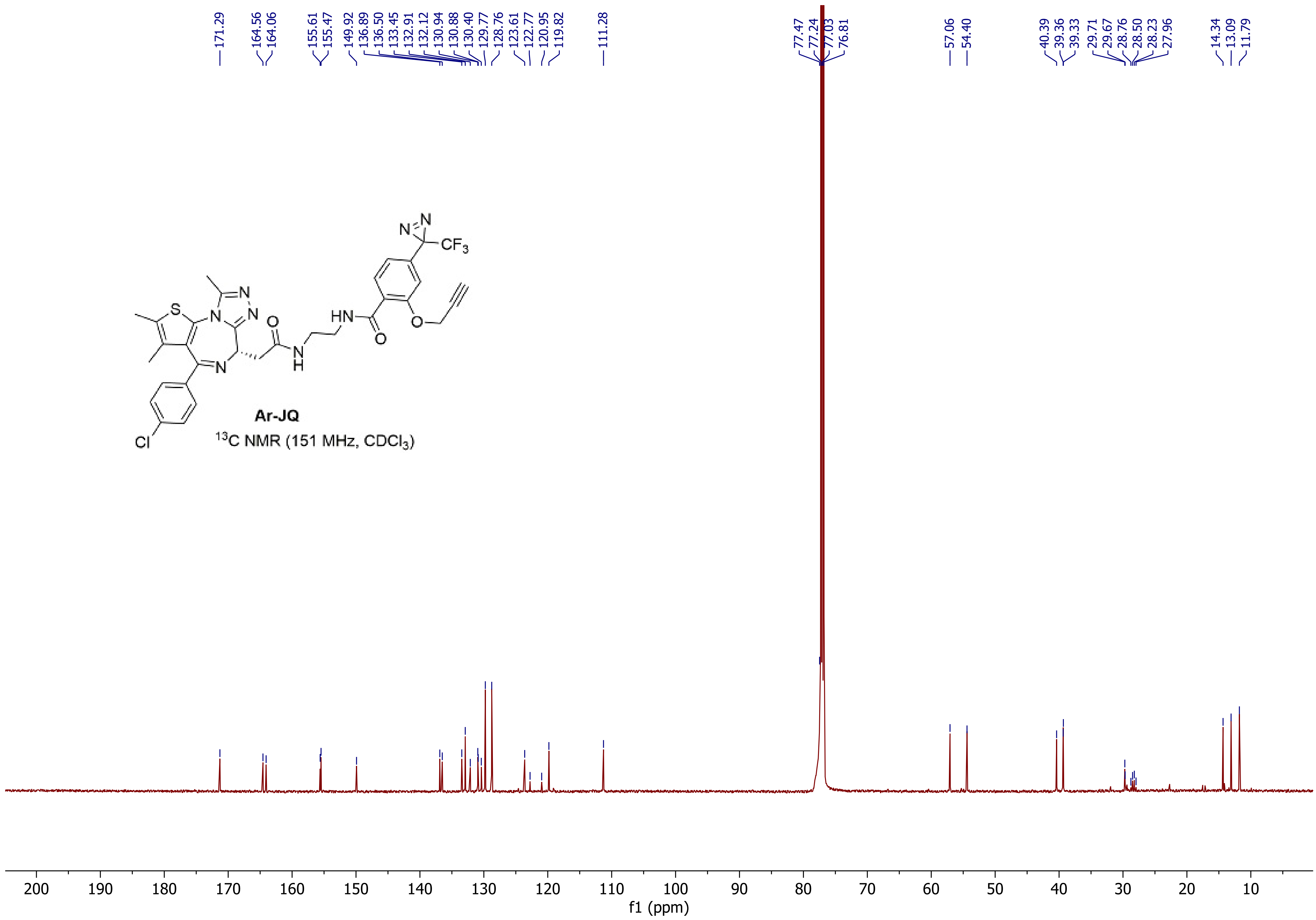
171.29
164.56
164.06
155.61
155.47
149.92
136.89
136.50
133.45
132.91
132.12
130.94
130.88
130.40
129.77
128.76
123.61
122.77
120.95
119.82
111.28
77.47
77.24
77.03
76.81
57.06
54.40
40.39
39.36
39.33
29.71
29.67
28.76
28.50
28.23
27.96
14.34
13.09
11.79
Ar-JQ
^{13}C NMR (151 MHz, $CDCl_3$)
200 190 180 170 160 150 140 130 120 110 100 90 80 70 60 50 40 30 20 10
f1 (ppm)

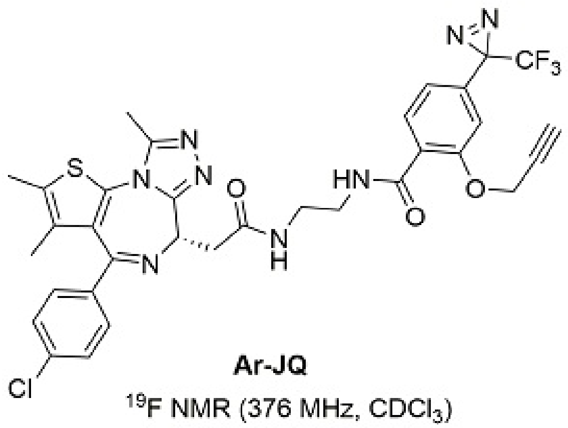

Ar-JQ

^{19}F NMR (376 MHz, $CDCl_3$)

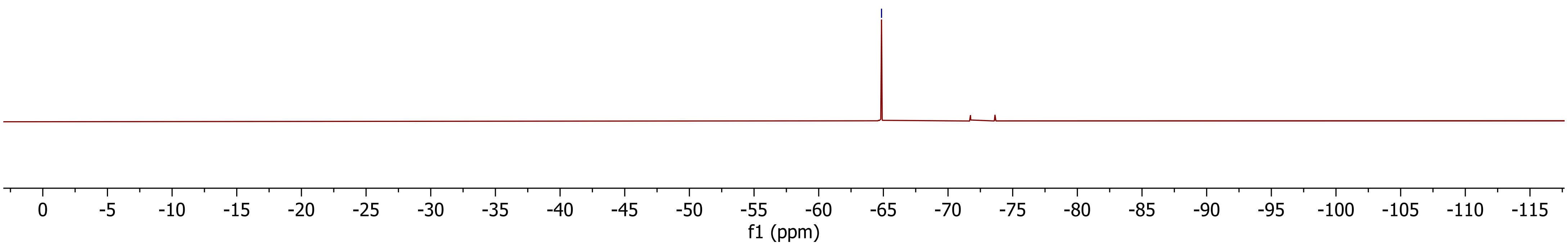

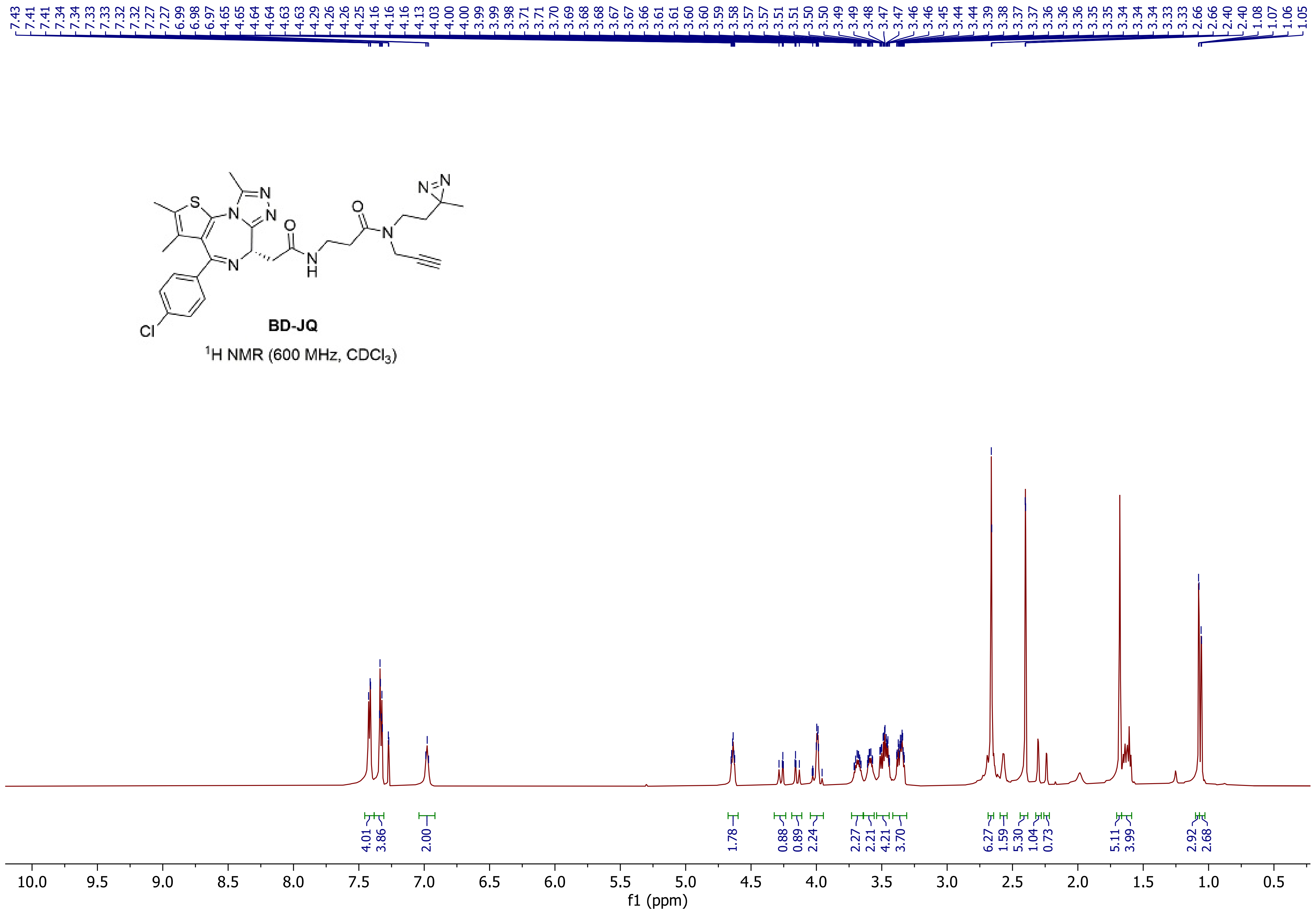
7.43 7.41 7.41 7.34 7.34 7.33 7.33 7.32 7.32 7.27 7.27 6.99 6.98 6.97 4.65 4.65 4.64 4.64 4.63 4.63 4.29 4.26 4.26 4.25 4.16 4.16 4.16 4.13 4.03 4.00 4.00 3.99 3.99 3.98 3.71 3.71 3.70 3.69 3.68 3.68 3.67 3.67 3.66 3.61 3.61 3.60 3.60 3.59 3.58 3.57 3.57 3.51 3.51 3.50 3.50 3.49 3.49 3.48 3.47 3.47 3.46 3.46 3.45 3.44 3.44 3.39 3.38 3.37 3.37 3.36 3.36 3.36 3.35 3.35 3.34 3.34 3.34 3.33 3.33 2.66 2.66 2.40 2.40 1.08 1.07 1.06 1.05
BD-JQ
^{1}H NMR (600 MHz, CDCl$_3$)
4.01 3.86 2.00 1.78 0.88 0.89 2.24 2.27 2.21 4.21 3.70 6.27 1.59 5.30 1.04 0.73 5.11 3.99 2.92 2.68
10.0 9.5 9.0 8.5 8.0 7.5 7.0 6.5 6.0 5.5 5.0 4.5 4.0 3.5 3.0 2.5 2.0 1.5 1.0 0.5
f1 (ppm)

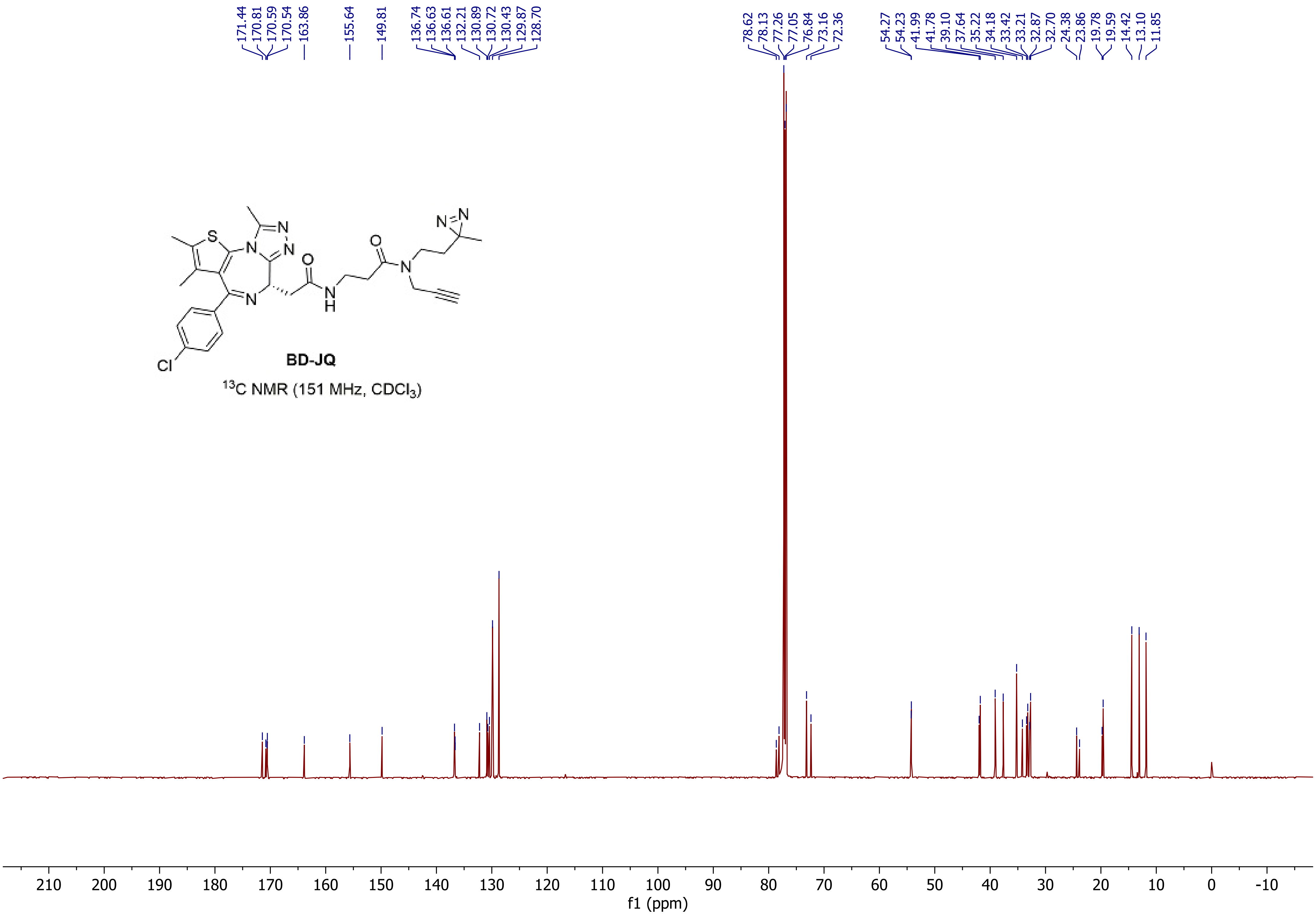
171.44
170.81
170.59
170.54
163.86
155.64
149.81
136.74
136.63
136.61
132.21
130.89
130.72
130.43
129.87
128.70
78.62
78.13
77.26
77.05
76.84
73.16
72.36
54.27
54.23
41.99
41.78
39.10
37.64
35.22
34.18
33.42
33.21
32.87
32.70
24.38
23.86
19.78
19.59
14.42
13.10
11.85
S
N
N
N
N
O
N
H
O
N
N
N
Cl
BD-JQ
13C NMR (151 MHz, CDCl3)
210
200
190
180
170
160
150
140
130
120
110
100
90
80
70
60
50
40
30
20
10
0
-10
f1 (ppm)

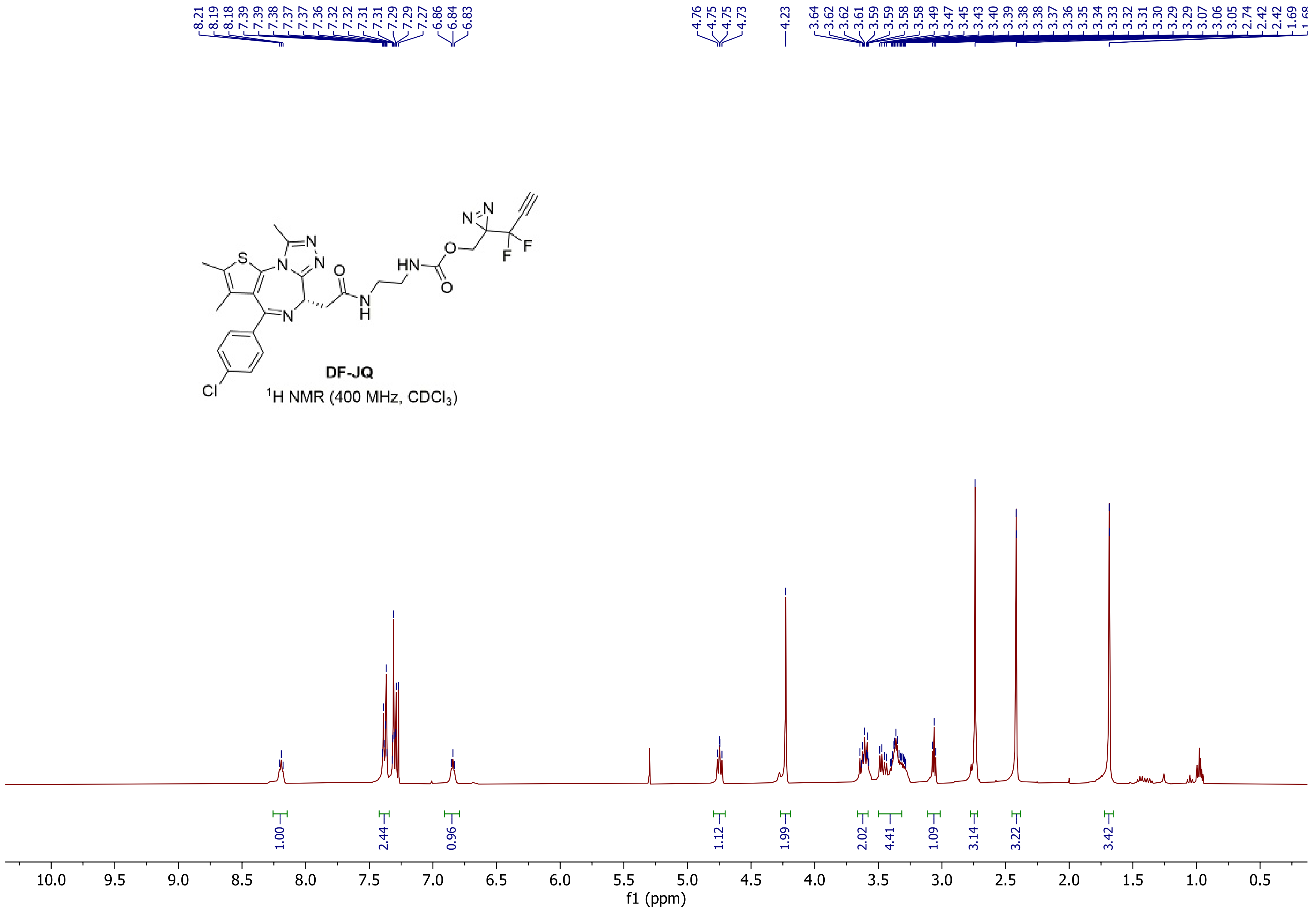
8.21 8.19 8.18 7.39 7.39 7.38 7.37 7.37 7.36 7.32 7.32 7.31 7.31 7.29 7.29 7.27 6.86 6.84 6.83
4.76 4.75 4.75 4.73
4.23
3.64 3.62 3.62 3.61 3.59 3.59 3.58 3.58 3.49 3.47 3.45 3.43 3.40 3.39 3.38 3.38 3.37 3.36 3.35 3.34 3.33 3.32 3.31 3.30 3.29 3.29 3.07 3.06 3.05 2.74 2.42 2.42 1.69
DF-JQ
^{1}H NMR (400 MHz, $CDCl_3$)
1.00 2.44 0.96 1.12 1.99 2.02 4.41 1.09 3.14 3.22 3.42
10.0 9.5 9.0 8.5 8.0 7.5 7.0 6.5 6.0 5.5 5.0 4.5 4.0 3.5 3.0 2.5 2.0 1.5 1.0 0.5
f1 (ppm)

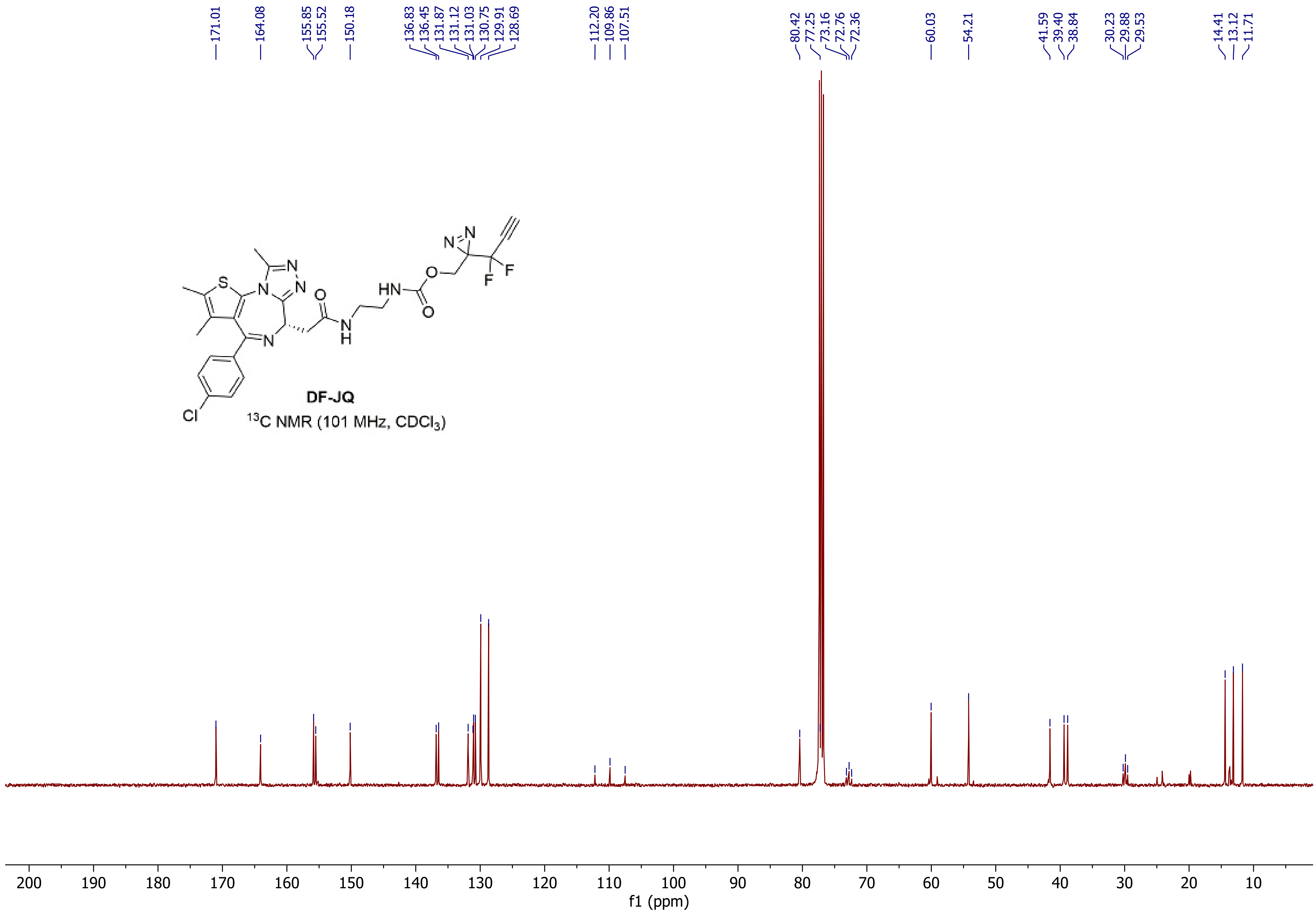

171.01
164.08
155.85
155.52
150.18
136.83
136.45
131.87
131.12
131.03
130.75
129.91
128.69
112.20
109.86
107.51
80.42
77.25
73.16
72.76
72.36
60.03
54.21
41.59
39.40
38.84
30.23
29.88
29.53
14.41
13.12
11.71
DF-JQ
^{13}C NMR (101 MHz, CDCl$_3$)
200
190
180
170
160
150
140
130
120
110
100
90
80
70
60
50
40
30
20
10
f1 (ppm)

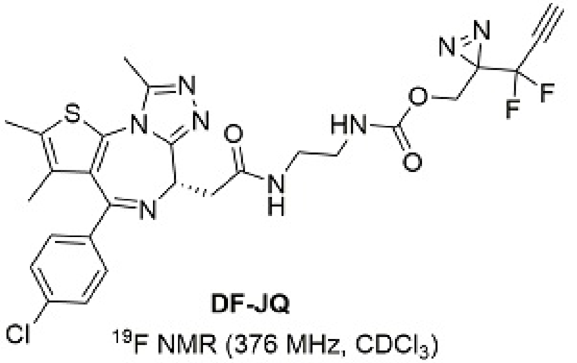

DF-JQ

^{19}F NMR (376 MHz, $CDCl_3$)

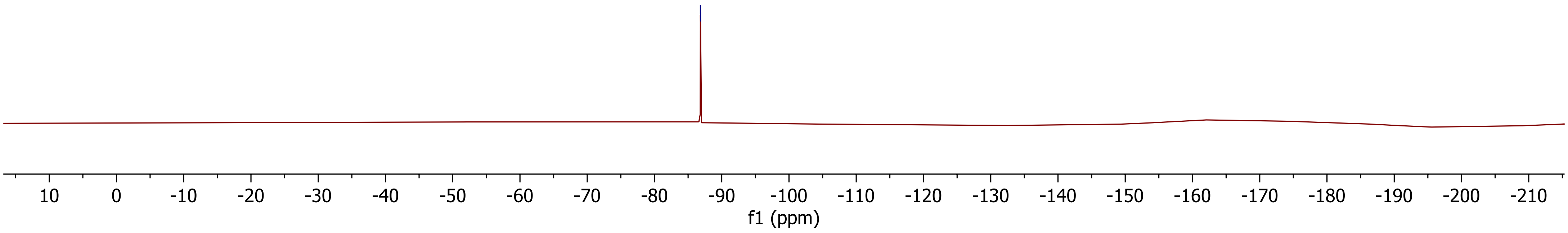

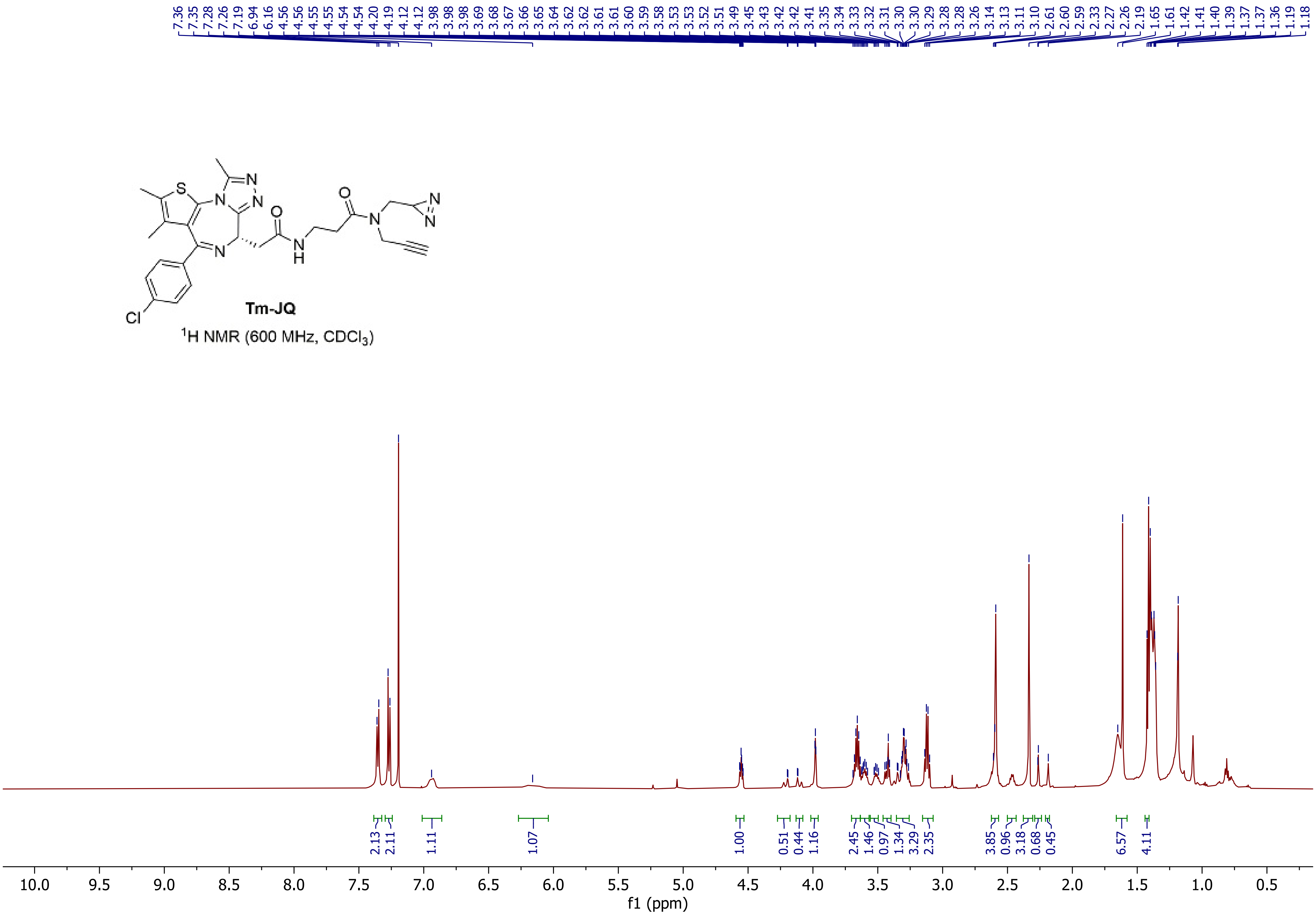
7.36 7.35 7.28 7.26 7.19 6.94 6.16 4.56 4.56 4.55 4.55 4.54 4.54 4.20 4.19 4.12 4.12 3.98 3.98 3.98 3.69 3.68 3.67 3.66 3.65 3.64 3.62 3.62 3.61 3.61 3.60 3.59 3.58 3.53 3.53 3.52 3.51 3.49 3.45 3.43 3.42 3.42 3.41 3.35 3.34 3.33 3.32 3.31 3.30 3.30 3.29 3.28 3.28 3.26 3.14 3.13 3.11 3.10 2.61 2.60 2.59 2.33 2.27 2.26 2.19 1.65 1.61 1.42 1.41 1.40 1.39 1.37 1.37 1.36 1.19 1.18
Tm-JQ
1H NMR (600 MHz, CDCl3)
2.13 2.11 1.11 1.07 1.00 0.51 0.44 1.16 2.45 1.46 0.97 1.34 3.29 2.35 3.85 0.96 3.18 0.68 0.45 6.57 4.11
10.0 9.5 9.0 8.5 8.0 7.5 7.0 6.5 6.0 5.5 5.0 4.5 4.0 3.5 3.0 2.5 2.0 1.5 1.0 0.5
f1 (ppm)

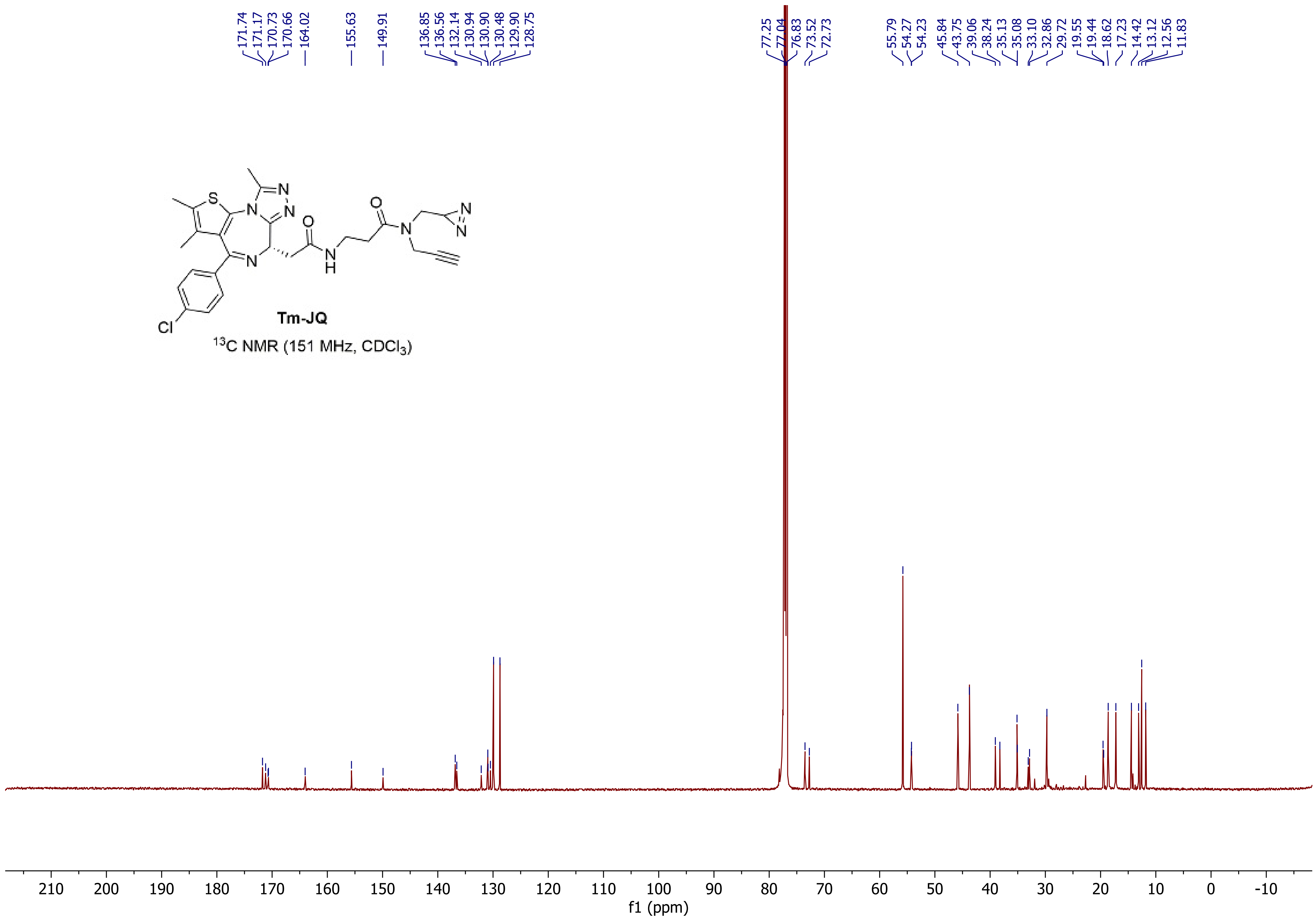
171.74
171.17
170.73
170.66
164.02
155.63
149.91
136.85
136.56
132.14
130.94
130.90
130.48
129.90
128.75
77.25
77.04
76.83
73.52
72.73
55.79
54.27
54.23
45.84
43.75
39.06
38.24
35.13
35.08
33.10
32.86
29.72
19.55
19.44
18.62
17.23
14.42
13.12
12.56
11.83
Tm-JQ
13C NMR (151 MHz, CDCl3)
210 200 190 180 170 160 150 140 130 120 110 100 90 80 70 60 50 40 30 20 10 0 -10
f1 (ppm)
